# HnRNPA2B1 ISGylation Regulates m6A‐Tagged mRNA Selective Export via ALYREF/NXF1 Complex to Foster Breast Cancer Development

**DOI:** 10.1002/advs.202307639

**Published:** 2024-04-16

**Authors:** Ting Jin, Liping Yang, Chao Chang, Haojun Luo, Rui Wang, Yubi Gan, Yan Sun, Yuetong Guo, Rui Tang, Shanchun Chen, Die Meng, Peijin Dai, Manran Liu

**Affiliations:** ^1^ Key Laboratory of Laboratory Medical Diagnostics, Chinese Ministry of Education Chongqing Medical University Chongqing 400016 China; ^2^ Department of Laboratory Medicine the Second Affiliated Hospital of Chongqing Medical University Chongqing 400010 China; ^3^ Department of Breast and Thyroid Surgery the Second Affiliated Hospital of Chongqing Medical University Chongqing 400010 China; ^4^ Department of Cell Biology and Medical Genetics, Basic Medical School Chongqing Medical University Chongqing 400016 China

**Keywords:** HnRNP2A2B1, ISG15, m6A mRNA, mRNA export

## Abstract

Regulating nuclear export precisely is essential for maintaining mRNA homeostasis and impacts tumor progression. However, the mechanisms governing nuclear mRNA export remain poorly elucidated. Herein, it is revealed that the enhanced hypoxic long no‐ncoding RNA (lncRNA prostate cancer associated transcript 6 (PCAT6) in breast cancer (BC) promotes the nuclear export of m6A‐modified mRNAs, bolstering breast cancer stem cells (BCSCs) stemness and doxorubicin resistance. Clinically, hypoxic PCAT6 correlates with malignant BC features and poor prognosis. Mechanically, PCAT6 functions as a scaffold between interferon‐stimulated gene 15 (ISG15) and heterogeneous nuclear ribonucleoprotein A2/B1 (hnRNPA2B1), leading to ISGylation of hnRNPA2B1, thus protecting hnRNPA2B1 from ubiquitination‐mediated proteasomal degradation. Interestingly, as an m6A reader, hnRNPA2B1 selectively mediates m6A‐tagged mRNAs nuclear export via the Aly/REF export factor (ALYREF)/ nuclear RNA export factor 1 (NXF1) complex, which promotes stemness‐related genes expression. HnRNPA2B1 knockdown or mRNA export inhibition can result in the retention of nuclear m6A‐tagged mRNA associated with stemness maintenance, which suppresses BCSCs self‐renewal and effectively improves the efficacy of doxorubicin therapy. These findings demonstrate the pivotal role of m6A‐modified mRNA nuclear export in BC progression, highlighting that the inhibition of m6A‐tagged mRNA and its nuclear export is a potential therapeutic strategy for the amelioration of cancer chemotherapy.

## Graphical abstract

Illustration of the underlying mechanism by which hypoxic PCAT6‐mediated hnRNPA2B1 ISGylation promotes BC progression. This process culminates in the stabilization of HnRNPA2B1, enhancing the FGFR1 mRNA nuclear export, which is subjected to N6‐methyladenosine (m6A) modifications.

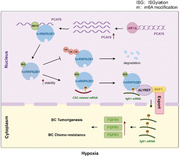

## 1 Introduction

Breast cancer (BC) is the most prevalent malignancy among women worldwide. With increasing annual morbidity, it represents a major threat to women health.^[^ ^1^ ^]^ Despite advancements in surgery, radiotherapy, chemotherapy, endocrine therapy, and immunotherapy, numerous BC patients experience therapeutic resistance, a key factor contributing to treatment failure and disease recurrence. Breast cancer stem cells (BCSCs), a subpopulation of tumor cells that undergo asymmetric cellular division for proliferation and differentiation, are primarily responsible for therapeutic resistance.^[^ ^2^ ^]^ Therefore, enhancing our understanding of the regulatory mechanism between therapeutic resistance and BCSCs stemness maintenance is crucial for improving BC therapy outcomes.

A hypoxic microenvironment, common in solid tumors, significantly affects the activation of drug resistance mechanisms.^[^ ^3^, ^4^ ^]^ Hypoxia impacts drug resistance through multiple mechanisms, including gene expression regulation, post‐translational modifications, and metabolic alterations.^[^ ^5^, ^6^, ^7^ ^]^ Cancer stem‐like cells (CSCs) exhibit drug resistance. Tumor hypoxia promotes CSCs stemness characteristics via hypoxia‐inducible factors (HIFs) and other signaling pathways, such as the Notch, Wnt, and JAK‐STAT.^[^ ^8^ ^]^ Whether this hypoxic enhances chemotherapy resistance by inducing stemness remains unclear. Our previous study identified hypoxia‐related lncRNAs, including the novel hypoxic lncRNA KB‐1980E6.3, which maintains BCSCs stemness by stabilizing c‐MYC mRNA.^[^ ^9^ ^]^ Here, we found a strong correlation between hypoxia‐induced lncRNA PCAT6 expression, BCSC stemness, and BC drug resistance. Exploring the specific molecular mechanisms is imperative for understanding hypoxia‐mediated resistance.

LncRNAs play crucial roles in regulating protein functions, including modulating activity, altering localization, and forming RNA‐protein complexes within subcellular structures.^[^ ^10^, ^11^, ^12^ ^]^ They influence cancer progression by affecting post‐translational modifications such as ubiquitination,^[^ ^13^ ^]^ phosphorylation,^[^ ^14^ ^]^ and acetylation.^[^ ^15^ ^]^ However, the interaction mechanism between lncRNAs and ubiquitin‐like modifications, including SUMOylation, neddylation, and ISGylation, remains poorly understood. Unraveling lncRNAs’ role in protein modifications could offer deeper insights into tumor development and cellular vulnerabilities.

Efficient genetic information transmission relies on precise mRNA export from the nucleus and its strict regulation. Malignant cancer cell processes require rapid and robust export of RNA‐encoding proteins from the nucleus. The export machinery encompasses mRNA export factors comprising recognition adaptors^[^ ^16^, ^17^ ^]^ and transporting factors, facilitating rapid mRNA passage through the nuclear envelope channel. Recent studies have highlighted dysregulation of mRNA export factors in human cancers.^[^ ^18^ ^]^ For instance, elevated expression of the export adaptor hTREX84 in BC cells enhances mRNA export and cellular proliferation.^[^ ^19^ ^]^ Nuclear PI3K signaling orchestrates cell proliferation through ALYREF‐mediated mRNA export functions.^[^ ^20^ ^]^ Furthermore, RNA modifications, particularly N6‐methyladenosine (m6A), are essential for RNA biology, including export.^[^ ^21^ ^]^ For example, m6A‐dependent mRNA nuclear export is vital for neural and osteoclast differentiation.^[^ ^22^, ^23^ ^]^ However, the mechanisms of m6A‐dependent mRNA nuclear export in malignant neoplasms remain elusive. We observed varied distribution of m6A‐tagged mRNAs in malignant BC cells, indicating a potential role of m6A modification in mRNA nuclear export. This suggests that m6A‐mediated mRNA nuclear export contributes to cancer progression and therapeutic resistance.

Herein, we found that the hypoxic lncRNA PCAT6 was highly expressed in BC and was strongly associated with the malignant progression of BC and the development of chemotherapeutic resistance. The ISG15‐mediated ISGylation of hnRNPA2B1 resulted in the stability and upregulation of hnRNPA2B1 proteins, crucial for the malignant progression of BC under hypoxia. Interestingly, the ISGylated‐hnRNPA2B1 governed the nuclear export of mRNA, especially CSC‐related mRNA, via the ALYREF/NXF1 complex in an m6A‐dependent manner, which is essential for BCSCs stemness maintenance and doxorubicin resistance. Targeting BC cells with the m6A inhibitor STM2457 combined with shRNA against PCAT6 or hnRNPA2B1 effectively inhibited the nuclear export of m6A‐tagged mRNA and ameliorated the malignant phenotypes of BCSCs. These novel findings will be beneficial for understanding tumor progression, chemotherapeutic resistance, and the pathobiology of m6A‐modified mRNA in BC.

## 2 Results

### 2.1 Hypoxic PCAT6 is Related with BC Progression and Poor Prognosis

The hypoxic microenvironment confers tumors with more aggressive properties, including proliferation, metastasis, and chemoresistance. We previously identified a series of hypoxia‐related lncRNAs in BC. To explore the potential relationship between hypoxia‐specific lncRNAs and tumor drug resistance, we reanalyzed our previous non‐coding RNA expression profiles in BT549 cells under normoxia and hypoxia (1% O_2_) (Figure S1a, Supporting Information). A total of 974 lncRNAs were upregulated, whereas 1366 lncRNAs were downregulated in hypoxic BT549 cells compared to normoxic BT549 cells (Figure S1a, Supporting Information). To further elucidate the potential of hypoxic microenvironment‐induced chemotherapy resistance especially doxorubicin resistance, we synthetically analyzed hypoxic BT549 cell RNA‐sequencing (RNAseq) and doxorubicin‐resistant MCF7 cells from GEO datasets.^[^ ^24^ ^]^ Additionally, 23 lncRNAs were identified in hypoxic‐ and doxorubicin‐resistant cells (Figure S1b, Supporting Information). PCAT6 was the most upregulated lncRNA in BC cells under hypoxia and doxorubicin treatment (Figure S1c,d, Supporting Information). Enhanced PCAT6 expression was further verified in a panel of hypoxic BC cells (Figure S1e, Supporting Information) and doxorubicin‐resistant BC cells (Figure S1f, Supporting Information), with PCAT6 expression levels exhibiting a dependency on doxorubicin dosage (Figure S1g, Supporting Information). Hence, high hypoxia‐specific PCAT6 expression may be associated with BC drug resistance.

As HIF‐1α is a key transcriptional regulator in hypoxia, we evaluated whether PCAT6 was regulated by HIF‐1α. After evaluating PCAT6 expression in lentivirus‐mediated HIF‐1α knockdown and control BC cells during hypoxia, a significant reduction in PCAT6 expression was observed upon the depletion of HIF1α (Figure S1h, Supporting Information). Additionally, analysis using Ensemble (http://asia.ensembl.org/index.html/) and JASPAR (https://jaspar.genereg.net/) databases revealed the presence of a hypoxia response element (HRE) (−162 to −148 bp) in the PCAT6 promoter region (Figure S1i, Supporting Information). Thus, we constructed pGL3‐PCAT6 wild‐type (WT) and pGL3‐PCAT6 mutant (MUT) reporter to assess the transcriptional regulation of HIF‐1α on PCAT6 using dual luciferase reporter assay. Upon transfection of pGL3‐PCAT6 WT or pGL3‐PCAT6 MUT reporter into HIF‐1α WT BT549 and HIF‐1α‐deficient cells, high luciferase activity of PCAT6 was detected only in the HIF‐1α wild type BT549 cells but not HIF‐1α‐deficient cells under hypoxia (Figure S1j, Supporting Information). A chromatin immunoprecipitation assay further confirmed that HIF‐1α could directly bind to the HRE site of the PCAT6 promoter in hypoxic BC cells (Figure S1k, Supporting Information). Thus, hypoxic PCAT6 is an HIF‐1α‐induced lncRNA and potentially involved in doxorubicin resistance in BC cells.

**Figure 1:**
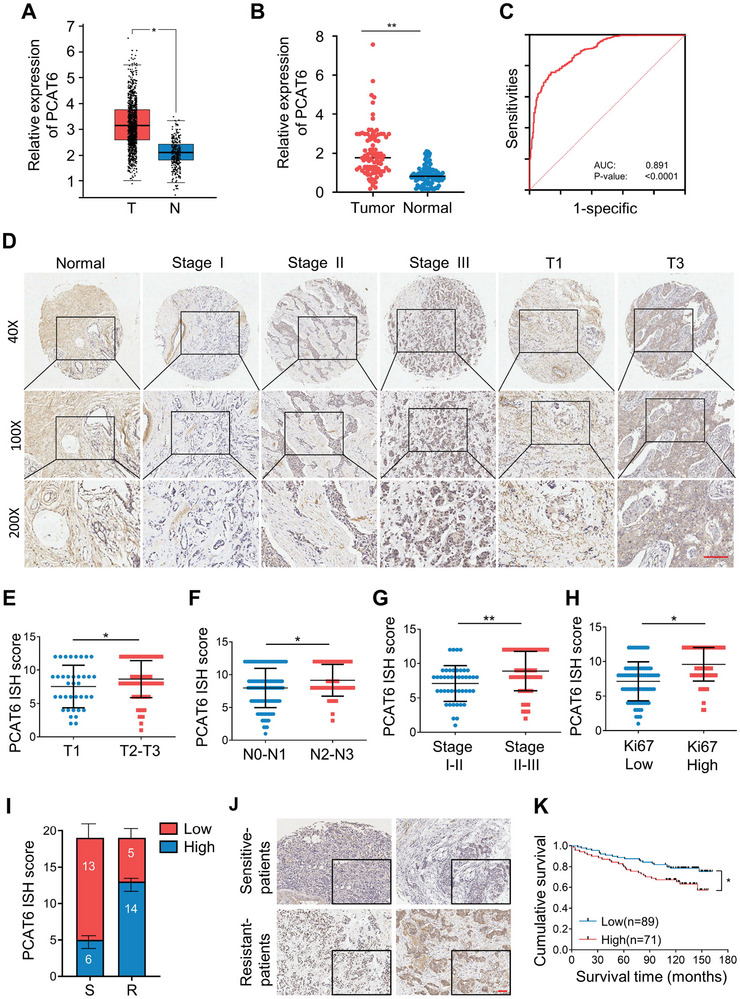
High level of hypoxic lncRNA PCAT6 is associated with breast cancer progression and chemotherapy resistance. A) PCAT6 expression levels in BC tissues (T) and adjacent non‐tumor tissues (N) from TCGA data sets. B) PCAT6 expression levels in 95 paired breast tumor tissues and adjacent non‐cancerous tissues. C) ROC curve of TCGA datasets and our 95 paired breast tumor tissues. D) Representative ISH images of PCAT6 expression in 160 BC tissues. Scale bar, 200 µm. E–H) Dot distribution graphs depicting PCAT6 IHC staining scores of 160 BC patients across different clinical T (E), N (F) stages, clinical grade (G), and Ki67 scores (H). I) ISH staining scores were calculated in chemotherapy resistant (R) and sensitive (S) BC samples (n = 38). ISH staining score > 8 was defined as high expression, and a score ≤ 8 was regarded as low expression. J) Representative ISH images of PCAT6 expression in doxorubicin resistant (R) and sensitive (S) BC samples. Scale bar, 100 µm. K) Kaplan–Meier survival curve of BC patients with high or low PCAT6 expression based on ISH staining score of 160 BC patients. (^*^ *P* < 0.05, ^**^ *P* < 0.01.).

To acquire insights into the clinical significance and expression patterns of PCAT6 in BC, we analyzed RNA‐seq data from The Cancer Genome Atlas (TCGA). There was an obvious increased PCAT6 in BC tissues compared to that in adjacent normal tissues (**Figure** **1**a), which was further confirmed in our cohort of 94 paired clinical BC specimens (Figure 1b). Receiver operating characteristic curve analysis performed on TCGA datasets, and our clinical BC cohort further supported the finding that PCAT6 might serve as a diagnostic marker for BC patients (Figure 1c). To expand our findings, the relationship between PCAT6 expression and BC clinical characteristic was analyzed using a human tissue microarray (TMA) containing 160 BC tissues via in situ hybridization (ISH). The TMA data showed that BC patients with high levels of PCAT6 showed worse clinical progress (Figure 1d), with a higher pathological grade (P = 0.0026) (**Table** **1**). These findings suggest that increased tumor size, lymph node metastasis, advanced disease staging, and higher ki67 histopathological score in BC were closely correlated with higher levels of PCAT6 (Figure 1e–h). In particular, enhanced PCAT6 expression in chemotherapy‐resistant breast cancers further supported that PCAT6 is a tumor‐promoting lncRNA (Figure 1i,j; Table S3, Supporting Information). Indeed, BC patients with enhanced PCAT6 expression had shorter overall survival than those with lower PCAT6 expression (Figure 1k). Summarily, PCAT6 plays an oncogenic and drug‐resistant role, and a high level of PCAT6 predicts poor prognosis in BC patients.

**Table 1:** Correlation between PCAT6 expression and clinicopathological features in 160 TNBC patients.

Characteristic		All case	PCAT6		Chi‐square	*P* value
			Low	High		
Age	<50	71	43	28	1.9700	0.1604
	≥50	89	44	45		
Grade	I‐II	45	33	12	9.0710	0.0026**
	II‐III	115	54	61		
T stage	T1	37	20	17	0.0018	0.9663
	T2‐3	123	66	57		
N stage	N0	63	33	30	0.1666	0.6832
	N1‐3	97	54	43		
TNM stage	I‐II	111	59	52	0.2181	0.6405
	III	49	28	21		

### 2.2 Nuclear Export of CSC‐Associated m6A‐Tagged RNAs is Crucial for BCSC Maintenance

For a comprehensive understanding of PCAT6's role in tumor progression, transcriptomic profiling of PCAT6 WT (Hs578T/shNC) and PCAT6 deficient cells (Hs578T/shPCAT6) under hypoxia was performed using RNA‐seq. A total of 3307 altered genes, including 630 upregulated and 2677 downregulated genes, were identified in PCAT6 knocked down Hs578T cells (false discovery rate <0.05; log CPM >0; fold change >4; Figure S2a, Supporting Information). The altered genes were mainly involved in the biological processes of tumorigenesis, maintenance of CSC stemness, and regulation of gene expression, which were possibly governed by signaling pathways related to mRNA surveillance and RNA transport (Figure S2b, Supporting Information). Gene ontology (GO) analysis revealed that these differentially expressed genes were involved in mRNA processing (Figure S2c, Supporting Information). As the most abundant internal marker of eukaryotic mRNA, m6A modification can functionally affect RNAs biogenesis; therefore, we screened m6A‐associated regulators and found that nine m6A regulators (WTAP, ZC3H13, IGF2BP2, IGF2BP3, FMR1, LRPPRC, YTHDC1, YTHDC2, YTHDF3) were decreased in PCAT6 knockdown cells compared to control cells (Figure S2d, Supporting Information).

**Figure 2:**
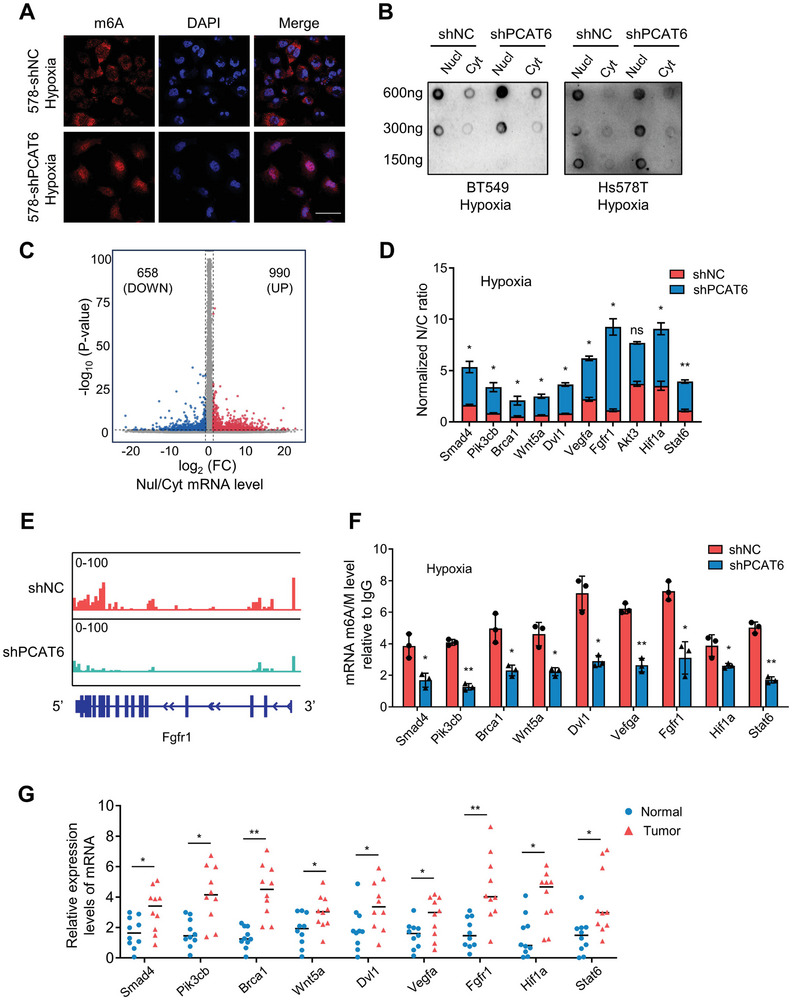
CSC‐associated m6A‐tagged RNA export from nuclear is crucial for BCSCs maintenance. A) m6A mRNA levels in PCAT6 knockdown and control BC cells were detected via confocal immunofluorescent assay. Scale bar, 50 µm. B) The nuclear and cytoplasmic m6A mRNA levels in PCAT6 knockdown and control BC cells under hypoxia were detected by conducting dot blotting using an anti‐m6A antibody. The total RNAs (600 ng, 300 ng, 150 ng) were spotted onto a Hybond‐N^+^ membrane. C) Volcano plot showing the ratio of nuclear and cytoplasmic mRNA levels from RNA‐seq. D) The relative nuclear (labeled as N) and cytoplasmic (labeled as C) mRNA ratio in shNC/Hs578T and shPCAT6/Hs578T cells under hypoxia were detected with qRT‐PCR. The data are represented as mean ± SD. E) Integrative genomics viewer tracks showing the m6A peak distribution in the mRNA transcript derived from MeRIP‐seq data in PCAT6 knockdown and control BC cells. F) Me‐RIP PCR was performed to detect m6A levels of mRNA in PCAT6 knockdown and control BC cells under hypoxia. G) mRNAs expression levels in 10 paired BC tissues and adjacent non‐cancerous tissues. (ns, no significant; *p < 0.05, **p < 0.01).

Next, we analyzed the m6A status of RNAs in PCAT6 deficiency and control BC cells by performing dot blots and immunofluorescence under hypoxia. Surprisingly, there was no difference in the total m6A levels among these cells, but significant nuclear accumulation of m6A‐tagged mRNAs was detected in hypoxic PCAT6 knockdown cells (**Figure** **2**a,b; Figure S2e, Supporting Information). We also found that PCAT6 had no effect on methyltransferase‐like 3 (METTL3) and methyltransferase‐like 14 (METTL14) expression (Figure S2f, Supporting Information), two catalytic cores in the methyltransferase (MTase) complex responsible for m6A installation.^[^ ^25^ ^]^ These suggested that PCAT6's influence on m6A‐modified mRNA distribution was not mediated via the MTase complex but probably via other mechanisms. To determine whether PCAT6 affects m6A‐tagged mRNA subcellular localization, we performed methylated RNA immunoprecipitation sequencing (MeRIP‐seq) to compare the m6A peak in PCAT6 deficiency (Hs578T/shPCAT6) and control Hs578T (Hs578T/shNC) cells under hypoxia (Figure S2g, Supporting Information). To further determine the subcellular distribution of m6A‐modified RNAs, RNAs isolated from the nucleus and cytoplasm was used for RNA‐seq (Figure 2c; Figure S2h, Supporting Information). We performed a comprehensive analysis of nuclear/cytoplasmic RNA‐seq data and meRIP‐seq data from PCAT6 knockdown and control BC cells and identified 174 m6A‐modified mRNAs that clustered in the nuclear of PCAT6 knockdown Hs578T (Figure S2i, Supporting Information), in which pluripotency‐ and tumorigenesis‐related signaling pathways (e.g., Wnt, PI3K‐AKT, and pluripotency of stem cell regulatory signaling) were significantly enriched (Figure S2j, Supporting Information). After qRT‐PCR analysis, nine m6A‐tagged mRNAs (Smad4, Pik3cb, Brca1, Wnt5a, Dvl1, Vegfa, Fgfr1, Hif1a, and Stat6) associated with pluripotency‐ and tumorigenesis‐related signaling were highly accumulated in PCAT6‐deficient BC nuclei (Figure 2d), compared to those in control BC cells under hypoxia. As shown in Figure S2k (Supporting Information), most of the listed mRNA showed no significant changes in nuclear accumulation, while the others (Wnt5a, Dvl1, Fgfr1) showed slightly increased nuclear accumulation in PCAT6‐ deficient BC cells under normoxia. In addition, PCAT6 overexpression in MDA‐MB‐231 cells led to significant nuclear export of pluripotency‐ and tumorigenesis‐related mRNAs under normoxia (Figure S2l, Supporting Information). The meRIP‐seq and meRIP‐PCR of m6A‐tagged mRNAs (Smad4, Pik3cb, Brca1, Wnt5a, Dvl1, Vegfa, Fgfr1, Hif1a, and Stat6) also showed a significant m6A peak fold change between PCAT6 knockdown and control cells (Figure 2e,f; Figure S2m, Supporting Information). The decrease in proteins related to stemness maintenance (HIF‐1α, VEGFA, BRCA1, WNT5a, FGFR1, STAT6, SMAD4, and DVL1) in PCAT6 knockdown cells further supports the critical role of PCAT6 in regulating protein expression via m6A‐tagged mRNA nuclear export (Figure S2n, Supporting Information). Moreover, these m6A‐tagged genes were highly expressed in clinical BC tissues (Figure 2g). Thus, PCAT6 was involved in regulating the nuclear export of m6A‐tagged mRNA in hypoxic BC cells to facilitate the malignant progression of breast tumors.

### 2.3 PCAT6 is Required for the Maintenance of BCSCs Properties

**Figure 3:**
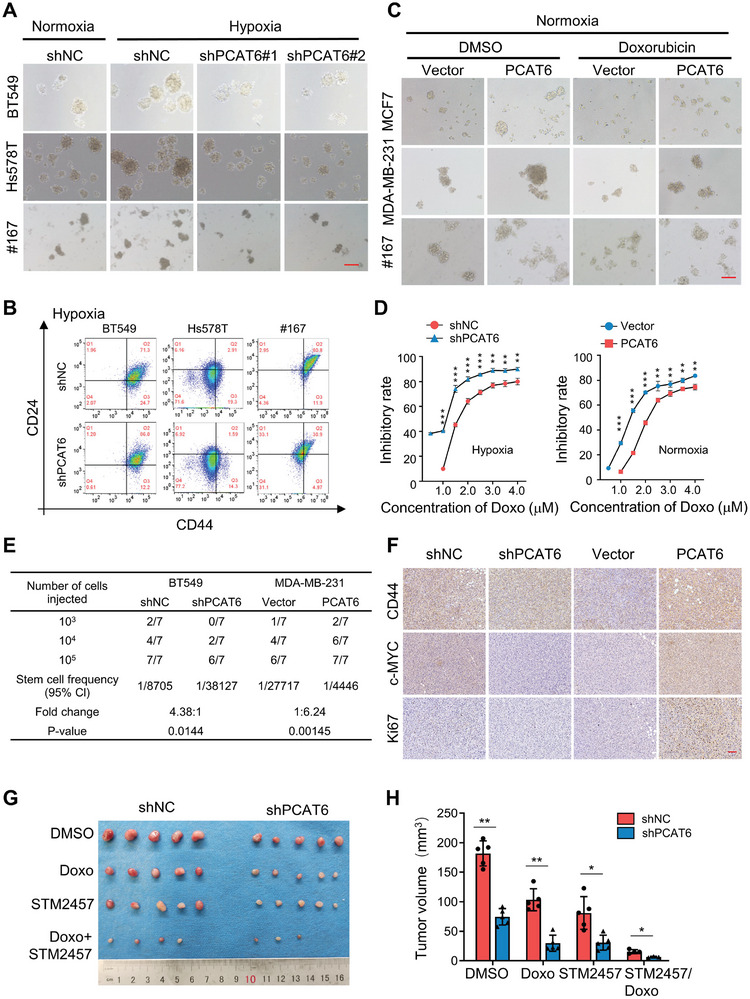
PCAT6 is required for the maintenance of BCSCs properties. A) Representative images of mammospheres in PCAT6 knockdown and control hypoxic BC cells. Original magnification, ×100. Scale bars, 100 µm. B) The proportion of CD44^+^/CD24^−^ BC cells was determined using flow cytometry. Data shown are representative of three or more independent experiments. C) Representative images of mammospheres in ectopic PCAT6 overexpressing BC cells under normoxia conditions. Original magnification, ×100. Scale bars, 100 µm. D) The doxorubicin dose‐dependent sensitivities of PCAT6 knockdown hypoxic BT549 cells or ectopic PCAT6 overexpressing MDA‐MB‐231 cells; their control cells were assessed via proliferation assay. The inhibitory rates are plotted as the fraction of dying cells relative to the number of untreated cells. The data are represented as mean ± SD. E) PCAT6‐silenced (shPCAT6#2) CSCs from hypoxic BT549 and PCAT6‐overexpressed non‐CSCs from MDA‐MB‐231 were diluted and subcutaneously implanted into BALB/c nude mice. Tumor initiation ratios were shown (N = 7 for each group). CSC frequency was calculated by performing the limiting dilution assay. CI: confidence interval. F) Representative images of CD44, c‐MYC, Ki67 IHC staining in each of xenograft tumor group (magnification, ×200. Scale bar, 100 µm). G‐H) PCAT6 knockdown decreased xenograft tumor growth in vivo; 1×10^6^ of PCAT knockdown or control BT549 sphere‐derived cells were injected subcutaneously into female BALB/c nude mice individually. After 20 days of injection, DMSO, doxorubicin (2 mg k^−1^g), STM2457 (50 mg k^−1^g) or doxorubicin combined with STM2457 was administered by intraperitoneal injection every 2 days, six times in total. G) Tumor sizes and H) volumes. (^*^ *p* < 0.05, ^**^ *p* < 0.01).

Hypoxic lncRNAs may be major factors in breast tumor development and cancer stemness maintenance.^[^ ^9^ ^]^ To ascertain whether PCAT6 promotes chemotherapy resistance by inducing CSC characteristics in BC cells, we examined the expression levels of PCAT6 in BCSC‐enriched mammospheres and parental BC cells. A significant increase in PCAT6 expression was identified in BCSCs‐enriched mammospheres, rather than in parent BC cells or monolayer‐cultured BCSCs (Figure S3a, Supporting Information), suggesting that enhanced hypoxic PCAT6 expression in BCSCs was required to maintain BC cell stemness. To test this hypothesis, engineered PCAT6 stable knockdown and overexpressing BC cells were established (Figure S3b,c, Supporting Information). The knockdown of PCAT6 notably decreased sphere formation (**Figure** **3**a; Figure S3d, Supporting Information), ratio of CD44^+^CD24^−^ cells (a well‐known CSC population in BC) (Figure 3b), and colony formation under hypoxia (Figure S3e,f, Supporting Information). In contrast, ectopic PCAT6 increased the stemness of BCSCs and led to the resistance of BC cells to doxorubicin‐induced growth inhibition and sphere formation inhibition (Figure 3c,d; Figure S3g–j, Supporting Information). However, PCAT6 knockdown BC cells were more sensitive to doxorubicin than control cells in vitro (Figure 3d). To further explore the role of PCAT6 in BCSCs tumorigenesis, nude mice were inoculated with sphere‐derived cells. In vivo limiting dilution assays revealed that the loss of PCAT6 dramatically reduced tumor incidence (Figure 3e), and reduced CD44^+^ breast CSCs, c‐MYC, and Ki67 were detected in PCAT6 knockdown cell‐formed xenografts compared with control xenografts (Figure 3f). Conversely, ectopic PCAT6 overexpression markedly increased tumor incidence and CD44^+^ breast CSCs, c‐Myc, and Ki67 expression in xenografts (Figure 3e,f). Furthermore, PCAT6 knockdown decreased xenograft tumor growth in mice (Figure 3g,h), and tumor growth of xenografts derived from shPCAT6 spheres was severely suppressed by doxorubicin treatment (Figure 3g,h). Thus, PCAT6 was required for the maintenance and self‐renewal of BCSCs, resulting in doxorubicin resistance in BC in vitro and in vivo.

### 2.4 ISG15 Stabilizes hnRNPA2B1 Protein via ISGylation and Ubiquitination Switch on hnRNPA2B1

**Figure 4:**
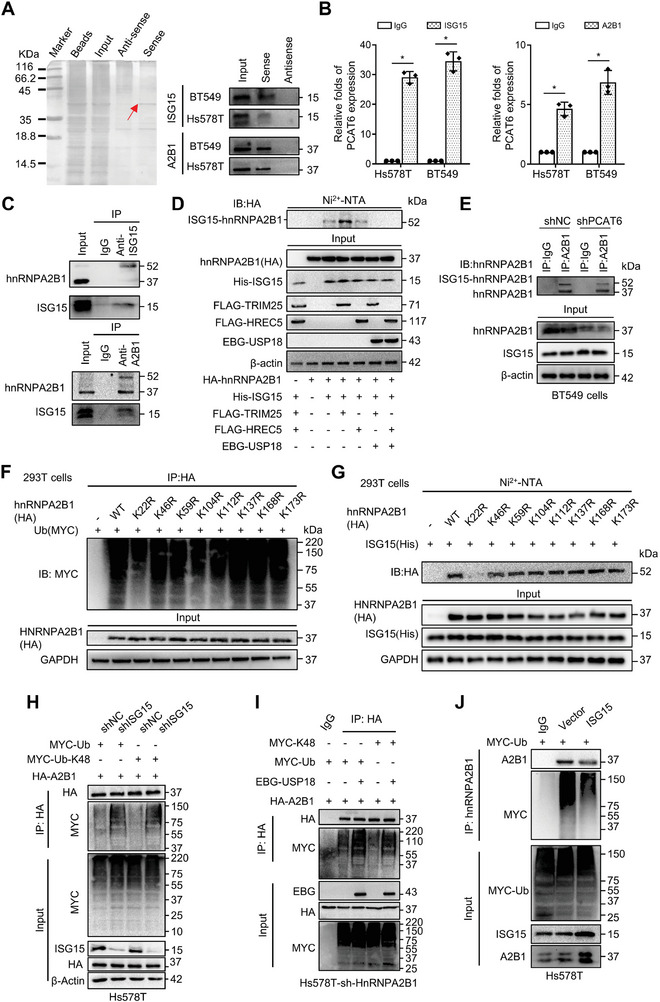
ISGylation and ubiquitination switch regulates hnRNPA2B1 stability. A) RNA pull‐down experiments were conducted using sense and antisense PCAT6, followed by silver staining. A significantly altered band is depicted. Western blotting assays confirmed the interaction of PCAT6 with ISG15 and hnRNPA2B1 (labeled as A2B1). B) RIP assays were conducted with spheres derived from Hs578T and BT549 cells using antibodies against ISG15 or hnRNPA2B1; IgG served as the negative control. The PCAT6 enriched in the RIP precipitates was analyzed with qRT‐PCR. C) Co‐IP showing the mutual binding between ISG15 and hnRNPA2B1 in hypoxic BC cells. D,E) Pull down‐western blotting to check ISGylation of HnRNPA2B1. In 293T cells, HA‐hnRNPA2B1 and His‐ISG15/Flag‐TRIM25 were transfected with or without EBG‐USP18 into HEK‐293T cells. ISG15‐conjugated proteins were captured using Ni^2+^‐NTA resin, followed by immunoblotting with anti‐HA antibody. Total cell lysates were immunoblotted with indicated antibodies. E) IP‐western blotting to check ISGylation of HnRNPA2B1. In PCAT6 knockdown and control BT549 cells, the lysates were precipitated with anti‐hnRNPA2B1 or normal IgG and immunoblotted with anti‐hnRNPA2B1 antibody. Total cell lysates were immunoblotted with indicated antibodies. F) Ubiquitination levels of hnRNPA2B1 were evaluated in HEK‐293T cells. HEK‐293T cells were transfected with point mutants of hnRNPA2B1 along with MYC‐Ub, as indicated. Ubiquitylation proteins were enriched with HA‐IP, and eluates were immunoblotted with anti‐MYC antibody. G) ISGylation levels of hnRNPA2B1 were evaluated in HEK‐293T cells. HEK‐293T cells were transfected with point mutants of hnRNPA2B1 along with hnRNPA2B1 tagged with His‐ISG15, as indicated. ISG15 conjugated proteins were enriched with Ni^2+^‐NTA resin, and eluates were immunoblotted with anti‐HA antibody. H,I) Total ubiquitination and K48‐linkage ubiquitination of hnRNPA2B1 were measured in H) ISG15‐knockdown and I) hnRNPA2B1‐knockdown. After transfection of HA‐hnRNPA2B1, MYC‐Ub, or MYC‐Ub‐K48, cell lysates were used for IP with anti‐HA antibody, followed by immunoblotting with anti‐HA and anti‐MYC antibodies, as indicated. Total cell lysates were immunoblotted with indicated antibodies. J) Total ubiquitination of hnRNPA2B1 was measured in ISG15 over‐expression Hs578T cells. After transfection of MYC‐Ub, cell lysates used for IP were incubated with anti‐hnRNPA2B1, followed by immunoblotting with anti‐hnRNPA2B1 (labeled as A2B1) and anti‐MYC antibodies, as indicated. Total cell lysates were immunoblotted with indicated antibodies. (^*^ *p* < 0.05, ^**^ *p* < 0.01).

Next, we investigated the role of PCAT6 in the export of stemness‐related mRNAs from the nucleus. LncRNAs exert their functions by interacting with specific binding proteins.^[^ ^26^ ^]^ To identify the potential binding proteins of PCAT6, RNA pull‐down coupled with mass spectrometry‐based analysis (LC‐MS/MS) was performed. ISG15, a ubiquitin‐like modifier,^[^ ^27^ ^]^ and hnRNPA2B1, an m6A reader,^[^ ^28^ ^]^ were enriched in the PCAT6 biotin‐labeled RNA pull‐down precipitates derived from hypoxic BT549 and Hs578T cells (**Figure** **4**a; Figure S4a and Table S5, Supporting Information). An RNA‐binding protein immunoprecipitation (RIP) assay using antibodies targeting ISG15 and hnRNPA2B1 showed significant enrichment of PCAT6 in the precipitates compared to the control precipitates (Figure 4b). Using deletion mapping and biotin‐labeled RNA pull‐down assays, we confirmed that the PCAT6 fragment transcribed from nucleotides 231–451 (Del2) could bind with ISG15 (Figure S4b, Supporting Information) and that transcribed from nucleotides 552–764 (Del4) could bind with hnRNPA2B1 (Figure S4b, Supporting Information). ISG15 and hnRNPA2B1 are bona fide interacting partners of PCAT6 in hypoxic BC cells.

LncRNAs act as scaffolders in regulating the stability, localization, and activity of binding proteins in the nucleus or cytoplasm in a dose‐dependent manner.^[^ ^29^ ^]^ To investigate whether PCAT6 could function as a scaffold for ISG15 and hnRNPA2B1, we confirmed the protein–protein interaction between ISG15 and hnRNPA2B1 in hypoxic tumor cells using a co‐immunoprecipitation (co‐IP) assay (Figure 4c). The ectopic expression of PCAT6 in BC cells dramatically promoted the interaction between ISG15 and hnRNPA2B1; however, PCAT6 knockdown in hypoxic BC cells reduced the binding between ISG15 and hnRNPA2B1 (Figure S4c, Supporting Information). As ISG15 acts as a key ubiquitin‐like modifier for protein ISGylation,^[^ ^30^ ^]^ we investigated whether hnRNPA2B1 could be ISGylated by ISG15 in hypoxic BC cells. As detected by Ni^2+^‐NTA resin pull‐down and western blotting, we found that hnRNPA2B1 underwent ISG15 modification. Tripartite moti‐fcontaining protein 25 (TRIM25), rather than HECT and RLD domain containing E3 ubiquitin‐ protein ligase 5 (HERC5), both being E3 enzymes of ISGylation, could increase the ISGylation of hnRNPA2B1 (Figure 4d). This was further confirmed by co‐IP (Figure S4d, Supporting Information). Furthermore, co‐transfection with ubiquitin‐specific peptidase 18 (USP18), an ISG15‐specific deubiquitinase,^[^ ^31^ ^]^ removed ISGylation from hnRNPA2B1 (Figure 4d). Moreover, ISG15‐conjugated hnRNPA2B1 markedly decreased upon PCAT6 knockdown and increased with ectopic PCAT6 (Figure 4e; Figure S4e–g, Supporting Information). Furthermore, the loss of PCAT6 resulted in a significant decrease in hnRNPA2B1 protein levels, which was rescued by ISG15 re‐expression (Figure S4h, Supporting Information). Hence, PCAT6 increases hnRNPA2B1 expression by regulating the ISGylation of hnRNPA2B1.

Given that ISGylation occurs at the same site or close to the ubiquitinated K site,^[^ ^27^ ^]^ we used the PhosphositePlus program (https://www.phosphosite.org/) to predict the major ubiquitylation sites in hnRNPA2B1. To screen for potential ubiquitylation sites, we individually mutated lysines (Ks) with a high ubiquitination potential ranking, namely K22, K46, K59, K104, K112, K137, K168, and K173, to arginine (R) among the candidate residues. The K22R almost abolished the ubiquitination of hnRNPA2B1 (Figure 4f), which also showed well conserved among vertebrates (Figure S4i, Supporting Information). Interestingly, K22 was also an ISGylated locus of hnRNPA2B1, as identified by ISGylation assays using Ni^2+^‐NTA pull‐down analysis (Figure 4g). Moreover, IP assays of Hs578T‐shPCAT6 cells transfected with HA‐hnRNPA2B1‐WT or ‐K22R together with His‐ISG15 and Flag‐TRIM25 confirmed that ISGylation of hnRNPA2B1‐K22R was notably reduced compared to that of hnRNPA2B1‐WT (Figure S4j, Supporting Information). Subsequently, hnRNPA2B1 WT or hnRNPA2B1 K22R MUT was transfected into hnRNPA2B1‐knockdown BC cells, and hnRNPA2B1 protein stability was evaluated through cycloheximide (CHX) chase analysis. The K22R MUT accelerated the degradation of the hnRNPA2B1 protein compared to that of hnRNPA2B1 WT (Figure S4k, Supporting Information). Hence, K22 is the major ISGylation site of hnRNPA2B1.

Next, we compared the effects of ubiquitination and ISGylation on hnRNPA2B1 protein stability. The loss of PCAT6 resulted in an obvious increase in hnRNPA2B1 ubiquitination (Figure S4l, Supporting Information). Given that K48‐linked ubiquitination is the most abundant and functionally well‐characterized polyubiquitin chain that leads to protein degradation,^[^ ^32^ ^]^ we determined whether the K48‐linked chain was present in polyubiquitinated hnRNPA2B1. The data showed that ubiquitination of hnRNPA2B1 is K48‐linked (Figure S4m, Supporting Information). Enhanced total and K48‐linked ubiquitination modifications of hnRNPA2B1 and reduced hnRNPA2B1 protein were also detected following ISG15 knockdown or transfection with USP18 (Figure 4h,i). However, ectopic ISG15 overexpression resulted in decreased ubiquitination of hnRNPA2B1 and higher protein levels of hnRNPA2B1 (Figure 4j). These findings suggested a potential competition between ISGylation and ubiquitination of hnRNPA2B1; ISGylation prevents hnRNPA2B1 from ubiquitination to maintain its stability. Immunohistochemical staining of the TMA of BC tissues further proved that there were significantly higher hnRNPA2B1 proteins in ISG15^high^ tumors and lower hnRNPA2B1 proteins in ISG15^low^ tissues (Figure S4n and Table S4, Supporting Information). Thus, ISG15 remarkably increased hnRNPA2B1 expression and inhibited hnRNPA2B1 ubiquitination in hypoxic BC cells and tumors.

### 2.5 HnRNPA2B1/ALYREF Bridges CSC‐Related m6A mRNA Selective Export from Nuclear via ALYREF/NXF1 Complex

**Figure 5:**
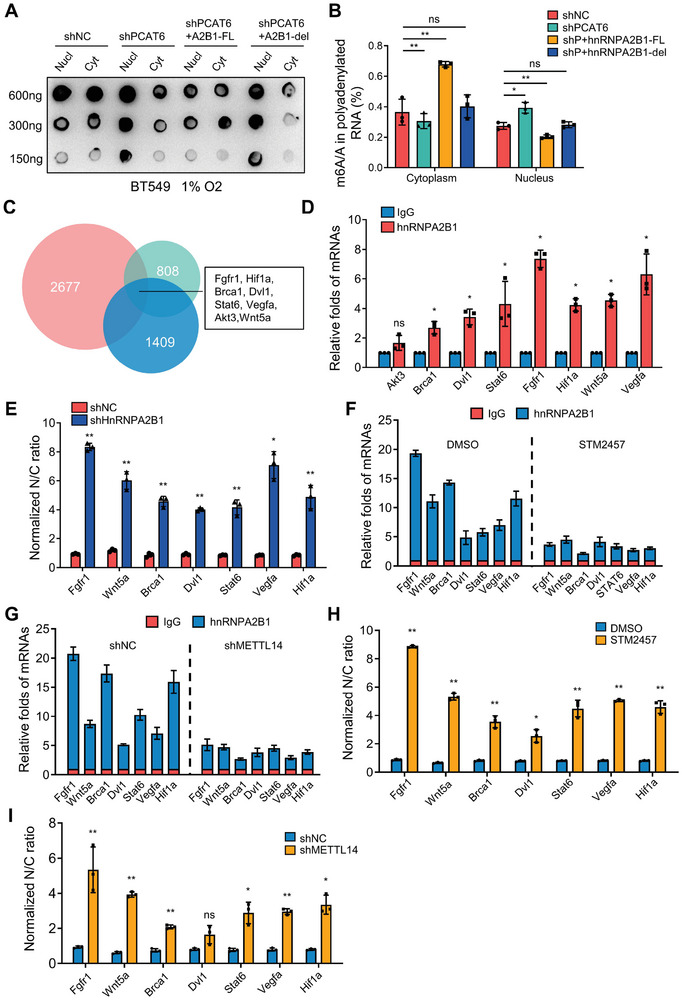
HnRNPA2B1 mediates CSC‐related mRNA nuclear export in an m6A‐dependent manner. A) Dot blotting showing m6A levels of nuclear and cytoplasmic mRNAs in PCAT6 knockdown and control BC cells with or without hnRNPA2B1 full length (A2B1‐FL) or hnRNPA2B1 deletion (A2B1‐del) using an anti‐m6A antibody. The total RNAs (600, 300, 150 ng) were spotted onto a Hybond‐N^+^ membrane. B) The m6A levels of nuclear and cytoplasmic mRNAs were detected via LS‐MS/MS in PCAT6 knockdown and control BC cells with or without hnRNPA2B1 full length or hnRNPA2B1 deletion. C) Venn diagram of DEGs (2677) in PCAT6 knockdown BC cells, hnRNPA2B1 immunoprecipitated mRNAs (1409), and DEGs (808) in hnRNPA2B1 knockdown cells. D) CSC‐related mRNAs were enriched with hnRNPA2B1. RIP assays were performed with spheres from hypoxic Hs578T cells using anti‐hnRNPA2B1; IgG served as the negative control. The indicated RNAs enriched in the RIP precipitates were determined through qRT‐PCR. E) The relative nuclear (labeled as N) and cytoplasmic (labeled as C) mRNA ratio were detected via qRT‐PCR in shNC/Hs578T and shHnRNPA2B1/Hs578T cells. F,G) CSC‐related mRNAs were enriched by hnRNPA2B1 under treatment with STM2457 (0.2 µm) (F) or knockdown of METTL14 (G). RIP assays and m6A mRNA detection were conducted as described in (D). The data are represented as mean ± SD. H,I) qRT‐PCR was performed to determine the relative nuclear (labeled as N) and cytoplasmic (labeled as C) mRNA ratio in hypoxic Hs578T cells after treatment with STM2457 (0.2 µm) or DMSO (H) or in METTL14‐knockdown Hs578T and control cells (I) under hypoxia. (ns, no significant; ^*^ *p* < 0.05, ^**^ *p* < 0.01).

As a m6A reader, hnRNPA2B1 is implicated in mRNA methylation as well as nucleocytoplasmic transport.^[^ ^33^ ^]^ Therefore, we assessed whether PCAT6‐induced m6A‐tagged mRNA nuclear export was mediated by hnRNPA2B1. We transfected hnRNPA2B1 full‐length (hnRNPA2B1‐FL) or a deletion mutant lacking the hnRNPA2B1 RRM1 domain (hnRNPA2B1‐del), which displays a binding preference for m6A‐containing RNAs,^[^ ^28^ ^]^ into PCAT6 knockdown BC cells. PCAT6 knockdown‐induced nuclear accumulation of m6A‐tagged mRNA was rescued by ectopic expression of hnRNPA2B1 full‐length but not by hnRNPA2B1‐del, as measured via dot blotting and LC‐MS/MS assays (**Figure** **5**a,b; Figure S5a,b, Supporting Information). As demonstrated by immunoblotting with anti‐m6A antibodies to HA‐hnRNPA2B1 IP lysates, PCAT6 knockdown decreased the m6A‐modified RNA bounded by hnRNPA2B1 in hypoxic BC cells (Figure S5c, Supporting Information), suggesting PCAT6 facilitates the export of m6A‐modified RNA in hypoxic BC cells via hnRNPA2B1.

To screen which m6A‐tagged mRNAs were exported by the PCAT6‐hnRNPA2B1 axis, we systematically analyzed mRNA and m6A‐tagged mRNA data from three datasets, including a known hnRNPA2B1‐recognized mRNAs from the hnRNPA2B1‐RIP‐seq database (http://starbase.sysu.edu.cn/), m6A‐tagged mRNAs from our PCAT6‐related meRIP‐seq data (shPCAT6 vs shNC), and mRNAs from the reported hnRNPA2B1 knockdown RNA‐seq dataset. A total of 59 hnRNPA2B1‐bound m6A mRNA were potentially exported via the PCAT6‐hnRNPA2B1 axis (data not shown). Among these mRNA, stemness maintenance‐related mRNA (e.g., Fgfr1, Brca1, Dvl1, Stat6, Hif1a, Wnt5a, Vegfa, and Akt3) were identified (Figure 5c), and seven of them (Fgfr1, Brca1, Dvl1, Stat6, Hif1a, Wnt5a, and Vegfa) were further validated in the hnRNPA2B1‐RIP precipitates (Figure 5d). Analysis of their nuclear and cytoplasmic distribution in hnRNPA2B1‐deficient and control BC cells under hypoxia revealed that all CSC‐related mRNA were located in the nuclei of hypoxic hnRNPA2B1‐deficient cells (Figure 5e). Overexpression of hnRNPA2B1 in normoxic BC cells promoted the nuclear export of CSC‐related mRNA (Figure S5d, Supporting Information). To understand whether hnRNPA2B1‐mediated mRNA export was related to m6A modification, METTL14 ‐knockdown cells and STM2457 (an m6A modification inhibitor) were used. Knockdown of METTL14 or STM2457 treatment led to decreased binding affinity between hnRNPA2B1 and m6A‐modified mRNA (Figure 5f–g), thus causing significant nuclear accumulation of mRNAs (Figure 5h,i), as observed in hnRNPA2B1 knockdown cells. However, the transfection of hnRNPA2B1‐FL, rather than hnRNPA2B1‐del, into PCAT6 knockdown cells decreased the nuclear accumulation of mRNA (Figure S5e, Supporting Information), consistent with the results observed in PCAT6 overexpressing cells under normoxia (Figure S5f, Supporting Information). Hence, hnRNPA2B1 mediates nuclear mRNA export in an m6A‐dependent manner.

**Figure 6:**
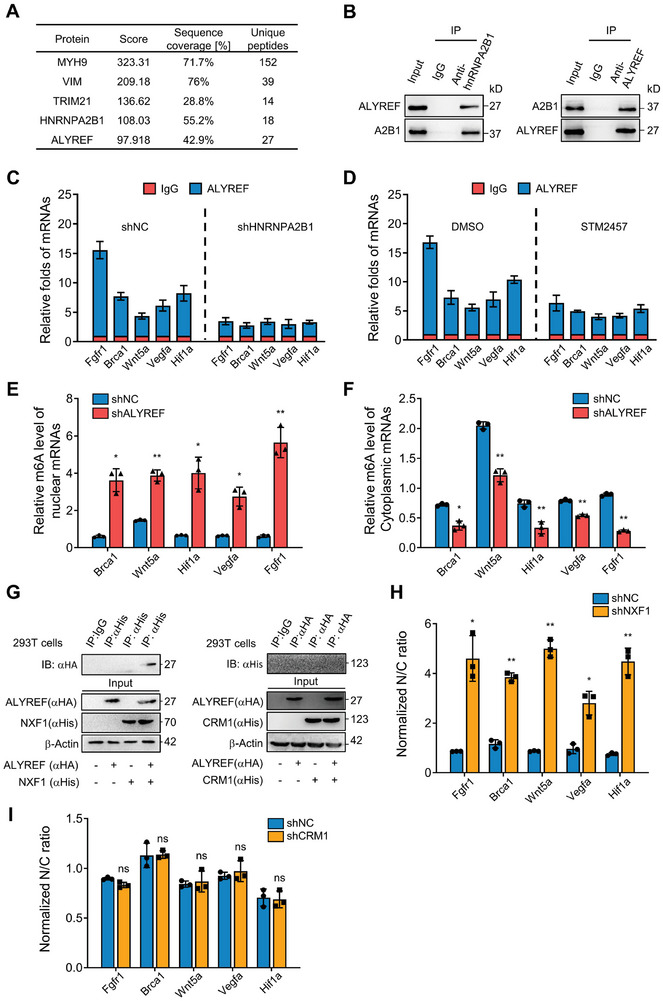
PCAT6/hnRNPA2B1 bridges m6A mRNA selective export via ALYREF. A) ALYREF was identified using mass spectrometry in hnRNPA2B1 immunoprecipitated proteins of Hs578T and MDA‐MB‐231 cells. B) Co‐IP showing the mutual binding between hnRNPA2B1 and ALYREF in hypoxic tumor cells. C,D) RIP was conducted using anti‐ALYREF or IgG antibodies with spheres derived from hnRNPA2B1 knockdown and control Hs578T cells (C) or Hs578T cells treated with STM2457 (0.2 µm) or DMSO (D). The indicated RNA enriched in the RIP precipitates was analyzed via qRT‐PCR. The data are represented as mean ± SD. E,F) The m6A levels of nuclear mRNA (E) and cytoplasmic mRNA (F) were determined by performing meRIP‐PCR in ALYREF knockdown and control Hs578T cells. G) Interaction between ALYREF and NXF1 (left) or between ALYREF and CRM1 (right) were detected through co‐IP. HEK293T cells were transfected with HA‐ALYREF and/or His‐NXF1 plasmids as well as HA‐ALYREF and/or His‐CRM1 for 24 h. Cell lysates were immunoprecipitated with anti‐HA or anti‐His antibodies, followed by western blotting to assess the indicated protein binding. H,I) The relative nuclear (labeled as N) and cytoplasmic (labeled as C) mRNA ratio were detected via qRT‐PCR in (H) NXF1‐knockdown Hs578T cells, I) CRM1‐knockdown Hs578T, and control cells. (ns, no significant; ^*^ *p* < 0.05, ^**^ *p* < 0.01).

RNA transport through nuclear pore complexes requires the export of adaptors and receptor proteins. Therefore, we sought to determine the export receptors and adaptors potentially involved in hnRNPA2B1‐mediated mRNA nuclear export using IP‐MS. ALYREF, a mRNA adaptor and core subunit of transcription‐export complex (TREX),^[^ ^34^ ^]^ exhibited a preferential affinity for hnRNPA2B1 (**Figure** **6**a), which was subsequently confirmed via immunoprecipitation (Figure 6b). Knockdown of hnRNPA2B1 or inhibition of m6A‐tagged mRNA methylation by STM2457 significantly decreased mRNA binding to ALYREF (Figure 6c,d). Loss of ALYREF led to an obvious accumulation of hnRNPA2B1‐recognized m6A mRNA in the nucleus (Figure 6e,f). These findings underscore the pivotal role of ALYREF as a critical adaptor in hnRNPA2B1‐mediated export of m6A mRNAs. Two conserved transport complexes govern nuclear RNA exports: NXF1 with its cofactor p15 (also known as NXT1) and chromosome region maintenance 1 protein homologue (CRM1).^[^ ^18^ ^]^ Thus, we determined which complex was necessary for CSC‐related m6A‐modified mRNA export. Co‐immunoprecipitation assay revealed a specific interaction between ALYREF and NXF1 rather than between ALYREF and CRM1 in 293T cells exogenously transfected with ALYREF, NXF1, or CRM1 (Figure 6g). Furthermore, the knockdown of NXF1, but not CRM1, significantly affected the distribution of CSC‐associated m6A mRNA in hypoxic BC cells (Figure 6h,i). Summarily, hnRNPA2B1 mediates the selective export of CSC‐associated m6A mRNA through ALYREF/NXF1, in contrast to the ALYREF/CRM1 complex.

### 2.6 m6A‐Tagged Fgfr1 is Crucial for BCSCs Stemness Maintenance

**Figure 7:**
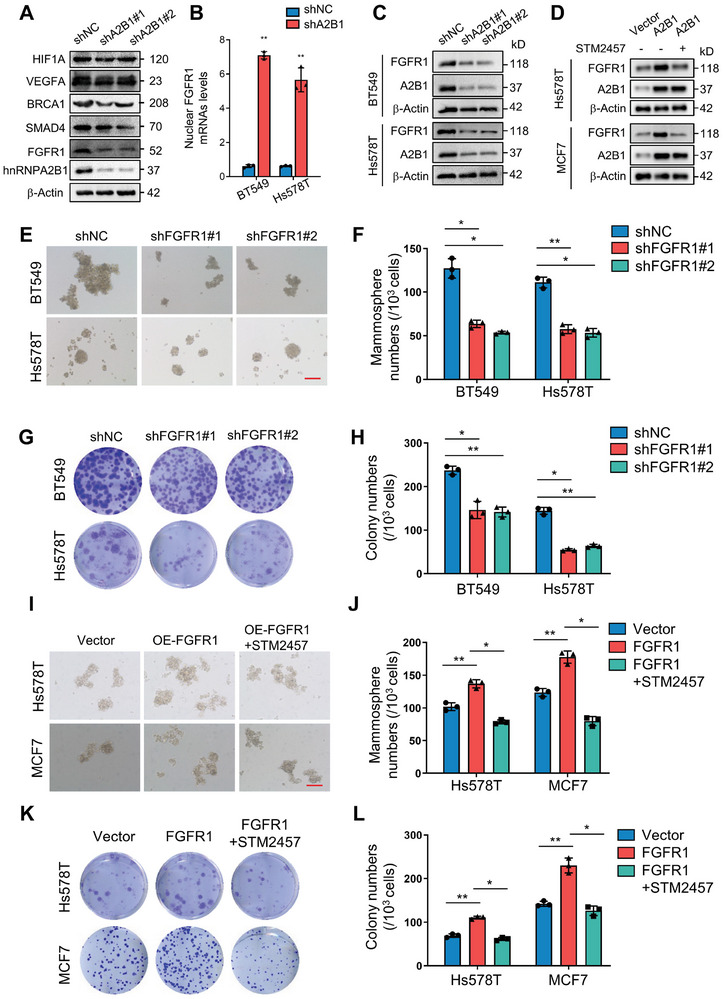
FGFR1 is essential for BCSCs stemness maintenance and doxorubicin resistance. A) Western blot analysis of stemness maintenance‐related proteins in cell lysates from hnRNPA2B1 knockdown and control BT549 cells. B) qRT‐PCR were used to detect Fgfr1 mRNA levels. C,D) Western blot analysis of FGFR1 and hnRNPA2B1 in cell lysates from hnRNPA2B1 knockdown (C) or hnRNPA2B1 (labeled as A2B1) overexpressing (D) and their control BC cells with or without STM2457 treatment. E,F) Mammospheres formation abilities were assessed in FGFR1 knockdown (shFGFR1#1, shFGFR1#2) and control BC cells. Representative images of spheres and mammosphere numbers are depicted in (E,F), respectively. Original magnification, ×100. Scale bars, 100 µm. G,H) Colony formation abilities were assessed in FGFR1 knockdown (shFGFR1#1, shFGFR1#2) and control BC cells. Representative images of spheres and colony numbers are shown in (G,H), respectively. I,J) The mammosphere formation abilities were assessed in FGFR1‐overexpressing (FGFR1) BC cells with or without STM2457 treatment, along with control BC cells. Representative images of spheres and mammosphere numbers are shown in (I,J), respectively. Original magnification, ×100. Scale bars, 100 µm. K,L) Colony formation abilities were assessed in FGFR1 overexpressing (FGFR1) with or without STM2457 treatment and control BC cells. Representative images of colonies and colony numbers are shown in (K,L), respectively. (^*^ *p* < 0.05, ^**^ *p* < 0.01).

To investigate the impact of hnRNPA2B1‐mediated m6A‐modified mRNA export on BCSCs stemness maintenance, Fgfr1 was selected for our subsequent research. Its mRNA and protein exhibited the most notable changes among hnRNPA2B1 target genes associated with the stem cell pluripotency maintenance (**Figure** **7**a). Fgfr1 mRNA accumulated in the nucleus of hnRNPA2B1 knockdown cells (Figure 7b), and fibroblast growth factor receptor 1 (FGFR1) protein levels were significantly decreased in whole cells (Figure 7c). Furthermore, ectopic hnRNPA2B1 overexpression induced an increase in FGFR1 protein levels, which were markedly attenuated by STM2457 treatment (Figure 7d). Because FGFR1 can function as an oncogenic protein in multiple biological processes, including BCSCs characteristics, we evaluated the role of increased FGFR1 expression in BCSCs enrichment and stemness maintenance. Knockdown of Fgfr1 notably decreased mammosphere formation, colony formation ability, and the percentage of BCSCs in hypoxic BC cells (Figure 7e–h). Conversely, FGFR1 increase significantly promoted mammosphere formation, colony formation ability, and the percentage of BCSCs in BC cells, whereas treatment with STM2457 abolished these effects (Figure 7i–l). Collectively, m6A‐tagged FGFR1 plays a crucial role in fueling BCSCs stemness characteristics.

### 2.7 Targeting hnRNPA2B1‐Mediated m6A RNA Export Increases BCSCs Sensitivity to Chemotherapeutic Drug

**Figure 8:**
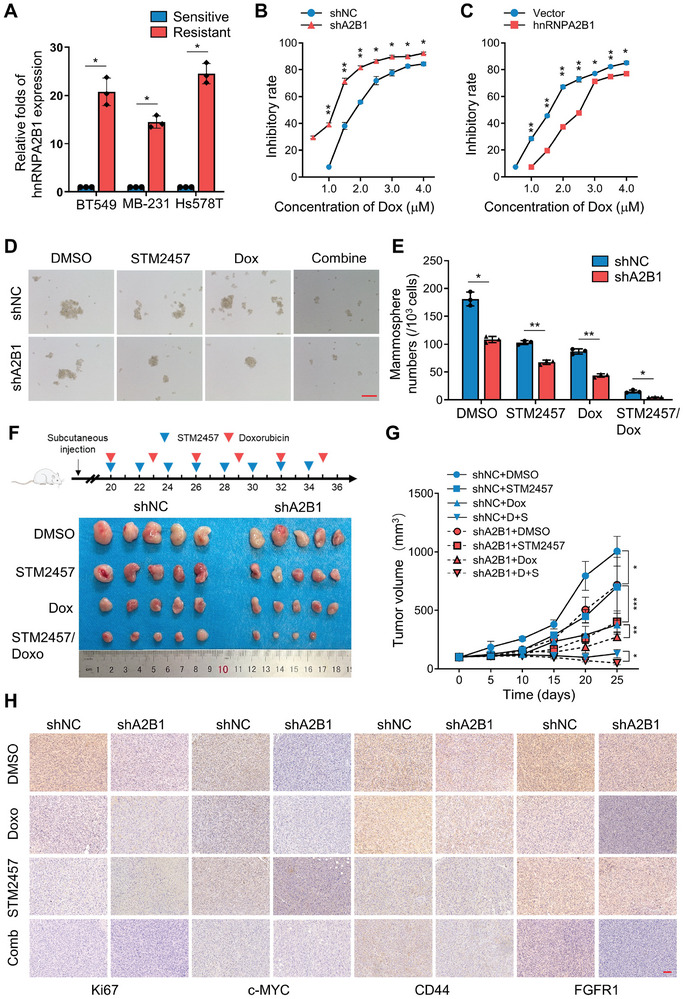
Inhibition of hnRNPA2B1‐mediated m6A RNA export increases the sensitivity of BCSCs to chemotherapeutic drug. A) qRT‐PCR was conducted to measure the levels of hnRNPA2B1 mRNAs in doxorubicin resistant Hs578T, BT549 and MDA‐MB‐231 cells. B,C) Dose‐dependent doxorubicin sensitivities were examined with proliferation assay for B) hnRNPA2B1 knocked down BT549 cells or C) hnRNPA2B1 overexpressing MDA‐MB‐231 cells and their controls. The inhibitory rates are plotted as the fraction of dying cells relative to the number of untreated cells. The data are represented as mean ± SD. D,E) Mammosphere formation abilities were assessed in hnRNPA2B1 knockdown BC cells and control BC cells. D) Representative images of spheres and E) mammosphere numbers, respectively. Original magnification, ×100. Scale bars, 100 µm. F,G) Loss of hnRNPA2B1 attenuated xenograft tumor growth in vivo; 1×10^6^ of hnRNPA2B1 knocked down (labeled as shA2B1) or control BT549 cells were injected subcutaneously into female BALB/c nude mice individually. After 20 days of injection, DMSO, doxorubicin (2 mg kg^−1^), STM2457 (50 mg kg^−1^) or doxorubicin combined with STM2457 was administered by intraperitoneal injection. F) Tumor sizes and G) volumes. The data are represented as mean ± SD. H) Images of IHC analysis of Ki‐67, c‐MYC, CD44, and FGFR1 from xenograft tumors. (^*^ *p* < 0.05, ^**^ *p* < 0.01).

Given the significant role of hnRNPA2B1 in exporting CSC‐related mRNAs in breast tumor cells, we hypothesized that targeting hnRNPA2B1 could abrogate chemoresistance in breast tumor. Initially, we observed an increase in hnRNPA2B1 expression in doxorubicin‐resistant BC cells (**Figure** **8**a). HnRNPA2B1 knockdown significantly increased the efficiency of doxorubicin in BCSCs (Figure 8b). However, the overexpression of ectopic hnRNPA2B1 decreased the efficiency of chemotherapeutic drugs in non‐BCSCs (Figure 8c). Notably, similar inhibition of mammosphere formation was achieved with m6A inhibition via STM2457 treatment (Figure 8d,e). A xenograft nude mouse experiment was performed by subcutaneously injecting hnRNPA2B1/WT or hnRNPA2B1/KD BT549 cells into nude mice and administering doxorubicin, STM2457, or doxorubicin combined with STM2457. As anticipated, the depletion of hnRNPA2B1 led to a substantial reduction in both tumor volume and weight compared to the control group, rendering the cells hypersensitive to doxorubicin (Figure 8f,g). The inhibition of m6A combined with doxorubicin significantly augmented the effectiveness of chemotherapy (Figure 8f,g). Accordingly, hnRNPA2B1‐depleted tumors treated with the m6A inhibitor STM2457 or STM2457 combined with doxorubicin showed substantially reduced CD44, c‐MYC, Ki67, and FGFR1 proteins, which are markers of cell proliferation and stemness characteristics in tumors (Figure 8h). Inhibition of hnRNPA2B1‐mediated m6A recognition can suppress breast tumor growth in vivo and increase the chemotherapeutic effectiveness of BC.

## 3 Discussion

The high mortality rate of BC patients remains a challenge in clinical practice. In‐depth studies and the elucidation of the molecular mechanisms underlying the malignant progression and chemoresistance of BC are fundamental steps for the development of effective therapeutic strategies and targeted drugs. In this study, we demonstrated that hypoxia‐induced lncRNA PCAT6 functions as a scaffold between ISG15 and hnRNPA2B1, leading to enhanced ISGylation of hnRNPA2B1, which protects hnRNPA2B1 from proteasomal degradation (Figure 4; Figure S4, Supporting Information). Enhanced hnRNPA2B1 recognizes CSC‐related mRNAs (e.g., FGFR1) in an m6A‐dependent manner and plays a crucial role in their nuclear export through selective binding to the ALYREF/NXF1 complex. The effective export of CSC‐related m6A mRNAs increases gene expression levels for the maintenance and self‐renewal of BCSCs, potentially leading to malignant breast tumor progression and resistance to chemotherapy drugs. Treating BC cells with an m6A inhibitor and shRNA targeting PCAT6 or hnRNPA2B1 effectively ameliorated the malignant phenotypes of BC.

Hypoxia‐induced lncRNA PCAT6 acts as a crucial regulator of BC development. Tumor malignancy and progression are closely influenced by various factors (such as the cellular microenvironment and nutrient, oxidative, and metabolic stress responses). Notably, hypoxic microenvironment can regulate hypoxia‐responsive genes via HIF‐1α, a key factor in tumor progression. Hypoxia‐responsive lncRNAs may play a crucial role in the malignant progression of solid tumors. Hypoxic lncRNA‐PMAN strongly suppresses ferroptosis by promoting the cytoplasmic translocation of ELAVL1 in peritoneal dissemination from gastric cancer.^[^ ^35^ ^]^ HIF‐1α‐induced lncRNA DACT3‐AS1 facilitates HCC metastasis via FOXA3 deacetylation.^[^ ^36^ ^]^ Consistent with this fact, we found that hypoxia‐regulated PCAT6 is crucial for BCSCs stemness maintenance and chemoresistance in vitro and in vivo. Notably, BC patients with high PCAT6 levels have poor overall survival and respond poorly to chemotherapy. Data from both the TCGA database and our cohort of 160 BC patients suggest that PCAT6 holds promise as a novel diagnostic and therapeutic target in BC. Hence, hypoxic PCAT6 plays a pivotal role in regulating the nuclear export of m6A‐modified RNAs to promote BCSCs stemness maintenance and doxorubicin resistance.

ISG15‐induced ISGylation of hnRNPA2B1 is essential for hnRNPA2B1 stability. HnRNPA2B1 is a pivotal factor in the modulation of diverse forms of cancer progression. Its stability and functionality are subject to several post‐translational modifications, encompassing ubiquitination,^[^ ^37^ ^]^ neddylation,^[^ ^38^ ^]^ and SUMOylation.^[^ ^39^ ^]^ Ubiquitination typically results in protein degradation through the proteasomal pathway. However, other post‐translational modifications such as SUMOylation and ISGylation exert distinct effects on protein processing, which represent significant factors influencing the behavior, processes, and functions of organisms. For instance, SUMOylation of DDX19 not only enhances but also stabilizes Gle1, thereby tightly regulating mRNA export.^[^ ^40^ ^]^ ISGylation of BECN1 competes with its ubiquitination of BECN1, effectively activating BECN1 to inhibit PIK3C3 activity, which plays a crucial role in autophagy activation during antiviral responses.^[^ ^23^ ^]^ Our study revealed an intriguing mechanism by which PCAT6 forms a complex with ISG15 and hnRNPA2B1, thereby facilitating hnRNPA2B1 stability via ISGylation. Interestingly, ISGylated hnRNPA2B1 serves as a protective shield against ubiquitination‐mediated degradation, thus introducing a novel paradigm for post‐translational modifications of hnRNPA2B1. Additionally, the positive correlation between ISG15 and hnRNPA2B1, coupled with their crucial roles in hypoxia‐induced malignant progression in BC, was validated in both BC cells and tissues, highlighting the potential of targeting hnRNPA2B1 ISGylation as a viable therapeutic approach for BC.

Interestingly, our study also revealed that hnRNPA2B1 selectively mediates m6A‐modified mRNA export via the ALYREF/NXF1 nuclear pore complex in BCSCs. Mammalian mRNA export, a highly selective process regulated by multiple mechanisms, must overcome the permeability barrier generated by nuclear pore proteins. Adaptors play a pivotal role in nuclear transport by identifying mRNAs and transferring them to transport factors tasked with mRNA export. The nuclear export of mRNA plays a regulatory role in many crucial biological processes such as gene expression, maintenance of pluripotency, hematopoiesis, proliferation, and cell survival. For instance, the THO complex, a member of the TREX family, preferentially interacts with pluripotency gene transcripts such as NANOG and SOX2 through Thoc5 and is required for self‐renewal by regulating their export and expression.^[^ ^41^ ^]^ Selective nuclear export of mRNAs is precisely regulated and essential for the maintenance of cellular homeostasis. In particular, the selective export of m6A‐modified mRNA is governed by different mechanisms.^[^ ^25^ ^]^ For instance, YTHDC1 binding to target transcripts is sufficient to facilitate m6A‐tagged mRNA selective nuclear export via SRSF3 and NXF1.^[^ ^42^ ^]^ During neural differentiation, FMRP selectively bind to m6A‐modified RNAs, facilitating their nuclear export via the CRM1.^[^ ^23^ ^]^ Similarly, our study revealed that hnRNPA2B1, an m6A reader, acts as a bridge for m6A selectivity in mRNA export by interacting with ALYREF, which leads to increased translation of CSC‐related m6A mRNAs and boosts the cytoplasmic abundance of these targets, including Fgfr1. Targeting hnRNPA2B1 using lentivirus‐mediated shRNA or blunting m6A mRNA export using the specific inhibitor STM2457 resulted in a remarkable reduction in BCSCs stemness and tumor initiation both in vitro and in vivo, thereby enhancing the therapeutic effect of doxorubicin. Unfortunately, the lack of small‐molecule inhibitors of hnRNPA2B1 and ALYREF has hindered further exploration of their clinical application, which could potentially become a promising research direction.

Conclusively, the hypoxic lncRNA PCAT6 plays a crucial role in BC cell stemness maintenance and tumor progression. PCAT6 is a lncRNA that directly regulates protein function via ISGylation. Our study also highlighted a new regulatory mechanism of m6A mRNA export, which closely impacts BC doxorubicin resistance in vitro and in vivo, and provided a rationale for targeting the PCAT6‐ISG15‐hnRNPA2B1‐FGFR1 signaling axis as a potential therapeutic strategy in BC.

## 4 Experimental Section

### Cell Culture

BT‐549, Hs578T, MDA‐MB‐231, and MCF‐7 were cultured in the recommended medium (Gibco, USA), containing 10% FBS (HyClone, Australia) and 1% streptomycin/penicillin (Beyotime, Shanghai, China). Incubation of the cells was performed in a humidified incubator at 37 °C. To induce hypoxia, the cells were cultured under conditions of 5% CO_2_, 1% O_2_, and 94% N_2_.

### Tissues Specimens

Human breast tumor and their adjacent normal breast tissues were acquired from the First Affiliated Hospital of Chongqing Medical University, and the clinical patient sample collection was approved by the Research Ethics Committee of Chongqing Medical University. All patients have signed informed consent forms.

### Plasmids and Short Interfering RNAs

Lipofectamine 3000 (Invitrogen) were used for small interfering RNA (siRNAs) against HIF‐1a (GenePharama, Shanghai, China) transfection. To achieve stable knockdown HIF‐1a, PCAT6, ISG15, hnRNPA2B1, METTL14, ALYREF, NXF1, CRM1, FGFR1, lentivirus‐mediated shRNA constructs targeting these genes were obtained from GenePharama (Shanghai, China) were constructed. Non‐targeting shRNA (shNC) were used for negative control. PCAT6, ISG15, hnRNPA2B1, METTL14, ALYREF, NXF1, CRM1, FGFR1 were overexpressed by lentivirus containing complete coding sequences constructed by Genechem (Shanghai, China). Empty lentivirus vectors were used as negative control. Stable cell lines were selected by puromycin. The pGL3‐PCAT6 WT reporter or pGL3‐PCAT6 MUT reporter were obtained by cloning PCAT6 promoter with HIF‐binding sites (WT: –GCGCG–) or its corresponding mutant sites (MUT: –GGCAG–) into a pGL3 luciferase reporter vector. The human hnRNPA2B1 full‐length and deletion of RRM1 domain (hnRNPA2B1‐del) were amplified by PCR and subcloned into vectors pEF‐5HA. The Table S1 (Supporting Information) provides the list of siRNA and shRNA sequences utilized in this research.

### Antibodies and Reagents

Antibodies against HIF‐1a,Ubiquitin, CRM1, CD44, VEGFA were obtained from CST. Antibodies against ISG15, hnRNPA2B1, c‐MYC, SMAD4 were from Proteintech (Rosemont, IL, USA). Antibodies against HA, MYC, His, FLAG were from Abmart (Shanghai, China). Antibodies against EBG were purchased from Sigma‐Aldrich (USA). Antibody against m6A, METTL3, METTL14, BRCA1 were from Abcam (Cambridge, UK). Antibodies against b‐Actin were obtained from ZSGB‐BIO (Beijing, China). The Ni^2+^‐NTA agarose beads were acquired from Qiagen.

### Protein Extraction and Western Blotting

For total protein extraction, cells were lysed by RIPA buffer (Beyotime, China) containing 1% PMSF (Beyotime, China) and quantified with BCA protein assay kit (Beyotime, China). The study utilized sodium dodecyl sulfate polyacrylamide gel electrophoresis (SDS‐PAGE) with an 8–12% gel to separate denatured protein samples (20–50 mg). The separated proteins were then transferred onto a polyvinylidene fluoride (PVDF) membrane. Following blocking with 5% non‐fat milk, the membranes were incubated overnight with the primary antibody at 4 °C. Subsequently, the membranes were hybridized with secondary antibodies (Biosharp, Beijing, China) specific to the species at room temperature for 1–2 h. To detect the proteins, the ECL detection reagents (Bio‐rad, America) were employed with enhanced chemiluminescence system (Amersham Pharmacia Biotech) and normalized the results to β‐Actin. Each experiment was repeated at least three times.

### CHX Chase Assay

For CHX chase assay, cells were treated with 10 µm cycloheximide for 0, 6, 12, and 18 h. Then cells were lysed by RIPA buffer (Beyotime, China) containing 1% PMSF (Beyotime, China) for immunoblotting with indicated antibodies.

### RNA Isolation and qRT‐PCR

Total RNAs from both tissues and cells were extracted by TRIZOL reagent (Invitrogen) according to the manufacturer's instruction. The nuclear and cytosolic RNA fractions were performed using the PARISTM Kit (Invitrogen, USA) according to the manufacturer's instructions. After reverse transcription of RNA to cDNA with PrimeScript RT Reagent Kit (Takara, Dalian, China), the cDNA was subjected to RT‐qPCR with SYBR Premix Ex Taq II Kit (Takara, Dalian, China) on a Bio‐RadCFX96 system (Bio‐Rad, CA, USA). The total RNA expression was normalized by β‐actin, while the nuclear RNA and cytosolic RNA was normalized by U6 or GAPDH, respectively. The primers used in the study are listed in Table S2 (Supporting Information).

### Chromatin Immunoprecipitation (ChIP)

Chromatin immunoprecipitation (ChIP) assays were conducted using the ChIP Assay Kit (Cell Signaling Technology, MA, USA) according to the manufacturer's instructions. Briefly speaking, cells were fixed with 37% formaldehyde to crosslink DNA to proteins. Then, DNA were sonicated into 200–800 bp fragments. Anti‐HIF‐1a were added for co‐precipitation. The complex was captured by Protein G magnetic beads. DNA were purified and detected by PCR.

### m6A Dot‐Blotting Assay

The m6A dot blot assay was conducted as follows. Briefly, total RNAs were extracted. Following the assessment of mRNA concentration and purity, denaturation of the mRNAs was performed at temperature of 95 °C for 2 min, subsequently followed by rapid cooling on ice. The RNAs (600 ng respectively) were double diluted and spotted onto a Hybond‐N^+^ membrane (GE Healthcare). Membranes were exposed to UV for RNA cross‐linking. Subsequently, the membranes were washed in PBST buffer for 5 min, blocked with 5% non‐fat milk in PBST at room temperature for 2 h and incubated with anti‐m6A antibody (1:1000, Abcam, USA) for about 12–16 h at 4 °C. The membranes were subsequently hybridized with the secondary antibodies for 1 h, then visualized with enhanced chemiluminescence system (Amersham Pharmacia Biotech).

### RNA In Situ Hybridization (ISH)

Utilizing a digoxin‐labeled probe specifically designed for PCAT6 (GenePharama, Shanghai, China), ISH was performed on TMA (Outdo Biotech, Shanghai, China) comprising 160 BC tissues and 10 normal tissues. The study initiated the dewaxing and rehydration of the TMA. Then, the tissues were subjected to proteinase K digestion at 37 °C for 10 min, followed by an overnight hybridization process with the PCAT6 probe at a temperature of 45 °C. Post‐hybridization, the tissues underwent an overnight incubation at 4 °C with biotin‐conjugated antibodies targeting digoxin, followed by DAB staining. Quantification of PCAT6 expression was executed by product of two criteria: the staining intensity (graded as strong = 3, moderate = 2, weak = 1 and negative = 0) and the extent of immunostaining (> 76% = 4, 51–75% = 3, 26–50% = 2, 5–25% = 1, < 5% = 0). Categorization into low or high expression groups was determined based on the mean ISH scores.

### Immunohistochemical Staining (IHC)

IHC staining was conducted on sections from tumor tissues of nude mice xenografts and TMA that contain 38 BC tissues. Briefly, tissue sections were deparaffinized, subjected to antigen retrieval. After endogenous peroxidase inactivation and blocked at room temperature. The primary antibodies against CD44 (1:200, CST), Ki67 (1:500, CST), c‐MYC (1:200, CST), ISG15 (1:200, Proteintech), hnRNPA2B1 (1:200, Proteintech), FGFR1 (1:200, Proteintech) were used to incubate with tissues at 4 °C overnight. Tissues were incubated with an enhanced peroxidase conjugated secondary antibody (ZSGB‐BIO, Beijing, China), followed by visualization with DAB. Images were captured with a light microscope (Olympus). IHC slides were independently scored by two independent observers. The immunostaining intensity was calculated by methods described in ISH score.

### Co‐Immunoprecipitation (Co‐IP)

Cell lysates were executed using lysis buffer S (containing 20 mm sodium phosphate at pH 7.4, 150 mm NaCl, 1% SDS, 1% Triton, 0.5% sodium deoxycholate, 5 mm EDTA, 5 mm EGTA, 20 mm MG132, 5 mm DTT, and a protease inhibitor cocktail). Following a 10‐min boiling step, the lysate was sonicated until achieving a fluid consistency. Subsequent to centrifugation, 10% of the lysates were employed as Input. The remaining lysates were incubated overnight at 4 °C with the antibodies and Protein A/G beads. The beads underwent five rounds of washing, then boiled for 10 min in SDS loading buffer, after which Western blotting analysis was performed.

### ISGylation Assay

ISGylation of hnRNPA2B1 was confirmed by two different methods. The ISGylation analysis by Ni^2+^‐NTA pulldown was performed as previously described with minor changes.^[^ ^43^ ^]^ Briefly speaking, His‐tagged bait proteins were purified, and cell lysates were mixed with purified protein and subjected to binding reactions. The mixtures were captured onto the Ni^2+^‐NTA resin and eluted with elution buffer. Western blotting assays were performed to identify the target proteins. An analysis of ISGylation, including both exogenous and endogenous ISGylation of hnRNPA2B1, was conducted using Co‐immunoprecipitation (Co‐IP) following the previously established protocol.^[^ ^43^ ^]^

### Mammosphere Formation and Self‐Renewal Capability Assay

Breast cancer cells were grown in serum‐free DMEM/F12 medium (Gibco, USA) at a concentration of 1×10^4^ cells mL^−1^. The medium was supplemented with 2% B27 (Gibco, USA), along with 20 ng mL^−1^ epidermal growth factor (EGF, PeproTech, USA), 20 ng mL^−1^ basic fibroblast growth factor (bFGF, PeproTech, USA), 0.4% albumin from bovine serum (BSA, Sigma‐Aldrich, USA), 2 µg mL^−1^ heparin (Sigma–Aldrich, USA), and insulin‐transferrin‐selenium (Invitrogen, USA). Mammospheres were cultured on 6‐well plates coated with 2% poly‐2‐hydroxyethyl methacrylate (poly‐HEMA, Sigma‐Aldrich, USA). Mammospheres were subjected incubation under normoxic or hypoxic conditions and subsequently transferred every 7 days. The self‐renewal capability was accomplished by evaluating the abundance of mammospheres (with a diameter surpassing 50 µm) as previously outlined.^[^ ^9^ ^]^ The representative images were captured using the microscope (OLYMPUS, Tokyo, Japan).

### Colony Formation Assay

For colony formation assay, breast cancer cells (1 × 10^3^ cells/well) were seeded into 6‐well plates and cultured in proper medium under normoxia or hypoxia conditions. After two weeks, cells were washed softly and fixed with methanol for 15 min and stained with 0.5% crystal violet for 5 min. The colonies images were captured by scanner and counted by image J software.

### Flow Cytometry Analysis

Following a 30‐min incubation at 4 °C with APC‐anti‐CD44 (BD Biosciences) at a dilution of 1:167, PE‐anti‐CD24 (BD Biosciences) at a dilution of 1:50, 1 × 10^6^ BC cells were washed four times with cold PBS. Subsequently, the cells were suspended in 100 µL of PBS. The CD44^+^/CD24^−/low^ cells were analyzed by flow cytometer (Beckman Coulter, High Wycombe, UK).

### RNA Pull‑Down and Mass Spectrometry

The biotin‐labeled RNA was transcribed in vitro using the MAXIscript Kit (Thermo Fisher Scientific, Inc). In accordance with the manufacturer's protocols, the Pierce Magnetic RNA‐Protein Pull‐Down Kit (Thermo Fisher Scientific) was utilized for conducting the RNA pull‐down assay. Biotin‐labeled RNA was mixed with cell lysates at 4 °C after incubation with DNase I. Then, magnetic beads were added for protein binding. The protein complexes were analyzed by mass spectrometry (5600‐plus, AB SCIEX, USA) and confirmed by western blot. The primer sequences are listed in Table S1 (Supporting Information), and the proteins pulled down by PCAT6 are listed in Table S5 (Supporting Information).

### RNA Sequencing and m6A‐Sequencing

Nuclear and cytoplasmic RNA extraction was same as above described. RNA‐seq and data analysis were performed by LC‐Bio Technology Co., Ltd. The nuclear RNA and cytoplasmic RNA from shNC and shPCAT6 BC cells were subjected to RNA‐seq, and three independent biological replicates of each group were performed. To conduct m6A sequencing, total RNA was extracted and purified from Hs578T cells, following which it was subjected to m6A RNA immunoprecipitation based on the manufacturer's instructions. Eluted m6A‐containing fragments (referred to as IPs), alongside untreated input control fragments, were subsequently converted into the final cDNA library using strand‐specific library preparation with the dUTP method. The paired‐end libraries, with an average insert size of approximately 100 ± 50 bp, were then subjected to paired‐end sequencing (PE150) using an Illumina Novaseq 6000 platform.

### m6A RNA Detection by Co‐Immunoprecipitation (Co‐IP)

HnRNPA2B1 bound m6A RNA was tested by Co‐immunoprecipitation as previous reported.^[^ ^44^ ^]^ Cell lysis was suspended with lysis buffer (50 mm Tris‐HCl pH 7.4, 150 mm NaCl, 1% NP‐40, 100 U mL^−1^ RNase inhibitor and Protease inhibitor cocktail) after UV‐crosslink. Then immunoprecipitated with anti‐HA antibody. The immunoprecipitation complex was washed twice with high‐salt buffer (50 mm Tris–HCl pH 7.4, 300 mm NaCl), followed by two additional washes with low‐salt buffer (50 mm Tris–HCl pH 7.4, 150 mm NaCl). The amount of hnRNPA2B1‐bound m6A RNAs were detected by Western blot analysis with anti‐m6A antibody.

### RNA Immunoprecipitation (RIP)

RIP experiments were employed using the Magna RIP RNA‐Binding Protein Immunoprecipitation Kit (Geneseed, CHINA) based on the manufacturer's instructions. Briefly, cells were washed with ice‐cold PBS and lysed in 1 mL RIP lysis buffer. After centrifuged (12, 000×g, 4 °C, 15 min), 10% of supernatant was utilized as input. Remaining extraction were incubated with suspended beads conjugated with hnRNPA2B1 antibody, ISG15 antibody or control IgG at 4 °C overnight and washed with RIP buffer four times. Then bead‐bound immunoprecipitate was digested with proteinase K at 55 °C and isolated by phenol/chloroform/isoamyl alcohol. The RNAs that co‐precipitated were subjected to detection using qRT‐PCR. The calculation of the IP enrichment ratio involved determining the ratio of the amount present in the immunoprecipitation (IP) samples to that in the input samples.

### Tumorigenesis Assay and Limiting Dilution Assay

Animal care guidelines approved by the Chongqing Medical University Experimental Animal Management Committee (license number: 2022117) were followed during all animal experiments. In the Laboratory Animal Center of the Chongqing Medical University, a pathogen‐free environment was established to ensure the well‐being of all mice.

For in vivo limiting dilution assay, the enriched mammosphere cells derived from engineered Hs578T/shPCAT6, MDA‐MB‐231/PCAT6, Hs578T/shNC and MDA‐MB‐231/vector sphere‐derived cells were used in Xenograft experiments. Three doses (1 × 10^5^, 1 × 10^4^, and 1 × 10^3^) of cells derived from the engineered Hs578T or MDA‐MB‐231 were subcutaneously inoculated into 6‐week‐old female nude mice (*n* = 7 per group). The tumor‐initiation frequency was calculated by employing the Extreme Limiting Dilution Analysis (http://bioinf.wehi.edu.au/software/elda/). For tumorigenesis assay, stably transfected BC spheres‐derived cells (1 × 10^6^) were suspended in 100 mL PBS, and then were injected subcutaneously into the mammary fat pads of 6‐week‐old BALB/C nude mice (*n* = 5 each group). Once tumor volumes were reached 100 mm^3^, each group were treated with DMSO, STM2457 (50 mg kg^−1^, every other day), Doxorubicin (2 mg kg^−1^, every three days) or STM2457 combined with doxorubicin (every three days) respectively. After 16 days, the nude mice were sacrificed, and tumors were harvested. For proper preservation, the tumors were snap‐frozen in liquid nitrogen or embedded in paraffin. Finally, the weight and volume of the tumors were measured (tumor volume = length × width^2^ × 1/2). The acquired tumor tissues were subjected to immunohistochemistry staining to detect CD44, Ki67, c‐MYC and FGFR1 expressions.

### Statistical Analysis and Data Availability

The data are presented as individual. The statistical methods employed in this study included two‐tailed unpaired or paired Student's t‐test, non‐parametric signed rank test, Mann–Whitney U test, ANOVA (with Dunnett's or LSD post hoc test), and Pearson correlation coefficients, depending on the type of the experiment. GraphPad prism 9 software were utilized for all data analyses. Significance was determined at *p* <0.05.

### Ethics Approval Statement and Patient Consent Statement

All human tumor tissue samples were collected in accordance with national and institutional ethical guidelines. The clinical patient sample collection was approved by the Research Ethics Committee of Chongqing Medical University (Reference number: 2022117). Animal care guidelines approved by the Chongqing Medical University Experimental Animal Management Committee (Reference number: 2022117) were followed during all animal experiments.

## Conflict of Interest

The authors declare no conflict of interest.

## Author Contributions

T.J. and L.Y. contributed equally to this work. T.J., L.Y., and M.L. designed this study; T.J., L.Y., C.C., R.W., Y.G., Y.G., R.T., and P.D. performed the experiments; H.L., Y.S., S.C., and Z.L. collected clinic samples and analyzed the data; T.J. and L.Y. organized the figures and drafted the initial manuscript; M.L. revised this manuscript; all authors read and approved the final manuscript.

## Supporting information

Supporting Information

## Data Availability

The data that support the findings of this study are available from the corresponding author upon reasonable request.
