# Supplementary material for: HnRNPA2B1 ISGylation Regulates m6A‐Tagged mRNA Selective Export via ALYREF/NXF1 Complex to Foster Breast Cancer Development
Source: Adv Sci (Weinh). 2024 Apr 16;11(24):2307639. doi: 10.1002/advs.202307639 (PMC11200088; doi:10.1002/advs.202307639)
Supplement: Supplementary file 1 — Supporting Information [file ADVS-11-2307639-s001.pdf]

ADVANCED SCIENCE
Open Access

Supporting Information

for *Adv. Sci.*, DOI 10.1002/advs.202307639

HnRNPA2B1 ISGylation Regulates m6A-Tagged mRNA Selective Export via ALYREF/NXF1 Complex to Foster Breast Cancer Development

*Ting Jin, Liping Yang, Chao Chang, Haojun Luo, Rui Wang, Yubi Gan, Yan Sun, Yuetong Guo, Rui Tang, Shanchun Chen, Die Meng, Peijin Dai and Manran Liu**

Supplemental Figures

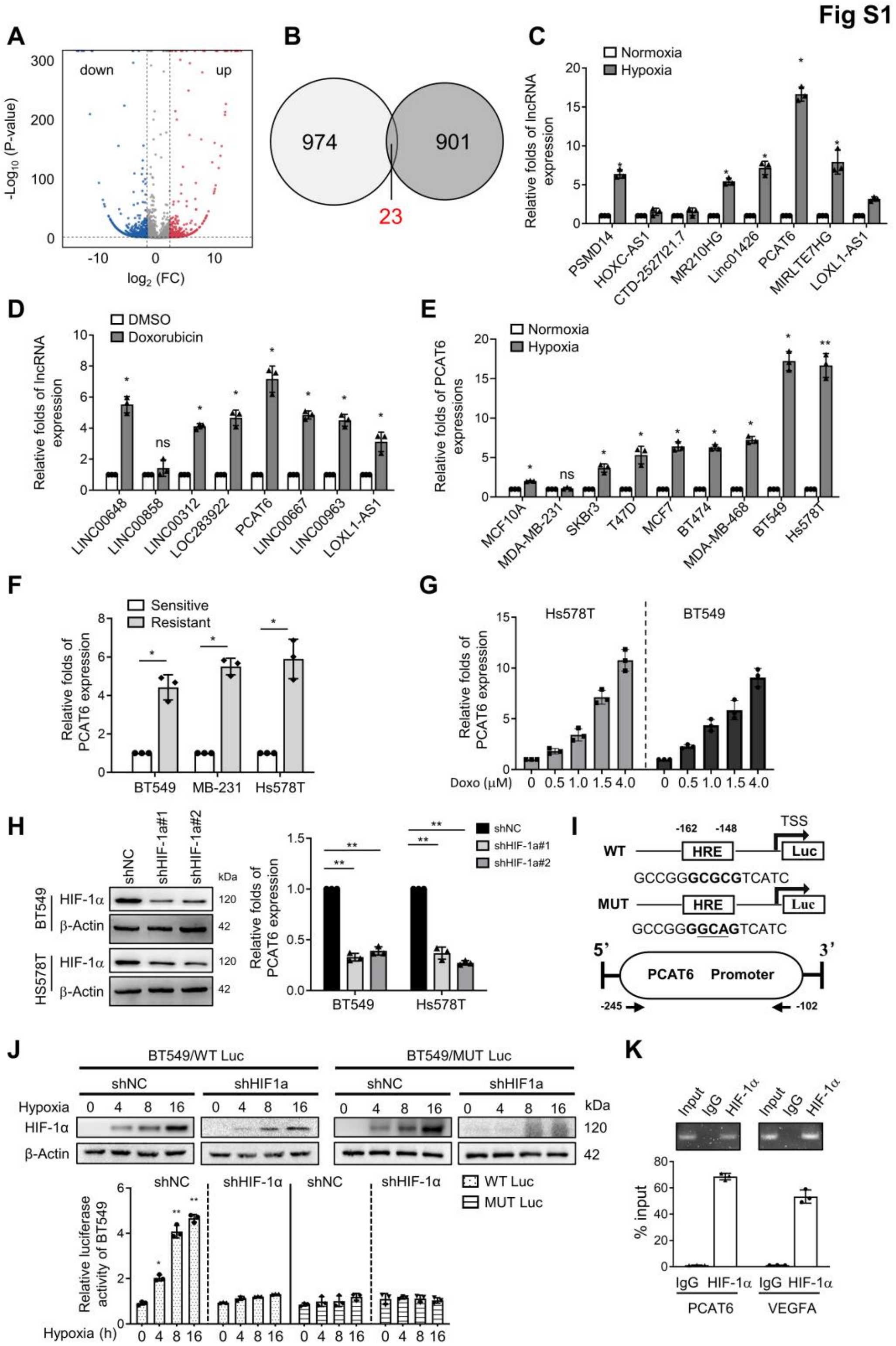

Figure. S1. HIF-1α reduced highly expression of PCAT6 in BC cells

A. The Volcano plot from RNA sequence analysis showing the differentially expressed lncRNAs in hypoxic BT549 cells compared with normoxic BT549 cells. **B.** Venn diagram shows the up-regulated lncRNAs in hypoxic BC cells and doxorubicin resistant BC cells. **C.** The levels of 8 randomly selected up-regulated lncRNAs were verified by qRT-PCR in hypoxic BT549 cells compared with normoxic BT549 cells. **D.** The levels of 8 randomly selected up-regulated lncRNAs were verified by qRT-PCR in BT549 cells treated with DMSO or doxorubicin. **E-G.** qRT-PCR was performed to determine the PCAT6 levels in various normoxia and hypoxia BC cells (E), or in doxorubicin resistant BC cells (F), or in BT549 and Hs578T BC cells treated with different dose of doxorubicin (G). **H.** Western blotting (left panel) was performed to determine the knockdown efficiency of HIF-1α in the indicated breast cancer cells under hypoxia. qRT-PCR (right panel) was performed to determine the PCAT6 levels in HIF-1a knockdown BC cells under hypoxia. **I.** Schematic illustration of the putative hypoxia-response element (HRE) in the promoter of PCAT6 gene. **J.** BT549 cells were transfected with pGL3-PCAT6 WT reporter, pGL3-PCAT6 MUT reporter or the control

r

e

p

o

r

t

e

r

c

o

m

b

i

n

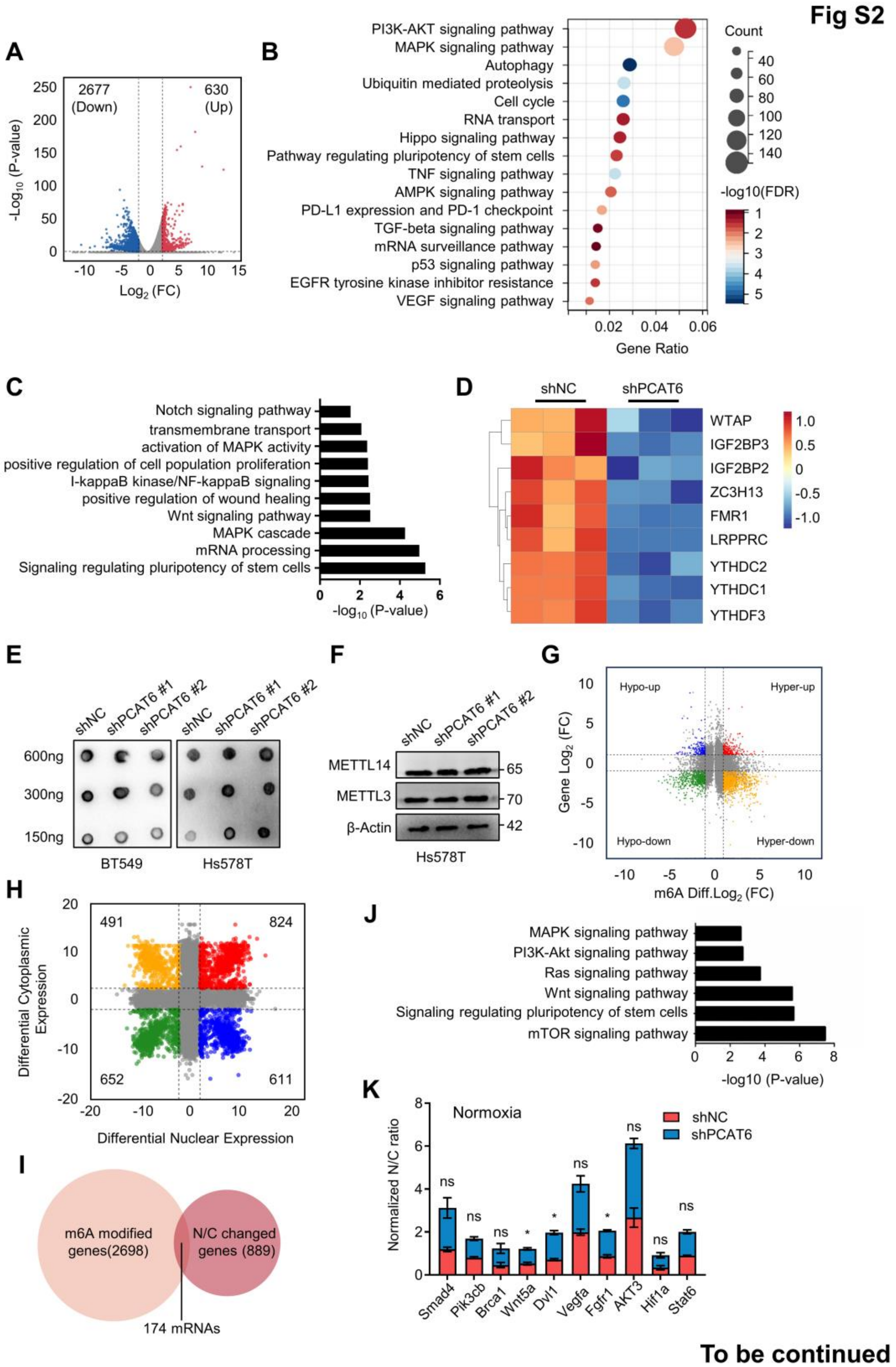

To be continued

Continued

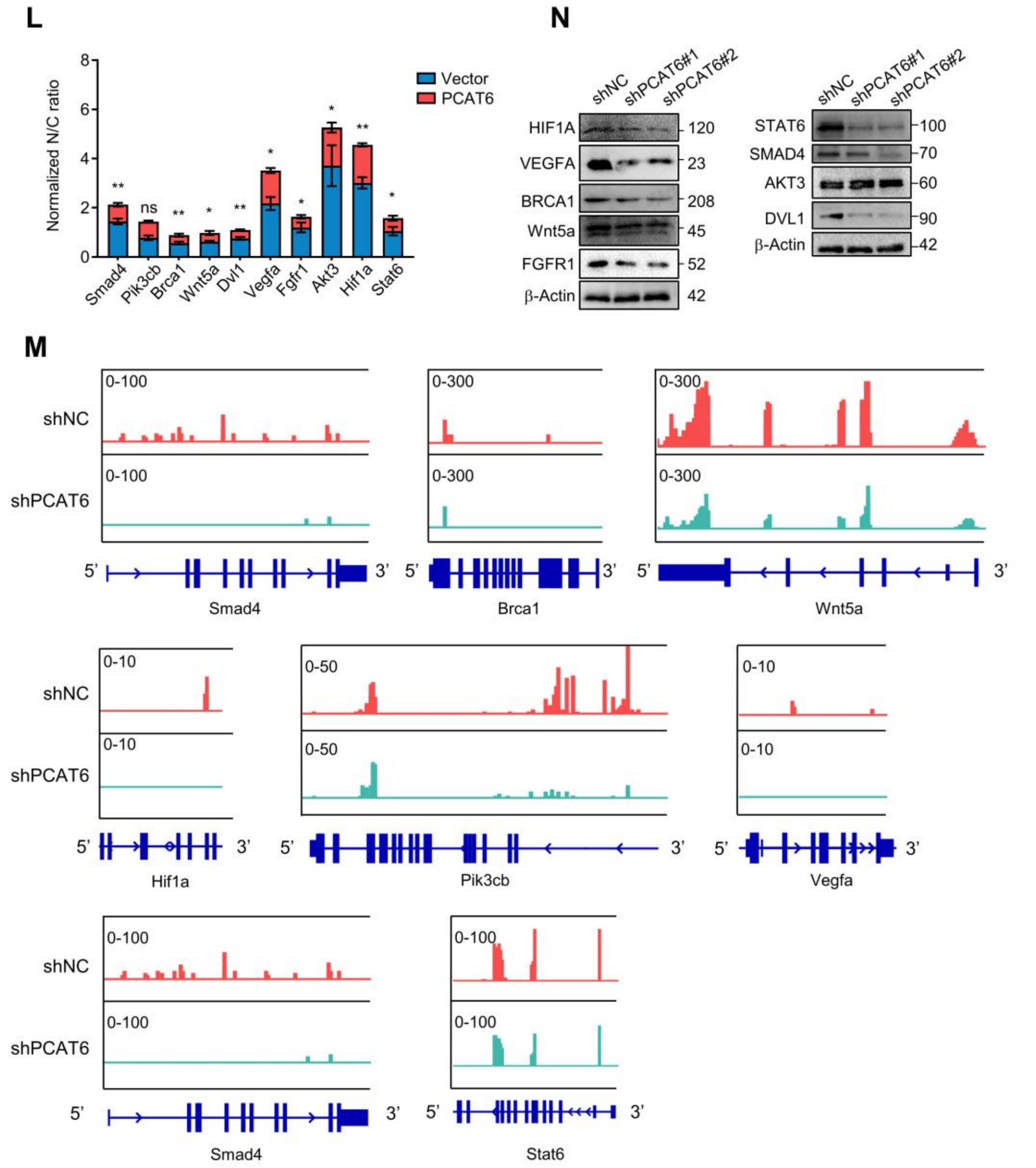

Figure S2. Related to Figure 2

A. Volcano plot showing the differential expression genes (DEGs) based on RNA-seq in shNC/Hs578T and shPCAT6/Hs578T cells. **B-C.** KEGG (B) or GO (C) enrichment scatter plot of DEGs in shNC/Hs578T and shPCAT6/Hs578T cells. **D.** Heatmap of DEGs related with m6A regulators in shNC/Hs578T and shPCAT6/Hs578T cells. **E.** The m6A levels of whole mRNAs in PCAT6 knockdown (shPCAT6#1, shPCAT6#2) and control BC cells were detected by dot blotting using an anti-m6A antibody. The total RNAs (600 ng) were double diluted and spotted onto a Hybond-N^+ membrane. **F.**

Western blot analysis of MTases (METTL3, METTL14) in cell lysates of PCAT6 knockdown and control BC cells under hypoxia environment. **G.** Volcano plot showing m6A mRNAs and DEGs in PCAT6 knocked down and control Hs578T cells. **H.** Volcano plot showing the nuclear and cytoplasmic mRNA levels from RNA-seq. **I.** Venn diagram showing 174 mRNAs both m6A-differential genes and nuclear retention genes in PCAT6 knockdown Hs578T cells. **J.** KEGG enrichment analysis of DEGs between PCAT6-silenced (shPCAT6/Hs578T) and control cells (shNC/Hs578T). **K.** The nuclear and cytoplasmic mRNA levels in shNC/Hs578T and shPCAT6/Hs578T cells under normoxia were detected by qRT-PCR. The data are represented as mean ± SD. **L.** qRT-PCR were performed to determine the nuclear (N) and cytoplasm (C) expression of differential expression mRNA in ectopic PCAT6 overexpressing and control Hs578T cells. The data are represented as mean ± SD. **M.** Integrative genomics viewer (IGV) tracks showing the m6A peak distribution in the mRNA transcript derived from MeRIP-seq data in PCAT6 knockdown and control BC cells. **N.** Western blot analysis in cell lysates from PCAT6 knockdown and control BC cells under hypoxia. (*$p < 0.05$, **$p < 0.005$, ***$p < 0.001$.)

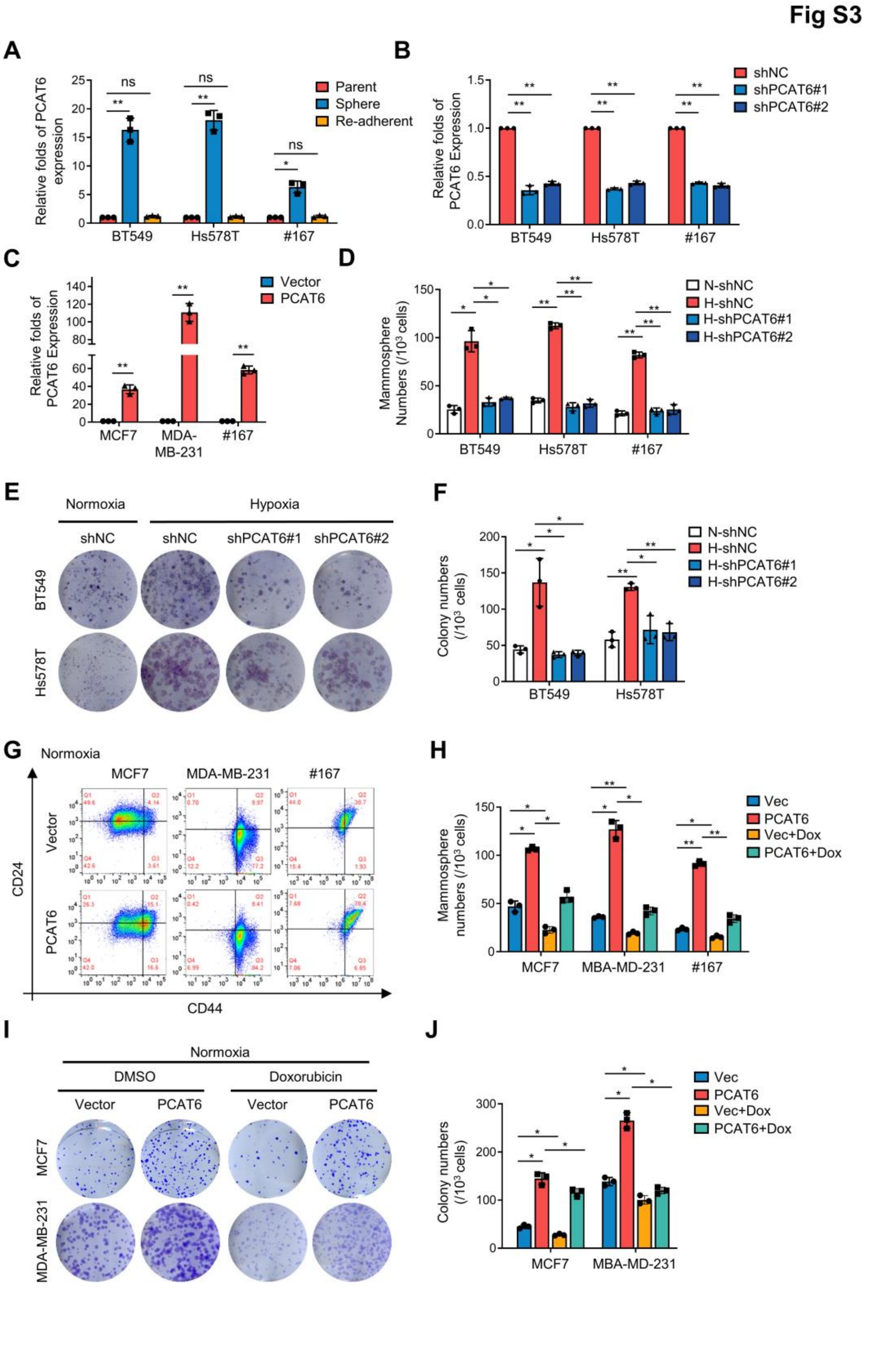

Figure S3. PCAT6 is required for the maintenance of BCSCs properties

A. qRT-PCR analysis of PCAT6 levels in parental breast cancer cells, spheres and re-adherent sphere cells. **B-C.** qRT-PCR analysis of PCAT6 levels in hypoxic BC cells

transfected with shNC (shNC), shPCAT6#1 and shPCAT6#2 (B), or in normoxic BC cells transfected with control lentivirus (Vector) and PCAT6-overexpressed lentivirus (PCAT6) (B). **D-F.** Mammospheres numbers (D) and representative colony formation images (E) and colony numbers (F) of PCAT6 knockdown and control BC cells under normoxia (labeled by N) or hypoxia (labeled by H) were shown. **G.** The proportion of $CD44^{+}/CD24^{-}$ BC cells was determined using flow cytometry. Data shown are from representative of three or more independent experiments. **H-J.** Mammospheres numbers (H) and representative colony formation images (I) and colony numbers (J) of ectopic PCAT6 overexpressing and control BC cells treated with DMSO or doxorubicin under normoxia were shown.

Fig S4

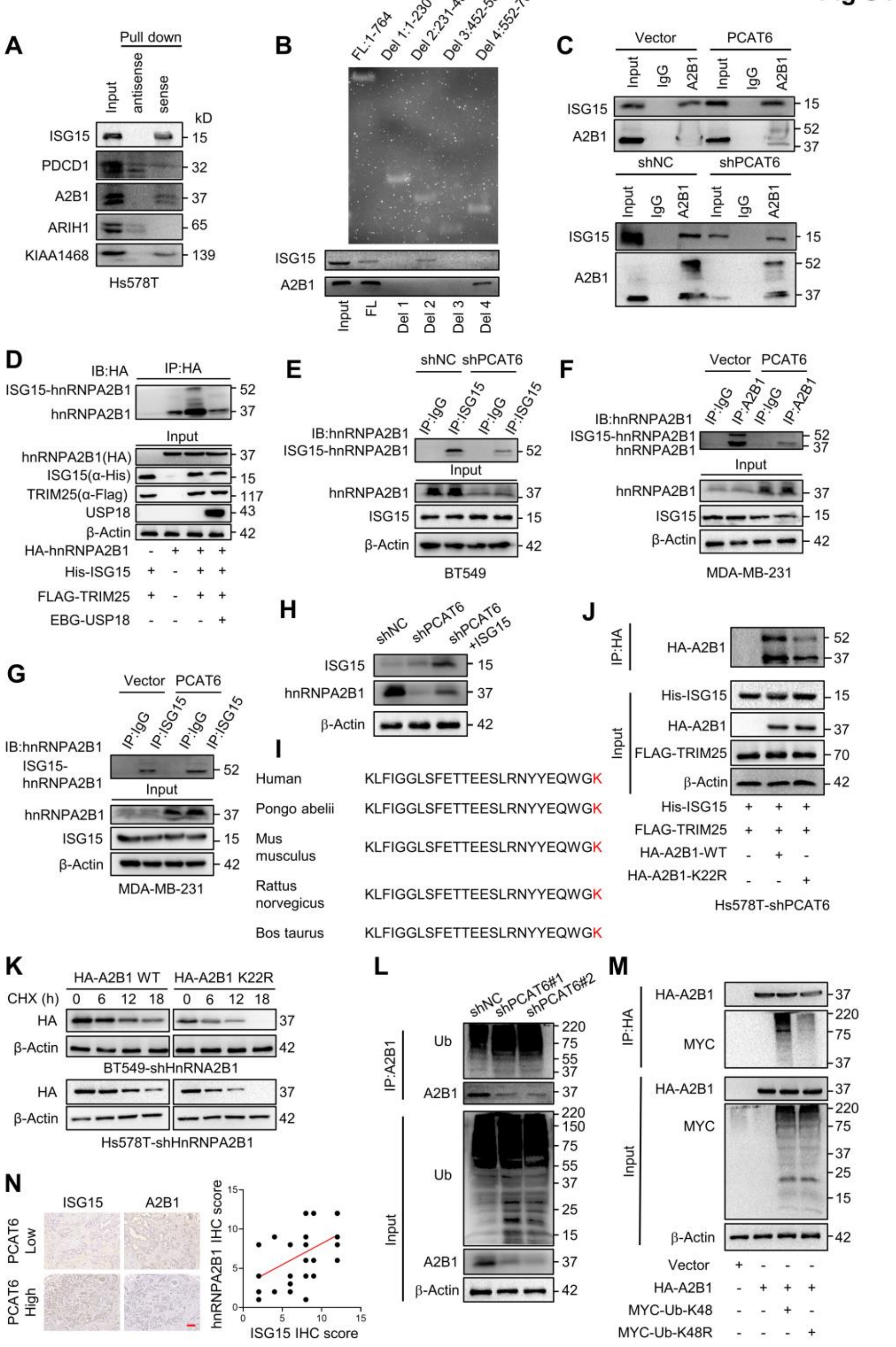

Figure S4. ISGylation of HnRNPA2B1 is regulated by PCAT6 in BC cells

A Immunoblot (IB) detection of proteins pulled down by PCAT6 in Hs578T cells. **B.** Immunoblot (IB) detection of ISG15 and hnRNPA2B1 pulled down by in vitro transcribed biotinylated RNAs corresponding to full length (FL) or different fragments

of PCAT6 (DEL1, DEL2, DEL3, DEL4) in HEK293T cells. **C.** Co-IP showing the mutual binding between ISG15 and hnRNPA2B1 in ectopic PCAT6 overexpression and control MDA-MB-231 cell (up panel) and in hypoxic PCAT6 knockdown (shPCAT6) and control (shNC) Hs578T cells (down panel). **D-G.** ISGylation of hnRNPA2B1 was measured in the indicated cells. In 293T cells (D), HA-hnRNPA2B1 and His-ISG15/Flag-TRIM25 were transfected with or without EBG-USP18 into HEK-293T cells as indicated. ISG15 conjugated hnRNPA2B1 proteins in cell lysates from 293T cells, PCAT6 knockdown BT549 cells (E), PCAT6 overexpressing MDA-MB-231 cells (F, G) and their control cells were immunoblotted with anti-HA, anti-ISG15, anti-hnRNPA2B1 antibody respectively, and then immunoblotted with indicated antibodies as shown. Total cell lysates were also immunoblotted with indicated antibodies. **H.** Western blot analysis of ISG15 and hnRNPA2B1 in cell lysates from PCAT6 knockdown or PCAT6 knockdown transfected with ISG15 and control BC cells. **I**. Cross-species sequence alignment of hnRNPA2B1. **J.** ISGylation of hnRNPA2B1 was measured in PCAT6-knockdown cells transfected with HA-hnRNPA2B1-WT or HA-hnRNPA2B1-K22R. Cell lysates were used for IP with anti-HA antibody, then immunoblotted with anti-HA. Total cell lysates were immunoblotted with indicated antibodies. **K**. Half-life of hnRNPA2B1 in BT549 and Hs578T cells were investigated. HnRNPA2B1 knockdown cells were transfected with hnRNPA2B1 wild type or hnRNPA2B1 K22R plasmid and exposure to cycloheximide (CHX) for the designed time. HnRNPA2B1 levels were assessed by immunoblotting. **L**. Total ubiquitination of hnRNPA2B1 was measured in PCAT6- knockdown cells. Cell lysates used for IP were incubated with anti-hnRNPA2B1 antibody, then immunoblotted with anti-hnRNPA2B1 (labeled as A2B1) and anti-MYC antibodies as indicated. Total cell lysates were immunoblotted with indicated antibodies. **M**. K48-linkage ubiquitination of hnRNPA2B1 was measured in 293T cells transfected with HA-hnRNPA2B1, MYC-Ub-K48 or MYC-Ub-K48R. **N.** Images of IHC analysis of ISG15 and hnRNPA2B1 from breast cancer tissues array. Original magnification, ×200. Scale bars, 100 μm. The

dot plot showing the expression correlation between ISG15 and hnRNPA2B1 from 38 BC tissues. (*p < 0.05, **p < 0.01)

Fig S5

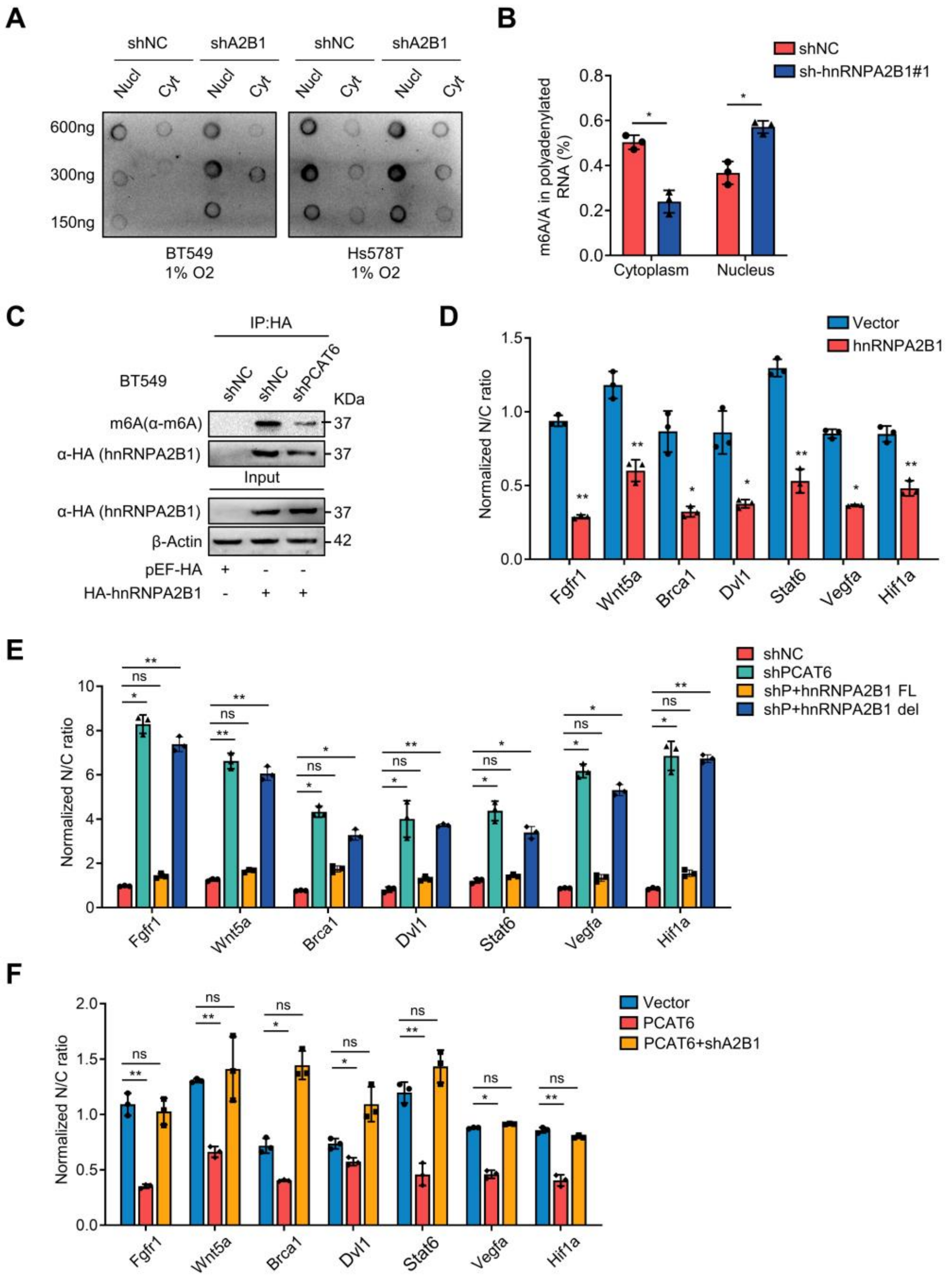

Figure S5. Related to Figure 5

A. Dot blotting showing m6A levels of nuclear and cytoplasmic mRNAs in hnRNPA2B1 knockdown and control BC cells using an anti-m6A antibody. The total

RNAs (600 ng, 300ng, 150ng) were spotted onto a Hybond-N^+ membrane. **B.** The m6A levels of cytoplasmic and nuclear mRNAs were detected via LC-MS/MS in hnRNPA2B1 knockdown and control BC cells. **C.** The UV cross-linked BC cells transfected with HA-hnRNPA2B1 were lysed for immunoprecipitated with anti-HA. The m6A levels of mRNA bound with hnRNPA2B1 in PCAT6 knockdown and control BC cells were detected by immunoblotting. **D-F.** qRT-PCR were conducted to test the nuclear and cytoplasmic levels of mRNAs in hnRNPA2B1 overexpressing tumor cells (D), PCAT6 knockdown hypoxic Hs578T cells with hnRNPA2B1 full length (labeled as A2B-FL) or hnRNPA2B1 deletion (labeled as A2B1-del) (E), and hnRNPA2B1 knocked down tumor cells with or without ectopic PCAT6 (F) and their control Hs578T cells. (ns, no significant; *$p < 0.05$, **$p < 0.01$.).

Supplemental Tables

Table S1. Oligos for shRNA

Oligos for shRNA	
shHIF1α#1	5’-CCGCUGGAGACACAAUCAUAU-3’
shHIF1α#2	5’-CCAUAUAGAGAUACUCAAATT-3’
shPCAT6#1	5’-CCAUCCUCAUUCGGUCCAUTT-3’
shPCAT6#2	5’-AAAUCGCAAGCGUUUGUGGTT-3’
shISG15#1	5’-GCACCGUGUUCAUGAAUCUTT-3’
shISG15#2	5’-GCAUCCUGGUGAGGAAUAATT-3’
sh-hnRNPA2B1	5'-GCTTCTTCCTATTTGCCATGG-3'
shCRM1	5'-CAGCTATATTTGCCCATGTTA-3'
shALYREF	5‘-TGGGAAACTGCTGGTGTCCAA-3’
shNXF1	5‘-CGCGAACGATTTCCCAAGTTA-3’
shFGFR1#1	5'-GCATGATGAAAGGTGTCAATA-3'
shFGFR1#2	5’- CGAGGCATTATTTGACCGGAT-3’
shMETTL14	5'-AAGGATGAGTTAATAGCTAAA-3'
Pull down	
PCAT6 sense F	5'-taatacgactcactataggg gacgccccagaggccggacc-3'
PCAT6 sense R	5'-ctgagttataagtgttgtaata-3'
PCAT6 anti-sense F	5'-taatacgactcactataggg ctgagttataagtgttgtaata-3'
PCAT6 anti-sense R	5'-gacgccccagaggccggacc-3'

Table S2. Primers for quantitative RT-PCR

Gene		
PCAT6	Forward	5’-GCTTTCCCTCGTCCTCTGCG -3’
	Reverse	5’-GCAAGCGTTTGTGGGTTTCA -3’
HIF1a	Forward	5’-GAACGTCGAAAAGAAAAGTCTCG -3’
	Reverse	5’-CCTTATCAAGATGCGAACTCACA -3’
β-Actin	Forward	5’-CCACTGGCATCGTGATGGA -3’
	Reverse	5’-CGCTCGGTGAGGATCTTCAT -3’
AC010894.3	Forward	5’-CCAAGTACAGACGCAGAGCA -3’
	Reverse	5’-TCATAGGGCCACCAAACACA -3’
PSMD14	Forward	5’-GTCACCACAGAGGCAAGACA -3’
	Reverse	5’-GCCAGGGTGACTGTGATACC -3’
HOXC-AS1	Forward	5’-CTCTGCGACACTTCCCCACC -3’
	Reverse	5’-AGCTACTTGCCCACGACCGA -3’
RP5-1198O20.4	Forward	5’-GTCTGCTGTTTCATTCAGTCCA -3’
	Reverse	5’-TGTCCATTTCAAGCTCCTTTCT -3’
hnRNPA2B1	Forward	5’-ATTGATGGGAGAGTAGTTGAGCC -3’
	Reverse	5’-AATTCCGCCAACAAACAGCTT -3’
ISG15	Forward	5'-CACAGCCATGGGCTGGGACCTG-3’
	Reverse	5‘-GCACGCCGATCTTCTGGGTGA-3’
METTL14	Forward	5’-CTCCCATGTACTTACAAGCC -3’
	Reverse	5’-TTAGCAGTGATGCCAGTTTC -3’
WNT5A	Forward	5’-ATTCTTGGTGGTCGCTAGGTA -3’
	Reverse	5’-CGCCTTCTCCGATGTACTGC -3’
SMAD4	Forward	5’- CTCATGTGATCTATGCCCGTC -3’
	Reverse	5’-AGGTGATACAACTCGTTCGTAGT -3’
MAPK8	Forward	5’- TGTGTGGAATCAAGCACCTTC -3’
	Reverse	5’- AGGCGTCATCATAAAACTCGTTC -3’
FGFR1	Forward	5‘-CCCGTAGCTCCATATTGGACA-3’
	Reverse	5‘-TTTGCCATTTTTCAACCAGCG-3’

VEGFA	Forward	5‘-AGGGCAGAATCATCACGAAGT-3’
	Reverse	5‘-AGGGTCTCGATTGGATGGCA-3’
AKT3	Forward	5‘-TGTGGATTTACCTTATCCCCTCA-3’
	Reverse	5‘-GTTTGGCTTTGGTCGTTCTGT-3’
STAT6	Forward	5’-TTCAGACACGAGAGATGGAACT-3‘
	Reverse	5’-CCAGCCTTCACTTGCTGAG-3‘
BRCA1	Forward	5'-GAAACCGTGCCAAAAGACTTC-3‘
	Reverse	5'-CCAAGGTTAGAGAGTTGGACAC-3‘
PIK3CB	Forward	5'-CCAAGGTTAGAGAGTTGGACAC-3‘
	Reverse	5'-GAGGTGAATTGAGGTCCCTAAGA-3‘
DVL1	Forward	5'-GAGGGTGCTCACTCGGATG-3‘
	Reverse	5'-GTGCCTGTCTCGTTGTCCA-3‘

Table S3. Correlation between PCAT6 expression and chemo-resistance features in 38 BC patients

Characteristic		All cases	PCAT6		Chi-square	*P* value
			Low	High		
Age	<50	18	8	10	0.6496	0.5158
	≥50	20	11	9		
Chemo-resistance	Yes	19	5	14	6.7560	0.0093**
	No	19	13	6		

Table S4. Correlation between ISG15 expression and hnRNPA2B1 expression in 38 BC patients

Characteristic		All cases	ISG15		Chi-square	*P* value
			Low	High		
Age	<50	18	11	7	0.0049	0.9442
	≥50	20	12	8		
Chemo-resistance	Yes	19	8	11	5.3970	0.0202*
	No	19	15	4		
hnRNPA2B1 expression	Low	23	17	6	4.3700	0.0366*
	High	15	6	9		

Table S5.1. Proteins specifically pulled down by PCAT6

Proteins Unique Sequence ID	Accession	Abundances (Normalized): PCAT6_antisense	Abundances (Normalized): PCAT6_sense	Abundance: PCAT6_antisense	Abundance: PCAT6_sense	Coverage [%]	# Peptides	# PSMs	# Unique Peptides	# AAs	calc. pI	Score Sequest HT: Sequest HT	# Peptides (by Search Engine): Sequest HT	Exp. q-value: Combined	Gene Symbol	Found in Sample: PCAT6_antisense	Found in Sample: PCAT6_sense
-1862365170183320000	P56182		466118.1236		253111.2	3	1	3	1	461	9.33	3.59	1	0	RRP1	High	High
8106811875095750000	P06454		311644.8617		169229.2	9	1	1	1	111	3.78	0	1	0.006	PTMA	Not Found	High
4746768512254430000	Q9Y4X5		659537.1595		358141.5	4	2	2	2	493	5.63	1.77	2	0	ARIH1	High	High

8005883181375690000	Q9P260		759161.8729		412239.7	1	1	3	1	1216	5.45	0	1	0.009	KIAA1468	High	High
-4577230366185340000	Q96GM8		1112616.587		604172.4	4	1	1	1	510	7.18	2.24	1	0.003	TOE1	Not Found	High
8144354822325390000	Q16799		2335473.932		1268208	2	2	5	1	776	4.69	5.85	2	0	RTN1	High	High
-4188399694065690000	P06732		303703.0708		164916.7	6	2	2	1	381	7.25	0	2	0	CKM	Not Found	High
484436176042567000	P02686		1267223.85		688127.2	3	1	1	1	304	9.79	0	1	0.007	MBP	Not Found	High
132073470049357000	Q58FF6		1150830.113		624923.1	14	8	35	1	505	4.73	45.73	8	0	HSP90AB4P	Not Found	High
-4288345364225070000	Q6ZVX7		1613183.983		875990.3	4	1	3	1	275	6.62	7.41	1	0	NCCRP1	Not Found	High

577500699946211000	Q8NFQ8		118603.8264		64404.18	2	1	1	1	470	4.96	0	1	0.009	TOR1AIP2	Not Found	High
6216510276447140000	P01112		141900.5076		77054.73	15	3	4	1	189	5.31	4.83	3	0	HRAS	Not Found	High
5033542782093600000	P05062		794395.5907		431372.3	5	2	5	1	364	7.87	8.71	2	0	ALDOB	Not Found	High
31313560900359600	P60201		936712.5486		508653.1	3	1	1	1	277	8.35	0	1	0.007	PLP1	Not Found	High
6955307407812840000	P02511		708243.1735		384589.8	5	1	1	1	175	7.33	0	1	0.009	CRYAB	Not Found	High
3509057874585170000	Q9GZS1		278114.841		151021.8	5	2	2	2	419	8.94	1.73	2	0		High	High
-5144804986028240000	P08727		2693246.559		1462485	18	11	70	1	400	5.14	95.61	11	0	KRT19	Not Found	High

7228226931451830000	O15084		191805.4024		104154.1	1	1	2	1	1053	6.25	1.83	1	0	ANKRD28	High	High
-8172346971524840000	Q13126		399250.093		216800.6	10	2	2	2	283	7.18	2.08	2	0	MTAP	High	High
-2440246384858700000	P36952		911136.2746		494764.7	5	2	2	2	375	6.05	1.69	2	0	SERPINB5	Not Found	High
-8621110072198190000	Q15116		12392526.83		6729383	6	2	4	2	288	8.02	3.92	2	0	PDCD1	Not Found	High
2351772574129650000	P35269		369425.8845		200605.4	3	1	1	1	517	7.49	0	1	0.01	GTF2F1	Not Found	High
-8226351996113540000	P48594		388889.9379		211174.8	8	3	5	1	390	6.21	0	3	0	SERPINB4	Not Found	High

7800791673536880000	P05067		2607473.631		1415909	3	2	3	2	770	4.82	2.14	2	0	APP	High	High
-2261861031269980000	Q9Y5I4		989613.5091		537379.4	1	1	2	1	1007	5.41	1.64	1	0.009	PCDHAC2	High	High
7876379763611590000	Q9UNX3		1053247.971		571934.1	44	10	31	1	145	10.55	44.07	10	0	RPL26L1	Not Found	High
5432716996293130000	P05161		627419.1293		340700.8	12	2	2	2	165	7.44	0	2	0.001	ISG15	Not Found	High
1074215629290590000	P22626		106585399.9		57877944	47	16	78	14	353	8.95	134.18	16	0	HNRNPA2B1	Not Found	High

Table S5.2. Proteins pulled down by PCAT6 sense and PCAT6 antisense

Proteins Unique Sequence ID	Accession	Abundances (Normalized): PCAT6_antisense	Abundances (Normalized): PCAT6_sense	Abundance: PCAT6_antisense	Abundance: PCAT6_sense	Coverage [%]	# Peptides	# PSMs	# Unique Peptides	# AAs	calc. pI	Score Sequest HT: Sequest HT	# Peptides (by Search Engine): Sequest HT	Sum PEP Score	Exp. q-value: Combined	Gene Symbol	Found in Sample :PCAT6_antisense	Found in Sample: PCAT6_sense
5362975432766660000	P13646	1094099	29769793	1094099	16165577	28	16	59	7	458	4.96	93.28	16	77.303	0	KRT13	High	High
-2761647676892230000	P68871	995222.7	12469312	995222.7	6771079	24	3	8	1	147	7.28	10	3	13.95	0	HBB	Peak Found	High
7521439039700900000	P19013	2131374	23615138	2131374	12823479	38	19	37	12	520	6.61	51.06	19	69.65	0		High	High

-6590876033132110000	P29508	402614.1	4430974	402614.1	2406105	10	4	7	2	390	6.81	1.67	4	10.813	0	SERPINB3	Peak Found	High
-8841542805290730000	P69905	999575.6	10621486	999575.6	5767673	30	4	9	4	142	8.68	12.43	4	15.68	0	HBA2; HBA1	High	High
-2575504775015730000	P05090	586361	6000013	586361	3258124	10	2	5	2	189	5.15	5.09	2	5.174	0	APOD	High	High
3885122189717850000	P47929	1832934	15653986	1832934	8500419	33	4	5	4	136	7.62	12.94	4	19.969	0	LGALS7; LGALS7B	Peak Found	High
9215017324203500000	Q6Y7W6	1553198	11734978	1553198	6372321	2	2	3	2	1299	5.54	1.72	2	5.227	0	GIGYF2	High	High
-3690320084066520000	P04259	5704816	40418807	5704816	21948198	55	40	157	4	564	8	292.88	40	179.343	0	KRT6B	High	High
8250154090209620000	Q9H2W6	137358.5	877524.9	137358.5	476513.1	8	2	2	2	279	7.05	0	2	4.818	0	MRPL46	High	High

8006254010702620000	P08779	24081718	1.4E+08	24081718	75971940	58	31	158	13	473	5.05	251.24	31	185.952	0	KRT16	High	High
5219195248541820000	Q92609	301979.1	1668597	301979.1	906080.7	7	4	4	4	795	6.54	1.69	4	9.168	0	TBC1D5	High	High
2151926267082360000	A8K2U0	144871.4	793145.3	144871.4	430693.3	1	2	2	2	1454	5.73	0	2	3.518	0.002	A2ML1	Peak Found	High
-7423866606079620000	Q9Y3M8	152532.2	808718.7	152532.2	439150	2	2	2	2	1113	7.02	0	2	4.708	0	STARD13	High	High
8176798073003430000	Q08554	4088184	19882932	4088184	10796819	7	5	8	5	894	5.43	13.78	5	20.293	0	DSC1	High	High
-7661195779620970000	P22735	574382.5	2723196	574382.5	1478749	10	7	9	7	817	6.04	4.45	7	21.426	0	TGM1	High	High
-3688625229951110000	P02538	1.4E+08	6.61E+08	1.4E+08	3.59E+08	54	42	147	7	564	8	278.75	42	189.698	0	KRT6A	High	High

9014625147701870000	Q5T160	518619.6	2435254	518619.6	1322390	8	5	6	5	578	8.21	5.91	5	12.039	0	RARS2	High	High
-4047471753011710000	P30408	27777214	1.28E+08	27777214	69357616	4	1	2	1	202	4.98	2.61	1	7.405	0	TM4SF1	High	High
2562963312153690000	P12883	305569.7	1395206	305569.7	757623.9	2	3	8	2	1935	5.8	15.5	3	12.487	0	MYH7	Peak Found	High
-8065211085214910000	Q96EL3	188030.3	851163.2	188030.3	462198.2	9	1	1	1	112	8.76	0	1	2.998	0.003	MRPL53	Peak Found	High
4258123771778810000	P55265	3780291	17010901	3780291	9237250	14	15	22	15	1226	8.65	22.3	15	51.45	0	ADAR	High	High
-8446105722917800000	P05121	2930867	13187166	2930867	7160887	11	5	8	5	402	7.2	3.97	5	15.32	0	SERPINE1	High	High
7105726009165370000	P13995	574462.8	2507885	574462.8	1361830	7	2	4	2	350	8.73	4.38	2	7.065	0	MTHFD2	High	High

8569081594674820000	Q9C075	121245.4	515941.6	121245.4	280166.3	5	2	2	1	422	6.54	3.66	2	6.031	0	KRT23	Peak Found	High
2087285006199930000	P61626	3329960	13557434	3329960	7361950	26	4	9	4	148	9.16	11.23	4	11.837	0	LYZ	High	High
-2891424664368030000	Q8N6T3	610590.1	2475951	610590.1	1344490	6	2	3	2	406	5.66	1.67	2	6.338	0	ARFGAP1	High	Peak Found
-6197247688658880000	Q8N1B4	289403.7	1145942	289403.7	622269	6	3	3	3	723	5.99	0	3	6.323	0	VPS52	High	High
-8021151771097250000	P02042	766416.3	2968379	766416.3	1611888	24	3	8	1	147	8.05	6.52	3	9.008	0	HBD	Peak Found	High
-6470269132294440000	Q8WUM0	2386259	9209796	2386259	5001098	6	6	7	6	1156	5.1	5.68	6	14.715	0	NUP133	High	High
-614997697265	P38435	338433.1	1239166	338433.1	672891.3	3	2	5	2	758	8.02	4.37	2	6.406	0	GGCX	High	High

4370000																		
588939397575272000	Q7L2J0	106673.5	386545.3	106673.5	209901.6	1	1	2	1	689	9.57	0	1	2.181	0.009	MEPCE	Peak Found	High
5631363744109010000	Q04695	4388439	15830903	4388439	8596488	48	24	103	8	432	5.02	163.07	24	113.862	0	KRT17	High	High
8891268094734710000	Q13428	3094167	10940598	3094167	5940958	1	2	3	2	1488	9.04	1.69	2	4.024	0	TCOF1	High	High
592112739915171000	Q8NCG7	1130980	3977143	1130980	2159666	3	2	3	2	672	6.55	0	2	6.454	0	DAGLB	High	High
4330394968015130000	Q9P2R3	2176149	7603698	2176149	4128956	7	7	10	7	1169	6.1	4	7	21.153	0	ANKFY1	High	High
-2618679762531220000	Q14533	1392470	4865358	1392470	2641984	11	5	8	4	505	5.47	7.94	5	13.494	0	KRT81	Peak Found	High
1498700468213320000	Q96LI6	1938150	6722537	1938150	3650468	3	1	5	1	401	7.18	6.6	1	3.183	0.003	HSFY1; HSFY2	High	High
903456444922	O75223	97248947	3.34E+08	97248947	1.82E+08	24	5	6	5	188	5.14	6.05	5	14.262	0	GGCT	High	High

8540000																		
-1474010075378450000	P15924	24190506	81783229	24190506	44409883	17	50	91	50	2871	6.81	89.75	50	147.041	0	DSP	High	High
-9059852176619990000	P31944	2676328	9032903	2676328	4905042	18	4	8	4	242	5.58	12.61	4	16.495	0	CASP14	High	High
4559835886279860000	O75342	888378.7	2981985	888378.7	1619276	6	4	6	4	701	7.64	2.45	4	11.228	0	ALOX12B	High	High
7253720687934920000	P19525	10598846	35250015	10598846	19141443	28	13	37	13	551	8.4	56.07	13	73.303	0	EIF2AK2	High	High
-3753879959614670000	P24821	539707	1723278	539707	935773.4	2	4	5	4	2201	4.89	3.8	4	9.675	0	TNC	High	High
945585302651271000	P43243	33132572	1.05E+08	33132572	57200140	25	21	71	21	847	6.25	99.57	21	102.963	0	MATR3	High	High
-599884902691	Q9BXL6	1210703	3837177	1210703	2083662	1	1	1	1	1004	5.92	0	1	2.724	0.005	CARD14	Peak Found	High

2210000																		
-823981804521467000	P46013	647678.3	2048294	647678.3	1112263	1	3	3	3	3256	9.45	0	3	6.893	0	MKI67	High	Peak Found
6765720071674470000	Q15633	573368.4	1812096	573368.4	984003.2	7	2	4	2	366	6.54	4.83	2	7.184	0	TARBP2	High	High
-6745372932164860000	P27482	284309.6	879971.3	284309.6	477841.5	24	3	5	2	149	4.42	3.64	3	7.241	0	CALML3	Peak Found	High
-3578102101740600000	Q01546	214038.3	656515.9	214038.3	356500.9	12	14	60	1	638	8.12	103.08	14	58.327	0	KRT76	Peak Found	High
4879216047058040000	P81605	33136822	1E+08	33136822	54512553	35	4	17	4	110	6.54	33.44	4	24.456	0	DCD	High	High
282539810863181000	Q9UL40	305403	922989.3	305403	501201.1	7	2	3	2	294	9.09	4.77	2	8.26	0	ZNF346	Peak Found	High
-140243199902963000	Q13835	629136.1	1887518	629136.1	1024959	3	2	9	2	747	9.13	17.03	2	14.228	0	PKP1	High	High

8066723703653810000	P53814	489898.8	1468369	489898.8	797353.1	2	2	2	2	917	9.07	0	2	4.167	0	SMTN	High	Peak Found
1879771635680970000	O95208	171659	513746.2	171659	278974.2	3	2	3	2	641	7.52	2.14	2	5.14	0	EPN2	High	High
1710541814749780000	P54619	8569814	25496963	8569814	13845347	12	4	9	4	331	6.92	1.77	4	12.042	0	PRKAG1	High	High
863607298942774000	Q16378	216314.9	639121.5	216314.9	347055.4	11	1	1	1	134	7.06	0	1	2.641	0.005	PRR4	Peak Found	High
2890344552958530000	O75569	1825006	5303126	1825006	2879700	17	4	7	4	313	8.41	9.92	4	21.053	0	PRKRA	High	High
1500672588244880000	P05089	3518095	10054849	3518095	5459979	16	5	7	5	322	7.21	10.31	5	17.489	0	ARG1	High	High
5735228591980060000	Q9HD42	1590116	4506292	1590116	2447004	9	2	2	2	196	8.06	3.85	2	4.542	0	CHMP1A	High	High
4058975179586110000	P23458	858843.6	2416990	858843.6	1312473	2	2	2	2	1154	7.55	0	2	3.509	0.002	JAK1	High	High

3230263422708410000	Q6UWP8	876117.9	2464429	876117.9	1338233	18	3	7	3	590	7.01	14.87	3	29.861	0	SBSN	High	High
-6534484608378340000	Q9H000	390769.1	1079681	390769.1	586287.7	5	2	5	2	416	7.61	0	2	5.108	0	MKRN2	High	High
-876330401098073000	P25311	2072277	5713810	2072277	3102710	23	6	14	6	298	6.05	16.91	6	23.469	0	AZGP1	High	High
-1775719120591890000	Q9UBC9	712299.3	1955590	712299.3	1061923	33	3	3	3	169	8.57	0	3	4.739	0	SPRR3	Peak Found	High
-5697908854691090000	P36406	704027.6	1925961	704027.6	1045834	1	1	4	1	574	6.38	1.79	1	2.093	0.01	TRIM23	High	High
-1703927075416440000	P07477	15543283	42228952	15543283	22931142	11	2	7	2	247	6.51	10.25	2	6.86	0	PRSS1	High	High
-857480523049	Q9Y3D7	188505.3	511762.8	188505.3	277897.1	7	1	1	1	125	9.7	0	1	2.548	0.006	PAM16	Peak Found	High

3060000																		
844941694654797000	P14923	11710965	31735511	11710965	17233000	32	20	44	18	745	6.14	52.64	20	75.908	0	JUP	High	High
-2039087024744640000	Q5D862	7731589	20865245	7731589	11330234	10	9	23	9	2391	8.31	60.18	9	97.872	0	FLG2	High	High
-6878293998477410000	Q86Y46	1067823	2877098	1067823	1562320	11	7	17	1	540	7.23	29.31	7	22.518	0	KRT73	Peak Found	High
3237045866174400000	P20930	3378241	9079156	3378241	4930158	3	7	14	7	4061	9.25	14.65	7	21.468	0	FLG	High	High
340121106600756000	Q9UPY3	204033.9	547619.7	204033.9	297368.1	1	2	2	2	1922	5.68	0	2	3.692	0.001	DICER1	Peak Found	High
6425786624467730000	O95758	1104906	2954646	1104906	1604430	12	6	14	3	552	9.04	12.57	6	20.889	0	PTBP3	High	High
-8225019653870580000	O76015	114363.8	304531.9	114363.8	165366.8	4	3	18	1	456	4.84	21.19	3	11.95	0	KRT38	Peak Found	High

-4814952899286620000	P31151	1540776	4052930	1540776	2200820	24	2	2	2	101	6.77	3.81	2	6.128	0	S100A7	High	High
621103420466231000	P12035	1252803	3285470	1252803	1784074	21	19	61	4	628	6.48	104.22	19	80.469	0	KRT3	Peak Found	High
1032132920388730000	Q6AZY7	4668734	12236728	4668734	6644781	2	1	1	1	606	6.54	0	1	2.138	0.01	SCARA3	Peak Found	High
-5599713626536470000	Q9GZZ8	365402.8	946688.3	365402.8	514070.1	17	2	3	2	138	5.5	3.8	2	6.474	0	LACRT	High	High
6885606707731030000	P59665	398395.4	1031469	398395.4	560107.6	19	2	3	2	94	6.99	1.96	2	5.433	0	DEFA1; DEFA1B	High	High
-2315785101654740000	Q8N556	799133.8	2065367	799133.8	1121534	2	2	2	2	730	8.68	2.02	2	4.269	0	AFAP1	High	High
6940581547763930000	Q9NUL3	9506930	24504879	9506930	13306626	28	15	31	14	570	9.61	41.51	15	57.791	0		High	High

-1347928425928220000	O60925	4451120	11453849	4451120	6219663	17	2	7	2	122	6.81	12.16	2	7.958	0	PFDN1	High	High
-567198980294406000	P06702	3794363	9684507	3794363	5258875	19	3	11	3	114	6.13	13.08	3	12.941	0	S100A9	High	High
3864726906583360000	Q7L3T8	183982.8	469383.9	183982.8	254884.6	3	1	2	1	475	8.1	0	1	2.914	0.004	PARS2	High	High
-942696880090980000	O95140	2424155	6080143	2424155	3301636	8	6	8	5	757	6.98	8.61	6	20.184	0	MFN2	High	High
-2024751910913560000	Q14210	155104.7	388474.4	155104.7	210949.2	7	1	1	1	128	8.21	0	1	2.096	0.01	LY6D	Peak Found	High
7628983176653610000	Q9UI47	1599008	3990022	1599008	2166660	2	2	10	1	895	6.16	17.58	2	6.681	0	CTNNA3	Peak Found	High
7926012984553530000	Q15517	454671.7	1130650	454671.7	613964.8	2	1	2	1	529	8.35	0	1	2.854	0.004	CDSN	High	High

2821542640267150000	P34741	2799810	6953440	2799810	3775853	13	3	5	3	201	4.86	2.74	3	10.091	0	SDC2	High	High
-4155367153969880000	P13645	1.47E+09	3.64E+09	1.47E+09	1.98E+09	50	39	275	30	584	5.21	505.37	39	274.993	0	KRT10	High	High
-8085176480319590000	Q08188	3028808	7500007	3028808	4072650	15	9	15	9	693	5.86	11.75	9	34.009	0	TGM3	High	High
-7376822484090120000	P05166	24718096	60470797	24718096	32836818	37	18	48	18	539	7.64	90.95	18	98.954	0	PCCB	High	High
-7631092510034680000	P31025	2182536	5256301	2182536	2854274	17	3	7	3	176	5.58	6.61	3	10.749	0	LCN1	High	High
-4144550306187800000	Q68E01	285189.9	683685.1	285189.9	371254.3	2	2	2	2	1043	5.8	0	2	4.648	0	INTS3	Peak Found	High
873608376829	P13647	1.89E+08	4.53E+08	1.89E+08	2.46E+08	55	42	196	27	590	7.74	361.66	42	227.301	0	KRT5	High	High

0230000																		
-6086246065885050000	P02533	7.07E+08	1.68E+09	7.07E+08	9.12E+08	74	42	197	16	472	5.16	312.8	42	249.705	0	KRT14	High	High
-7321332483161140000	O75477	680747	1615533	680747	877265.6	5	2	2	2	348	7.87	1.64	2	4.575	0		High	High
1307074189875800000	O14732	395687.6	938052.3	395687.6	509380.6	11	3	7	3	288	6.61	6.74	3	7.575	0	IMPA2	High	High
-2039602622287550000	P12273	956795	2257318	956795	1225768	27	5	7	5	146	8.05	1.61	5	13.663	0	PIP	High	High
-1860543310075140000	Q02413	11071608	25764107	11071608	13990411	15	12	37	12	1049	5.03	44.07	12	65.83	0	DSG1	High	High
1981774019348370000	P0DOY2	567393.6	1318788	567393.6	716127.8	9	1	2	1	106	7.24	2.35	1	4.112	0	IGLC2	Peak Found	High
-871817	Q14703	1294592	3004078	1294592	1631273	1	1	1	1	10	8.75	0	1	2.601	0.006	MBTPS1	High	Peak Found

4445000000000										52								
-5798091253452060000	Q86YZ3	34296727	79514640	34296727	43177996	24	23	68	23	2850	10.04	158.18	23	208.175	0	HRNR	High	High
6838394532557100000	Q8WVV4	377663.8	873155.7	377663.8	474140.5	2	1	2	1	589	6.32	2.02	1	2.771	0.004	POF1B	Peak Found	High
7019173360477080000	Q96QA5	1231529	2842967	1231529	1543786	6	3	6	3	445	5.29	7.45	3	7.195	0	GSDMA	High	High
-6605193621902280000	P35908	8E+08	1.84E+09	8E+08	9.97E+08	70	53	292	37	639	8	578.79	53	381.359	0	KRT2	High	High
1373534053104030000	O14653	694236.8	1585661	694236.8	861044.8	5	1	1	1	212	8.06	0	1	2.822	0.004	GOSR2	High	Peak Found
2478117011496910000	Q13206	444650.5	1012530	444650.5	549823.7	2	2	2	2	875	8.63	0	2	4.51	0	DDX10	High	Peak Found
423259825915	Q86Y56	2572625	5845576	2572625	3174261	2	2	4	2	855	6.42	0	2	4.374	0	HEATR2; DNAAF5	High	High

2990000																		
-4082560414167460000	P05109	4011756	8990576	4011756	4882058	38	4	7	4	93	7.03	10.82	4	14.388	0	S100A8	High	High
1999315358904820000	P01040	6393172	14298431	6393172	7764326	63	6	14	6	98	5.5	14.59	6	23.302	0	CSTA	High	High
-8467787264096670000	O00506	130084.9	290253.4	130084.9	157613.3	4	1	2	1	426	6.74	0	1	2.73	0.005	STK25	Peak Found	High
8342734995322820000	Q9UHQ4	892137.2	1979464	892137.2	1074887	13	3	5	3	241	9.54	2.43	3	13.129	0	BCAP29	High	High
7874915043186480000	P20962	5383441	11918565	5383441	6472013	23	2	7	2	102	4.16	2.8	2	10.202	0	PTMS	High	High
-8431092815676890000	Q8N0Y7	451275.7	998432.6	451275.7	542168.3	17	4	21	1	254	6.65	37.57	4	32.585	0	PGAM4	High	Peak Found
-232056303043	Q5RKV6	2048052	4521285	2048052	2455146	9	2	3	2	272	6.28	1.79	2	5.258	0	EXOSC6	High	High

7250000																		
5741463921085590000	O75962	252675.2	556888.7	252675.2	302401.4	1	2	2	2	3097	6.37	2.21	2	5.376	0	TRIO	High	High
4801012359298600000	Q6L8Q7	1663208	3652762	1663208	1983521	9	6	14	6	609	6.57	13.49	6	17.311	0	PDE12	High	High
5403198551536360000	Q14692	226198.8	496386.9	226198.8	269547.7	1	1	2	1	1282	6.44	0	1	2.596	0.006	BMS1	High	Peak Found
-8750059555873910000	Q96KB5	1200628	2622421	1200628	1424026	9	3	14	3	322	5.12	12.38	3	10.261	0	PBK	High	High
1457710463433020000	Q8N6M5	681556.8	1483043	681556.8	805321.3	3	1	3	1	391	6.16	3.65	1	3.484	0.002		High	High
-8624164927491720000	O75449	341432.3	740718.4	341432.3	402224.5	2	1	6	1	491	6.9	3.36	1	3.289	0.002	KATNA1	High	High
-418514708217	Q9ULD0	391300.3	844001.6	391300.3	458309.3	6	7	11	1	1010	6.65	6.97	7	17.038	0	OGDHL	High	High

0080000																		
3849836082424780000	Q14574	330594.9	708771.3	330594.9	384876.6	1	1	4	1	896	6.1	4.33	1	3.8	0.001	DSC3	High	High
654081652263858000	Q12905	38321833	82132207	38321833	44599385	39	13	67	13	390	5.26	103.72	13	86.216	0	ILF2	High	High
-4805951626840680000	P50336	576138.4	1230430	576138.4	668147.3	4	2	2	2	477	8.16	2.6	2	5.596	0	PPOX	Peak Found	High
4279350405355290000	Q9NWW5	179137.1	380139.4	179137.1	206423.1	4	1	1	1	311	8.87	0	1	2.207	0.009	CLN6	High	Peak Found
-2491250249564730000	P49902	77304077	1.62E+08	77304077	87983489	5	2	2	2	561	6.14	0	2	3.88	0	NT5C2	High	Peak Found
4548642001083050000	Q6UXV4	127638.7	267442.9	127638.7	145226.7	7	1	1	1	268	9.52	0	1	3.271	0.002	APOOL	Peak Found	High
2861301330543470000	Q8N766	14835574	30992641	14835574	16829607	16	14	26	14	993	7.66	24.36	14	43.671	0	EMC1	High	High

-4219070174713900000	P04049	6906606	14378818	6906606	7807978	3	2	4	2	648	9.2	1.7	2	6.195	0	RAF1	High	High
-3973589334991480000	P04264	2.31E+09	4.8E+09	2.31E+09	2.6E+09	65	54	455	46	644	8.12	819.19	54	432.204	0	KRT1	High	High
-7745810489150010000	O95292	484481.2	1000240	484481.2	543149.6	9	2	7	1	243	7.3	9.79	2	8.158	0	VAPB	High	High
365092219044477000	P30530	925929.9	1907906	925929.9	1036030	7	5	10	5	894	5.39	7.76	5	12.245	0		High	High
-4480157126328650000	O75937	13285295	27363134	13285295	14858714	27	7	13	7	253	9.06	11.8	7	23.899	0	DNAJC8	High	High
-466153880284673000	Q12873	316248.8	647677.7	316248.8	351701.6	3	6	11	1	2000	7.3	4.26	6	19.126	0	CHD3	High	Peak Found
8782400516531810000	Q16610	110336.8	224888.8	110336.8	122119	5	2	2	2	540	6.71	1.69	2	7.269	0	ECM1	High	High

-8649453304125060000	Q12906	78423724	1.6E+08	78423724	86661463	34	28	106	28	894	8.76	152.83	28	171.7	0	ILF3	High	High
6835034816184820000	Q13363	2672759	5436093	2672759	2951904	17	7	18	5	440	6.77	25.58	7	26.193	0	CTBP1	High	High
5389640593732990000	Q96FX8	378270.3	767400.6	378270.3	416713.5	4	1	1	1	193	7.03	2.04	1	2.212	0.009	PERP	High	Peak Found
-5619853924693830000	P23490	586681.4	1183569	586681.4	642701.2	3	1	6	1	312	8.09	5.61	1	2.737	0.005	LOR	High	High
-4574090656214790000	Q13765	58541938	1.18E+08	58541938	64032242	26	4	18	4	215	4.56	28.53	4	36.885	0	NACA	High	High
7989790464056540000	P35527	1.04E+09	2.09E+09	1.04E+09	1.13E+09	55	32	257	31	623	5.24	405.25	32	236.483	0	KRT9	High	High
-6948099861594560000	P31327	3866935	7657957	3866935	4158420	6	9	13	8	1500	6.74	14.35	9	26.538	0	CPS1	High	High

6326411103029320000	P53602	6873076	13552685	6873076	7359372	15	6	12	6	400	7.23	8.8	6	22.58	0	MVD	High	High
-7088025864314270000	O95793	6397200	12602970	6397200	6843658	24	11	21	10	577	9.44	31.2	11	46.824	0	STAU1	High	High
2825312433296890000	Q96HU8	9006189	17637438	9006189	9577472	6	1	2	1	199	8.76	4.82	1	3.591	0.001	DIRAS2	High	High
-2303647177069570000	Q6KB66	5127184	9966458	5127184	5411980	24	11	21	10	452	5.67	23.63	11	31.747	0	KRT80	High	High
4622690060897350000	Q9UII2	506660.4	973676	506660.4	528725	8	1	1	1	106	9.35	2	1	2.615	0.006	ATPIF1	Peak Found	High
-6469775095371740000	P01876	657970.5	1259050	657970.5	683688.5	5	2	2	2	353	6.51	0	2	3.64	0.001	IGHA1	High	High
3382628014078000000	O75891	682675.7	1306242	682675.7	709314.9	3	3	5	2	902	5.94	4.25	3	11.817	0	ALDH1L1	High	High

7082716862048420000	Q9BPX3	3503323	6690220	3503323	3632920	7	8	11	8	1015	5.59	9.43	8	20.731	0	NCAPG	High	High
6116188343313860000	Q6P1L8	372102.6	702752.6	372102.6	381608.3	17	2	3	2	145	10.24	1.61	2	5.44	0	MRPL14	High	High
6257643935121490000	Q15120	200954.5	378603.8	200954.5	205589.2	4	2	4	2	406	8.37	1.78	2	3.651	0.001	PDK3	High	High
350297391460659000	Q96CT7	428129.9	800101.8	428129.9	434470.8	8	2	3	2	223	9.54	4.71	2	6.638	0	CCDC124	High	Peak Found
-9155703229501670000	P31751	83137.13	155038.1	83137.13	84188.73	5	2	2	1	481	6.37	1.8	2	6.527	0	AKT2	High	Peak Found
-4019797043021120000	O60427	267248.6	497126.3	267248.6	269949.2	3	2	3	2	444	8.87	0	2	4.531	0	FADS1	High	High
-7283858502544500000	Q9NV31	500503.3	930726.5	500503.3	505402.6	8	1	4	1	184	9.5	5.98	1	4.327	0	IMP3	High	High

-3146791204946970000	Q9Y6W5	1540147	2811908	1540147	1526921	8	4	5	4	498	5.53	6.15	4	12.675	0	WASF2	High	High
-6656401588938300000	Q14676	1491395	2721762	1491395	1477970	2	3	4	3	2089	5.47	6.23	3	8.029	0	MDC1	High	High
-228772176721817000	Q96HY6	557884.9	1017408	557884.9	552472.2	2	1	3	1	314	5.12	3.7	1	2.071	0.01	DDRGK1	High	High
1824813434461880000	P84157	1042995	1894376	1042995	1028683	5	1	1	1	204	4.26	2.68	1	3.029	0.003	MXRA7	High	Peak Found
877541021914049000	Q53S08	143597	260408.1	143597	141406.7	15	3	4	1	254	6.74	4.86	3	11.147	0	WTH3DI; RAB6D	High	Peak Found
755217233260055000	O95786	1731177	3110943	1731177	1689302	6	6	9	6	925	6.4	3.62	6	14.278	0	DDX58	High	High
278040229627500000	Q9Y241	256522.8	460575.3	256522.8	250101.3	23	1	2	1	93	9.79	0	1	2.655	0.005	HIGD1A	High	High
-8779736381332900000	Q9UI42	418711.9	750249.3	418711.9	407399.9	2	1	3	1	421	6.7	4.02	1	3.577	0.001	CPA4	High	High

-2293599998867620000	Q8WVC6	266537.2	475293.7	266537.2	258093.7	4	1	1	1	231	9.58	1.98	1	2.266	0.009	DCAKD	High	Peak Found
-5130532040742560000	Q9HCN8	899239.1	1603282	899239.1	870613.4	13	3	3	3	221	7.03	0	3	5.762	0	SDF2L1	High	High
-3372054881013800000	Q9NVZ3	2081330	3694342	2081330	2006100	13	2	2	2	263	8.38	0	2	3.727	0.001	NECAP2	High	Peak Found
-4979023771098870000	Q9Y4P3	4665479	8271222	4665479	4491435	23	9	16	9	447	9.44	15.2	9	34.705	0	TBL2	High	High
-8623681790240610000	P07305	1052802	1864251	1052802	1012325	5	1	1	1	194	10.84	2.29	1	4.169	0	H1F0	Peak Found	High
7820543804174250000	P00374	268724.5	475218.7	268724.5	258053	5	1	2	1	187	7.42	0	1	4.018	0	DHFR; DHFRP1	High	High
350498965346	P51570	1853613	3222915	1853613	1750105	22	6	9	6	392	6.46	2.17	6	20.253	0	GALK1	High	High

1020000																		
-2089871811675690000	P26599	35295687	61256781	35295687	33263623	24	11	33	8	531	9.17	41.31	11	50.647	0	PTBP1	High	High
-895527807797415000	O14556	300522.9	518309.3	300522.9	281452	4	2	7	1	408	8.19	6.07	2	5.585	0	GAPDHS	Peak Found	High
7263541365096530000	Q13435	35879050	61666969	35879050	33486363	23	16	33	16	895	5.67	31.94	16	57.458	0	SF3B2	High	High
-6385363124040600000	Q13217	749967.9	1285750	749967.9	698187.4	7	3	4	3	504	6.15	2.03	3	10.123	0	DNAJC3	High	High
2183625062591560000	Q9NZT1	2707580	4595007	2707580	2495178	33	3	6	3	146	4.44	1.79	3	8.709	0	CALML5	High	High
-5991678768207070000	Q9UNF1	1328168	2249202	1328168	1221360	8	5	7	5	606	9.32	11.76	5	15.031	0	MAGED2	High	High
782407681045	P05165	47730990	80558142	47730990	43744637	41	28	75	28	728	7.52	126.43	28	156.154	0	PCCA	High	High

8490000																		
-3348700563280300000	Q8IWA4	2436602	4104422	2436602	2228781	5	3	5	2	741	6.33	2.55	3	8.697	0	MFN1	High	High
8344699724709940000	O00763	12133069	20435459	12133069	11096851	21	46	175	30	2458	6.49	270.98	46	226.422	0	ACACB	High	High
-6246281522193440000	O60573	164825.2	277197.7	164825.2	150523.7	4	1	2	1	245	8.88	3.57	1	3.33	0.002	EIF4E2	High	High
-3203916588114810000	P02452	5610945	9390147	5610945	5099032	8	10	20	10	1464	5.8	28.64	10	31.617	0	COL1A1	High	High
-8019113709048030000	Q9NPE3	278688.1	465671.3	278688.1	252868.6	14	1	2	1	64	9.99	0	1	3.124	0.003	NOP10	High	High
770343860011856000	Q96P63	1332917	2225873	1332917	1208692	4	2	3	2	405	5.53	4.09	2	5.529	0	SERPINB12	High	High
362946439677958000	Q9Y679	789613.7	1316563	789613.7	714919	8	3	3	3	410	8.65	1.89	3	8.319	0		High	High

-404480449398085000	P51809	2591759	4292406	2591759	2330860	16	4	6	4	220	8.6	1.64	4	10.698	0	VAMP7	High	Peak Found
5123694492544730000	Q99417	1783195	2945487	1783195	1599457	20	2	5	2	103	5.91	6.24	2	6.019	0	MYCBP	High	High
7198883007019980000	Q8N1N4	3037641	5014497	3037641	2722970	18	11	23	8	520	6.02	33.55	11	31.211	0	KRT78	High	High
7643769137675680000	P82933	336864.9	554065	336864.9	300868.1	9	3	3	3	396	9.51	3.1	3	13.156	0	MRPS9	High	High
-5442712666023180000	O43251	1828796	3006141	1828796	1632393	15	4	6	4	390	7.27	13.22	4	18.783	0	RBFOX2	High	High
8849242953917420000	Q9HCD5	417620.8	685082.1	417620.8	372012.9	5	2	2	2	579	9.6	0	2	5.816	0	NCOA5	High	Peak Found
-7057642348640180000	P02768	51371508	84221813	51371508	45734082	42	27	81	27	609	6.28	115.5	27	129.533	0	ALB	High	High

-3265471816786770000	Q9Y657	415325.3	680882.5	415325.3	369732.4	4	1	1	1	262	6.96	1.64	1	3.293	0.002	SPIN1	High	Peak Found
-7614140924344960000	P14136	415325.3	680882.5	415325.3	369732.4	6	3	4	1	432	5.52	5.01	3	7.915	0	GFAP	Peak Found	High
276333743643228000	O95870	364770	597748.6	364770	324589.1	3	2	3	2	558	8.13	3.52	2	5.969	0	ABHD16A	High	High
-2523415922773780000	Q9Y3C1	263699	430968.8	263699	234024.4	8	1	1	1	178	9.94	0	1	3.552	0.002	NOP16	High	Peak Found
6639457363078970000	P35659	8222859	13436003	8222859	7296011	20	9	22	9	375	8.56	21.37	9	36.573	0	DEK	High	High
-9086652691068790000	P51970	182726.4	297488.8	182726.4	161542.2	4	1	2	1	172	7.65	1.78	1	2.545	0.006	NDUFA8	High	High
-7809965917378940000	P42892	10556175	17185691	10556175	9332164	15	9	18	9	770	5.88	18.68	9	37.226	0	ECE1	High	High

-1072858414751410000	P56589	192769.5	310397.9	192769.5	168552.1	3	1	1	1	373	8.15	1.78	1	4.015	0	PEX3	High	Peak Found
-5758489394402470000	Q8WWZ4	3855855	6182501	3855855	3357219	1	1	2	1	1543	6.65	4.19	1	2.675	0.005	ABCA10	Peak Found	High
3327789120096560000	Q9NR56	4083102	6546519	4083102	3554887	18	6	10	6	388	8.9	9.3	6	18.428	0	MBNL1	High	High
3549591940651770000	P17252	2856224	4574765	2856224	2484186	10	6	14	5	672	7.05	3.92	6	17.546	0	PRKCA	High	High
29627463948576900	Q9NUQ8	2288239	3649786	2288239	1981905	7	5	8	5	709	6.34	7.69	5	15.656	0	ABCF3	High	High
6208634135307330000	P17480	1356609	2162036	1356609	1174028	7	5	8	5	764	5.81	10.61	5	16.82	0	UBTF	High	High
-1295791422585610000	Q99720	2292253	3650179	2292253	1982118	9	2	5	2	223	5.96	10.41	2	15.407	0	SIGMAR1	High	High

2121660018572640000	Q9Y6I9	488218.8	774079.5	488218.8	420340.2	8	2	6	2	313	4.86	5.52	2	7.485	0	TEX264	High	High
-7081455264055240000	Q16658	1.53E+08	2.41E+08	1.53E+08	1.31E+08	38	17	56	17	493	7.24	82.64	17	93.165	0	FSCN1	High	High
-5077624206486070000	Q8TC07	1102356	1737179	1102356	943321.9	6	4	6	4	691	5.67	1.7	4	9.62	0	TBC1D15	High	High
-2098807042560150000	P13489	84971438	1.34E+08	84971438	72649955	48	18	64	18	461	4.82	124.36	18	110.92	0	RNH1	High	High
8020879242086100000	Q53GA4	1012887	1594237	1012887	865701.7	6	1	2	1	152	9.17	2.26	1	3.683	0.001	PHLDA2	High	High
2730798192549930000	P08123	7904827	12432871	7904827	6751291	8	10	22	10	1366	8.95	17.9	10	31.919	0	COL1A2	High	High
5207215076680510000	Q9H300	365283.1	573680.7	365283.1	311519.8	2	1	1	1	379	9.79	1.76	1	3.033	0.003	PARL	High	Peak Found

-1249577678687790000	Q9HD20	2952088	4634263	2952088	2516495	2	3	4	3	1204	8.13	1.79	3	4.865	0	ATP13A1	High	High
-2325443013124670000	Q6NXE6	206539.9	323751.8	206539.9	175803.5	2	1	1	1	501	6.24	1.93	1	3.553	0.002	ARMC6	High	Peak Found
-6182568076537300000	P35030	3842338	6011823	3842338	3264537	4	1	2	1	304	7.49	4.66	1	6.904	0	PRSS3	High	Peak Found
500586164942944000	P67809	24533949	38355943	24533949	20828023	44	11	35	6	324	9.88	74.18	11	95.625	0	YBX1	High	High
-8565367477804830000	Q6IPM2	54327.95	84559.35	54327.95	45917.37	2	1	1	1	695	9.07	0	1	2.224	0.009	IQCE	High	Peak Found
-7216673517695950000	Q8TDZ2	196412.9	304576.8	196412.9	165391.1	2	2	3	2	1067	6.4	2.11	2	7.685	0	MICAL1	High	High
2983297722228310000	P28370	547450.8	845596.6	547450.8	459175.4	3	3	6	1	1054	8.09	9.72	3	14.492	0	SMARCA1	High	Peak Found

7610728054593450000	Q86SZ2	454209.6	701043.5	454209.6	380680.3	5	1	2	1	158	8.68	0	1	2.535	0.006	TRAPPC6B	High	High
-3953719107085220000	Q9Y6K9	581831.5	896827.8	581831.5	486995	6	2	3	2	419	5.71	3.94	2	5.484	0	IKBKG	High	High
6449078799256650000	Q53FA7	2564238	3944780	2564238	2142092	7	2	2	2	332	7.17	0	2	5.254	0	TP53I3	High	Peak Found
-8814237146634600000	Q15642	2646522	4057142	2646522	2203107	14	6	10	6	601	5.73	5.78	6	24.564	0	TRIP10	High	High
-8770420070751580000	Q16718	323516.4	495807.6	323516.4	269233.2	9	1	1	1	116	5.99	0	1	3.144	0.003	NDUFA5	Peak Found	High
-8678565654600360000	Q8WWV3	484622.9	742001.5	484622.9	402921.3	2	1	1	1	396	9.11	0	1	3.287	0.002	RTN4IP1	High	Peak Found
-162580331296	Q15269	590544.8	902671.3	590544.8	490168.1	2	2	2	2	919	6.15	0	2	4.148	0	PWP2; LOC102724159	High	High

2440000																		
-1917909410339360000	Q96QC0	574378.3	876777.5	574378.3	476107.2	1	1	2	1	940	9.17	0	1	2.14	0.01	PPP1R10	High	Peak Found
-3405025144750980000	P52732	317541.4	484369.5	317541.4	263022.1	5	4	7	4	1056	5.64	7.88	4	12.05	0	KIF11	High	High
7534759113121090000	Q9Y3D9	1498566	2277008	1498566	1236460	16	3	7	3	190	8.9	12.01	3	10.413	0	MRPS23	High	High
5604929834230100000	P53794	681603.5	1033430	681603.5	561172.7	2	1	1	1	718	7.27	0	1	2.658	0.005	SLC5A3	High	Peak Found
-8824926395777540000	Q96MT3	2015692	3050169	2015692	1656301	1	1	1	1	831	6.24	1.63	1	2.403	0.008	PRICKLE1	High	Peak Found
8552270873577270000	Q8IWA5	192796.4	290927.5	192796.4	157979.3	3	2	3	2	706	8.57	0	2	4.867	0	SLC44A2	High	High
539692156188	Q96GD0	1342061	2019147	1342061	1096436	15	3	6	3	296	6.55	6.21	3	9.912	0	PDXP	High	High

3890000																		
5949671281598120000	Q13464	608431.2	914174.7	608431.2	496414.6	3	5	11	2	1354	5.9	10.92	5	13.055	0	ROCK1	High	High
6827826663467100000	Q9UK41	648775.4	974465.5	648775.4	529153.7	7	2	2	2	221	5.54	0	2	3.079	0.003	VPS28	High	Peak Found
6201262043542300000	P34896	2615132	3924483	2615132	2131071	11	4	9	3	483	7.71	9.81	4	15.995	0	SHMT1	High	High
-2064458318068970000	Q96QK1	20355021	30545849	20355021	16586990	11	8	21	8	796	5.49	32.06	8	41.413	0	VPS35	High	High
6501697818920190000	Q9Y3B9	477044.9	713140.8	477044.9	387249.3	9	2	2	2	282	5.52	2.44	2	8.013	0	RRP15	High	High
469142345215753000	Q9Y2R9	459028.4	685060.4	459028.4	372001.1	10	2	5	2	242	9.99	2.41	2	6.451	0	MRPS7	High	High
-2887047081584330000	P54652	1379025	2053916	1379025	1115316	21	14	58	3	639	5.74	119.49	14	88.913	0	HSPA2	High	Peak Found

2945964816553270000	Q9Y2X0	200882.6	298053	200882.6	161848.5	1	1	2	1	877	7.37	1.67	1	2.511	0.006	MED16	High	Peak Found
-7879323310516700000	Q14517	136233.8	201818.8	136233.8	109591.5	0	1	2	1	4588	5	2.31	1	4.561	0	FAT1	High	High
-1376480877538070000	Q14232	606239.1	897517.5	606239.1	487369.5	8	2	3	2	305	7.33	0	2	7.934	0	EIF2B1	High	High
8682459872602530000	Q8N108	419339	620810.2	419339	337112	4	2	3	2	512	4.42	0	2	6.736	0	MIER1	High	High
3627252899551950000	Q9NSC5	407412.2	602431.4	407412.2	327132	11	4	6	4	361	5.45	0	4	8.043	0	HOMER3	High	High
-792882720935006000	Q9HAV4	2907925	4279214	2907925	2323696	6	6	12	6	1204	5.8	5.72	6	17.296	0	XPO5	High	High
8454366493935610000	P19404	549034	806998	549034	438215.6	5	1	2	1	249	8.06	0	1	2.625	0.005	NDUFV2	High	High

2507883408374060000	O14874	263740.6	385487.8	263740.6	209327.4	2	1	4	1	412	8.82	0	1	3.228	0.002	BCKDK	High	High
-5712517081782200000	Q86V48	731476.6	1062785	731476.6	577112.9	2	2	3	2	1076	8.5	3.76	2	4.693	0	LUZP1	High	High
-8085181882771770000	Q9Y6M1	3063067	4449645	3063067	2416244	19	8	13	8	599	8.46	5.98	8	26.669	0	IGF2BP2	High	High
2064522308959820000	Q92841	24069295	34908552	24069295	18956022	32	21	64	15	729	8.27	99.04	21	90.546	0	DDX17	High	High
-6993927886623030000	P31947	6927284	10037907	6927284	5450779	48	14	61	8	248	4.74	91.77	14	62.734	0	SFN	High	High
-8590591323131710000	Q86UL3	421929.9	610582.3	421929.9	331558.1	3	1	1	1	456	9.19	0	1	2.943	0.004	AGPAT6; GPAT4	High	Peak Found
-379276756923	P49411	75989347	1.1E+08	75989347	59604193	46	18	66	18	452	7.61	137.96	18	122.168	0	TUFM	High	High

8330000																		
6174880606405180000	P56545	9705599	13994691	9705599	7599389	21	8	19	6	445	6.95	20.38	8	25.311	0	CTBP2	High	High
3553931004733420000	P62306	712933.8	1025420	712933.8	556822.9	9	1	1	1	86	4.67	0	1	2.479	0.007	SNRPF	Peak Found	High
1964393919908820000	Q96I99	19374639	27854148	19374639	15125344	37	15	33	15	432	6.39	42.36	15	63.406	0	SUCLG2	High	High
-9099132526023130000	Q9Y6D5	1506330	2165512	1506330	1175915	4	6	9	2	1785	6.33	6.18	6	19.054	0	ARFGEF2	High	Peak Found
-5506725299494210000	P25786	45716590	65621246	45716590	35633612	52	12	40	12	263	6.61	31.19	12	59.133	0	PSMA1	High	High
-1496446301178740000	Q9UHJ6	220282	315831.8	220282	171502.8	3	1	1	1	478	6.83	0	1	3.881	0	SHPK	High	Peak Found
-191174189199	Q9NX14	1137977	1631543	1137977	885959.6	7	1	3	1	153	5.22	5.61	1	3.767	0.001	NDUFB11	High	High

1950000																		
668756523857915000	Q8TB61	2238371	3203642	2238371	1739640	8	3	4	3	432	9.16	5.6	3	9.578	0	SLC35B2	High	High
1319936172901890000	P45973	574469.3	821862.3	574469.3	446287.2	4	1	1	1	191	5.86	0	1	2.495	0.007	CBX5	High	Peak Found
1400550207963650000	Q8WWY3	2318684	3314300	2318684	1799730	7	3	4	3	499	5.78	4.22	3	9.553	0	PRPF31	High	High
7915311945115270000	O15212	1654174	2363805	1654174	1283592	21	3	6	3	129	8.88	3.68	3	7.83	0	PFDN6	High	High
-7577661902964430000	Q3LXA3	913799.8	1303888	913799.8	708036.3	11	4	6	4	575	7.49	5.03	4	15.42	0	DAK; TKFC	High	High
197200824509217000	P31040	12470642	17747425	12470642	9637197	16	9	21	9	664	7.39	23.56	9	42.653	0	SDHA	High	High
-5786559644591860000	Q9BSF4	1534275	2182276	1534275	1185018	7	2	3	2	260	8.09	3.6	2	4.205	0	C19orf52; TIMM29	High	Peak Found

-92542688516821200	Q14699	3470203	4932970	3470203	2678698	16	6	13	6	578	5.67	14.14	6	27.411	0	RFTN1	High	High
3789194265575880000	Q96RQ1	1054780	1495531	1054780	812102.4	6	2	6	2	377	6.77	6.2	2	11.543	0	ERGIC2	High	High
2458252490920420000	P16402	62472407	88568834	62472407	48094599	21	9	30	9	221	11.02	62.71	9	48.373	0	HIST1H1D	High	High
3945672141617060000	Q9P2E9	1.44E+08	2.04E+08	1.44E+08	1.11E+08	42	52	125	52	1410	8.6	205.56	52	260.341	0		High	High
5638304543600080	Q9NZJ7	872397	1232254	872397	669137.7	3	1	2	1	389	9.32	0	1	2.37	0.008	MTCH1	High	Peak Found
-8113893803591480000	Q7Z794	67351077	95113793	67351077	51648639	26	17	40	12	578	5.99	51.92	17	69.66	0	KRT77	High	High
-1875053903051500000	Q5ZPR3	1018043	1436515	1018043	780055.6	2	1	8	1	534	4.91	1.65	1	4.758	0	CD276	High	High
622969922407	Q13107	537707.9	757322.7	537707.9	411241	4	4	5	3	963	5.71	3.61	4	11.517	0	USP4	High	High

0920000																		
-6685402705848510000	Q92820	16074581	22638876	16074581	12293350	34	10	24	10	318	7.11	21.5	10	35.889	0	GGH	High	High
5638474282161260000	Q969Z0	2898187	4078835	2898187	2214887	10	5	13	5	631	7.42	9.72	5	20.071	0	TBRG4	High	High
9124965961007810000	P11387	4085254	5727905	4085254	3110364	10	8	15	8	765	9.31	9.74	8	23.101	0	TOP1	High	High
8995657341512930000	P47914	950156.1	1330576	950156.1	722528.9	9	1	2	1	159	11.66	5.25	1	6.832	0	RPL29	High	High
-9120451514636770000	Q12979	736477.6	1031112	736477.6	559914.1	3	2	3	2	859	6.55	1.78	2	4.504	0	ABR	High	High
4128416838288870000	P11177	6469867	9056156	6469867	4917669	23	7	15	7	359	6.65	16.39	7	31.244	0	PDHB	High	High
-462940392749	P62987	522675.1	730794	522675.1	396835.4	64	10	39	1	128	9.83	45.33	10	47.969	0	UBA52	Peak Found	High

6450000																		
-5628662921146720000	Q9UL18	241882.8	337807.4	241882.8	183436	5	4	6	2	857	9.16	0	4	10.905	0	AGO1; EIF2C1	High	Peak Found
-4387362286314670000	Q14318	798240.9	1114430	798240.9	605157	9	3	6	3	412	4.84	0	3	9.001	0	FKBP8	High	High
-1018884242771770000	P30048	17278500	24107086	17278500	13090616	37	8	23	8	256	7.78	27.86	8	42.524	0	PRDX3	High	High
-1776648160380260000	Q10472	1574127	2193300	1574127	1191004	8	4	5	4	559	7.72	0	4	8.872	0	GALNT1	High	High
2772167970486260000	Q6ZXV5	780302.6	1087004	780302.6	590264.3	4	4	7	3	915	8.87	1.65	4	9.657	0	TMTC3	High	High
5313402656067690000	Q8IY17	3750678	5215707	3750678	2832230	7	7	11	7	1375	7.74	14.77	7	28.955	0		High	High

7059158695698350000	Q9H0U4	11436656	15901977	11436656	8635083	38	7	28	2	201	5.73	37.37	7	37.259	0	RAB1B	High	High
882715983706929000	Q9NZ08	2893175	4018556	2893175	2182154	9	7	13	7	941	6.46	15.75	7	21.164	0	ERAP1	High	High
682087624881918000	O00400	249967.3	347109.8	249967.3	188487.3	4	2	3	2	549	7.33	5.5	2	9.29	0	SLC33A1	High	High
1601999827614050000	O95067	447598.8	621529.6	447598.8	337502.7	3	1	1	1	398	8.9	0	1	2.735	0.005	CCNB2	High	Peak Found
-8265724560765900000	Q9BVP2	3526062	4891207	3526062	2656020	11	6	9	6	549	9.16	8.87	6	16.583	0	GNL3	High	High
385044699666679000	P49756	3198667	4434400	3198667	2407965	7	5	6	5	843	6.32	8.11	5	16.722	0	RBM25	High	High
-692133851352470000	Q14376	800027.5	1107549	800027.5	601420.8	6	2	5	2	348	6.73	1.67	2	5.932	0	GALE	High	High
5620405547330280000	Q7Z4V5	986770.5	1364451	986770.5	740923.6	6	4	6	3	671	7.49	7.15	4	13.211	0	HDGFRP2; HDGFL2	High	High

3677224009341930000	P48729	2009293	2777445	2009293	1508207	15	5	7	5	337	9.57	5.82	5	13.328	0	CSNK1A1	High	High
3056061449621500000	Q3SXM5	1283255	1771875	1283255	962162.8	5	2	5	2	330	8.72	0	2	5.889	0	HSDL1	High	High
5384337703464320000	Q16822	5087499	7023551	5087499	3813925	22	11	18	11	640	7.62	14.58	11	31.631	0		High	High
-8682712823667690000	Q7Z2E3	398364.2	549723.8	398364.2	298510.7	3	1	1	1	356	9.17	0	1	2.272	0.009	APTX	High	Peak Found
-2320605404870650000	Q8NC51	17386780	23977353	17386780	13020169	26	11	28	11	408	8.65	25.43	11	45.343	0	SERBP1	High	High
-9085174644749990000	Q4G0F5	543915.5	750019	543915.5	407274.9	6	2	2	2	336	7.36	2.22	2	6.201	0	VPS26B	High	Peak Found
-750359377190588000	O75616	169990.5	234050.7	169990.5	127094.1	4	1	1	1	437	8.84	0	1	2.654	0.005	ERAL1	Peak Found	High

8789508812130390000	Q9BTE7	595313.3	816122.1	595313.3	443170.2	4	1	2	1	237	5.58	0	1	3.015	0.003	DCUN1D5	High	Peak Found
-9023390433505770000	P53597	7756795	10629969	7756795	5772280	14	5	10	5	346	8.79	11.23	5	18.007	0	SUCLG1	High	High
-6548420804533650000	O43353	662271.4	905118.5	662271.4	491497	3	2	3	1	540	7.09	0	2	4.909	0	RIPK2	High	Peak Found
-296072814185556000	Q9BUI4	254092.4	346691.3	254092.4	188260.1	2	1	3	1	534	7.31	2.12	1	4.932	0	POLR3C; LOC101060460	High	High
-8489357358395880000	Q86SX6	521668.8	711726.6	521668.8	386481.4	14	2	2	2	157	6.79	2.63	2	9.003	0	GLRX5	High	High
-8984223167418130000	P62633	1723240	2348452	1723240	1275255	18	3	5	3	177	7.71	4.36	3	12.371	0	CNBP	High	High
-241471088453	Q96G21	230129.5	312929.6	230129.5	169926.9	3	1	2	1	291	9.47	3.75	1	3.878	0	IMP4	High	High

5390000																		
-4363739394428500000	O75330	4840478	6545789	4840478	3554491	1	1	1	1	724	5.83	0	1	2.379	0.008	HMMR	High	Peak Found
1074940222938940000	Q9NWM8	309564.4	417915.5	309564.4	226936.3	5	1	2	1	211	6.07	0	1	2.402	0.008	FKBP14	High	High
6343350754074720000	P83881	3650413	4923073	3650413	2673325	18	3	9	2	106	10.58	14.62	3	10.027	0	RPL36A	High	High
-7883409800749110000	Q7KZ85	1762806	2376885	1762806	1290695	2	2	2	2	1726	4.91	2.31	2	6.616	0	SUPT6H	High	Peak Found
-309007205683403000	Q8IWS0	814584.5	1097515	814584.5	595972.1	5	2	3	2	365	8.68	1.69	2	4.774	0	PHF6	High	High
-3647581331953330000	Q658Y4	2458939	3310102	2458939	1797450	3	3	4	3	838	6.39	3.84	3	5.97	0	FAM91A1	High	High
868363632382073000	Q6UB35	3126644	4208753	3126644	2285435	8	7	12	7	978	8.06	7.21	7	19.387	0	MTHFD1L	High	High

-3364177795992670000	P42765	64187145	86365873	64187145	46898348	34	13	46	13	397	8.09	77.54	13	71.53	0	ACAA2	High	High
-1882334441137320000	Q9BUJ2	15256958	20503700	15256958	11133908	20	16	40	15	856	6.92	53.34	16	87.264	0	HNRNPUL1	High	High
2009664634068220000	P15151	3650932	4903960	3650932	2662945	13	4	10	4	417	6.52	10.44	4	25.208	0	PVR	High	High
7054273267636820000	Q08211	72741545	97333781	72741545	52854135	27	33	94	33	1270	6.84	142.53	33	154.763	0	DHX9	High	High
1875589299266390000	Q14139	1810749	2422289	1810749	1315350	4	4	7	4	1066	5.24	6.1	4	12.126	0	UBE4A	High	High
-2723380110301130000	Q9UKS6	790980.7	1057460	790980.7	574221.4	6	2	3	2	424	6.18	3.84	2	6.842	0	PACSIN3	High	High
875070389354990000	O00273	1493177	1992290	1493177	1081852	14	4	10	4	331	4.79	3.88	4	11.538	0	DFFA	High	High

9158883592383280000	P60983	8893386	11860248	8893386	6440345	14	2	4	2	142	5.29	2.57	2	7.777	0	GMFB	High	High
-1581896134396950000	P49006	499516.5	665873.1	499516.5	361582	7	1	2	1	195	4.67	4.44	1	5.818	0	MARCKSL1	High	High
-2471884395781020000	Q9Y5X2	169873.5	226378.3	169873.5	122927.8	3	1	2	1	465	7.39	0	1	3.188	0.003	SNX8	High	High
-8372926252788990000	Q5JTZ9	3329152	4433897	3329152	2407693	13	11	20	11	985	6.27	23.11	11	37.89	0	AARS2	High	High
-547053484993874000	Q13085	4.5E+09	5.98E+09	4.5E+09	3.25E+09	56	140	995	124	2346	6.37	1893.99	140	1076.898	0	ACACA	High	High
-7043086942307680000	P63220	10940311	14547913	10940311	7899800	35	4	18	4	83	8.5	27.84	4	35.414	0	RPS21	High	High
6734575471453550000	Q6FI81	919887.1	1222844	919887.1	664028	10	2	4	2	312	5.62	0	2	8.443	0	CIAPIN1	High	High

-5155365173843630000	P48507	3589871	4766185	3589871	2588131	18	4	7	4	274	6.02	4.37	4	13.377	0	GCLM	High	High
-877235499680530000	Q15366	28862381	38289935	28862381	20792179	36	9	28	4	365	6.79	43.75	9	39.588	0	PCBP2	High	High
1099504193891770000	Q13557	9293384	12320819	9293384	6690444	12	6	14	5	499	7.25	11.86	6	23.619	0	CAMK2D	High	High
-3390977729729590000	P14735	3247170	4297403	3247170	2333574	5	6	15	6	1019	6.61	10.3	6	20.227	0	IDE	High	High
4995035234308880000	Q9Y6B6	2863241	3785661	2863241	2055688	28	7	23	3	198	6.11	24.16	7	31.584	0	SAR1B	High	High
-3780861066822230000	Q9H788	624590.1	825515.3	624590.1	448270.9	3	1	1	1	454	8.06	1.88	1	3.961	0	SH2D4A	High	Peak Found
-6898362588139570000	Q01105	9022973	11920773	9022973	6473212	19	5	9	5	290	4.32	12.36	5	21.828	0	SET	High	High

155133754937223000	O43402	832807.3	1099150	832807.3	596859.6	4	1	3	1	210	6.4	1.82	1	2.576	0.006	EMC8	High	High
5468856902780970000	O60884	6351398	8370751	6351398	4545481	10	5	9	5	412	6.48	12.21	5	17.463	0	DNAJA2	High	High
5446146544739150000	Q15654	999277.6	1316810	999277.6	715053.6	6	2	2	2	476	7.37	0	2	4.098	0	TRIP6	High	Peak Found
2085339884306230000	Q13501	409027.6	538298.1	409027.6	292306.3	5	1	2	1	440	5.22	3.89	1	7.299	0	SQSTM1	High	High
-4697370261215880000	O43583	10052880	13226616	10052880	7182310	21	4	6	4	198	5.3	5.83	4	8.386	0	DENR	High	High
6495840506053030000	Q9NRV9	977953.3	1282799	977953.3	696584.9	11	2	2	2	189	5.8	2.06	2	4.949	0	HEBP1	High	High
5422158606125750000	Q86U42	1833934	2405220	1833934	1306081	6	2	3	2	306	5.06	4.48	2	7.295	0	PABPN1	High	High
-576204318165	P46109	1532484	2009569	1532484	1091235	16	3	4	3	303	6.74	5.23	3	6.697	0	CRKL	High	High

0020000																		
-7309351128836490000	P26583	4179723	5478723	4179723	2975053	34	9	19	6	209	7.81	25.32	9	29.998	0	HMGB2	High	High
-5823057819559300000	O15230	257605.2	337410.7	257605.2	183220.6	1	3	3	3	3695	7.02	1.64	3	6.23	0	LAMA5	High	Peak Found
-8148433569690540000	Q96C36	4691425	6142994	4691425	3335765	20	6	26	5	320	7.77	29.13	6	21.982	0	PYCR2	High	High
-4738177424081240000	Q8WWI1	2121945	2776691	2121945	1507797	3	4	7	4	1683	8.09	12.29	4	16.301	0	LMO7	High	High
-901453278608650000	Q05682	12923845	16901292	12923845	9177730	18	13	26	13	793	5.66	33.21	13	44.294	0	CALD1	High	High
-7926875629278280000	Q9UKV5	489425.8	639719	489425.8	347379.9	3	2	4	2	643	6.39	1.78	2	6.619	0	AMFR	High	High

-7071014728097240000	Q9BW27	1225789	1601048	1225789	869400.1	3	2	3	2	656	5.55	4.58	2	5.95	0	NUP85	High	High
-6843403857140780000	Q8NBL1	1360425	1775891	1360425	964343.1	7	3	7	3	392	8.72	3.92	3	9.369	0	POGLUT1	High	High
1039714562309970000	O14735	854359.6	1113487	854359.6	604645.2	10	2	4	2	213	8.03	0	2	3.87	0	CDIPT	High	High
4873460257973370000	Q7L0J3	526857.7	685393.8	526857.7	372182.1	3	2	3	2	742	5.57	3.61	2	7.095	0	SV2A	High	High
4722809731006210000	P48047	20836912	27086707	20836912	14708609	44	8	22	8	213	9.96	36.31	8	33.603	0	ATP5O	High	High
-5532813342924820000	Q9Y383	5871117	7630365	5871117	4143437	16	6	13	6	392	10.01	3.99	6	19.006	0	LUC7L2	High	High
-7918100123629270000	Q9NWV4	1745447	2265971	1745447	1230466	30	3	4	3	160	5.01	0	3	7.209	0	C1orf123	High	High

3678214923131320000	Q96RQ3	25869337	33472518	25869337	18176228	31	18	51	18	725	7.78	90.94	18	98.377	0	MCCC1	High	High
-2849777412656730000	Q13190	1471296	1900033	1471296	1031755	5	2	5	2	355	9.16	9.85	2	6.843	0	STX5	High	High
-154520297388586000	Q9HCC0	9168055	11836236	9168055	6427306	28	12	29	12	563	7.68	38.7	12	57.596	0	MCCC2	High	High
6590851508053800000	Q9BQ52	4114474	5299313	4114474	2877630	13	9	16	9	826	7.9	13.52	9	27.398	0	ELAC2	High	High
8650097818779680000	P62857	9740314	12533655	9740314	6806018	46	3	19	3	69	10.7	20.47	3	15.613	0	RPS28	High	High
3819928772784180000	Q03001	4136938	5316218	4136938	2886810	2	11	13	11	7570	5.25	7.47	11	24.595	0	DST	High	High
688156256911313000	Q5T0N5	907939	1165410	907939	632840.5	3	2	2	2	605	6.64	2.08	2	6.36	0	FNBP1L	High	Peak Found
6084836262507510000	Q6ZUV0	5566614	7134608	5566614	3874231	8	2	2	1	252	7.15	2.24	2	5.096	0	LOC344967	High	Peak Found

-2260804329009370000	Q13045	41972413	53750207	41972413	29187407	13	17	34	17	1269	6.05	32.26	17	58.424	0	FLII	High	High
-4655855300014540000	Q06330	1043555	1336360	1043555	725669.2	4	2	3	2	500	7.18	4.58	2	8.255	0	RBPJ	High	High
7260357318160890000	O75439	2917471	3733120	2917471	2027157	13	6	11	6	489	6.83	15.1	6	18.553	0	PMPCB	High	High
2391996285989380000	P35625	2556344	3267633	2556344	1774388	19	4	5	4	211	8.72	6.67	4	10.172	0	TIMP3	High	High
-973328225883047000	Q8IV48	639682.3	817657.5	639682.3	444003.9	2	1	2	1	349	6.7	1.67	1	2.468	0.007	ERI1	High	High
3200179838073860000	O76031	3883335	4961723	3883335	2694312	11	6	10	6	633	7.58	8.22	6	21.424	0	CLPX	High	High
6674387397432110000	P68371	35518664	45337037	35518664	24618893	56	24	252	3	445	4.89	435.56	24	162.608	0	TUBB4B	High	High
341483115598	P06703	1.32E+08	1.69E+08	1.32E+08	91760396	32	5	18	5	90	5.48	19.57	5	19.416	0	S100A6	High	High

1600000																		
6670005547915150000	O15400	405742.5	517653.9	405742.5	281096.2	5	1	1	1	261	5.55	0	1	3.511	0.002	STX7	High	Peak Found
1728322554936910000	Q14978	757051.5	965677.5	757051.5	524381.7	3	2	5	2	699	9.47	3.66	2	5.367	0	NOLC1	High	High
-2599348248423270000	P23258	1744559	2224394	1744559	1207889	12	4	10	4	451	6.14	8.09	4	10.916	0	TUBG1	High	High
-6424990256346310000	P04792	76082658	96979636	76082658	52661828	65	14	57	14	205	6.4	106.45	14	100.803	0	HSPB1	High	High
-4729149100999830000	Q9NT62	1291757	1646325	1291757	893986.6	11	3	5	3	314	4.74	7.64	3	13.177	0	ATG3	High	High
-2254798804738060000	O60271	4804971	6112408	4804971	3319156	10	12	15	12	1321	5.15	5.2	12	28.737	0	SPAG9	High	High

-6149624510723030000	P30043	9685585	12314440	9685585	6686980	44	6	11	6	206	7.65	9.12	6	31.433	0	BLVRB	High	High
8957423501143170000	P68366	18881993	23991972	18881993	13028107	42	18	131	5	448	5.06	224.48	18	129.47	0	TUBA4A	High	High
-3328900627537440000	Q03111	317715	403533.4	317715	219126.5	2	1	1	1	559	8.59	1.76	1	3.478	0.002	MLLT1	High	Peak Found
-1141422113329580000	Q04446	33963478	43076033	33963478	23391124	26	16	44	16	702	6.32	95.5	16	103.152	0	GBE1	High	High
-3585481793110580000	Q7Z7M9	516172	653738.6	516172	354992.8	1	1	1	1	940	9.47	0	1	2.688	0.005	GALNT5	Peak Found	High
-8547587141624530000	Q9UKV8	1192170	1508246	1192170	819007.1	6	5	8	3	859	9.19	1.6	5	14.575	0	AGO2; EIF2C2	High	Peak Found
-655322416209	Q9Y3A3	807816.6	1021389	807816.6	554634.1	4	1	2	1	225	5.78	0	1	2.533	0.006	MOB4	High	High

0550000																		
-658594625945977000	O00139	11142293	14073140	11142293	7641989	14	10	19	9	706	6.68	22.7	10	29.39	0	KIF2A	High	High
8333462526308280000	O60671	1568462	1980455	1568462	1075426	6	2	5	2	282	4.83	1.78	2	3.628	0.001	RAD1	High	High
-4835883825689200000	Q92979	973435.1	1228713	973435.1	667215.1	10	2	3	2	244	9.17	3.22	2	6.632	0	EMG1	High	High
-6888861531159930000	Q9H832	3345257	4204723	3345257	2283247	12	4	5	4	354	5.62	1.63	4	12.403	0	UBE2Z	High	High
1367538517643270000	Q13724	10762965	13520647	10762965	7341974	14	10	25	10	837	8.9	27.13	10	34.782	0	MOGS	High	High
6108137657648570000	O95571	1828026	2295888	1828026	1246712	11	2	6	2	254	6.83	7.29	2	11.293	0	ETHE1	High	High
-650713592391	Q9BSC4	142743	179263.6	142743	97343.6	2	1	3	1	688	8.46	0	1	2.575	0.006	NOL10	High	High

9710000																		
-3931502688338010000	P35249	7501376	9407456	7501376	5108431	21	8	16	8	363	8.02	18.12	8	25.296	0	RFC4	High	High
-5021864284547300000	Q96GD4	437125.7	547903.6	437125.7	297522.3	10	3	4	3	344	9.29	0	3	7.55	0	AURKB	High	High
-7761663828976320000	Q9NXE4	616539.6	772221	616539.6	419331	3	2	4	2	866	8.27	3.87	2	6.027	0		High	High
1240749293216840000	Q13526	3311393	4146975	3311393	2251888	23	3	4	3	163	8.82	5.78	3	14.913	0	PIN1	High	High
-5392183546824290000	Q8NEY1	772106.1	966548	772106.1	524854.3	1	2	4	2	1877	8.07	0	2	6.889	0	NAV1	High	Peak Found
6030770174415930000	P52294	2928688	3662888	2928688	1989020	10	7	12	3	538	5.01	14.23	7	19.149	0	KPNA1	High	High

-6077742637667710000	Q00535	633447.3	792158.2	633447.3	430157.3	7	2	5	1	292	7.66	1.88	2	7.08	0	CDK5	High	High
9079512265754270000	Q3SY69	11113300	13875915	11113300	7534892	19	17	35	16	923	6.52	20.2	17	49.132	0	ALDH1L2	High	High
1949176893953300000	Q9NZZ3	863366.9	1076190	863366.9	584392.2	13	2	4	2	219	4.83	5.4	2	7.531	0	CHMP5	High	High
-4068487784358330000	Q8TDX7	1681697	2090774	1681697	1135331	11	3	6	3	302	8.25	0	3	7.527	0	NEK7	High	High
-4968943538571680000	Q96GC5	582975.3	723953.6	582975.3	393120.9	6	1	3	1	212	8.98	1.94	1	5.343	0	MRPL48	High	High
2431037713967110000	Q9NW13	880265.7	1093020	880265.7	593530.8	3	3	6	3	759	9.22	4.3	3	9.016	0	RBM28	High	High
-105519067553436000	Q04323	2231220	2762122	2231220	1499886	7	2	7	2	297	5.25	12.66	2	9.379	0	UBXN1	High	High

-1610110799858010000	P20674	2793760	3450517	2793760	1873698	16	3	9	3	150	6.79	11.86	3	7.815	0	COX5A	High	High
8827666052570190000	Q15056	4810942	5940458	4810942	3225784	22	4	10	4	248	7.23	13.03	4	14.776	0	EIF4H	High	High
8165870367093880000	Q86TI2	7524373	9282655	7524373	5040662	9	8	18	8	863	6.46	14.54	8	27.478	0	DPP9	High	High
-909250529717419000	P26447	3165438	3904874	3165438	2120423	36	4	10	4	101	6.11	6.44	4	10.263	0	S100A4	High	High
-6469801124856570000	P36404	1621728	1998392	1621728	1085166	24	4	7	4	184	6.34	7.64	4	13.62	0	ARL2	High	High
-4076214252454430000	Q969G3	790336.9	972819.7	790336.9	528260	9	3	4	3	411	4.88	7.26	3	10.986	0	SMARCE1	High	High
5114472407412580000	P02788	2401072	2955331	2401072	1604802	6	4	9	4	710	8.12	13.8	4	11.34	0	LTF	High	High

-4996953049836210000	O14618	1097409	1349608	1097409	732863.5	14	4	7	4	274	5.58	8.04	4	10.432	0	CCS	High	High
5857120018545350000	Q8NFH3	859725.9	1056931	859725.9	573934.2	6	2	3	2	380	5.63	5.6	2	8.468	0	NUP43	High	High
8648350258145660000	Q15717	20126469	24732314	20126469	13430127	31	9	31	9	326	9.17	35.06	9	37.654	0	ELAVL1	High	High
8004592446864810000	O75688	817640.4	1003424	817640.4	544878.7	6	3	4	3	479	5.05	3.87	3	7.022	0	PPM1B	High	Peak Found
-3635452200201590000	Q12904	4351228	5329008	4351228	2893755	23	5	11	5	312	8.43	6.79	5	24.732	0	AIMP1	High	High
-8291794919948140000	Q14203	16017277	19556208	16017277	10619401	16	15	30	15	1278	5.81	32.82	15	70.012	0	DCTN1	High	High
-2847148615950550000	P20700	425943.2	519422.2	425943.2	282056.3	4	3	5	2	586	5.16	4.59	3	8.571	0	LMNB1	High	High

-7941008687587080000	O43570	254512.6	310229.3	254512.6	168460.5	4	1	3	1	354	7.23	0	1	3.656	0.001	CA12	High	High
3857525489381910000	A8MXV4	217899.5	265312.7	217899.5	144070	4	1	1	1	375	7.64	0	1	2.129	0.01	NUDT19	High	Peak Found
6802113826893240000	P07108	5569366	6760413	5569366	3671035	51	3	5	3	87	6.57	11.07	3	16.294	0	DBI	High	High
5299507636150980000	Q9Y697	1116761	1355421	1116761	736019.9	9	4	8	4	457	8.31	4.28	4	12.153	0	NFS1	High	High
2927347659774260000	Q9H7B2	604133.3	732326.6	604133.3	397667.6	8	2	4	2	306	9.99	2.33	2	10.321	0	RPF2	High	High
2099795290618700000	Q9NYU1	1639697	1987443	1639697	1079220	3	5	13	2	1516	6.89	8.01	5	12.606	0	UGGT2	High	Peak Found
-7043642977461230000	Q92575	1276757	1546823	1276757	839955.1	6	2	2	2	508	6.38	5.95	2	11	0	UBXN4	High	Peak Found

8733073153951390000	Q9BU23	6430877	7776023	6430877	4222532	4	3	6	3	707	10.1	1.98	3	6.37	0	LMF2	High	High
-4765552648658300000	Q96JH7	682059.7	824246.6	682059.7	447581.9	2	2	3	2	1222	7.2	4.48	2	8.335	0	VCPIP1	High	High
-1068600970013110000	Q99584	7980400	9627783	7980400	5228073	43	6	15	6	98	6.16	13.33	6	15.383	0	S100A13	High	High
-7331601319371960000	P21964	6702539	8062644	6702539	4378173	22	4	9	4	271	5.47	3.94	4	11.385	0	COMT	High	High
-5390707735378390000	Q8IYB3	463866.6	557045.4	463866.6	302486.5	1	1	2	1	904	11.84	1.94	1	3.811	0.001	SRRM1	High	High
1737787773374730000	P12277	5908977	7084430	5908977	3846983	15	5	9	4	381	5.59	9.66	5	15.426	0	CKB	High	High
-785716841192	Q9Y5B8	207349.2	248404.9	207349.2	134888.7	3	1	3	1	376	6.47	0	1	3.389	0.002	NME7	High	Peak Found

0130000																		
-6090359432096680000	Q8NCN5	3995499	4784516	3995499	2598085	5	4	4	4	879	6.35	5.32	4	10.746	0	PDPR	High	Peak Found
295875548179926000	P12235	2438309	2916088	2438309	1583492	25	9	24	4	298	9.76	41.23	9	39.724	0	SLC25A4	High	High
-1393141994460150000	Q6ZQW0	970680.1	1160511	970680.1	630180.3	2	1	1	1	420	6.9	0	1	2.139	0.01	IDO2	High	Peak Found
5539470002617870000	Q9P2R7	4224946	5050287	4224946	2742404	10	5	12	5	463	7.42	2.6	5	16.492	0	SUCLA2	High	High
4793391525152750000	P61020	1711531	2044254	1711531	1110070	33	6	20	3	215	8.13	34.48	6	23.795	0	RAB5B	High	High
-154777414140805000	P20810	2535568	3027821	2535568	1644166	8	5	10	5	708	5.07	10.15	5	18.58	0	CAST	High	High
-6717857941727910000	Q8WZA9	448665.8	535147.4	448665.8	290595.4	4	2	2	2	623	4.88	1.88	2	3.688	0.001	IRGQ	High	Peak Found

7835862942369070000	P06753	12066361	14390130	12066361	7814120	43	16	68	7	285	4.72	138.74	16	100.77	0	TPM3	High	High
-8252267867784180000	Q86YP4	680200.8	810731.4	680200.8	440242.9	3	2	3	1	633	9.94	2.24	2	6.564	0	GATAD2A	High	Peak Found
-3431130476793290000	Q8IUR0	765701.4	911980	765701.4	495222.9	4	1	4	1	188	9.66	3.9	1	2.431	0.007	TRAPPC5	High	High
-1208867046530030000	P47813	5225624	6215350	5225624	3375056	39	5	8	5	144	5.24	3.75	5	14.455	0	EIF1AX; LOC101060318; LOC107984923	High	High
-4437370582903790000	Q2M2H8	6308180	7497003	6308180	4071019	0	1	2	1	2515	5.21	1.64	1	3.424	0.002	LOC93432; MGAM2	High	High
-4846658872779080000	P61964	439268.7	521708.6	439268.7	283297.9	3	1	2	1	334	8.27	1.74	1	3.869	0	WDR5	High	High
-249192115312	Q9UHV9	1651028	1960038	1651028	1064339	15	2	3	2	154	6.58	5.31	2	9.727	0	PFDN2	High	High

9940000																		
-3452513591771990000	Q6ICG6	393522.9	467119.4	393522.9	253654.9	2	1	1	1	404	7.94	2	1	3.648	0.001	KIAA0930	High	Peak Found
1612491288449710000	Q13155	8357931	9919393	8357931	5386423	26	6	13	6	320	8.22	15.38	6	21.86	0	AIMP2	High	High
-8447148284608470000	Q9H444	2366112	2807813	2366112	1524697	16	3	8	3	224	4.82	14.61	3	16.121	0	CHMP4B	High	High
-3321226471008760	Q9Y2Q3	1621325	1923472	1621325	1044483	12	2	4	2	226	8.41	4.93	2	8.215	0	GSTK1	High	High
8504895740421250000	Q9Y4W2	352426.1	417774.3	352426.1	226859.5	1	1	2	1	734	4.73	1.61	1	2.45	0.007	LAS1L	High	High
5073984553663010000	Q86UV5	390005.9	462091.5	390005.9	250924.7	1	1	2	1	1035	6.05	0	1	2.617	0.006	USP48	High	Peak Found
2746028524908130000	P16070	90168011	1.07E+08	90168011	57988662	14	11	33	11	742	5.33	56.82	11	57.259	0	CD44	High	High

4624493070796370000	Q7Z7H8	572903.9	678179.5	572903.9	368264.7	4	1	2	1	261	9.58	4.22	1	3.764	0.001	MRPL10	High	High
4270153949205940000	Q9UBV2	3218805	3809985	3218805	2068896	13	7	16	7	794	5.39	23.53	7	32.879	0	SEL1L	High	High
2003542592102700000	Q9H4I3	2497605	2955922	2497605	1605123	3	1	1	1	376	8	0	1	2.23	0.009	TRABD	High	Peak Found
-1552754402547080000	Q5MIZ7	173737.8	205488.2	173737.8	111584.1	3	3	3	1	849	4.96	0	3	5.964	0	SMEK2; PPP4R3B	High	Peak Found
-6980726595472840000	P24941	470750.1	556632.8	470750.1	302262.5	14	4	8	2	298	8.68	2.14	4	13.874	0	CDK2	High	High
5366408655713320000	Q07817	217789.6	257477.2	217789.6	139815.1	4	1	2	1	233	4.93	0	1	2.368	0.008	BCL2L1	High	Peak Found
-6528342238616590000	Q92542	1814428	2144623	1814428	1164572	7	5	9	5	709	5.99	12.05	5	16.033	0	NCSTN	High	High

7129338671837300000	P78559	7424774	8772043	7424774	4763390	10	20	31	18	2803	4.92	25.23	20	51.28	0	MAP1A	High	High
-6657686065862130000	Q9Y371	3549877	4191514	3549877	2276074	15	6	12	5	365	6.04	9.34	6	18.676	0	SH3GLB1	High	High
-3741204735065000000	Q8WXF1	2801670	3302437	2801670	1793288	7	4	7	3	523	6.67	7.99	4	10.933	0	PSPC1	High	High
-4994783386370790000	P09471	599234.4	706294.8	599234.4	383531.8	11	3	9	1	354	5.53	15.35	3	12.845	0	GNAO1	High	High
-818357579634992000	Q13671	3556937	4187903	3556937	2274113	6	5	5	5	783	8.02	3.53	5	11.863	0	RIN1	High	Peak Found
-7462391840116260000	Q05086	676194.6	796088	676194.6	432291.3	3	2	2	2	875	5.22	0	2	5.773	0	UBE3A	High	Peak Found
-178906797964	P46976	3662301	4311154	3662301	2341041	10	3	7	3	350	5.53	7.55	3	10.576	0	GYG1	High	High

3480000																		
4697106387219560000	Q6P2E9	1922140	2262248	1922140	1228445	2	3	4	3	1401	5.86	3.79	3	9.25	0	EDC4	High	High
1047119725647510000	P23588	2406669	2831333	2406669	1537469	14	6	10	6	611	5.73	15.32	6	29.113	0	EIF4B	High	High
-5911471042018290000	Q8ND56	206066.3	242344	206066.3	131597.5	2	1	2	1	463	9.52	1.67	1	3.206	0.002	LSM14A	High	High
-8300178027695560000	P34059	3664059	4301704	3664059	2335909	4	2	6	2	522	6.74	1.73	2	9.288	0	GALNS	High	High
-8114492968310580000	P98194	506225.7	593416.2	506225.7	322236.5	1	1	1	1	919	6.74	0	1	3.929	0	ATP2C1	High	Peak Found
-1866024987760350000	Q9Y2A7	6792971	7952020	6792971	4318102	12	11	20	11	1128	6.62	28.84	11	43.676	0	NCKAP1	High	High
-545964	Q9NWH9	1204609	1409635	1204609	765459.5	2	3	4	3	10	7.87	2.46	3	8.502	0	SLTM	High	High

3991948160000										34								
-5464412286684590000	Q6NSJ5	442384.7	517564.4	442384.7	281047.5	3	2	2	1	796	6.96	1.75	2	5.795	0	LRRC8E	Peak Found	High
-11327904792231000	Q7Z2K6	4376910	5119829	4376910	2780167	9	8	14	8	904	7.52	19.1	8	33.777	0	ERMP1	High	High
6933649257231020000	P16930	1783690	2085344	1783690	1132383	5	2	5	2	419	6.95	4.7	2	5.06	0	FAH	High	High
-6735907081845110000	Q9Y2L1	3627919	4241461	3627919	2303196	8	7	12	7	958	7.14	13.59	7	24.403	0	DIS3	High	High
1131809670189220000	Q96EY7	1145143	1336631	1145143	725816.4	4	2	3	2	689	6.42	0	2	7.121	0	PTCD3	High	High
735333771594595000	Q9BYN8	813451.7	949303.4	813451.7	515490.2	14	3	4	3	205	10.39	4.22	3	7.418	0	MRPS26	High	Peak Found
4686891910023400000	P62140	3438732	4011366	3438732	2178250	25	7	22	1	327	6.19	20.92	7	44.275	0	PPP1CB	High	High

-5925761923209870000	Q12824	1235808	1441194	1235808	782596.3	3	1	1	1	385	6.23	2.37	1	3.759	0.001	SMARCB1	Peak Found	High
2174498408520760000	Q02241	478773.7	558336.5	478773.7	303187.6	3	2	3	2	960	8.51	0	2	6.295	0	KIF23	High	High
-3098195104215070000	P23919	16435740	19153532	16435740	10400740	46	10	28	10	212	8.27	25.96	10	36.518	0	DTYMK	High	High
-694554637971528000	Q9Y2H6	1118182	1301105	1118182	706525.6	2	3	5	3	1198	6.71	0	3	9.482	0	FNDC3A	High	High
-5647606517486860000	Q8N138	1161549	1349877	1161549	733009.6	7	1	3	1	153	9.64	1.92	1	3.715	0.001	ORMDL3	High	High
2244898098371360000	Q86VS8	1760111	2044791	1760111	1110361	8	5	8	5	718	5.17	4.26	5	15.219	0	HOOK3	High	High
8380855263151820000	Q96EC8	617181.7	716533.4	617181.7	389091.6	5	1	3	1	236	5.64	5.82	1	4.189	0	YIPF6	High	Peak Found

3875322263221300000	P51553	3987203	4629045	3987203	2513661	12	4	12	4	393	8.5	12.99	4	18.34	0	IDH3G	High	High
8506961570143960000	Q99497	56988787	66138659	56988787	35914577	57	12	51	12	189	6.79	75.65	12	69.108	0	PARK7	High	High
-6781148619442230000	Q96N66	4934244	5726225	4934244	3109452	9	4	12	4	472	8.97	22.55	4	24.658	0	MBOAT7	High	High
8981469479003520000	Q9BZE1	307294.6	356355.4	307294.6	193507.9	2	1	2	1	423	8.59	0	1	2.72	0.005	MRPL37	High	High
-3563159699507810000	P06280	148947.1	172659.1	148947.1	93757.26	12	4	4	4	429	5.6	0	4	11.273	0	GLA	High	Peak Found
-367492356169293000	Q12965	1654322	1916990	1654322	1040963	6	6	11	6	1108	8.92	10.15	6	19.396	0	MYO1E	High	High
2447510198658430000	Q9H910	968721.8	1122090	968721.8	609316.5	15	2	5	2	190	9.26	4.38	2	7.407	0	HN1L; JPT2	High	High

-478504765432675000	P30520	4161411	4816337	4161411	2615365	15	7	20	7	456	6.55	14.6	7	28.006	0	ADSS	High	High
-852377223734167000	Q4G176	1605940	1858360	1605940	1009126	5	3	6	3	576	8.37	5.85	3	8.27	0	ACSF3	High	High
-6719730535871430000	P16989	5504451	6369236	5504451	3458619	21	8	24	3	372	9.77	45.68	8	46.76	0	CSDA; YBX3	High	High
-6708028902102910000	P21281	9979879	11525244	9979879	6258432	23	9	16	9	511	5.81	24.7	9	35.19	0	ATP6V1B2	High	High
-81494833362564800	Q8WVJ2	1382040	1594977	1382040	866103.3	13	2	3	2	157	5.07	2.44	2	6.73	0	NUDCD2	High	High
5987314834012530000	O60888	1484293	1712771	1484293	930068.1	8	1	7	1	179	5.5	13.76	1	6.961	0	CUTA	High	High
-4669002677123590000	P06493	15405821	17756936	15405821	9642361	27	8	25	6	297	8.4	40.5	8	30.586	0	CDK1	High	High

3075207303668690000	P37198	1153004	1328933	1153004	721636.3	7	4	8	4	522	5.31	10.88	4	14.681	0	NUP62	High	High
-4709536422529610000	P20618	11081771	12753411	11081771	6925350	26	5	13	5	241	8.13	16.39	5	20.699	0	PSMB1	High	High
419048109363271000	Q10713	2708819	3108471	2708819	1687960	15	7	13	7	525	6.92	10.93	7	22.605	0	PMPCA	High	High
7666744292030770000	Q9BRK5	4250475	4874376	4250475	2646881	22	6	18	6	362	4.86	15.76	6	32.905	0	SDF4	High	High
7014036665860800000	Q13459	926772.1	1062602	926772.1	577013.5	3	6	9	5	2157	8.75	0	6	12.383	0	MYO9B	High	High
-4874948600473350000	O60218	1953327	2235699	1953327	1214028	11	3	6	3	316	7.84	1.91	3	10.954	0	AKR1B10	High	High
3279356034599550000	Q9P0V3	3358789	3843931	3358789	2087329	6	6	11	6	963	7.71	11.53	6	17.508	0	SH3BP4	High	High
-181677439452	P26232	186005	212868	186005	115591.5	6	5	16	1	953	5.71	14.29	5	15.833	0	CTNNA2	High	High

0300000																		
8359022296663290000	Q14258	7480208	8548259	7480208	4641871	12	7	10	7	630	8.09	8.63	7	23.291	0	TRIM25	High	High
8978349436204480000	Q13148	5938165	6784154	5938165	3683928	9	4	10	4	414	6.19	20.03	4	26.71	0	TARDBP	High	High
3347831162215740000	Q9UEW8	788516.8	900816.6	788516.8	489160.9	7	3	5	1	545	6.29	1.7	3	5.623	0	STK39	High	High
-5905184492137340000	P08559	2.82E+08	3.22E+08	2.82E+08	1.75E+08	12	5	12	5	390	8.06	13.12	5	13.978	0	PDHA1	High	High
-3588526575422440000	P42285	7382508	8426488	7382508	4575747	11	11	17	11	1042	6.52	17.8	11	32.795	0	SKIV2L2	High	High
2383613337026510000	Q93052	2427634	2768855	2427634	1503542	15	5	7	5	612	7.37	2.44	5	17.019	0	LPP	High	High
3478663416261510000	O43772	3219332	3671007	3219332	1993428	8	3	4	3	301	9.41	1.89	3	6.067	0	SLC25A20	High	Peak Found

2541081725476020000	Q8NBJ7	3432693	3913890	3432693	2125318	16	4	9	4	301	8	11.96	4	16.261	0	SUMF2	High	High
-9211506236520860000	O76071	328798.6	374876.9	328798.6	203565.4	2	1	2	1	339	4.97	0	1	2.429	0.007	CIAO1	High	High
8403799351275240000	Q9Y5S2	261126.6	297671.2	261126.6	161641.3	1	1	2	1	1711	6.37	0	1	2.143	0.009	CDC42BPB	High	High
6077570543552490000	Q8N1G4	18746507	21364141	18746507	11601144	25	13	31	13	583	8.28	28.32	13	57.548	0	LRRC47	High	High
-5095696648215940000	P09972	10088596	11490549	10088596	6239591	20	6	29	2	364	6.87	63.11	6	52.214	0	ALDOC	High	High
7945611468628580000	Q96I25	1460254	1663086	1460254	903087.8	7	3	3	3	401	5.97	0	3	7.972	0	RBM17	High	Peak Found
-4307491490075500000	P32322	21911101	24949262	21911101	13547934	37	10	33	9	319	7.61	60.06	10	53.505	0	PYCR1	High	High

-8194889804076800000	Q9UBU9	10439721	11864280	10439721	6442535	5	3	6	3	619	8.51	3.87	3	7.073	0	NXF1	High	High
7996509007212380000	B2RTY4	360753.9	409952.3	360753.9	222612	1	2	2	1	2548	8.88	0	2	2.923	0.004	MYO9A	High	Peak Found
-1088137077242890000	P51659	15553252	17671769	15553252	9596114	29	16	31	16	736	8.84	42.23	16	72.517	0	HSD17B4	High	High
-5669236386910030000	P46783	24617134	27933616	24617134	15168496	44	9	30	9	165	10.15	44.23	9	43.545	0	RPS10	High	High
-1528073227460310000	Q9H9T3	421851.7	478375.2	421851.7	259767.1	10	4	6	4	547	8.88	5.86	4	13.704	0	ELP3	High	High
-3914763755689380000	Q86UU1	307485.5	348567.5	307485.5	189278.9	1	1	2	1	1377	8.63	4.7	1	4.64	0	PHLDB1	High	Peak Found
193290723324093000	P33316	4513732	5107792	4513732	2773630	21	5	9	5	252	9.36	12.79	5	15.58	0	DUT	High	High

-8649705542655530000	Q8N573	204247.9	231017.2	204247.9	125446.8	4	2	2	2	874	5.47	0	2	5.414	0	OXR1	High	Peak Found
-2766829585988960000	O75190	3315147	3747905	3315147	2035185	7	2	3	2	326	9.16	2.36	2	6.119	0	DNAJB6	Peak Found	High
-285446999307316000	A9QM74	765045.8	864093.5	765045.8	469219.6	2	1	1	1	516	6.04	2.38	1	4.699	0	KPNA7	High	Peak Found
-8120903851432860000	P01889	2721558	3073544	2721558	1668994	16	4	14	2	362	5.85	24.74	4	19.047	0	HLA-B	High	High
6452221919959730000	O75122	292676.5	330501.1	292676.5	179468.5	2	3	4	2	1294	8.4	1.74	3	6.772	0		High	Peak Found
6076185631256900000	Q9P016	218298.8	246317.8	218298.8	133755.4	7	2	2	2	225	9.25	0	2	3.127	0.003	THYN1	High	Peak Found
-6212598245542670000	Q92879	706283.7	796706.8	706283.7	432627.3	7	3	5	3	486	8.46	4.11	3	10.319	0	CELF1	High	High

5142259406840290000	Q14738	313654.9	353723.2	313654.9	192078.6	6	3	10	1	602	8.13	1.89	3	8.113	0	PPP2R5D	Peak Found	High
6088825967158890000	O43290	1731823	1952101	1731823	1060029	7	5	7	5	800	6.13	4.02	5	13.639	0	SART1	High	High
-453379581051164000	P30086	18910092	21312499	18910092	11573101	40	7	24	7	187	7.53	40.65	7	31.56	0	PEBP1	High	High
-7380698507310520000	P41227	1312124	1478810	1312124	803022.6	19	4	5	4	235	5.64	4.85	4	15.67	0	NAA10	High	High
-1621109291993520000	P01008	214013.9	241191.2	214013.9	130971.5	2	1	1	1	464	6.71	1.73	1	3.009	0.003	SERPINC1	High	Peak Found
469821544412833000	Q8NEJ9	592786.5	668001	592786.5	362737.5	4	1	4	1	315	9.57	0	1	3.71	0.001	NGDN	High	High
-4653603657253560000	Q7Z7H5	5153018	5797365	5153018	3148082	23	6	11	5	227	8.28	18.47	6	21.028	0	TMED4	High	High

722049298036069000	Q9BVK6	16492990	18551304	16492990	10073719	43	10	15	9	235	8.02	15.21	10	29.543	0	TMED9	High	High
6320190731642740000	Q9NVV5	338455.5	380412	338455.5	206571.1	4	1	2	1	238	7.17	4.82	1	5.277	0		High	High
2292844797767430000	Q9H074	2500881	2810193	2500881	1525990	7	3	7	3	479	4.81	11.51	3	12.611	0	PAIP1	High	High
-1135638411849560000	O76021	1229146	1380989	1229146	749904	8	4	7	4	490	10.13	4.67	4	11.612	0	RSL1D1	High	High
6653265809957970000	Q14157	874909.7	982209.5	874909.7	533358.8	3	2	3	2	1087	7.11	1.9	2	5.903	0	UBAP2L	High	High
-6465314109903610000	Q99615	903005.1	1012143	903005.1	549613.3	11	5	5	5	494	6.96	4.12	5	13.172	0	DNAJC7	High	Peak Found
8338185896590090000	Q9H0B6	2209845	2475663	2209845	1344333	11	6	10	3	622	7.15	8.46	6	15.676	0	KLC2	High	Peak Found
224152608357	Q9ULX6	544748.5	610106.9	544748.5	331299.9	6	2	4	2	646	5.05	1.86	2	9.457	0	AKAP8L	High	High

2890000																		
-7224600777690590000	P50991	1.37E+08	1.53E+08	1.37E+08	83051448	47	25	92	24	539	7.83	115.66	25	106.693	0	CCT4	High	High
-7852506099469630000	Q9H7D0	3011460	3361861	3011460	1825556	3	6	10	6	1870	7.96	4.98	6	16.198	0	DOCK5	High	High
-278084852242495000	P62861	2648325	2953831	2648325	1603988	17	1	3	1	59	12.15	0	1	3.697	0.001	FAU	High	High
2514407203909910000	O95363	326189	363615.6	326189	197450.3	2	1	3	1	451	7.46	0	1	2.483	0.007	FARS2	High	High
-917272071926626000	Q99797	376538.1	419716.8	376538.1	227914.4	1	1	3	1	713	7.05	1.66	1	3.995	0	MIPEP	High	High
-6342560622817440000	Q96PU8	380367.7	423948	380367.7	230212	7	2	4	2	341	8.56	0	2	5.237	0	QKI	High	High
70063898949050700	Q96BI1	862896.4	961108	862896.4	521900.3	3	1	6	1	424	9.57	10.29	1	4.017	0	SLC22A18	High	High

2025093551518490000	Q9UBX3	2854000	3176314	2854000	1724800	13	4	8	4	287	9.54	11.85	4	11.368	0	SLC25A10	High	High
908398624639479000	P62913	35413856	39387944	35413856	21388419	29	7	15	7	178	9.6	15.82	7	21.087	0	RPL11	High	High
-3154088969782950000	Q9NP58	1501198	1666573	1501198	904981.4	7	5	8	5	842	8.48	1.97	5	14.472	0	ABCB6	High	High
2921825731244050000	Q99714	8234077	9137355	8234077	4961761	37	7	16	7	261	7.78	21.44	7	27.818	0	HSD17B10	High	High
-1129418023889810000	P40429	8204006	9102169	8204006	4942654	20	5	11	5	203	10.93	18.91	5	19.456	0	RPL13A	High	High
1328397515797570000	A6NDG6	759838.7	842842.6	759838.7	457679.9	5	2	3	2	321	6.14	3.57	2	3.746	0.001	PGP	High	High
-536665306058471000	Q07020	5233286	5801436	5233286	3150292	22	4	8	4	188	11.72	18.18	4	15.416	0	RPL18	High	High
-405652652129	Q14690	2759377	3058898	2759377	1661041	5	7	12	7	18	8.87	5.61	7	20.874	0	PDCD11	High	High

7060000										71								
-7926750901402690000	Q9UKG1	4325192	4793034	4325192	2602711	7	4	5	4	709	5.41	1.9	4	10.69	0	APPL1	High	High
-5723353511819680000	Q92947	764716.8	847242.9	764716.8	460069.3	9	2	3	2	438	8.06	2.06	2	7.028	0	GCDH	High	High
-5219962160653880000	Q15785	12286233	13611920	12286233	7391537	36	11	22	11	309	8.98	19.68	11	35.053	0	TOMM34	High	High
-985965989456980000	Q8TEQ6	2714872	3007121	2714872	1632925	5	7	13	7	1508	6.62	8.08	7	17.412	0	GEMIN5	High	High
8832352295829310000	P09622	18238459	20192874	18238459	10965123	18	10	19	10	509	7.85	28.54	10	37.806	0	DLD	High	High
-4487628495145980000	Q13425	1075818	1190055	1075818	646222.9	6	3	4	3	540	8.82	6.2	3	10.016	0	SNTB2	High	High

5235213345735990000	O96000	2516716	2780727	2516716	1509989	35	5	7	5	172	8.48	4.02	5	15.181	0	NDUFB10	High	High
7629254407620290000	Q15428	1885169	2081092	1885169	1130074	7	3	6	3	464	9.64	4.55	3	11.296	0	SF3A2	High	High
657128700828351000	Q16643	12580475	13866674	12580475	7529874	12	8	15	8	649	4.45	22.84	8	33.919	0	DBN1	High	High
-2764493771843330000	P08651	643115.5	708327.6	643115.5	384635.7	3	1	2	1	508	8.38	2.53	1	4.362	0	NFIC	High	High
1736163179168070000	Q9Y2V7	259603.7	285917.1	259603.7	155258.5	2	1	1	1	657	5.76	0	1	3.424	0.002	COG6	High	Peak Found
-3366896728289430000	Q9NUQ6	1353693	1490795	1353693	809530.9	10	4	6	4	558	9.64	1.75	4	15.599	0	SPATS2L	High	High
-9207268972416960000	O15066	186389.7	205236.1	186389.7	111447.2	2	1	1	1	747	7.69	0	1	5.58	0	KIF3B	High	Peak Found
-141937	P42677	11018527	12131303	11018527	6587534	25	4	12	3	84	9.45	26	4	23.113	0	RPS27	High	High

0867446700000																		
-2604748441743250000	Q6P1J9	1585623	1745692	1585623	947944.6	7	4	5	4	531	9.61	5.24	4	9.513	0	CDC73	High	High
-3472433998448830000	O75323	2131871	2346050	2131871	1273951	12	5	13	3	286	9.36	13.96	5	13.042	0	GBAS; NIPSNAP2	High	High
-2070723625944500000	Q01629	2194589	2413654	2194589	1310661	12	1	5	1	132	7.42	11.81	1	9.365	0	IFITM2	High	High
-7795104145569460000	Q2M389	1375026	1511576	1375026	820815	3	4	8	4	1173	7.44	8.15	4	11.603	0	KIAA1033; WASHC4	High	High
-6923013739007890000	P60033	3768886	4142829	3768886	2249637	12	2	2	2	236	5.29	5.23	2	12.424	0	CD81	High	Peak Found
3827413943900730000	Q9Y2W2	465405.9	511323.8	465405.9	277658.8	2	1	1	1	641	8.38	1.98	1	3.074	0.003	WBP11	High	Peak Found

-4400715945048600000	P17813	1349953	1481838	1349953	804666.6	8	4	6	4	658	6.61	2.3	4	10.671	0	ENG	High	High
-516006275001987000	Q14566	16607316	18217436	16607316	9892422	19	16	27	16	821	5.41	40.21	16	60.433	0	MCM6	High	High
983713321704176000	O00264	6058405	6637435	6058405	3604256	21	5	14	4	195	4.7	13.94	5	18.368	0	PGRMC1	High	High
3312961601966620000	Q96EY8	254666.6	278544.2	254666.6	151254.9	4	1	1	1	250	8.6	0	1	2.179	0.009	MMAB	High	Peak Found
2809797377402090000	P42785	22124961	24180562	22124961	13130515	15	8	16	8	496	7.21	12.44	8	28.038	0	PRCP	High	High
8694247867552340000	Q9BQ75	739398.7	807744.7	739398.7	438621.1	6	2	3	2	279	9.19	0	2	4.457	0	CMSS1	High	Peak Found
-8162183631045640000	Q01085	3319243	3625773	3319243	1968865	7	3	4	3	375	7.74	3.76	3	8.148	0	TIAL1	High	High
-772324153782	Q02388	1720160	1878436	1720160	1020027	3	8	10	8	29	6.27	8.11	8	18.896	0	COL7A1	High	High

4490000										44								
-1575128181987340000	Q9GZM5	454660.1	496426.1	454660.1	269569	5	2	8	2	350	5.76	0	2	4.272	0	YIPF3	High	High
3065352799485710000	Q9P0P0	185115.1	202107.5	185115.1	109748.3	6	1	1	1	153	5.06	0	1	2.134	0.01	RNF181	High	Peak Found
-3373715061212050000	P10620	12826028	14002161	12826028	7603446	27	4	16	4	155	9.39	24.26	4	25.89	0	MGST1	High	High
3800161177308870000	Q8NEM2	1477826	1612942	1477826	875859	4	3	5	3	672	4.75	3.41	3	8.053	0	SHCBP1	High	High
8643593738164180000	Q9H6S0	1280354	1396850	1280354	758516.8	2	2	2	2	1430	8.4	4.09	2	7.722	0	YTHDC2	High	High
-2243304084648640000	P49585	965183.5	1052732	965183.5	571653.7	8	2	3	2	367	7.25	0	2	6.193	0	PCYT1A	High	High
-468234324268	O43818	1168779	1274343	1168779	691993.2	10	4	6	4	475	7.85	8.09	4	13.261	0	RRP9	High	High

7860000																		
4267228453861490000	Q8IVM0	936940.9	1021127	936940.9	554491.7	4	1	3	1	306	6.65	2.21	1	3.433	0.002	CCDC50	High	High
-3471624657461710000	P35555	700879.6	762816.1	700879.6	414224	1	4	4	4	2871	4.93	3.67	4	10.488	0	FBN1	High	High
-7181785604482640000	Q9NR50	4127902	4492400	4127902	2439461	17	6	8	6	452	6.47	3.96	6	15.612	0	EIF2B3	High	High
-6100821946699500000	Q5QNW6	5781227	6288558	5781227	3414809	36	5	18	2	126	10.32	26.09	5	24.414	0	HIST2H2BF	High	High
5299836175419680000	P42166	3604158	3918065	3604158	2127586	12	6	7	2	694	7.66	7.04	6	16.856	0	TMPO	High	High
-4736314326613780000	P62277	47861565	52000400	47861565	28237228	38	7	30	7	151	10.54	46.85	7	36.527	0	RPS13	High	High

-2364171353668640000	Q00653	882783.1	958594.2	882783.1	520535.3	4	3	5	3	900	6.25	5.7	3	11.95	0	NFKB2	High	High
-5860754846925800000	O95400	1043542	1132238	1043542	614827.4	7	2	2	2	341	4.61	2.01	2	5.201	0	CD2BP2	Peak Found	High
-6442873191784740000	Q9BXW7	4714131	5112915	4714131	2776412	13	4	14	4	423	8.13	25.7	4	25.129	0	CECR5; HDHD5	High	High
-8787657634019770000	Q2TAA2	141682.5	153634.4	141682.5	83426.45	10	2	3	2	248	5.3	1.63	2	3.62	0.001	IAH1	High	High
-2411801021050740000	Q9NSE4	15353496	16618227	15353496	9024021	25	21	43	21	1012	7.2	48.58	21	74.424	0	IARS2	High	High
-5199431404915780000	P49810	450056.8	486935.5	450056.8	264415.4	2	1	2	1	448	4.59	0	1	2.353	0.008	PSEN2	High	Peak Found

-1153318479582770000	P53677	496305.5	536863.4	496305.5	291527.3	4	2	3	1	418	7.56	1.72	2	4.583	0	AP3M2	High	High
-5552515509337540000	Q8WXE9	2998846	3243471	2998846	1761268	1	1	3	1	905	5.39	6.82	1	4.095	0	STON2	High	High
2037005348220660000	O00151	3627845	3923006	3627845	2130269	25	6	8	6	329	7.02	10.41	6	23.489	0	PDLIM1	High	High
7903736519056340000	P53985	5074773	5483981	5074773	2977908	9	4	9	4	500	8.66	11.77	4	24.029	0	SLC16A1	High	High
-9120785921578440000	A0A0B4J2D5	4218705	4558105	4218705	2475140	13	4	6	4	268	8.27	7.69	4	12.109	0	C21orf33; LOC102724023	High	High
-1964659957025430000	Q9H2H8	646753.5	698578.4	646753.5	379341.7	9	1	3	1	161	6.79	2.16	1	4.153	0	PPIL3	High	High
2965496322835630000	Q9NYM9	131990.7	142549.4	131990.7	77407.07	14	1	1	1	111	8.16	0	1	2.591	0.006	BET1L	High	Peak Found

5815926444524440000	Q7Z406	384267.1	414978.8	384267.1	225341.5	5	12	32	1	1995	5.6	68.78	12	55.483	0	MYH14	High	High
4449270274494760000	O15269	755564.4	815505.4	755564.4	442835.3	2	1	1	1	473	6.01	1.9	1	2.123	0.01	SPTLC1	High	Peak Found
4400189645946560000	O00488	271269.5	292578.7	271269.5	158875.9	7	1	2	1	134	9.82	0	1	2.32	0.008	ZNF593	High	High
-4439921504138330000	Q6P4E1	241544.5	260425.2	241544.5	141416	3	1	2	1	436	5.29	0	1	2.245	0.009		High	High
8343966935655590000	P56385	4969993	5356897	4969993	2908899	25	2	4	2	69	9.35	7.18	2	5.891	0	ATP5I	High	High
-3754812835242020000	Q6ZSR9	420023.8	452677.3	420023.8	245812.6	3	1	3	1	355	5.26	6.5	1	4.79	0		High	High
-9217624131527770000	O00330	2658802	2864809	2658802	1555647	10	5	8	5	501	8.66	7.73	5	15.641	0	PDHX	High	High

8766016140567220000	Q08380	3825307	4121359	3825307	2237978	11	6	13	6	585	5.27	7.23	6	20.918	0	LGALS3BP	High	High
8472695818303020000	P22087	13539974	14581771	13539974	7918185	34	8	18	8	321	10.18	37.1	8	45.329	0	FBL	High	High
-2222501069186750000	Q86W42	1019895	1098157	1019895	596320.7	16	4	5	4	341	7.43	6.16	4	15.294	0	THOC6	High	High
5216339322949840000	Q96PZ0	1656773	1783631	1656773	968546.4	8	5	6	5	661	6.37	6.08	5	18.407	0	PUS7	High	High
4459435427997330000	O60231	79218.61	85249.95	79218.61	46292.38	1	1	1	1	1041	6.8	0	1	2.235	0.009	DHX16	Peak Found	High
8813199514956100000	O94822	315126.3	339109.1	315126.3	184142.8	2	2	2	2	1766	6.25	1.82	2	5.022	0	LTN1	High	Peak Found
-1126668279084600000	Q96AQ6	1257895	1352256	1257895	734301.5	5	3	3	3	731	5.33	3.65	3	6.701	0	PBXIP1	High	High

545176652009721000	Q9P013	208397.8	223683.7	208397.8	121464.6	5	1	1	1	229	5.71	0	1	2.772	0.004	CWC15	High	Peak Found
-933507358187812000	P48634	668518.5	717545.5	668518.5	389641.2	3	3	6	3	2157	9.45	0	3	6.358	0	PRRC2A	High	High
6598258105920110000	Q5TDH0	271772.7	291554.9	271772.7	158320	2	1	1	1	399	5.05	1.72	1	4.142	0	DDI2	High	Peak Found
5909350322516910000	P40222	12479695	13371492	12479695	7260980	36	16	29	16	546	6.52	31.35	16	55.525	0	TXLNA	High	High
-3289873417268450000	Q9NX40	1634778	1750904	1634778	950775	12	3	10	3	245	7.49	5.13	3	10.877	0	OCIAD1	High	High
-5588804604694790000	P36957	24258996	25948932	24258996	14090774	20	8	30	8	453	8.95	36.85	8	38.735	0	DLST	High	High
2210442272290700000	P09493	2542712	2719675	2542712	1476836	34	15	42	4	284	4.74	66.2	15	73.389	0	TPM1	High	High
-691950049710	Q9UJU6	1323692	1415184	1323692	768472.3	5	2	4	2	430	5.05	5.63	2	9.173	0	DBNL	High	High

1410000																		
-9189590484777070000	Q13185	6430661	6866368	6430661	3728571	23	5	10	4	183	5.33	13.31	5	28.99	0	CBX3; C15orf57; CCDC32	High	High
9221367924290970000	Q07812	3841927	4101367	3841927	2227122	26	4	11	4	192	5.22	16.36	4	18.544	0	BAX	High	High
2660523182149500000	Q9UKK9	6087913	6493871	6087913	3526298	26	6	11	6	219	4.94	10.76	6	22.077	0	NUDT5	High	High
-4350604871328290000	Q14956	1353547	1443350	1353547	783766.9	2	1	3	1	572	6.64	0	1	2.128	0.01	GPNMB	High	High
8417235359346520000	Q9NR12	5624101	5992771	5624101	3254191	17	8	21	8	457	8.41	17.22	8	24.855	0	PDLIM7	High	High
1674300386967470000	Q68CQ4	344865.2	367448.3	344865.2	199531.6	3	2	3	2	756	5.88	0	2	6.231	0	DIEXF	High	High
6901270763620920000	Q6P1N9	3532558	3759640	3532558	2041558	14	4	8	4	297	6.96	6.19	4	14.058	0	TATDN1	High	High

1614464300928510000	P55854	5258866	5592332	5258866	3036745	20	2	6	1	103	5.49	7.54	2	10.467	0	SUMO3	High	High
-5068644939638430000	P62491	27452624	29189612	27452624	15850527	41	9	29	9	216	6.57	40.18	9	30.338	0	RAB11A	High	High
5296989858477960000	Q9Y4K0	1522506	1618597	1522506	878929.4	9	5	11	5	774	6.38	2.64	5	15.551	0	LOXL2	High	High
-815619529761870000	Q6P996	4596145	4883627	4596145	2651904	12	8	16	8	788	5.38	5.9	8	25.27	0	PDXDC1; LOC102724985	High	High
1867390860608560000	Q9BRJ2	439714.7	467174.4	439714.7	253684.8	6	2	5	2	306	9.03	3.67	2	3.965	0	MRPL45	High	High
-5797732752713600000	Q15050	215604.6	228914.9	215604.6	124305.2	4	1	1	1	365	10.7	0	1	3.174	0.003	RRS1	High	Peak Found
3245672403803650000	P62263	58177779	61768974	58177779	33541754	43	10	49	10	151	10.05	87.68	10	74.325	0	RPS14	High	High

8165115417245550000	Q15293	16239654	17227458	16239654	9354844	47	14	32	14	331	5	31.23	14	64.109	0	RCN1	High	High
-8925362845928820000	Q12996	3775140	4004130	3775140	2174320	9	6	11	6	717	8.12	19.81	6	22.99	0	CSTF3	High	High
-3812741614734950000	Q7L9L4	1756099	1862318	1756099	1011275	16	3	8	3	216	6.73	9.34	3	14.59	0	MOB1B	High	High
-2591568921065240000	Q8NB90	365085.6	387049.7	365085.6	210175.5	3	2	11	1	893	5.66	19.64	2	7.998	0	SPATA5	High	High
-5403362594162540000	Q15007	516106.1	546927.3	516106.1	296992.2	8	2	3	2	396	5.19	6.09	2	10.493	0	WTAP	High	Peak Found
7415542887713690000	Q01469	14299504	15136813	14299504	8219584	70	12	31	12	135	7.01	49.78	12	74.272	0	FABP5	High	High
-332403139363	P49458	4075856	4309599	4075856	2340196	33	3	9	3	86	7.97	9.6	3	13.826	0	SRP9; SRP9P1	High	High

7840000																		
-3573373759787640000	P29279	1147092	1212430	1147092	658373.1	10	3	4	3	349	8	1.92	3	7.206	0	CTGF	High	Peak Found
5893062326726900000	Q5T750	5028112	5312604	5028112	2884847	11	3	7	3	250	7.97	6.59	3	9.491	0	C1orf68	High	High
6827410314429010000	Q9UM22	2508539	2650163	2508539	1439090	9	2	3	2	224	6.6	3.77	2	6.939	0	EPDR1	High	High
-9199223731198630000	Q9UIJ7	5658892	5975370	5658892	3244742	26	6	8	6	227	9.16	2	6	14.944	0	AK3	High	High
5100891898626730000	Q86WA6	496402.4	524078.3	496402.4	284584.7	2	1	2	1	291	9.14	0	1	2.278	0.008	BPHL	High	High
3647234260862610000	Q8TEA8	2367975	2498607	2367975	1356792	16	3	4	3	209	8.24	4.64	3	10.91	0	DTD1	High	High
1310996743308860000	Q9Y3A5	8179646	8626176	8179646	4684181	32	9	20	9	250	8.75	11.41	9	27.664	0	SBDS	High	High

-2814878339460430000	O43819	350360.3	369407.8	350360.3	200595.6	6	1	2	1	266	8.85	3.42	1	4.164	0	SCO2	High	Peak Found
-3674589448003340000	Q5T749	7297361	7690360	7297361	4176015	20	8	15	8	579	8.27	15.13	8	38.097	0	KPRP	High	High
5753903603639700000	Q15021	7013212	7387952	7013212	4011802	9	12	17	12	1401	6.61	11.74	12	35.344	0	NCAPD2	High	High
9165342661713370000	Q969U7	3524365	3710094	3524365	2014653	14	4	12	4	264	6.98	5.5	4	15.036	0	PSMG2	High	High
-8855314221236890000	Q5T0F9	264251.9	277904.3	264251.9	150907.5	1	1	1	1	858	5.26	2.01	1	2.9	0.004	CC2D1B	High	Peak Found
2601253202356670000	P31937	1172849	1233287	1172849	669698.9	13	3	5	3	336	8.13	2.29	3	11.485	0	HIBADH	High	High
-3197858597980690000	Q9HA64	1170509	1230452	1170509	668159.6	16	4	5	4	309	7.33	4.13	4	11.722	0	FN3KRP	High	Peak Found

8260092317082080000	Q13362	5945724	6250079	5945724	3393914	8	4	10	2	524	6.87	1.89	4	9.829	0	PPP2R5C	High	High
2758380383998060000	Q86V81	3592929	3774321	3592929	2049530	21	4	13	4	257	11.15	22.31	4	16.625	0	ALYREF	High	High
6087199310060450000	Q9UI30	1449782	1522931	1449782	826981.1	22	2	7	2	125	5.26	11.9	2	11.831	0	TRMT112	High	High
4298785526974990000	Q9BTU6	700126.8	735395.8	700126.8	399334.2	4	2	4	2	479	8.29	1.68	2	6.86	0	PI4K2A	High	High
-8782574511401720000	Q06124	2433770	2555309	2433770	1387583	8	4	8	4	593	7.3	6.58	4	13.879	0	PTPN11	High	High
2867565850229160000	P12081	16387890	17206235	16387890	9343320	23	13	34	10	509	5.88	36.14	13	50.399	0	HARS	High	High
-7667668452715930000	O60264	9393842	9859555	9393842	5353930	12	13	22	11	1052	8.09	23.91	13	38.988	0	SMARCA5	High	High

6550652705279000000	P50552	6557638	6882319	6557638	3737233	18	6	14	6	380	8.94	26.74	6	30.43	0	VASP	High	High
-291186294764485000	P33993	14021063	14712879	14021063	7989379	31	17	37	17	719	6.46	42.06	17	61.423	0	MCM7	High	High
3175822401937940000	Q15942	6448163	6754195	6448163	3667659	16	7	17	7	572	6.67	11.99	7	22.323	0	ZYX	High	High
123318016964233000	O75489	2879619	3015997	2879619	1637745	18	4	11	4	264	7.5	14.3	4	14.761	0	NDUFS3	High	High
-765021547925747000	P49366	1134974	1188272	1134974	645255	8	2	5	2	369	5.36	6.15	2	9.236	0	DHPS	High	High
4377208032786870000	P51858	11957671	12496652	11957671	6785925	38	8	23	7	240	4.73	22.35	8	24.101	0	HDGF	High	High
1505271825198650000	P41236	572512.5	598317.2	572512.5	324897.9	12	2	2	2	205	4.74	1.93	2	6.882	0	PPP1R2	High	Peak Found
-1398033593130890000	O43264	3328683	3474520	3328683	1886732	2	2	5	2	779	6.27	1.6	2	5.234	0	ZW10	High	High

2100681429736930000	O95218	957672.1	999448.8	957672.1	542720.1	3	1	3	1	330	10.01	1.61	1	2.283	0.008	ZRANB2	High	High
6910924152517680000	P13612	621362.6	648374.5	621362.6	352080	2	2	2	2	1032	6.48	0	2	5	0	ITGA4	High	Peak Found
4778122268276290000	P49406	535287.1	558441.1	535287.1	303244.4	3	1	1	1	292	9.5	2.18	1	2.434	0.007	MRPL19	High	Peak Found
6950244637905260000	Q9Y3B4	2306841	2405760	2306841	1306375	16	2	3	2	125	9.38	4.29	2	7.511	0	SF3B14; SF3B6	High	High
8235165147539180000	Q16666	4921486	5130805	4921486	2786127	12	9	17	9	785	9.28	11.35	9	25.22	0	IFI16	High	High
-4352192512000310000	Q9Y619	435679.1	453693.4	435679.1	246364.3	11	3	4	3	301	9.13	0	3	4.795	0	SLC25A15	High	Peak Found
5005223025872710000	O60524	3932765	4094882	3932765	2223601	5	6	10	6	1076	6.35	7.08	6	16.121	0	NEMF	High	High
122083546465	Q7L2E3	5931881	6172395	5931881	3351730	9	11	26	11	11	8.78	24.03	11	28.875	0	DHX30	High	High

8620000										94								
-7186431258293950000	Q04837	8099911	8428082	8099911	4576613	27	4	12	4	148	9.6	15.7	4	16.131	0	SSBP1	High	High
-5658103532290020000	P49770	1292461	1343936	1292461	729783.5	7	3	4	3	351	6.16	3.79	3	9.17	0	EIF2B2	High	High
8342243932904840000	Q13555	432278.3	449297	432278.3	243977	4	2	6	1	558	7.83	2.58	2	9.286	0		Peak Found	High
-4534228375388640000	P52948	5116943	5318072	5116943	2887817	6	10	17	10	1817	6.4	12.3	10	30.363	0	NUP98	High	High
5656121017139870000	Q01081	4699397	4881530	4699397	2650766	12	3	5	3	240	8.81	8.01	3	9.49	0	U2AF1; LOC102724594; U2AF1L5	High	High
-181287195693513000	Q96EA4	358676.2	372398.5	358676.2	202219.6	2	1	1	1	605	5.47	0	1	3.341	0.002	SPDL1	High	Peak Found
889850864413	Q01130	3079646	3197259	3079646	1736174	12	3	5	3	221	11.85	7.82	3	10.834	0	SRSF2	High	High

2710000																		
2331175161516180000	Q6YN16	4831681	5010839	4831681	2720983	12	4	8	4	418	7.99	9.96	4	11.353	0	HSDL2	High	High
7449371538365950000	P12270	25461734	26405302	25461734	14338592	19	40	60	40	2363	5.02	71.11	40	131.967	0	TPR	High	High
-2245217909650060000	Q14573	3398920	3524400	3398920	1913818	4	10	14	9	2671	6.48	9.96	10	29.911	0	ITPR3	High	High
8889615092674920000	Q04721	670925.9	695688.1	670925.9	377772.2	1	2	3	2	2471	5.14	2.08	2	5.855	0	NOTCH2	High	High
2739049111137790000	P13861	10010234	10379577	10010234	5636312	27	10	21	10	404	5.07	29.02	10	33.289	0	PRKAR2A	High	High
-5457086219515970000	P61086	11007553	11412399	11007553	6197155	37	6	20	6	200	5.44	25.33	6	23.486	0	UBE2K	High	High
410623999248744000	Q6PKG0	6035926	6254313	6035926	3396214	13	12	25	12	1096	8.82	23.18	12	42.124	0	LARP1	High	High

2129054283155620000	Q9NPA0	1220811	1264639	1220811	686723.7	11	2	4	2	242	9.25	1.89	2	5.671	0	EMC7	High	High
-5876331061237710000	Q14247	4289770	4443120	4289770	2412701	19	9	16	9	550	5.4	12.59	9	32.459	0	CTTN	High	High
8751597953454220000	P09496	8901881	9219571	8901881	5006406	15	6	16	6	248	4.51	18.18	6	16.563	0	CLTA	High	High
7501998466441850000	P58215	3065669	3172351	3065669	1722648	11	6	12	6	753	6.86	12.04	6	21.111	0	LOXL3	High	High
-3241628752191740000	Q9Y6W3	441723.8	456965.7	441723.8	248141.3	2	2	2	2	813	7.65	0	2	6.356	0	CAPN7	High	Peak Found
5814198643835650000	O60711	728940	753723.8	728940	409286.7	12	3	5	3	386	6.01	0	3	6.527	0	LPXN	High	High
-6351504497919020000	P51648	7169902	7409776	7169902	4023653	18	8	17	8	485	7.88	13.15	8	26.028	0	ALDH3A2	High	High

5275540959932170000	Q14847	4222375	4359873	4222375	2367496	24	6	11	6	261	7.05	17.61	6	24.967	0	LASP1	High	High
-1734934898581350000	P61457	186378.5	192441.6	186378.5	104499.6	13	1	3	1	104	6.8	0	1	2.513	0.006	PCBD1	High	High
-1016656761563180000	O15127	1172257	1210358	1172257	657248	10	2	6	2	329	6.1	1.74	2	11.123	0	SCAMP2	High	High
5927523368410570000	P78527	3.35E+08	3.46E+08	3.35E+08	1.88E+08	31	127	341	127	4128	7.12	449.19	127	504.055	0	PRKDC	High	High
-5634495844004760	O60907	2441156	2518419	2441156	1367551	4	2	5	2	577	6.55	7.88	2	15.612	0	TBL1X	High	High
1056370544189750000	P28065	1148222	1183298	1148222	642553.7	7	2	3	2	219	5.03	0	2	4.498	0	PSMB9	High	High
-2868911152044450000	Q99570	355839.1	366698.6	355839.1	199124.5	2	2	2	2	1358	7.17	0	2	6.569	0	PIK3R4	High	Peak Found

-268059577032989000	Q9NVJ2	7173507	7391061	7173507	4013490	26	5	11	5	186	8.43	8.83	5	14.294	0	ARL8B	High	High
6548869844035820000	Q9UKM9	8306301	8553128	8306301	4644515	23	7	22	7	306	9.17	35.18	7	32.754	0	RALY	High	High
-3980314117452880000	P62979	59377183	61086367	59377183	33171085	73	13	52	4	156	9.64	66.96	13	67.068	0	RPS27A	High	High
6564521941388930000	P56199	4848770	4988198	4848770	2708688	2	3	4	3	1179	6.29	0	3	5.875	0	ITGA1	High	High
5197563919464370000	Q9BTC0	112399	115598.7	112399	62772.35	0	1	1	1	2240	7.88	0	1	2.095	0.01	DIDO1	High	Peak Found
7482655995969630000	Q9Y237	2622180	2693229	2622180	1462476	21	4	6	4	131	9.77	11.03	4	13.373	0	PIN4	High	High
5503163437977980000	Q92508	443057.2	455030.2	443057.2	247090.3	1	3	4	3	2521	7.47	3.81	3	6.357	0	PIEZO1	High	Peak Found
-263361048088	O75608	4777134	4904883	4777134	2663447	14	3	7	3	230	6.77	3.47	3	8.109	0	LYPLA1	High	High

9660000																		
6104369569183710000	P36915	1207584	1239865	1207584	673270.6	6	4	7	4	607	5.8	7.37	4	9.088	0	GNL1	High	High
6576269532483730000	Q06136	1790028	1837797	1790028	997959.5	14	4	9	4	332	7.12	17.35	4	18.069	0	KDSR	High	High
5083710564544010000	P84103	13158193	13500653	13158193	7331117	27	6	12	6	164	11.65	16.14	6	17.281	0	SRSF3	High	High
-1107540500627020000	Q96EE3	742744.9	761446	742744.9	413480	3	1	4	1	360	8.09	1.8	1	4.152	0	SEH1L	High	High
-5993686438301710000	Q96P11	152545.5	156384.3	152545.5	84919.72	7	2	3	2	429	8.62	0	2	5.328	0	NSUN5	High	High
-3545967028758680000	P36543	3172607	3252133	3172607	1765971	8	2	7	2	226	8	8.04	2	6.859	0	ATP6V1E1	High	High
848518254912	O14776	5121513	5247715	5121513	2849611	7	8	10	8	10	8.65	2.08	8	17.642	0	TCERG1	High	High

1430000										98								
-2311882197343430000	Q9NY61	178276.1	182666.8	178276.1	99191.6	3	1	1	1	560	4.94	0	1	3.028	0.003	AATF	High	Peak Found
-3668121595413890000	Q96D46	2430262	2489921	2430262	1352076	14	6	11	6	503	7.14	10.4	6	20.559	0	NMD3	High	High
-558081350695480000	O43172	1459800	1493678	1459800	811096.3	7	3	5	3	522	7.42	0	3	9.827	0	PRPF4	High	Peak Found
-3530209516211160000	Q5JRA6	5631383	5760960	5631383	3128313	2	3	7	3	1907	4.84	7.93	3	11.674	0	MIA3	High	High
-2661255315618110000	O43493	1390659	1422467	1390659	772427	5	2	3	2	437	5.63	1.78	2	4.569	0		High	High
715968340642640000	Q9H9A6	2770762	2833635	2770762	1538719	10	6	12	6	602	6.43	13.43	6	18.42	0	LRRC40	High	High
-178379203363	Q8N7H5	997246.6	1018978	997246.6	553324.8	6	3	4	3	531	4.63	2.2	3	8.306	0	PAF1	High	Peak Found

4380000																		
1852807762900550000	P28070	7983968	8156543	7983968	4429161	28	6	14	6	264	5.97	14.83	6	32.12	0	PSMB4	High	High
7240944493038270000	Q9Y676	736961.7	752817.6	736961.7	408794.6	11	2	3	2	258	9.38	0	2	4.862	0	MRPS18B	High	Peak Found
5817906340983340000	P61956	3026142	3090466	3026142	1678183	23	2	8	1	95	5.5	12.81	2	14.036	0	SUMO2	High	High
-631898127598295000	P01023	1433325	1462934	1433325	794401.8	1	2	5	2	1474	6.46	6.39	2	6.605	0	A2M	High	High
3284671458686940000	Q14498	6791244	6924873	6791244	3760341	15	7	12	7	530	10.1	10.49	7	22.55	0	RBM39	High	High
7950271419257620000	P30049	3167983	3228005	3167983	1752869	14	2	9	2	168	5.49	16.35	2	9.797	0	ATP5D	High	High
-8500687369034290000	P62820	16931807	17252181	16931807	9368270	40	8	25	3	205	6.21	31.54	8	29.579	0	RAB1A	High	High

-888778230352659000	O14908	1744822	1777667	1744822	965307.7	10	3	3	3	333	6.28	6.6	3	12.208	0	GIPC1	High	Peak Found
8250102899765150000	Q9BVA1	536707.4	546810.1	536707.4	296928.5	47	20	218	1	445	4.89	387.67	20	134.392	0	TUBB2B	High	Peak Found
8628250330126930000	O00170	3565503	3632255	3565503	1972385	23	6	10	6	330	6.29	16.38	6	20.263	0	AIP	High	High
-8950408743700010000	O60645	1157166	1178699	1157166	640056.4	6	4	7	4	745	6.29	3.69	4	11.567	0		High	High
6211818470247110000	Q9BQB6	697628.3	710255.3	697628.3	385682.4	15	2	4	2	163	9.36	3.99	2	9.303	0	VKORC1	High	High
-7626065268017050000	P07996	4938776	5026178	4938776	2729312	8	9	16	9	1170	4.94	12.87	9	28.834	0	THBS1	High	High
-3984777124948270000	O94903	2218065	2256317	2218065	1225224	12	3	10	3	275	7.5	12.21	3	11.387	0	PROSC; PLPBP	High	High

2106052778808330000	P62910	2122282	2158104	2122282	1171892	27	3	7	3	135	11.33	5.7	3	13.039	0	RPL32	High	High
5517999105151400000	Q9NWT1	1311799	1333095	1311799	723896.3	4	2	2	2	392	8.91	0	2	4.08	0	PAK1IP1	High	Peak Found
9211390500631630000	P04156	364595.6	370387.2	364595.6	201127.5	8	2	3	2	253	9	1.63	2	6.715	0	PRNP	High	High
-2725190465399900000	P62829	32220652	32726161	32220652	17770942	39	5	24	5	140	10.51	29.12	5	16.126	0	RPL23	High	High
8454971752249740000	Q9BSD7	3886281	3943723	3886281	2141518	42	6	13	6	190	9.54	23.45	6	30.158	0	NTPCR	High	High
-7691749111498670000	O14531	231150.6	234531.1	231150.6	127354.9	3	1	1	1	572	7.09	1.76	1	2.756	0.004	DPYSL4	High	Peak Found
6144698658960750000	Q8IXB1	3782040	3834912	3782040	2082432	8	7	11	7	793	7.18	5	7	18.228	0	DNAJC10	High	High

-4137720252324400000	O60716	10602518	10750488	10602518	5837724	15	13	26	13	968	6.23	32.91	13	48.602	0	CTNND1	High	High
-3936589147855350000	P54886	42130665	42704985	42130665	23189637	25	19	53	19	795	7.12	88.86	19	87.095	0	ALDH18A1	High	High
-6821815504623320000	Q9UMX0	1143919	1159094	1143919	629410.7	12	4	5	2	589	5.11	6.83	4	13.576	0	UBQLN1	High	High
1516272036315920000	Q15637	3990870	4041384	3990870	2194550	9	5	7	5	639	8.98	7.99	5	22.419	0	SF1	High	High
7546150518504420000	O00186	2585099	2617810	2585099	1421522	8	5	10	5	592	7.8	6.05	5	16.002	0	STXBP3	High	High
-1756895692335450000	O95865	4384798	4439644	4384798	2410813	18	5	11	4	285	6.01	11.54	5	16.964	0	DDAH2	High	High
1112026886534240000	Q99653	1170385	1185015	1170385	643486.1	14	2	6	2	195	5.1	6.31	2	7.847	0	CHP1	High	High

7369433181564390000	P08708	29704358	30061004	29704358	16323710	30	7	20	7	135	9.85	35.92	7	37.789	0	RPS17; RPS17L	High	High
3568941862227660000	Q9NVP2	173313.2	175321.4	173313.2	95202.91	7	1	1	1	202	4.56	2.09	1	3.258	0.002	ASF1B	High	Peak Found
-5864065326781890000	P27708	17582821	17785789	17582821	9658029	13	25	52	24	2225	6.46	49.15	25	83.729	0	CAD	High	High
8869464350434740000	O95372	1227728	1241373	1227728	674089.9	3	1	1	1	231	7.23	1.83	1	2.45	0.007	LYPLA2	High	Peak Found
-5604147819280000000	P50613	293922.7	297154.8	293922.7	161360.8	3	1	1	1	346	8.47	1.87	1	2.947	0.004	CDK7	Peak Found	High
5843152145544880000	Q6KC79	133671.3	134941.6	133671.3	73275.91	0	1	2	1	2804	7.91	0	1	2.697	0.005	NIPBL	High	High
-3137341147702060000	P26440	1970189	1988797	1970189	1079956	9	4	7	4	426	8.05	8.33	4	11.424	0		High	High

-5631999146533420000	Q02878	10848217	10940300	10848217	5940795	20	6	17	6	288	10.58	17.61	6	23.369	0	RPL6	High	High
5656717572997730000	P30566	4558908	4597432	4558908	2496495	7	3	9	3	484	7.11	18.07	3	13.374	0	ADSL	High	High
1650974207865810000	P04818	2761280	2782755	2761280	1511090	22	5	13	5	313	7.01	12.18	5	21.343	0	TYMS	High	High
114843226561293000	P67936	90422834	91123451	90422834	49481805	60	22	56	11	248	4.69	100.46	22	115.471	0	TPM4	High	High
-8024352083789970000	Q9UKV3	409521.2	412650.5	409521.2	224077.3	1	2	2	2	1341	6.43	3.78	2	6.377	0	ACIN1	High	Peak Found
108491433269500000	Q9Y5X1	4631515	4664083	4631515	2532688	13	7	15	7	595	5.58	14.09	7	21.177	0	SNX9	High	High
556799029181218000	Q13596	2126350	2140708	2126350	1162446	15	7	13	6	522	5.15	18.55	7	29.046	0	SNX1	High	High
2002509444900540000	Q9BTY2	1022312	1028646	1022312	558574.8	5	2	3	2	467	6.25	1.8	2	5.308	0	FUCA2	High	High

-3489297846321020000	O75306	1627376	1636577	1627376	888693.4	12	5	9	5	463	7.55	1.91	5	13.238	0	NDUFS2	High	High
3497199974885210000	Q15070	1008791	1014299	1008791	550784.1	5	2	2	2	435	9.45	2.06	2	6.741	0	OXA1L	High	Peak Found
-8985233522455600000	P61758	2283151	2294193	2283151	1245792	13	3	7	3	197	7.11	3.74	3	9.218	0	VBP1	High	High
-4972524321603700000	Q8IW35	534891.8	537464.5	534891.8	291853.7	2	2	2	2	865	5.02	0	2	4.867	0	CEP97	High	Peak Found
7437759637941570000	Q92597	18171129	18255518	18171129	9913101	35	8	23	8	394	5.82	46.92	8	86.358	0	NDRG1	High	High
4890762250346220000	P35250	4109040	4127470	4109040	2241297	15	6	8	6	354	6.44	5.33	6	17.163	0	RFC2	High	High
-7428789248326480000	O00712	2319363	2329435	2319363	1264928	5	2	4	2	420	8.87	2.02	2	4.829	0	NFIB	High	High

1361543221458190000	O60547	1620764	1626966	1620764	883474.2	13	4	5	4	372	7.31	0	4	13.196	0	GMDS	High	High
-2715683272122760000	Q14197	703421.4	706112.3	703421.4	383432.7	11	2	3	2	206	10.07	5.03	2	9.445	0	ICT1; MRPL58	High	High
3771039295331550000	P12694	1226786	1230902	1226786	668403.8	11	4	6	4	445	8.27	5	4	18.739	0	BCKDHA	High	High
8273145356875310000	Q9BXI6	653045.5	655212.5	653045.5	355793.1	7	3	8	3	508	8.44	0	3	7.071	0	TBC1D10A	High	High
-7202922108567290000	Q8IZP0	1577055	1581646	1577055	858864.7	5	2	5	2	508	7.06	3.58	2	6.687	0	ABI1	High	High
-795610127174802000	P15311	46113600	46179439	46113600	25076333	39	28	75	17	586	6.27	107.05	28	122.045	0	EZR	High	High
-2205363031532400000	Q16763	836525.1	837184.1	836525.1	454607.3	14	3	5	3	222	8.38	3.58	3	6.398	0	UBE2S	High	High

-8242626749563960000	Q9Y606	349179.2	349183.8	349179.2	189613.6	2	1	1	1	427	8.41	1.87	1	3.298	0.002	PUS1	High	Peak Found
3338073365584810000	P83731	33888829	33886458	33888829	18401005	36	7	19	7	157	11.25	26.12	7	28.797	0	RPL24	High	High
6856624260157840000	O60256	3174030	3172894	3174030	1722943	15	5	8	5	369	7.44	3.88	5	19.059	0	PRPSAP2	High	High
-749299575230837000	Q9HAT2	308346.5	308145.4	308346.5	167329	2	1	1	1	523	7.33	2.07	1	3.521	0.002	SIAE	High	Peak Found
-5958552957432290000	Q9BRJ6	188148.5	187992	188148.5	102083.3	11	1	2	1	194	9.64	0	1	2.74	0.005	C7orf50	High	High
8719462215085100000	Q8N983	456332.8	455928.1	456332.8	247577.8	8	2	3	2	215	8.65	1.67	2	5.16	0	MRPL43	High	High
1723896581553540000	Q07666	26444538	26401184	26444538	14336356	23	9	22	8	443	8.66	35.16	9	41.724	0	KHDRBS1	High	High

-5097662091813910000	Q02252	1361394	1358851	1361394	737882.7	5	3	10	3	535	8.5	7.27	3	7.386	0	ALDH6A1	High	High
5046480723636320000	Q9Y314	405762.2	404994.7	405762.2	219920	12	3	4	3	301	8.82	1.67	3	6.448	0	NOSIP	High	Peak Found
-3090947774688950000	P43005	933100.6	931205.2	933100.6	505662.5	5	2	4	2	524	5.71	2.43	2	8.343	0	SLC1A1	High	High
6049594169438390000	Q52LJ0	1968915	1963720	1968915	1066338	15	5	9	3	433	8.69	8.86	5	23.571	0		High	High
1785172786280040000	P06730	5100491	5084139	5100491	2760786	12	3	6	3	217	6.15	5.68	3	9.683	0	EIF4E	High	High
-8745389805375580000	P24539	20351575	20283488	20351575	11014328	32	9	23	9	256	9.36	47.69	9	58.078	0	ATP5F1	High	High
-8293533738236190000	O75347	5670514	5649608	5670514	3067847	55	7	17	7	108	5.29	28.32	7	34.836	0	TBCA	High	High

-2939496649029220000	P51812	1456482	1450848	1456482	787838.6	5	3	4	3	740	6.89	3.6	3	7.959	0	RPS6KA3	High	Peak Found
5271819707192530000	Q9H7E9	507196.9	505142.4	507196.9	274302.1	7	1	2	1	229	9.95	4.98	1	6.178	0	C8orf33	High	High
-7674938862773320000	A3KMH1	1157529	1152696	1157529	625936.5	2	4	7	4	1905	7.4	1.65	4	9.841	0	VWA8	High	High
5883384996074350000	Q8TD08	3103583	3089378	3103583	1677592	1	1	3	1	544	8.97	1.8	1	2.537	0.006	MAPK15	High	High
5950688437789860000	O95716	382683.8	380912.2	382683.8	206842.7	15	4	7	1	219	4.93	3.52	4	9.619	0	RAB3D	High	Peak Found
5836622772794950000	P06400	1338635	1332204	1338635	723412.5	3	2	4	2	928	7.94	4.17	2	8.216	0	RB1	High	High
4208729749156870000	O60678	403531.4	401144.5	403531.4	217829.3	2	1	1	1	531	5.35	0	1	3.223	0.002	PRMT3	High	Peak Found

-4865533649162520000	P28702	755036.8	750355.5	755036.8	407457.6	3	2	3	2	533	8.18	0	2	6.461	0	RXRB	High	Peak Found
-4872825174478960000	P57088	4619367	4589949	4619367	2492431	21	5	9	5	247	9.7	9.17	5	13.946	0	TMEM33	High	High
-1437686464987710000	Q9BUF5	13432818	13342808	13432818	7245404	40	18	172	7	446	4.88	248.07	18	115.196	0	TUBB6	High	High
5021225223718000000	P31949	25602179	25409371	25602179	13797782	41	6	20	6	105	7.12	38.48	6	24.562	0	S100A11	High	High
1798322026652800000	Q5VTU8	3131722	3107473	3131722	1687418	31	2	6	2	51	10.14	7.02	2	4.344	0	ATP5EP2	High	High
-701884836930743000	O96033	544910.1	540491.6	544910.1	293497.5	13	1	1	1	88	4.72	1.84	1	3.556	0.002	MOCS2	High	Peak Found
7953357218888960000	Q3KQU3	2856102	2832592	2856102	1538153	6	4	6	4	841	10.11	3.89	4	9.231	0	MAP7D1	High	High

-8894400129343680000	Q9BUH6	220614.4	218783.1	220614.4	118803.5	4	1	1	1	204	5.48	1.85	1	2.826	0.004	C9orf142; PAXX	High	Peak Found
4583007171926660000	Q9H1I8	393780.4	390508.7	393780.4	212053.8	1	1	1	1	757	5.16	0	1	2.41	0.008	ASCC2	High	Peak Found
-4617078502424800000	Q9H857	6852517	6794340	6852517	3689459	15	9	13	9	520	6.77	10.42	9	22.512	0	NT5DC2	High	High
968979890096343000	P53611	1793106	1777089	1793106	964993.8	7	2	4	2	331	5.03	8.64	2	10.909	0	RABGGTB	High	High
-3143517004493310000	Q01970	2243574	2222703	2243574	1206971	6	7	15	7	1234	5.9	9.21	7	20.088	0	PLCB3	High	High
-7527974897574340000	Q16778	42873707	42458394	42873707	23055734	36	5	14	2	126	10.32	18.2	5	23.364	0	HIST2H2BE	High	High
7232907791819210000	Q9Y5Z4	1478121	1463652	1478121	794791.3	16	3	6	3	205	4.63	7.89	3	9.914	0	HEBP2	High	High

-4837145647589030000	P46937	466521.2	461923.3	466521.2	250833.3	5	1	2	1	504	5.17	0	1	2.684	0.005	YAP1	High	High
-4264296356835160000	Q96ND0	496205.3	491283.6	496205.3	266776.5	6	1	3	1	272	9.76	0	1	4.526	0	FAM210A	High	High
7299810347757170000	Q03135	12540261	12411749	12540261	6739821	29	5	14	5	178	6.02	25.7	5	26.738	0	CAV1	High	High
8767729618822810000	O14933	581125.4	575102.6	581125.4	312291.9	5	1	1	1	153	7.88	1.81	1	2.438	0.007	UBE2L6	Peak Found	High
3538184499835990000	P43897	1330934	1317060	1330934	715189.3	13	3	3	3	325	8.38	2.05	3	7.016	0	TSFM	High	High
-2817610694198920000	P62841	11924389	11798579	11924389	6406858	31	4	19	4	145	10.39	27.87	4	41.023	0	RPS15	High	High
8876703773954470000	O43759	3087787	3053238	3087787	1657968	10	2	5	2	233	4.68	8.3	2	7.652	0	SYNGR1	High	High

7099090004337320000	P63167	5068401	5011187	5068401	2721172	46	3	7	3	89	7.4	12.53	3	17.983	0	DYNLL1	High	High
-1644148839842950000	Q9BRK3	1556985	1538871	1556985	835636.8	10	4	5	4	442	7.23	4.01	4	12.759	0	MXRA8	High	Peak Found
-256091368978195000	Q32MZ4	5553525	5486526	5553525	2979290	13	7	11	6	808	4.65	20.55	7	40.652	0	LRRFIP1	High	High
-6114421503582250000	P61619	35512573	35048857	35512573	19032211	11	6	19	6	476	8.06	34.53	6	23.508	0	SEC61A1	High	High
-7190222159830220000	Q9H0A0	4741254	4678425	4741254	2540476	11	11	22	11	1025	8.27	17.28	11	32.157	0	NAT10	High	High
-1877789849069090000	Q9UIW2	6724617	6634968	6724617	3602917	3	6	11	6	1896	6.92	3.62	6	14.953	0	PLXNA1	High	High
-236860523614	Q96PB1	11863233	11697682	11863233	6352069	1	1	2	1	797	8.82	1.66	1	2.105	0.01	CASD1	High	High

8650000																		
4137218576109140000	Q06210	38976678	38429121	38976678	20867760	33	18	40	16	699	7.11	66.52	18	107.232	0	GFPT1	High	High
2824475933773920000	Q14108	15160612	14940312	15160612	8112880	14	8	20	8	478	5.14	34.84	8	35.885	0	SCARB2	High	High
-1633285367868470000	P35241	8940704	8810068	8940704	4784038	32	23	61	10	583	6.37	77.49	23	90.383	0	RDX	High	High
7752798674986430000	Q5T4S7	25676170	25296277	25676170	13736370	7	29	51	29	5183	6.04	38.59	29	94.915	0	UBR4	High	High
4024286234021820000	P78406	3973484	3914228	3973484	2125502	23	7	16	7	368	7.83	10.39	7	23.274	0	RAE1	High	High
6939588679820240000	P23193	9924298	9776105	9924298	5308615	28	9	19	9	301	8.38	27.82	9	36.584	0	TCEA1	High	High
6379053334718360000	O95340	7193577	7082524	7193577	3845948	19	11	22	11	614	8.03	26.53	11	35.74	0	PAPSS2	High	High

-8150015609920890000	P58107	25722454	25314424	25722454	13746225	9	8	16	2	5088	5.62	16.57	8	28.913	0		Peak Found	High
-6073182747863780000	Q9ULT8	1603951	1578144	1603951	856962.7	2	5	7	5	2610	5.35	2.3	5	11.944	0	HECTD1	High	High
-6337407282914380000	P48960	3878434	3815831	3878434	2072070	11	8	13	8	835	6.87	13.54	8	37.847	0	CD97; ADGRE5	High	High
3913128170310310000	O00487	37940365	37323144	37940365	20267193	24	6	18	6	310	6.52	19.84	6	21.934	0	PSMD14	High	High
8757061134285980000	Q13586	3259392	3205750	3259392	1740785	4	3	4	3	685	6.67	5.63	3	9.263	0	STIM1	High	High
3290991412812470000	Q5T9A4	1146705	1127616	1146705	612317.6	22	14	38	1	648	9.2	38.79	14	47.576	0	ATAD3B	High	High
-4630001761870020000	P34897	37185958	36565362	37185958	19855703	34	16	51	15	504	8.53	77.41	16	63.379	0	SHMT2	High	High

7322766248926000000	Q13564	1662559	1634480	1662559	887554.4	2	1	1	1	534	5.4	1.94	1	3.02	0.003	NAE1	High	Peak Found
4947084340596130000	Q8TF05	2220406	2182530	2220406	1185156	4	4	7	4	950	4.77	1.85	4	12.644	0	PPP4R1	High	High
-3936479494832140000	Q8TCS8	3653571	3590748	3653571	1949846	8	7	14	7	783	7.77	13.03	7	19.915	0	PNPT1	High	High
-3970443704612930000	P36578	37306284	36643746	37306284	19898267	41	19	46	19	427	11.06	71.17	19	70.955	0	RPL4	High	High
4118942694403510000	Q13492	10643239	10451430	10643239	5675330	13	8	13	8	652	7.9	14.83	8	33.82	0	PICALM	High	High
-9125415552757100000	Q05639	1256114	1233164	1256114	669631.9	34	14	113	2	463	9.03	226.1	14	93.204	0	EEF1A2	High	High
-145222249138625000	Q9BYG3	622653.3	611226.4	622653.3	331907.8	5	1	3	1	293	9.88	4.76	1	6.918	0	MKI67IP; NIFK	High	High

6076863243069160000	Q8WX93	13389803	13129071	13389803	7129341	16	18	35	18	1383	7.09	38.43	18	61.088	0	PALLD	High	High
-8574193745658390000	P46926	14287739	14005558	14287739	7605290	30	7	16	7	289	6.92	16.07	7	24.109	0	GNPDA1	High	High
-4539117867176900000	P50402	7168887	7026657	7168887	3815611	40	8	17	8	254	5.5	13.14	8	38.957	0	EMD	High	High
-4078763853778870000	O95816	1995623	1956017	1995623	1062155	25	5	7	5	211	6.7	7.24	5	21.587	0	BAG2	High	High
-2222820814571890000	Q9UJ70	9009464	8828569	9009464	4794084	28	9	19	9	344	6.24	14.5	9	34.485	0	NAGK	High	High
4892501601992700000	Q5K651	1931389	1891371	1931389	1027051	4	7	10	7	1589	7.83	1.96	7	19.121	0	SAMD9	High	High
3010766793389340000	P0DP25	18480711	18090954	18480711	9823740	48	9	27	8	149	4.22	41.52	9	41.741	0	CALM3; CALM2; CALM1	High	High

-2088552440920440000	Q9Y570	7832783	7666676	7832783	4163154	19	8	15	8	386	5.97	15.69	8	22.301	0	PPME1	High	High
4254593612476960000	Q9BTZ2	2317617	2268142	2317617	1231645	15	5	10	5	278	8.56	8.35	5	16.973	0	DHRS4	High	High
5898955459380030000	Q05048	3969626	3884769	3969626	2109505	13	5	13	5	431	6.58	3.54	5	21.106	0	CSTF1	High	High
8845776874603620000	Q92930	1055749	1032928	1055749	560899.7	41	9	23	3	207	9.07	20.33	9	28.869	0	RAB8B	High	High
-3934264021141740000	Q9UI12	1551114	1516169	1551114	823309	8	3	5	3	483	6.48	3.79	3	6.28	0	ATP6V1H	High	High
-8034699390765470000	Q9UID3	697243.9	681401.3	697243.9	370014.2	4	3	6	3	782	6.47	2.79	3	10.216	0	VPS51	High	High
4173136633731680000	P29590	4569178	4464390	4569178	2424250	6	5	11	5	882	6.21	10.49	5	15.485	0	PML	High	High

1449875230825110000	Q03252	10001049	9769551	10001049	5305056	9	5	8	4	620	5.59	5.06	5	14.74	0	LMNB2	High	High
8871370198028650000	P62995	3336978	3259065	3336978	1769735	6	2	4	2	288	11.25	6	2	5.041	0	TRA2B	High	High
-9009655612245830000	Q9Y4G6	1062589	1037660	1062589	563469.8	6	16	34	4	2542	5.57	27.43	16	63.58	0	TLN2	High	High
7321724515391940000	Q8WUY8	12427892	12131472	12427892	6587625	4	2	5	2	206	10.74	2.19	2	4.22	0	NAT14	High	High
3248682762130000000	Q9BQ39	1706933	1666175	1706933	904765.6	8	5	12	3	737	9.17	15.17	5	19.72	0	DDX50	High	High
2315597211980710000	Q9BRQ8	1208605	1179681	1208605	640590	7	2	2	2	373	9.11	5.31	2	11.05	0	AIFM2	High	Peak Found
-2307788867192090000	Q9UBI6	3637485	3550263	3637485	1927862	35	2	9	2	72	8.97	9.64	2	10.151	0	GNG12	High	High

5314014285931230000	P18124	50414927	49188285	50414927	26710195	41	13	41	13	248	10.65	60.63	13	56.872	0	RPL7	High	High
2160825122742080000	Q13162	15671958	15284366	15671958	8299708	36	9	33	6	271	6.29	49.48	9	47.792	0	PRDX4	High	High
2420827179212210000	P30622	1837748	1789778	1837748	971884.2	5	6	8	6	1438	5.36	3.53	6	15.573	0	CLIP1	High	High
2869240267963350000	Q9UQB8	281271	273855.3	281271	148708.8	4	2	3	2	552	8.9	1.68	2	6.559	0	BAIAP2	High	High
8364348392468160000	P04040	9090738	8850455	9090738	4805969	25	11	21	11	527	7.39	29.69	11	47.226	0	CAT	High	High
-4130668541852850000	Q9BTV4	7028901	6843039	7028901	3715903	21	8	13	8	400	8.13	10.11	8	21.061	0	TMEM43	High	High
7969393992682690000	Q9NUJ1	1031629	1003939	1031629	545158.4	8	2	4	2	306	8.57	2.15	2	5.73	0	ABHD10	High	High
-892609892924	Q9NR28	3375705	3284539	3375705	1783568	12	3	6	3	239	5.9	8.78	3	12.493	0	DIABLO	High	High

9150000																		
6200383293614890000	Q9UK45	559870	544424.1	559870	295632.8	8	1	3	1	103	5.27	1.69	1	2.748	0.004	LSM7	High	High
-6438842107367460000	P11498	2.75E+08	2.67E+08	2.75E+08	1.45E+08	41	39	146	39	1178	6.84	194.38	39	184.746	0	PC	High	High
4893836570063620000	O75494	640886.9	622790.3	640886.9	338187.2	8	2	2	2	262	11.27	1.92	2	6.925	0	SRSF10; LOC100996657	High	Peak Found
562653879591220000	P09488	4705584	4571111	4705584	2482202	22	5	9	3	218	6.7	5.6	5	15.339	0	GSTM1	High	High
2170625950346530000	O75396	30973711	30087685	30973711	16338198	39	10	27	10	215	6.92	43.62	10	49.152	0	SEC22B	High	High
2409289599634590000	P42357	20215204	19630201	20215204	10659581	3	2	4	2	657	6.95	3.64	2	6.994	0	HAL	High	High
-6920554993800990000	Q02880	1599624	1553262	1599624	843451.2	5	9	15	6	1626	8	5.76	9	20.728	0	TOP2B	High	High

-1796554414882970000	Q8TCG1	6740869	6541376	6740869	3552094	11	11	20	11	905	6.23	10.4	11	27.918	0	KIAA1524; CIP2A	High	High
6043463436452300000	Q5SSJ5	15159313	14708585	15159313	7987048	15	9	23	9	553	9.67	33.1	9	26.623	0	HP1BP3	High	High
3335218336630150000	Q9UFN0	1140557	1106405	1140557	600799.6	11	3	6	3	247	9.16	0	3	8.064	0	NIPSNAP3A	High	High
-7181464862980120000	Q13630	11644816	11287248	11644816	6129195	29	7	12	7	321	6.6	22.12	7	30.029	0	TSTA3	High	High
7576876112341400000	O43813	4398848	4260612	4398848	2313595	9	4	6	4	399	7.75	8.2	4	12.102	0	LANCL1	High	High
8388783144784420000	P82930	1089364	1054761	1089364	572755.7	13	3	5	3	218	9.98	2.65	3	10.204	0	MRPS34	High	High
-15854148761745100	Q6IN85	1483803	1436594	1483803	780098.6	5	4	8	2	833	4.94	2.56	4	12.25	0	SMEK1; PPP4R3A	High	High

4463882674882170000	Q5NDL2	180780.7	174923.4	180780.7	94986.8	2	1	2	1	527	7.05	1.64	1	3.267	0.002	EOGT	High	High
4240907950946080000	Q9BV44	1669079	1614674	1669079	876799.6	6	3	7	3	507	6.37	1.65	3	8.285	0	THUMPD3	High	High
4406338825216400000	Q16740	2328017	2251913	2328017	1222833	9	2	3	2	277	8.09	2.27	2	7.196	0	CLPP	High	High
-5945011317712510000	P25787	12201208	11797012	12201208	6406007	31	6	15	6	234	7.43	25.07	6	28.498	0	PSMA2	High	High
8976168612742670000	Q13243	2182463	2110052	2182463	1145799	15	4	9	3	272	11.59	11.92	4	19.493	0	SRSF5	High	High
-1176127761844360000	Q9H2G2	3335955	3225149	3335955	1751319	3	4	9	3	1235	5.15	8.03	4	13.776	0	SLK	High	High
-3003909500726810000	O60443	378571.7	365703.2	378571.7	198583.9	4	2	2	2	496	5.17	0	2	4.369	0	DFNA5; GSDME	High	Peak Found

5561612098835550000	P38646	1.24E+08	1.2E+08	1.24E+08	65128528	46	30	76	30	679	6.16	130.71	30	159.663	0	HSPA9	High	High
7862445690525930000	P32455	5756322	5555133	5756322	3016545	6	4	6	4	592	6.32	3.07	4	11.372	0	GBP1	High	High
4962531905740650000	Q99715	2535275	2446456	2535275	1328473	4	10	14	10	3063	5.53	3.78	10	27.386	0	COL12A1	High	High
-570604753130224000	O15438	249251.7	240236.1	249251.7	130452.9	1	1	1	1	1527	7.2	0	1	2.224	0.009	ABCC3	High	Peak Found
-5973761010667610000	Q86Y07	189538.2	182682.3	189538.2	99200.03	2	1	1	1	508	8.84	0	1	2.826	0.004	VRK2	High	Peak Found
-476306151852591000	Q15102	7102201	6844250	7102201	3716561	26	7	16	7	231	6.84	11.48	7	20.742	0	PAFAH1B3	High	High
-8309198551146020000	O15226	235981.8	227397.3	235981.8	123481.2	2	2	4	2	690	8.79	0	2	3.845	0.001	NKRF	High	High

5625477449042210000	P04179	5106033	4918012	5106033	2670576	16	4	6	4	222	8.25	11.59	4	14.307	0		High	High
-4838568104168170000	Q8TBC4	2542617	2448703	2542617	1329693	14	5	10	5	463	5.45	10.71	5	20.772	0	UBA3	High	High
-1270194887050030000	Q9UBW8	2498449	2405976	2498449	1306492	16	3	4	3	275	8.22	2.58	3	12.622	0	COPS7A	High	High
-4031307080017590000	P18077	38339134	36919887	38339134	20048216	33	5	20	5	110	11.06	29.32	5	17.889	0	RPL35A	High	High
-4178938987355290000	P62312	1703352	1640057	1703352	890582.8	19	2	3	2	80	9.58	0	2	3.334	0.002	LSM6	High	High
208143562843118000	Q8NFH5	368181	354309.7	368181	192397.1	10	2	2	2	326	9.09	0	2	4.138	0	NUP35	High	Peak Found
-1329067276742600000	Q8WYP5	443287	426521.8	443287	231609.6	1	2	4	2	2266	6.6	1.91	2	5.156	0	AHCTF1	High	High

-6803105277320920000	Q99848	2305392	2217460	2305392	1204124	11	4	5	4	306	10.1	5.81	4	9.651	0	EBNA1BP2	High	High
6641856841102680000	P62826	68412164	65777110	68412164	35718250	30	9	36	9	216	7.49	45.13	9	44.661	0	RAN	High	High
8349989266333900000	P49841	3370169	3239379	3370169	1759046	16	6	8	3	420	8.78	5.22	6	20.536	0	GSK3B	High	Peak Found
-9082551203190850000	Q16777	48927558	47021591	48927558	25533638	21	4	18	2	129	10.9	20.08	4	12.871	0	HIST2H2AC	High	High
-5200719085873830000	Q8NFW8	410548.5	394371.7	410548.5	214151.5	3	1	2	1	434	7.93	3.87	1	3.924	0	CMAS	High	High
-4388967430464990000	P43034	10388365	9977258	10388365	5417845	24	8	19	8	410	7.37	19.58	8	29.108	0	PAFAH1B1	High	High
6102726942936210000	Q14152	65299480	62677718	65299480	34035219	28	38	82	38	1382	6.79	115.67	38	136.314	0	EIF3A	High	High

-6225000128541680000	Q99836	159722.9	153244.3	159722.9	83214.61	2	1	1	1	296	6.15	0	1	2.671	0.005	MYD88	High	Peak Found
-7261913870359170000	O75165	4572667	4387000	4572667	2382226	7	14	20	14	2243	6.74	14.71	14	52.816	0	DNAJC13	High	High
-4714570572578110000	Q86YS6	242400.9	232542.3	242400.9	126275	10	2	2	1	212	5.64	0	2	5.665	0	RAB43	High	Peak Found
-200305812606160000	Q3MHD2	866311.4	830470.6	866311.4	450961.7	11	2	4	2	195	7.74	7.3	2	9.776	0	LSM12; LSM12P1	High	High
3602347345364680000	P09429	54414756	52161731	54414756	28324834	54	16	36	13	215	5.74	55.15	16	85.004	0	HMGB1	High	High
2201882454177380000	P63241	1.02E+08	97901341	1.02E+08	53162332	41	12	44	12	154	5.24	105.7	12	93.687	0	EIF5A	High	High
-4267649636453150000	P16949	14630345	14024201	14630345	7615414	42	9	23	8	149	5.97	34.72	9	24.416	0	STMN1	High	High

-8781078747776300000	Q9NY12	1926853	1845621	1926853	1002208	24	5	10	5	217	10.92	3.62	5	11.042	0	GAR1	High	High
5521000316515270000	Q9UKM7	1359937	1302266	1359937	707156	2	1	3	1	699	7.72	4.03	1	7.817	0	MAN1B1	High	High
2476357386497250000	Q07617	199395.7	190935.9	199395.7	103681.9	1	1	2	1	926	6.86	0	1	2.796	0.004	SPAG1	High	Peak Found
-651184813655133000	P13807	6030351	5774052	6030351	3135422	10	7	10	6	737	6.18	16.22	7	23.623	0	GYS1	High	High
7656229856008540000	O95453	1649975	1579527	1649975	857714	5	3	4	3	639	6.2	3.6	3	8.937	0	PARN	High	High
-1992477291119830000	Q7Z388	505869.2	484089.5	505869.2	262870	2	2	2	2	723	8.16	1.7	2	4.225	0	DPY19L4	High	Peak Found
-2946380844768100000	Q96I24	5373477	5138308	5373477	2790201	28	14	26	11	572	8.38	22.62	14	39.879	0	FUBP3	High	High

6639217336905110000	Q8N5K1	1712375	1636370	1712375	888580.8	22	3	4	3	135	9.61	4.2	3	10.506	0	CISD2	High	High
-3704891385856810000	Q9UKX5	3075519	2938864	3075519	1595860	5	7	11	7	1188	6.7	9.45	7	15.84	0	ITGA11	High	High
-4626058579617190000	Q5VYS8	119645.5	114254.5	119645.5	62042.4	1	1	1	1	1495	6.83	0	1	3.547	0.002	ZCCHC6	High	Peak Found
-5085755615855730000	P50895	2906795	2775073	2906795	1506918	14	7	7	7	628	5.81	7.89	7	18.66	0	BCAM	High	High
-7317656068237780000	Q15836	8770349	8372367	8770349	4546358	50	6	13	6	100	8.79	21.95	6	30.369	0	VAMP3	High	High
271678290460553000	P43307	8792971	8393585	8792971	4557880	12	2	4	2	286	4.49	5.31	2	12.33	0	SSR1	High	High
-2007854671863600000	P46734	1308428	1248962	1308428	678210.8	7	3	4	3	347	7.43	6.12	3	7.37	0	MAP2K3	High	Peak Found

-6064539384067370000	Q9UJX3	758199.4	723350.1	758199.4	392793.1	3	2	2	2	599	5.64	0	2	4.387	0	ANAPC7	High	Peak Found
-3951454542439150000	Q9HB40	4844309	4616278	4844309	2506729	11	5	13	5	452	5.81	18.09	5	15.442	0	SCPEP1	High	High
-9023891521044100000	Q96BX8	592398.5	564486.5	592398.5	306527.2	4	1	2	1	217	8.63	0	1	2.14	0.01	MOB3A	High	High
6094529598036820000	Q13442	2444484	2327641	2444484	1263955	6	2	5	2	181	8.87	10.46	2	9.706	0	PDAP1	High	High
1530625692360910000	Q8IWB7	614601.7	584435	614601.7	317359.6	10	4	5	4	410	7.33	2	4	10.296	0	WDFY1	High	Peak Found
7890676929847470000	Q6NZI2	66754607	63460494	66754607	34460282	32	12	43	12	390	5.6	63.21	12	71.93	0	PTRF; CAVIN1	High	High
769140260772308000	P07910	56039002	53272775	56039002	28928153	34	14	47	13	306	5.08	67.9	14	75.225	0	HNRNPC	High	High

2951553745667670000	P31689	10779986	10239337	10779986	5560159	29	9	18	9	397	7.08	18.97	9	52.603	0	DNAJA1	High	High
4819084498940790000	Q6ZVM7	401834.9	381470.1	401834.9	207145.7	1	1	1	1	507	4.79	0	1	2.159	0.009	TOM1L2	High	Peak Found
8312610725791650000	P17812	25320398	24026436	25320398	13046822	37	20	46	18	591	6.46	76.44	20	76.21	0	CTPS1	High	High
6480137070562710000	P07437	5.74E+08	5.45E+08	5.74E+08	2.96E+08	59	26	274	4	444	4.89	485.97	26	174.279	0	TUBB	High	High
-2373737580190420000	Q9BW60	2371892	2249463	2371892	1221502	9	2	5	2	279	9.6	10.58	2	10.679	0	ELOVL1	High	High
-8282495750123490000	Q96AE4	29203583	27692327	29203583	15037472	42	23	51	18	644	7.61	63.74	23	83.553	0	FUBP1	High	High
-485685737815851000	Q6EMK4	1132169	1073338	1132169	582843.5	6	3	5	3	673	7.39	5.64	3	9.799	0	VASN	High	High
888482384241	Q9BZF9	7071316	6701527	7071316	3639060	9	12	19	12	14	7.03	12.51	12	38.118	0	UACA	High	High

5200000										16								
4145241579063420000	P68400	4675253	4430077	4675253	2405618	17	5	10	5	391	7.74	14.91	5	19.429	0	CSNK2A1	High	High
5912307548831860000	P25398	31540564	29876024	31540564	16223262	57	7	20	7	132	7.21	28.16	7	32.989	0	RPS12	High	High
-8207864549745430000	Q96T76	1207400	1143247	1207400	620805.6	3	3	4	3	1030	6.35	1.73	3	7.6	0	MMS19	High	High
6844923263172620000	Q9BXP5	12554510	11885377	12554510	6453991	13	13	26	13	876	5.96	20.63	13	36.761	0	SRRT	High	High
-1367534402013740000	O75947	15712897	14872857	15712897	8076250	28	5	14	5	161	5.3	20.1	5	20.015	0	ATP5H	High	High
-1630432664316480000	Q16563	5745181	5433892	5745181	2950709	4	1	6	1	259	8.43	10.39	1	3.615	0.001	SYPL1	High	High
-538121778148	Q99536	21003328	19860722	21003328	10784758	28	10	29	10	393	6.29	40.88	10	51.476	0	VAT1	High	High

7520000																		
738970082796446000	Q8N5M9	550117.4	520176.4	550117.4	282465.9	7	1	3	1	183	9.73	4.57	1	5.355	0	JAGN1	High	High
660217191297362000	P22695	10997411	10397124	10997411	5645840	26	9	19	9	453	8.63	31.09	9	49.505	0	UQCRC2	High	High
2272121550069660000	P35268	56766309	53648989	56766309	29132444	30	5	12	5	128	9.19	20.44	5	26.788	0	RPL22	High	High
5134426129896320000	Q96IX5	1158276	1094618	1158276	594398.9	17	1	1	1	58	9.76	0	1	2.877	0.004	USMG5	High	Peak Found
-6782447041087310000	Q9UNF0	10441230	9866767	10441230	5357846	21	10	27	10	486	5.2	32.98	10	37.961	0	PACSIN2	High	High
370304273166594000	O94906	8669953	8189173	8669953	4446880	16	15	32	15	941	8.25	38.72	15	50.959	0	PRPF6	High	High
2457834817562910000	Q9GZY8	264414.7	249724.6	264414.7	135605.3	8	2	3	2	342	8.95	2.96	2	8.185	0	MFF	High	High
-653635778587	P48723	2125966	2007137	2125966	1089914	8	3	9	3	471	5.76	3.64	3	11.83	0	HSPA13	High	High

2280000																		
-2857975298597400000	Q96N67	4875906	4602008	4875906	2498980	7	13	18	13	2140	6.8	5.36	13	30.821	0	DOCK7	High	High
1618262664170980000	P61978	48428652	45665880	48428652	24797461	37	18	77	18	463	5.54	78.83	18	66.99	0	HNRNPK	High	High
-1761258682097810000	Q9NS86	241675.3	227814.3	241675.3	123707.6	4	1	1	1	450	7.43	0	1	3.862	0	LANCL2	High	Peak Found
5160886389078820000	Q9H939	881376.5	829948	881376.5	450677.9	5	2	3	2	334	8.48	5.82	2	5.551	0	PSTPIP2	High	High
1968544072233020000	P23246	84061921	79140731	84061921	42974955	34	24	69	23	707	9.44	103.69	24	109.917	0	SFPQ	High	High
5955155913055990000	P28062	3250224	3059219	3250224	1661215	18	4	10	4	276	7.43	17.26	4	19.979	0	PSMB8	High	High
-571108148151	P07858	39606772	37269922	39606772	20238292	22	7	34	7	339	6.3	50.63	7	48.605	0	CTSB	High	High

4940000																		
364086976847350000	Q7L592	1964510	1848099	1964510	1003554	5	2	2	2	441	8.34	1.84	2	6.441	0	NDUFAF7	High	High
-3532720828751180000	Q9UM00	1380940	1298812	1380940	705280.4	9	2	6	2	239	10.26	3.56	2	5.783	0		High	High
2594177095458510000	P24386	202318.5	190245.8	202318.5	103307.2	1	1	2	1	653	4.75	0	1	3.445	0.002	CHM	High	Peak Found
-7794810011178550000	O75348	857651.9	806335.6	857651.9	437855.9	17	2	7	2	118	8.79	2.54	2	6.385	0	ATP6V1G1	High	High
-2014146262064770000	P09001	1056513	993125.3	1056513	539286.4	5	2	4	2	348	9.48	1.88	2	4.881	0	MRPL3	High	High
-2326963864063350000	O95202	5602383	5263037	5602383	2857931	14	9	15	9	739	6.7	15.65	9	31.497	0	LETM1	High	High
481587960249	Q6UVK1	1460867	1372187	1460867	745124.3	3	6	8	6	23	5.47	6.32	6	14.071	0	CSPG4	High	High

4010000										22								
-8027894993262290000	Q13131	3409751	3202378	3409751	1738953	8	5	7	5	559	8.12	4.28	5	15.892	0	PRKAA1	High	High
5046726788440780000	Q9UNL2	3503449	3289852	3503449	1786453	11	2	5	2	185	9.61	7.44	2	12.051	0	SSR3	High	High
8246483793918850000	Q9NVI7	28463963	26720756	28463963	14509890	29	17	43	4	634	8.98	50.82	17	55.348	0	ATAD3A	High	High
-8980500697727060000	O43815	847309.1	795385.8	847309.1	431910	8	4	6	4	780	5.27	1.67	4	6.976	0	STRN	High	High
-6180191668945880000	Q9H269	540988.8	507738.6	540988.8	275711.9	4	3	4	3	839	6.77	5.42	3	9.188	0	VPS16	High	High
3740771680866670000	P62993	2616942	2455879	2616942	1333590	16	4	7	4	217	6.32	3.39	4	8.25	0	GRB2	High	High
790649955359	Q7Z5L9	537154.7	503917.6	537154.7	273637	5	2	2	2	587	8.69	2.74	2	9.282	0	IRF2BP2	High	Peak Found

2380000																		
-6123043622142940000	P55145	9532497	8939782	9532497	4854476	37	8	13	8	182	8.69	11.17	8	32.684	0	MANF	High	High
-4791788072284180000	Q96GM5	871485.3	817044.8	871485.3	443671.2	4	2	2	2	515	9.25	0	2	6.797	0	SMARCD1	High	Peak Found
8335320329939260000	Q9UBR2	13897229	13027916	13897229	7074412	27	6	13	6	303	7.11	19.96	6	19.895	0	CTSZ	High	High
-6082885004397230000	P36959	363459.4	340632.1	363459.4	184969.9	3	1	3	1	345	7.06	3.33	1	3.159	0.003	GMPR	High	High
6990414283188080000	O43660	1833226	1717544	1833226	932659.9	4	3	4	3	514	9.17	1.72	3	8.225	0	PLRG1	High	High
4196907148442890000	Q9Y2D0	1198855	1123151	1198855	609892.8	8	3	6	3	317	7.81	0	3	9.611	0	CA5B	High	High
521180101499	O43143	27543775	25802623	27543775	14011326	30	25	65	25	795	7.46	59.17	25	84.276	0	DHX15	High	High

6610000																		
-7992266646399470000	Q9Y605	137951.9	129189.5	137951.9	70152.39	8	1	1	1	127	4.7	0	1	3.174	0.003	MRFAP1	High	Peak Found
-4576818443980190000	P52292	12097064	11322002	12097064	6148067	19	9	21	9	529	5.4	24.65	9	34.937	0	KPNA2	High	High
-2742942577736860000	Q9Y2D5	2138801	2000985	2138801	1086574	7	4	9	4	859	5.11	1.71	4	12.687	0	AKAP2; PALM2-AKAP2	High	High
-7997158032231500000	Q9Y6D9	628491.4	587782.6	628491.4	319177.4	3	2	2	2	718	5.92	3.68	2	6.939	0	MAD1L1	High	Peak Found
-3000409602916510000	O15357	746398.1	697979.6	746398.1	379016.5	2	2	3	2	1258	6.54	3.68	2	8.044	0	INPPL1	High	Peak Found
342990274906048000	P48730	503016	469849.5	503016	255137.4	3	1	2	1	415	9.74	0	1	2.766	0.004	CSNK1D	Peak Found	High

1603503262744250000	P62805	41436945	38696109	41436945	21012740	51	6	23	6	103	11.36	43.24	6	39.126	0	HIST1H4A; HIST1H4F; HIST1H4D; HIST1H4J; HIST2H4A; HIST2H4B; HIST1H4H; HIST1H4C; HIST4H4; HIST1H4E; HIST1H4I; HIST1H4B; HIST1H4K; HIST1H4L	High	High
-487996163861	Q12765	5003173	4671125	5003173	2536512	13	5	9	5	414	4.75	14.11	5	29.972	0	SCRN1	High	High

1310000																		
-2819385337701020000	Q9UHB9	8345889	7787779	8345889	4228916	21	12	23	12	627	8.56	24.57	12	41.911	0	SRP68	High	High
528371413836643000	Q92945	45982058	42873745	45982058	23281277	41	23	60	19	711	7.3	72.35	23	92.62	0	KHSRP	High	High
-6347721978712800000	P17301	9152404	8533554	9152404	4633886	11	11	20	11	1181	5.31	16.77	11	36.913	0	ITGA2	High	High
-1914895012756730000	P08240	9546567	8898446	9546567	4832029	19	11	25	11	638	8.95	20.6	11	44.118	0	SRPR; SRPRA	High	High
8718116979404920000	P51116	514118.7	479060.4	514118.7	260139.1	8	4	13	2	673	6.23	16.62	4	14.229	0	FXR2	High	Peak Found
1164349567664320000	Q9NX62	4018501	3743794	4018501	2032953	8	4	7	4	359	6.86	8.12	4	11.137	0	IMPAD1	High	High
4878144000314540000	Q9NWU5	1311516	1221752	1311516	663435.1	9	2	5	2	206	9.94	9.04	2	7.551	0	MRPL22	High	High

-1447147241946060000	P61604	33973879	31647191	33973879	17185040	62	7	28	7	102	8.92	33.98	7	28.931	0	HSPE1	High	High
4936048942270730000	O00567	11675460	10872547	11675460	5904004	21	12	29	12	594	9.19	45.39	12	45.274	0	NOP56	High	High
105236357953779000	Q9H0R4	398346.2	370822.9	398346.2	201364	7	2	2	2	259	6.24	0	2	3.433	0.002	HDHD2	High	Peak Found
-8439229847927280000	Q658P3	6950806	6466887	6950806	3511646	13	6	13	6	488	8.6	18.69	6	27.754	0	STEAP3	High	High
1348431233397440000	P62495	7417173	6899758	7417173	3746702	20	8	20	8	437	5.71	22.07	8	40.135	0	ETF1	High	High
5508614142316910000	Q07973	537519.6	499970.8	537519.6	271493.9	1	1	2	1	514	8.75	0	1	2.324	0.008	CYP24A1	Peak Found	High
-5308082143838350000	P42766	10528583	9790796	10528583	5316593	37	5	17	5	123	11.05	12.43	5	21.686	0	RPL35	High	High

-728784246141405000	P09543	2556224	2376852	2556224	1290677	13	5	8	5	421	9.07	8.89	5	16.483	0	CNP	High	High
-3086265819139450000	O14773	2188566	2034945	2188566	1105015	4	2	6	2	563	6.48	0	2	5.921	0	TPP1	High	High
4429006779143250000	Q14320	4987643	4632167	4987643	2515356	18	7	8	7	339	6.83	2.74	7	24.984	0	FAM50A	High	High
4529992090848900000	Q01968	229780.8	213375.8	229780.8	115867.2	1	1	2	1	901	6.55	0	1	2.323	0.008	OCRL	High	Peak Found
1948435004993000000	P52594	395997.2	367639.5	395997.2	199635.4	6	2	2	2	562	8.63	0	2	4.252	0	AGFG1	High	High
-3427598645898200000	O14828	4586573	4256631	4586573	2311433	10	3	7	3	347	7.64	7.53	3	12.615	0	SCAMP3	High	High
4066776278631370000	P17612	5177666	4804251	5177666	2608802	17	6	12	6	351	8.79	6.83	6	16.509	0	PRKACA	High	High

-5318863470258600000	Q8IWE2	5899488	5473929	5899488	2972450	15	8	15	8	563	4.68	15.01	8	29.908	0	FAM114A1	High	High
-1176718520801110000	P11441	541891.5	502655.8	541891.5	272951.9	11	2	4	2	157	8.66	3.63	2	4.819	0	UBL4A	High	High
-7100137965290350000	Q9UJW0	427898.1	396860.7	427898.1	215503.1	7	2	5	2	460	7.34	3.82	2	8.3	0	DCTN4	High	High
3834517730795480000	Q05655	369089.6	341967	369089.6	185694.8	4	3	5	2	676	7.75	4.56	3	8.572	0	PRKCD	High	High
2236542177406700000	P52597	6597294	6110810	6597294	3318289	19	6	22	5	415	5.58	33.62	6	40.599	0	HNRNPF	High	High
4915736494579550000	Q96TA1	22321279	20670289	22321279	11224369	22	18	35	18	746	6.19	37.69	18	67.801	0	FAM129B	High	High
8586713697824140000	P46379	3373583	3123658	3373583	1696207	5	6	9	6	1132	5.6	10.02	6	17.878	0	BAG6	High	High

5241599952766810000	P09211	38413761	35565940	38413761	19312997	70	11	33	11	210	5.64	37.95	11	54.166	0	GSTP1	High	High
-3538958500289370000	P49336	153244.8	141780.6	153244.8	76989.62	2	1	1	1	464	8.57	0	1	2.517	0.006	CDK8	High	Peak Found
6049442156646560000	Q6UXN9	2438740	2255918	2438740	1225007	19	6	12	6	313	7.69	6.85	6	13.518	0	WDR82	High	High
-5049084120360680000	Q6PL24	96796.09	89529.29	96796.09	48616.14	2	1	1	1	325	4.74	0	1	2.116	0.01	TMED8	High	Peak Found
-8095866348413870000	Q8WUB8	263409.6	243630.2	263409.6	132295.9	2	1	1	1	498	6.62	1.65	1	2.242	0.009	PHF10	High	Peak Found
9004188409903140000	P52758	2203508	2037409	2203508	1106353	27	3	10	3	137	8.68	16.13	3	17.973	0	HRSP12; RIDA	High	High
6222929657638760000	P23284	1.89E+08	1.75E+08	1.89E+08	94816775	57	15	104	15	216	9.41	145.81	15	94.577	0	PPIB	High	High

-3269351355245220000	Q8NF37	10928542	10099821	10928542	5484399	15	8	29	8	534	6.02	41.15	8	29.726	0	LPCAT1	High	High
3946682035619070000	Q00688	6408755	5922438	6408755	3215999	33	7	16	7	224	9.28	24.1	7	27.851	0	FKBP3	High	High
8611257715597880000	Q15555	1592741	1471241	1592741	798912.7	13	3	5	3	327	5.57	9.7	3	16.329	0	MAPRE2	High	High
-4912222715256490000	O43390	68714554	63472190	68714554	34466633	35	21	71	14	633	8.13	92.23	21	109.942	0	HNRNPR	High	High
1534536598926650000	P05198	20969638	19364537	20969638	10515320	29	10	31	10	315	5.08	38	10	44.796	0	EIF2S1	High	High
6973365880698080000	Q9UHD8	22612749	20870480	22612749	11333076	30	13	37	13	586	8.97	45.06	13	55.599	0	SEPT9	High	High
-2473277628257970000	Q5SRE5	211489.3	195144.2	211489.3	105967.1	1	2	3	2	1749	6.73	0	2	6.321	0	NUP188	High	High

-3069559242831620000	Q7Z460	1908605	1761018	1908605	956267.1	3	4	7	3	1538	9.03	5.28	4	11.127	0	CLASP1	High	High
562319574892213000	P04406	1.64E+09	1.51E+09	1.64E+09	8.21E+08	65	25	216	24	335	8.46	408.23	25	270.509	0	GAPDH	High	High
-8217852517475640000	Q9H3U1	4262379	3931170	4262379	2134702	9	8	15	8	944	6.07	17.55	8	25.45	0	UNC45A	High	High
7638543548033190000	Q00765	9675029	8913845	9675029	4840391	21	5	12	5	189	8.1	29.81	5	29.873	0	REEP5	High	High
4037115124737050000	P04439	35116051	32345658	35116051	17564321	37	11	25	9	365	6	40.56	11	50.726	0	HLA-A	High	High
-3428809261446700000	Q9UNN5	6553555	6035735	6553555	3277522	13	8	16	8	650	4.88	15.44	8	38.958	0	FAF1	High	High
-5915355530920660000	Q13637	2468100	2272997	2468100	1234281	14	3	7	2	225	6.54	10.03	3	10.597	0	RAB32	High	High

-5985118232380260000	Q15363	8341496	7668818	8341496	4164317	29	4	9	4	201	5.17	9.39	4	14.721	0	TMED2	High	High
3823742586445680000	Q9NPQ8	5360635	4926436	5360635	2675150	22	10	17	10	531	5.33	16.01	10	26.899	0	RIC8A	High	High
3988620745573500000	P55786	26590169	24435348	26590169	13268869	24	22	47	22	919	5.72	59.96	22	82.347	0	NPEPPS	High	High
157541479094675000	Q96AB3	144899.9	133128.8	144899.9	72291.52	10	1	1	1	205	7.77	0	1	2.272	0.009	ISOC2	High	Peak Found
5195748519794980000	P48509	6241901	5734220	6241901	3113793	17	4	9	4	253	7.47	12.48	4	15.558	0	CD151	High	High
1708640290626260000	P37108	7717969	7089538	7717969	3849757	23	4	10	4	136	10.04	8.81	4	17.916	0	SRP14	High	High
-6880355764133460000	P26358	8019197	7363925	8019197	3998754	10	16	24	16	1616	7.75	13.17	16	43.143	0	DNMT1	High	High
-319296939457	Q9BT17	265373.3	243611	265373.3	132285.5	3	1	1	1	334	9.47	0	1	3.009	0.003	MTG1	Peak Found	High

5090000																		
-1307645131965220000	Q96CG8	1214526	1113082	1214526	604425	12	3	3	3	243	7.99	1.87	3	6.495	0	CTHRC1	High	High
-9168897576622990000	O43617	3655249	3349882	3655249	1819051	17	3	7	3	180	4.96	1.89	3	11.392	0	TRAPPC3	High	High
-1377635877244790000	P36873	842877.6	772380.7	842877.6	419417.8	29	8	22	1	323	6.54	20.69	8	49.639	0	PPP1CC	High	Peak Found
1428811385271770000	Q9H488	4431064	4059705	4431064	2204499	9	3	10	3	388	8.53	5.86	3	10.803	0	POFUT1	High	High
-792306396308701000	Q9Y2W1	6067487	5558623	6067487	3018440	9	8	12	8	955	10.15	12.07	8	24.656	0	THRAP3	High	High
-1760362922626910000	Q14011	1376312	1260683	1376312	684575.3	20	2	4	2	172	9.51	2.1	2	6.288	0	CIRBP	High	High

-4415570283207220000	Q08257	4827579	4421747	4827579	2401094	22	6	11	6	329	8.44	8.63	6	18.537	0	CRYZ	High	High
6596351157083830000	Q8NC56	1587839	1454036	1587839	789569.9	6	3	3	3	503	9	1.84	3	8.607	0	LEMD2	High	Peak Found
-8194884886177580000	Q14192	3786571	3466592	3786571	1882427	21	6	13	6	279	7.55	11.3	6	14.537	0	FHL2	High	High
-8198800325193530000	P30050	77046429	70532932	77046429	38300754	59	8	22	8	165	9.42	56.06	8	53.551	0	RPL12	High	High
-4830311805247250000	Q9NPD3	1519903	1391085	1519903	755386.4	10	2	3	2	245	6.52	2.56	2	5.982	0	EXOSC4	High	High
-231036959167870000	P43357	304963.7	279112.6	304963.7	151563.6	3	1	2	1	314	4.59	0	1	2.312	0.008	MAGEA3;MAGEA6	High	Peak Found
-402747489269	Q9BZI7	348069	318510.1	348069	172957.2	6	3	4	3	483	9.48	0	3	7.205	0	UPF3B	High	High

6140000																		
-1887782222287180000	Q9ULV4	35555093	32534952	35555093	17667112	25	14	46	14	474	7.08	77.44	14	79.685	0	CORO1C	High	High
7436482478148800000	Q86TB9	6885412	6300276	6885412	3421172	2	1	1	1	770	6.67	0	1	2.466	0.007	PATL1	High	Peak Found
3793795971274930000	Q96S59	951588.1	870335.2	951588.1	472609	3	2	4	2	729	6.79	3.63	2	7.854	0	RANBP9	High	High
-5091177938667010000	P41743	198490.4	181540.4	198490.4	98579.97	2	1	1	1	596	5.85	1.66	1	4.04	0	PRKCI	High	Peak Found
35575557204019300	Q96L34	384877.9	351904.4	384877.9	191090.9	2	2	3	2	752	9.66	0	2	4.691	0	MARK4	High	High
-2799145337949170000	O15294	1961240	1792377	1961240	973295.6	7	6	8	6	1046	6.7	7.28	6	18.741	0	OGT	High	High
-381899878977	Q9Y4W6	11103877	10144470	11103877	5508644	12	11	25	11	797	8.66	23.81	11	42.666	0	AFG3L2	High	High

0130000																		
-4518810540954690000	Q8TAT6	6568409	6000519	6568409	3258399	19	11	16	11	608	6.38	25.69	11	41.369	0	NPLOC4	High	High
-675276312923681000	Q96GY0	273253.7	249603.1	273253.7	135539.3	2	1	1	1	325	9.82	1.81	1	2.254	0.009	ZC2HC1A	High	Peak Found
-956583544951272000	Q8IY81	473310.5	431911.9	473310.5	234536.6	1	1	2	1	847	8.4	3.68	1	2.98	0.003	FTSJ3	High	High
315483607845859000	Q9BRF8	213096.8	194421.2	213096.8	105574.5	6	2	5	2	314	6.2	1.69	2	4.872	0	CPPED1	High	High
4191413839330820000	O14979	9622034	8778608	9622034	4766955	16	9	16	7	420	9.57	21.68	9	30.16	0	HNRNPDL; HNRPDL	High	High
5730527264906500000	Q93045	6499532	5928618	6499532	3219355	11	2	3	1	179	8.32	6.41	2	6.513	0	STMN2	High	Peak Found
6991992927110110000	Q99504	179742.9	163856	179742.9	88977.02	2	1	1	1	573	5.21	1.77	1	2.866	0.004	EYA3	High	Peak Found

-8882498354911650000	Q00341	53721583	48931065	53721583	26570520	29	39	84	39	1268	6.87	101.52	39	141.953	0	HDLBP	High	High
5676242339415690000	Q9H6X2	535390.5	487537.1	535390.5	264742.1	5	2	3	1	564	7.61	3.85	2	6.916	0	ANTXR1	High	Peak Found
-8166757714401600000	O75964	7477217	6807969	7477217	3696860	34	3	14	3	103	9.64	25.47	3	16.98	0	ATP5L	High	High
-3455107625301320000	Q86TX2	2773330	2523702	2773330	1370419	7	3	7	3	421	7.34	3.62	3	9.915	0	ACOT1	High	High
-9171781996039320000	P57105	262003.9	238329.7	262003.9	129417.6	8	1	1	1	145	6.3	0	1	2.887	0.004	SYNJ2BP	High	Peak Found
6179525910857730000	P49590	1315817	1196085	1315817	649497.4	9	5	14	2	506	8.24	7.35	5	17.55	0	HARS2	High	High
-555755608938	Q9UN86	5943020	5402239	5943020	2933521	12	4	13	4	482	5.55	25.6	4	46.931	0	G3BP2	High	High

4340000																			
5868182849043770000	Q15084	1.35E+08	1.23E+08	1.35E+08	66780810	38	17	81	17	440	5.08	164.71	17	122.038	0	PDIA6	High	High	
1214418954468830000	Q96C01	550891.3	500655	550891.3	271865.4	12	2	2	2	138	7.61	1.95	2	4.869	0	FAM136A	High	Peak Found	
8959755046282960000	Q9NUQ7	298030.4	270700.7	298030.4	146995.8	4	1	2	1	469	7.01	0	1	2.373	0.008	UFSP2	High	High	
4163681238843760000	Q13144	2674439	2428127	2674439	1318520	10	6	8	6	721	5.08	8.22	6	18.405	0	EIF2B5	High	High	
-4877506006391290000	P28072	19161645	17396197	19161645	9446473	17	4	19	4	239	4.92	44.44	4	15.158	0	PSMB6	High	High	
1392288504420240000	Q9Y320	2754364	2499823	2754364	1357452	17	5	9	5	296	8.69	4.51	5	14.575	0	TMX2	High	High	
8288854614025870000	P62258	1.18E+08	1.07E+08	1.18E+08	57916758	65	21	123	18	255	4.74	209.29	21	115.497	0	YWHAE	High	High	

5660191974967280000	P15144	90056226	81720368	90056226	44375749	30	32	88	32	967	5.48	138.85	32	141.71	0	ANPEP	High	High
-6076277421311930000	O43930	377086.6	342168.2	377086.6	185804	3	1	2	1	277	6.96	0	1	3.008	0.003	PRKY	High	High
454806171855366000	P40939	69029543	62633123	69029543	34011004	28	21	74	21	763	9.04	137.15	21	105.834	0	HADHA	High	High
-3895528791649610000	Q86UE4	6681044	6061603	6681044	3291568	14	6	18	6	582	9.32	15.33	6	27.461	0	MTDH	High	High
4099103662110180000	Q08722	9317080	8452409	9317080	4589823	11	4	8	4	323	7.21	9.88	4	11.351	0	CD47	High	High
8546786530734250000	Q00325	52219682	47356156	52219682	25715313	26	12	45	12	362	9.38	68.8	12	36.476	0	SLC25A3	High	High
-2219235455752220000	P29558	1111488	1007184	1111488	546920.3	4	1	2	1	406	8.79	2.08	1	4.232	0	RBMS1	Peak Found	High

6090154179084710000	P36551	12069924	10935564	12069924	5938224	34	12	29	12	454	8.25	32.08	12	56.228	0	CPOX	High	High
2249257681079230000	O00622	2347437	2126656	2347437	1154815	10	4	6	4	381	8.21	1.98	4	14.138	0	CYR61	High	High
-2296645769357170000	Q4G0J3	1142540	1034559	1142540	561785.5	5	3	3	3	582	9.55	0	3	7.497	0	LARP7	High	Peak Found
4874670657009690000	Q6P2Q9	22712736	20563207	22712736	11166221	16	34	63	34	2335	8.84	41.11	34	107.695	0	PRPF8	High	High
5545156882891980000	P07954	18986177	17188014	18986177	9333426	25	10	17	10	510	8.76	22.44	10	42.662	0	FH	High	High
2192359693732380000	O95433	13637974	12335701	13637974	6698526	35	11	28	11	338	5.53	27.96	11	37.754	0	AHSA1	High	High
-2292593222020040000	Q92925	339876.5	307405.3	339876.5	166927.1	3	1	2	1	531	9.64	0	1	3.725	0.001	SMARCD2	High	High

5028999113090770000	Q8WVB6	207387.1	187538.4	207387.1	101837	1	1	1	1	975	7.21	0	1	3.016	0.002	CHTF18	High	Peak Found
-4968571585111950000	P55036	6631882	5997021	6631882	3256499	21	7	22	7	377	4.79	22.42	7	30.104	0	PSMD4	High	High
-8026992804565500000	Q13257	2287405	2068331	2287405	1123144	9	2	4	2	205	5.08	2.02	2	6.198	0	MAD2L1	High	Peak Found
-4672476971480330000	P67870	2169940	1961819	2169940	1065306	15	3	4	3	215	5.55	4.9	3	7.462	0	CSNK2B	High	Peak Found
-7687399049633850000	Q9BY67	2652142	2396936	2652142	1301583	10	3	4	3	442	5.07	7.25	3	9.007	0	CADM1	High	High
-7996952704107550000	P28331	5880437	5313621	5880437	2885400	21	12	29	12	727	6.23	31.35	12	45.029	0	NDUFS1	High	High
-662526894904	Q8N163	15011594	13564511	15011594	7365793	15	13	22	13	923	5.22	23.07	13	44.332	0	CCAR2; KIAA1967	High	High

5140000																		
-1852571755298910000	Q9UKX7	1421548	1284347	1421548	697425.5	9	3	3	3	468	7.06	4.01	3	10.704	0	NUP50	High	High
-1014508030414010000	P62899	34334503	31017437	34334503	16843071	14	3	12	3	125	10.54	18.37	3	8.821	0	RPL31	High	High
4495210949063330000	Q7L576	13238606	11959175	13238606	6494065	13	15	39	15	1253	6.9	34.01	15	66.012	0	CYFIP1	High	High
4154359228263860000	P46781	34254816	30941214	34254816	16801681	48	13	33	13	194	10.65	37.96	13	35.078	0	RPS9	High	High
687508345564059000	Q15436	34505371	31146368	34505371	16913083	35	22	57	20	765	7.08	79.54	22	104.248	0	SEC23A	High	High
-6145524999594340000	O60518	940475.8	848574.8	940475.8	460792.6	3	3	4	2	1105	5.01	0	3	6.569	0	RANBP6	High	High
6910081975799460000	O15042	14807952	13350135	14807952	7249383	6	7	13	7	1029	8.47	12.44	7	23.377	0	U2SURP	High	High

8283666536217060000	P50281	6219124	5606608	6219124	3044497	9	5	9	5	582	7.77	14.7	5	15.001	0	MMP14	High	High
9172177916429740000	Q99816	3764702	3393870	3764702	1842937	9	4	7	4	390	6.46	8.58	4	12.16	0	TSG101	High	High
4718065310323380000	Q5VSL9	545823.5	491983.3	545823.5	267156.5	2	2	9	2	837	6.29	3.24	2	5.45	0	STRIP1	High	High
-9218003044822150000	O95881	11717482	10556538	11717482	5732405	32	5	6	5	172	5.4	3.59	5	11.012	0	TXNDC12	High	High
-6220946712320520000	Q7L5D6	302753.2	272553.9	302753.2	148002.1	4	1	4	1	327	5.41	0	1	5.647	0	GET4	High	High
-4550175075661580000	P00403	2103920	1893725	2103920	1028329	4	1	4	1	227	4.82	4.28	1	3.469	0.002	COX2	High	High
7212172965787630000	Q15031	2063017	1856896	2063017	1008330	7	6	9	6	903	8.22	3.38	6	15.668	0	LARS2	High	High

5417466030528510000	Q9H936	2841501	2557416	2841501	1388727	12	4	7	4	323	9.29	8.9	4	9.464	0	SLC25A22	High	High
-7687733002656650000	P18031	9615321	8651099	9615321	4697715	27	10	23	10	435	6.27	23.66	10	39.367	0	PTPN1	High	High
3269651725271290000	P02751	27845202	25051118	27845202	13603244	11	21	53	21	2477	5.5	80.43	21	110.175	0		High	High
3369137616140020000	Q9NZJ9	803645.3	723002.9	803645.3	392604.6	19	2	3	2	180	6.35	1.72	2	5.767	0	NUDT4	High	High
5057410005729890000	Q7Z4Q2	305758.5	275009.5	305758.5	149335.5	2	1	3	1	680	5.11	1.85	1	2.688	0.005	HEATR3	High	High
5188184621199480000	Q9GZT3	2857011	2569657	2857011	1395374	18	2	5	2	109	10.24	8.67	2	6.869	0	SLIRP	High	High
289825156273590000	Q5JTV8	2899731	2608003	2899731	1416196	10	5	10	5	583	8.18	7.74	5	13.273	0	TOR1AIP1	High	High
-428705817762	O95757	8990871	8084718	8990871	4390159	22	16	32	12	839	5.88	38.59	16	62.285	0	HSPA4L	High	High

2280000																		
-2128989799875940000	Q92888	2398144	2156100	2398144	1170804	6	5	7	5	912	5.66	6.34	5	14.34	0	ARHGEF1	High	High
6148920523022940000	P35606	59159423	53182620	59159423	28879197	35	28	76	28	906	5.27	109.88	28	136.164	0	COPB2	High	High
-3264120083815800000	O14737	3485664	3132899	3485664	1701225	34	5	10	5	125	6.04	13.26	5	23.432	0	PDCD5	High	High
-5710815529521280000	Q9BQ70	1921274	1726272	1921274	937399.2	4	3	9	3	676	6.35	7.05	3	8.937	0	TCF25	High	High
8059296943260830000	Q15286	2720330	2442941	2720330	1326564	33	6	16	4	201	8.29	27.54	6	25.887	0	RAB35	High	High
-3718906404474100000	P19174	1897690	1704059	1897690	925337.3	4	4	7	4	1290	6.05	6.01	4	15.875	0	PLCG1	High	High

8567632749878030000	Q9H553	980259.9	879889.1	980259.9	477796.9	5	2	2	2	416	7.05	1.67	2	3.333	0.002	ALG2	High	Peak Found
3220209801310800000	P60891	4967174	4457311	4967174	2420406	17	4	9	2	318	6.98	26.69	4	28.921	0	PRPS1	High	High
1473642583666420000	P16615	32246739	28926834	32246739	15707834	24	25	66	25	1042	5.34	89.28	25	93.785	0	ATP2A2	High	High
2475503787040520000	Q9UM54	5635210	5054173	5635210	2744514	13	16	24	16	1294	8.53	17.15	16	52.102	0	MYO6	High	High
-2125908269399920000	Q9UKK3	1230209	1103190	1230209	599053.8	2	4	6	4	1724	5.66	1.86	4	9.814	0	PARP4	High	High
5021829867861970000	Q14671	3089755	2770342	3089755	1504350	3	4	4	4	1186	6.84	7.5	4	13.374	0	PUM1	High	Peak Found
1922976354547060000	Q969G5	17416478	15615231	17416478	8479374	21	5	18	5	261	6.43	33.02	5	31.346	0	PRKCDBP; CAVIN3	High	High
-231059307036	Q15599	2422810	2171122	2422810	1178961	15	4	10	4	337	7.93	15.76	4	17.997	0	SLC9A3R2	High	High

2030000																		
2637590011477770000	P46459	11671088	10445408	11671088	5672060	22	16	29	16	744	6.95	21.71	16	54.207	0	NSF	High	High
-6941898924238940000	P52565	16675944	14922827	16675944	8103385	30	7	20	7	204	5.11	30.05	7	41.502	0	ARHGDIA	High	High
-374289036912732000	Q15042	905453.1	810194.7	905453.1	439951.5	3	3	4	3	981	5.55	0	3	6.852	0	RAB3GAP1	High	High
-174490317750568000	P60709	3.62E+09	3.24E+09	3.62E+09	1.76E+09	74	32	316	10	375	5.48	487.77	32	287.907	0	ACTB	High	High
4204109305997430000	Q07960	17081349	15274494	17081349	8294347	23	10	21	10	439	6.29	27.04	10	49.933	0	ARHGAP1	High	High
-7478524853152130000	P43487	14050122	12562744	14050122	6821814	27	6	14	6	201	5.29	15.25	6	24.832	0	RANBP1	High	High
-141835042818	O43678	671900.4	600690.6	671900.4	326186.7	30	2	2	2	99	9.57	0	2	5.102	0	NDUFA2	High	Peak Found

7620000																		
-6634047056467400000	P62318	24428262	21837679	24428262	11858284	32	3	14	3	126	10.32	27.46	3	21.414	0	SNRPD3	High	High
-6261217039934200000	P39656	37712254	33701020	37712254	18300309	17	7	21	7	456	6.55	36.78	7	36.145	0	DDOST	High	High
-1450740357169920000	P40121	30318932	27086020	30318932	14708236	28	7	30	7	348	6.19	43.49	7	44.148	0	CAPG	High	High
-5234927014907960000	Q9H0S4	13848275	12369885	13848275	6717088	10	4	10	4	455	9.1	7.16	4	11.148	0	DDX47	High	High
5448927889689110000	Q16539	1683362	1503433	1683362	816393.2	8	3	3	3	360	5.78	1.88	3	6.424	0	MAPK14	High	High
3002712347046380000	P78371	63289987	56524633	63289987	30693975	49	20	47	20	535	6.46	83.95	20	112.706	0	CCT2	High	High

-6780722004559030000	P0DN79	4370674	3903292	4370674	2119564	7	4	6	4	551	6.65	7.42	4	11.859	0	CBS; LOC102724560; CBSL	High	High
2399497510241030000	P25705	2.1E+08	1.87E+08	2.1E+08	1.02E+08	37	21	115	21	553	9.13	171.43	21	123.164	0	ATP5A1	High	High
-4685450292699180000	P43246	13619463	12162732	13619463	6604600	18	14	31	14	934	5.77	39.56	14	54.836	0	MSH2	High	High
7474721122624860000	Q8IVL5	3976822	3548935	3976822	1927141	11	7	17	6	708	5.71	11.32	7	21.878	0	LEPREL1; P3H2	High	High
1688448728000240000	Q9BZH6	105782.8	94333.2	105782.8	51224.76	3	3	3	3	1224	6.92	0	3	6.758	0	WDR11	High	Peak Found
-7543503445909430000	P13798	11117374	9911640	11117374	5382213	20	13	22	13	732	5.48	21.83	13	44.011	0	APEH	High	High
-4797790429512710000	O75874	30909649	27548968	30909649	14959625	39	15	39	14	414	7.01	57.1	15	67.044	0	IDH1	High	High

3877552904475960000	Q9NX63	575418.1	512851.9	575418.1	278488.5	11	2	2	2	227	8.28	2.14	2	7.593	0	CHCHD3	High	Peak Found
-4838480320139960000	Q13951	345906.8	308229.7	345906.8	167374.7	5	1	1	1	182	6.6	1.96	1	3.149	0.003	CBFB	High	Peak Found
3211480978683400000	O95232	529647.1	471873.6	529647.1	256236.5	2	1	2	1	432	9.79	2.37	1	4.367	0	LUC7L3	High	High
8046944082778510000	P32119	23918829	21306831	23918829	11570024	28	7	33	6	198	5.97	51.98	7	26.128	0	PRDX2	High	High
6250507139563180000	P31641	689778.8	614303.9	689778.8	333578.9	1	1	2	1	620	7.39	0	1	2.938	0.004	SLC6A6	High	High
-2978485946285830000	Q8NHH9	454812.4	404976	454812.4	219909.9	3	1	1	1	583	5.48	2.15	1	3.919	0	ATL2	High	Peak Found
3408608203848960000	Q9Y6K0	546410.4	486426.9	546410.4	264139.3	2	1	1	1	416	8.21	1.87	1	4.288	0	CEPT1	High	Peak Found

692688187917917000	P82979	3013849	2682919	3013849	1456877	15	3	5	3	210	6.42	4.48	3	10.845	0	SARNP	High	High
1718244645714620000	Q9UIA9	26990007	24024734	26990007	13045898	16	18	36	18	1087	6.32	42.58	18	58.488	0	XPO7	High	High
5314781854584040000	O75436	6576448	5853016	6576448	3178302	19	6	13	6	327	6.57	13.98	6	26.612	0	VPS26A	High	High
-8254999691274010000	Q9HAV0	2356395	2097069	2356395	1138749	20	7	15	2	340	6	21.36	7	21.798	0	GNB4	High	High
-3928558694847440000	Q9UBQ5	4318946	3840391	4318946	2085407	24	4	10	4	218	4.93	15.13	4	18.819	0	EIF3K	High	High
92352165089450500	O15355	8939838	7946980	8939838	4315365	26	10	21	10	546	4.36	25.47	10	49.073	0	PPM1G	High	High
-5207555211981110000	Q08J23	16751198	14889669	16751198	8085380	25	16	37	16	767	6.77	41.3	16	69.402	0	NSUN2	High	High
-457073368993	P61106	13403272	11906376	13403272	6465394	45	8	19	7	215	6.21	27.13	8	29.454	0	RAB14	High	High

4670000																		
-6874102383142950000	Q5TAT6	224099.9	199052.1	224099.9	108089.2	2	1	2	1	717	9.17	0	1	2.415	0.008	COL13A1	High	High
712228557573966000	Q9ULC3	2521629	2239744	2521629	1216225	16	3	6	3	237	6.6	2.64	3	11.509	0	RAB23	High	High
2100533050409740000	Q9P0K7	3367632	2990777	3367632	1624050	11	10	12	10	980	6.21	5.93	10	27.544	0	RAI14	High	High
-8826640121646050000	P13473	14415028	12801676	14415028	6951559	4	2	5	2	410	5.63	5.73	2	6.152	0	LAMP2	High	High
9128432651374980000	Q9NNW5	746560.5	662646.7	746560.5	359830	3	3	3	3	1121	6.87	4.09	3	8.188	0	WDR6	High	Peak Found
-273580326344755000	P26885	6248187	5545121	6248187	3011109	19	3	7	3	142	9.13	8.71	3	14.881	0	FKBP2	High	High
652466818170242000	Q9H993	893298.9	792536.9	893298.9	430362.9	7	3	4	3	441	5.76	1.74	3	4.805	0	C6orf211; ARMT1	High	High

2668394544474580000	Q99470	899683.1	798020.7	899683.1	433340.8	18	2	6	2	211	7.33	2.63	2	6.724	0	SDF2	High	High
5579729439990200000	P06733	1.71E+09	1.51E+09	1.71E+09	8.21E+08	69	37	211	34	434	7.39	389.05	37	317.457	0	ENO1	High	High
3476317971320690000	O43156	907589.4	804080.8	907589.4	436631.5	2	2	3	2	1089	5.97	4.68	2	6.217	0	TTI1	High	High
4245346630009390000	P50479	804824	713016.6	804824	387181.9	8	3	6	3	330	7.91	3.86	3	7.038	0	PDLIM4	High	High
439747598683617000	Q9UI10	3039163	2692196	3039163	1461915	14	6	14	6	523	9.38	12.64	6	23.694	0	EIF2B4	High	High
-5100342078982030000	Q8N684	2369274	2098139	2369274	1139330	8	4	5	4	471	8	7.92	4	13.23	0	CPSF7	High	High
-8670958433918100000	P26373	31875037	28224186	31875037	15326282	38	8	20	8	211	11.65	39.97	8	31.346	0	RPL13	High	High
-612318433386	Q96BY6	842880.7	746185.7	842880.7	405193.3	2	5	8	5	21	7.14	1.6	5	13.756	0	DOCK10	High	High

2710000										86								
8304538899155540000	Q9NVD7	4373669	3870736	4373669	2101885	17	6	10	5	372	5.95	11.67	6	26.465	0	PARVA	High	High
-8632288723347510000	Q03013	212387.6	187956.9	212387.6	102064.3	15	3	6	1	218	5.9	2.26	3	10.676	0	GSTM4	High	High
-8332835024876140000	Q13445	2839300	2512327	2839300	1364242	8	2	6	2	227	4.48	5.6	2	8.929	0	TMED1	High	High
5004824679755990000	Q13867	3239056	2865190	3239056	1555854	11	4	9	4	455	6.27	14.65	4	19.285	0	BLMH	High	High
-5921429182500700000	O43765	4745250	4195707	4745250	2278351	19	5	11	5	313	4.87	10.84	5	22.663	0	SGTA	High	High
-7785579833791400000	P17980	24706086	21842525	24706086	11860916	32	13	34	13	439	5.24	38.02	13	52.213	0	PSMC3	High	High

-4839945324263830000	Q9NTX5	2836729	2506601	2836729	1361133	10	3	4	3	307	8.21	5.59	3	7.325	0	ECHDC1	High	High
1847486651469820000	O60762	1421132	1255745	1421132	681893.8	4	1	2	1	260	9.57	5.63	1	5.84	0	DPM1	High	High
353320057128082000	Q13242	2200237	1943921	2200237	1055587	14	3	9	3	221	8.65	5.79	3	10.427	0	SRSF9	High	High
338610440228744000	Q13620	31402131	27741091	31402131	15063952	22	22	49	22	913	7.37	63.81	22	86.043	0	CUL4B	High	High
-4570011154676120000	Q9NRF9	691716.9	611013.5	691716.9	331792.2	7	1	4	1	147	4.74	5.16	1	2.834	0.004	POLE3	High	High
-3533689105478900000	Q15369	10971566	9691361	10971566	5262597	59	6	19	6	112	4.78	28.39	6	33.575	0	TCEB1; ELOC	High	High
-6811152372553300000	Q9Y2X3	9302580	8216031	9302580	4461465	18	8	15	8	529	8.92	25.23	8	44.423	0	NOP58	High	High

2825155014730550000	Q6GMV2	1385083	1222601	1385083	663896.3	2	1	1	1	418	5.05	1.71	1	2.475	0.007	SMYD5	High	Peak Found
8965966782522850000	P30837	6034598	5325592	6034598	2891900	21	9	23	7	517	6.8	24.94	9	33.066	0	ALDH1B1	High	High
1858767456070720000	P55735	8484843	7487123	8484843	4065654	22	6	16	6	322	5.48	27.2	6	24.131	0	SEC13	High	High
-9007113237130650000	O15498	7019421	6192749	7019421	3362783	18	5	6	5	198	6.92	6.08	5	14.257	0	YKT6	High	High
2386724705728610000	Q9UHB6	12066414	10644147	12066414	5779979	15	9	15	9	759	6.84	9.83	9	33.567	0	LIMA1	High	High
5335677543697520000	P17844	90070011	79417630	90070011	43125317	36	24	72	18	614	8.92	110.9	24	98.922	0	DDX5	High	High
3146849743976840000	Q02809	65008980	57299269	65008980	31114617	34	23	66	23	727	6.95	88.18	23	101.94	0	PLOD1	High	High
308083216839	Q9Y6D6	4529577	3991689	4529577	2167565	7	11	16	7	18	5.85	9.77	11	30.925	0	ARFGEF1	High	High

9390000										49								
-797188196996991000	P15170	9925253	8742735	9925253	4747475	16	8	19	8	499	5.62	24.96	8	29.857	0	GSPT1	High	High
7316364973511520000	Q8IYI6	366914.9	323104.7	366914.9	175452.1	2	2	2	2	725	5.49	0	2	4.754	0	EXOC8	High	Peak Found
2868702294912810000	P60174	3.21E+08	2.83E+08	3.21E+08	1.54E+08	80	17	84	17	249	6.9	167.58	17	118.906	0	TPI1	High	High
-5870318523395650000	P49591	19374151	17056311	19374151	9261909	30	13	33	13	514	6.43	48.23	13	62.311	0	SARS	High	High
-1407036769668950000	Q9HAN9	2015590	1774396	2015590	963531.7	12	3	8	3	279	8.87	14.74	3	10.192	0	NMNAT1	High	High
-6707241685728670000	Q9NY93	866365.4	762450.4	866365.4	414025.4	4	2	4	2	547	9.26	5.44	2	7.178	0	DDX56	High	High
-499050701687	P42126	4435973	3903175	4435973	2119500	18	5	14	5	302	8.54	17.15	5	19.213	0	ECI1	High	High

8310000																		
3710113209643880000	O75794	398493.6	350600.3	398493.6	190382.8	2	1	3	1	336	4.81	0	1	2.371	0.008	CDC123	High	High
8013273140133150000	Q9H3P7	3921655	3449549	3921655	1873172	15	6	11	6	528	5.06	11.28	6	21.732	0	ACBD3	High	High
-5797978110445310000	Q9BZG1	4120352	3623684	4120352	1967731	21	5	9	5	259	7.88	5.95	5	17.295	0	RAB34	High	High
1141936988023860000	Q9NP61	1219239	1072172	1219239	582210.3	5	3	7	3	516	7.36	1.71	3	6.616	0	ARFGAP3	High	High
6179011071974480000	Q9NWS0	447218.5	393258.8	447218.5	213547.2	5	1	3	1	290	5.14	0	1	4.609	0	PIH1D1	High	High
-1664770674535560000	Q96T37	185677.4	163223.2	185677.4	88633.38	2	1	2	1	977	10.08	2.12	1	4.053	0	RBM15	High	High
5136159273712400000	P38919	10262433	9018748	10262433	4897356	29	14	40	11	411	6.73	73.93	14	73.034	0	EIF4A3	High	High

-4617626252802510000	Q9NZU5	156476.3	137497.6	156476.3	74663.86	3	1	1	1	365	7.93	0	1	2.844	0.004	LMCD1	High	Peak Found
-4852741013431450000	Q86SE5	1003225	881543.9	1003225	478695.5	7	2	5	1	291	7.93	1.85	2	7.081	0	RALYL	High	High
-6945972540220550000	Q96ST3	225504.9	198133.7	225504.9	107590.4	1	1	2	1	1273	7.25	0	1	2.095	0.01	SIN3A	High	High
-303633341198715000	Q96PV6	220838.3	193946.6	220838.3	105316.8	1	1	1	1	800	9.11	0	1	2.875	0.004		High	Peak Found
5185588165699990000	P20290	5043922	4429322	5043922	2405208	6	2	4	2	206	9.38	1.95	2	3.393	0.002	BTF3	High	High
-5752760027524990000	O95470	2010916	1765838	2010916	958884.3	11	6	8	6	568	9.16	1.75	6	11.538	0	SGPL1	High	High
4532618803689770000	P00491	2740367	2405304	2740367	1306127	29	7	16	7	289	6.95	17.48	7	24.044	0	PNP	High	High

-7373318550391890000	Q13636	1063549	933482.8	1063549	506899.3	18	4	7	4	194	7.06	10.4	4	9.997	0	RAB31	High	High
-4786951349264090000	Q9P2I0	2167421	1902012	2167421	1032829	5	4	4	4	782	5.11	2.03	4	8.545	0	CPSF2	High	Peak Found
-3430961605673790000	Q13033	2071439	1817396	2071439	986881.4	5	4	4	3	797	5.36	3.92	4	10.484	0	STRN3	High	Peak Found
2951655110054190000	Q5VW32	320116.3	280812.9	320116.3	152486.9	3	1	2	1	411	7.65	0	1	3.5	0.002	BROX	High	High
-1146438711958020000	Q16851	26875451	23563999	26875451	12795710	32	16	36	16	508	8.15	31.51	16	58.025	0	UGP2	High	High
-6979633451004200000	P31946	39670912	34778392	39670912	18885343	47	16	74	6	246	4.83	130.98	16	86.975	0	YWHAB	High	High
817877645912	P05387	32460959	28453264	32460959	15450676	81	7	22	7	115	4.54	43.7	7	47.659	0	RPLP2	High	High

3090000																		
-7829088903597680000	O75976	727358.7	637326.6	727358.7	346080.7	3	3	4	3	1380	6.05	3.93	3	10.051	0	CPD	High	High
-5032092853006600000	Q13303	637167.4	558253.1	637167.4	303142.3	5	2	3	2	367	9	1.92	2	4.818	0	KCNAB2	High	High
5364683920046450000	O75340	5001328	4381026	5001328	2378983	16	3	13	3	191	5.4	5.29	3	17.08	0	PDCD6	High	High
-3757999433169180000	Q9NTJ3	12271708	10748118	12271708	5836437	16	19	35	19	1288	6.79	38.12	19	66.319	0	SMC4	High	High
2735556346422020000	P53384	522284.3	457394	522284.3	248373.8	3	1	2	1	320	5.33	3.52	1	3.369	0.002	NUBP1	High	High
7100532501288980000	P55795	4091337	3582902	4091337	1945585	25	9	32	4	449	6.3	37.42	9	51.495	0	HNRNPH2	High	High
-527718111714	P60866	42725976	37406440	42725976	20312424	23	3	25	3	119	9.94	47.68	3	31.964	0	RPS20	High	High

3700000																		
2015185769260420000	P53396	77144213	67539297	77144213	36675151	34	33	78	33	1101	7.33	74.53	33	125.421	0	ACLY	High	High
-6712994512663590000	Q4G0N4	2531670	2216141	2531670	1203408	12	5	7	5	442	8.18	1.63	5	13.748	0	NADK2; NADKD1	High	High
3973865475325370000	P15374	2378443	2081860	2378443	1130490	14	3	6	3	230	4.92	4.46	3	12.924	0	UCHL3	High	High
-2806673757180560000	P54709	11362951	9944950	11362951	5400301	18	5	16	5	279	8.35	7.69	5	14.807	0	ATP1B3	High	High
706443412800658000	O14683	1434023	1254883	1434023	681425.6	5	1	2	1	189	9.55	4.86	1	4.469	0	TP53I11	High	High
7060921209574590000	Q01780	2520266	2205414	2520266	1197583	11	9	15	9	885	8.46	19.57	9	29.364	0	EXOSC10	High	High
-16918109513615600	P51149	37889442	33139448	37889442	17995365	45	8	45	8	207	6.7	80.96	8	49.661	0	RAB7A	High	High

2142775620520920000	P15559	1.32E+08	1.15E+08	1.32E+08	62448156	32	12	57	12	274	8.88	110.37	12	64.975	0	NQO1	High	High
-8809117759024250000	P29692	1.26E+08	1.1E+08	1.26E+08	59968047	51	12	45	10	281	5.01	72.1	12	72.613	0	EEF1D	High	High
-6138498598484700000	P30405	5522234	4824913	5522234	2620022	12	4	13	4	207	9.38	10.15	4	10.451	0	PPIF	High	High
2575238413296380000	Q8IVF2	2969110	2589813	2969110	1406319	6	3	4	3	5795	5.36	5.03	3	16.992	0	AHNAK2	High	Peak Found
-5697914900789610000	Q7RTV0	2711190	2364554	2711190	1283999	25	3	7	3	110	8.41	5.88	3	10.374	0	PHF5A	High	High
-238548380464884000	Q8NEW0	2601089	2268334	2601089	1231749	6	2	6	2	376	6.95	11.28	2	6.441	0	SLC30A7	High	High
5794288011295630000	O60502	2994020	2610985	2994020	1417815	8	6	8	6	916	4.91	2.58	6	17.678	0	MGEA5	High	Peak Found

5644554619979620000	O75251	1297937	1131857	1297937	614620.3	11	3	6	3	213	9.99	4.77	3	9.077	0	NDUFS7	High	High
-7123841880797260000	P09382	2.8E+08	2.44E+08	2.8E+08	1.33E+08	61	9	47	9	135	5.5	75.2	9	59.333	0	LGALS1	High	High
-3509050462547540000	Q04917	17256161	15045223	17256161	8169849	46	16	77	10	246	4.84	125.47	16	78.837	0	YWHAH	High	High
-2537127350626280000	P49959	2951089	2572888	2951089	1397128	6	4	6	4	708	5.9	6.25	4	15	0	MRE11A; MRE11	High	High
4704340040215320000	Q9GZN8	947722.9	826228.4	947722.9	448658.1	8	1	2	1	174	6.84	4.04	1	4.271	0	C20orf27	High	High
-3764850706603640000	Q9ULC4	2341760	2040816	2341760	1108203	16	3	7	3	181	8.82	1.98	3	8.235	0	MCTS1	High	High
1156142612126300000	P07814	61699037	53761193	61699037	29193373	36	48	107	48	1512	7.33	150.56	48	214.463	0	EPRS	High	High

-469021931412063000	P53618	38374318	33426920	38374318	18151467	28	24	73	24	953	6.05	93.57	24	122.889	0	COPB1	High	High
-1665507599286510000	P62873	58257483	50717627	58257483	27540657	28	9	36	4	340	6	56.89	9	54.291	0	GNB1	High	High
-475333416762784000	P43007	441248.3	384115.4	441248.3	208582.1	2	1	1	1	532	6.25	0	1	3.446	0.002	SLC1A4	High	Peak Found
6971851119340030000	Q712K3	1296900	1128564	1296900	612831.9	5	1	2	1	238	4.42	0	1	2.117	0.01	UBE2R2	Peak Found	High
-4107495286689160000	P42566	2306092	2006556	2306092	1089599	12	9	15	9	896	4.64	3.53	9	24.106	0	EPS15	High	High
7524617474399660000	Q6DD88	35391790	30783449	35391790	16716011	31	16	34	16	541	5.66	44.37	16	61.591	0	ATL3	High	High
-5299456138050830000	O00115	1239790	1077772	1239790	585251.1	6	2	6	2	360	8.05	5.66	2	6.665	0	DNASE2	High	High

1547781430018800000	P30101	2.54E+08	2.21E+08	2.54E+08	1.2E+08	40	25	136	25	505	6.35	232.3	25	149.351	0	PDIA3	High	High
6821659264702230000	P13284	1719942	1495034	1719942	811832.4	4	1	2	1	250	4.88	2.38	1	5.664	0	IFI30	High	High
4523717261131920000	Q8NEZ4	6765386	5877605	6765386	3191654	0	1	1	1	4911	6.49	0	1	2.13	0.01	KMT2C; MLL3	Peak Found	High
5558821625919880000	Q9UDY4	3570329	3101070	3570329	1683941	12	4	8	3	337	8.5	8.98	4	15.4	0	DNAJB4	High	High
-700006474042265000	Q7Z739	4125458	3582738	4125458	1945497	8	4	7	2	585	9.04	12.7	4	14.711	0	YTHDF3	High	Peak Found
636260914267966000	Q6YP21	614803.2	533595.7	614803.2	289752.9	5	2	3	2	454	8.19	0	2	5.292	0	CCBL2; KYAT3	High	Peak Found
1231047339708810000	O00203	9365252	8126887	9365252	4413058	14	15	28	15	1094	6.04	23.88	15	44.185	0	AP3B1	High	High
528813137520318000	Q9UBG0	6204826	5384055	6204826	2923647	10	12	21	12	1479	5.83	17.74	12	40.706	0	MRC2	High	High

-3534429540462620000	P09525	17479595	15165386	17479595	8235099	31	11	29	10	319	6.13	33.85	11	39.229	0	ANXA4	High	High
8759113245393660000	O75844	6354248	5512394	6354248	2993337	16	7	11	7	475	7.49	5.66	7	18.089	0	ZMPSTE24	High	High
7245763616426790000	Q9NZ01	10983809	9527645	10983809	5173697	21	7	17	7	308	9.45	16.88	7	21.362	0	TECR	High	High
3968134916858170000	P61247	1.41E+08	1.22E+08	1.41E+08	66394894	43	15	73	15	264	9.73	78.64	15	80.266	0	RPS3A	High	High
-1414691960117410000	P33991	17204836	14920544	17204836	8102146	18	15	30	15	863	6.74	35.02	15	67.671	0	MCM4	High	High
280272277997118000	Q9Y316	1154469	1000640	1154469	543367	17	4	7	4	297	7.14	1.81	4	13.315	0	MEMO1	High	High
-5951829502980740000	Q92989	647306.2	561023	647306.2	304646.4	6	3	5	3	425	6.62	1.68	3	7.709	0	CLP1	High	Peak Found

1997849162023250000	P63208	6456220	5593049	6456220	3037134	18	4	9	4	163	4.54	13.98	4	11.601	0	SKP1	High	High
5390020710088670000	O75368	9109941	7889205	9109941	4283992	59	5	9	5	114	5.25	14.81	5	24.09	0	SH3BGRL	High	High
2256235105362560000	P62937	2.79E+08	2.42E+08	2.79E+08	1.31E+08	67	11	68	11	165	7.81	82.31	11	84.85	0	PPIA	High	High
-7564071337663160000	Q03519	2244090	1942977	2244090	1055075	5	3	4	3	686	8.02	6.32	3	9.534	0	TAP2	High	High
-8857038607970440000	Q16836	3775670	3268868	3775670	1775059	21	5	8	5	314	8.85	5.79	5	15.022	0	HADH	High	High
5177131133338510000	Q8IXM3	726819.8	629048.5	726819.8	341585.6	13	2	4	2	137	9.57	5.59	2	4.304	0	MRPL41	High	High
1992212741193310000	Q13247	14343594	12413882	14343594	6740979	15	5	16	4	344	11.43	20.86	5	23.777	0	SRSF6	High	High

-2057473982284880000	O95302	12721406	11006857	12721406	5976937	17	10	33	9	570	5.08	21.21	10	27.872	0	FKBP9	High	High
6132827760965530000	Q7Z3D6	1709154	1477611	1709154	802371.4	13	5	6	5	616	6.79	2.38	5	13.074	0	C14orf159; DGLUCY	High	High
7340038753290800000	Q9Y5B9	18137220	15670643	18137220	8509464	12	12	18	12	1047	5.66	14.33	12	38.029	0	SUPT16H	High	High
-8438939394960090000	O00116	4139988	3576812	4139988	1942278	13	7	14	7	658	7.34	11.27	7	22.058	0	AGPS	High	High
-4943228809179220000	Q8IWX8	1785522	1541851	1785522	837254.9	5	4	7	4	916	9.04	5.92	4	15.003	0	CHERP	High	High
8321925279742630000	Q9Y6Y8	2330574	2012172	2330574	1092648	8	7	12	7	1000	5.54	10.67	7	18.225	0	SEC23IP	High	High
3259999118554290000	Q16181	26337746	22737218	26337746	12346751	28	13	35	13	437	8.63	54.03	13	67.856	0	SEPT7	High	High

-8215909947984890000	P27816	34594570	29857423	34594570	16213161	33	25	61	25	1152	5.43	76.83	25	123.952	0	MAP4	High	High
-7576515921520270000	P13667	88917077	76710835	88917077	41655475	51	32	80	32	645	5.07	124.41	32	139.775	0	PDIA4	High	High
-1817111684576730000	O15511	18923097	16313990	18923097	8858814	34	4	13	4	151	5.67	17.35	4	16.321	0	ARPC5	High	High
5740935786864810000	Q9BRX2	1940244	1672594	1940244	908251.1	18	6	10	6	385	6.34	8.68	6	18.588	0	PELO	High	High
-7328219287373480000	Q7LG56	550626.3	474649.1	550626.3	257743.7	12	4	9	1	351	4.97	5.2	4	11.106	0	RRM2B	High	Peak Found
3035595102958390000	P46777	56425650	48630054	56425650	26407065	40	14	54	14	297	9.72	57.6	14	78.819	0	RPL5	High	High
-700049213662343000	P06396	23739438	20455237	23739438	11107591	20	13	31	13	782	6.28	33.47	13	53.537	0	GSN	High	High

-1427693447118430000	P60468	17608663	15172317	17608663	8238863	22	2	8	2	96	11.56	7.76	2	6.976	0	SEC61B	High	High
6929162783006900000	P20042	13373291	11514747	13373291	6252731	23	9	15	9	333	5.8	33.56	9	42.229	0	EIF2S2	High	High
4549685978582370000	Q9Y399	857710.6	738505.3	857710.6	401022.7	6	2	4	2	296	9.26	3.9	2	5.762	0	MRPS2	High	High
-6037717816811940000	Q5T8P6	662637.4	570521.8	662637.4	309804.4	2	2	2	2	1007	9.16	0	2	5.404	0	RBM26	High	Peak Found
5588374002070680000	P63000	18295252	15743183	18295252	8548855	28	6	15	6	192	8.5	20.14	6	19.455	0	RAC1	High	High
-3779865839115360000	P00367	68027514	58518442	68027514	31776652	46	20	69	20	558	7.8	117.18	20	120.088	0	GLUD1	High	High
-4483992310026690000	P28300	3600877	3096917	3600877	1681686	6	2	6	2	417	8.09	9.1	2	8.377	0	LOX	High	High

6560846777174150000	P53621	1.06E+08	91405539	1.06E+08	49634985	39	44	117	44	1224	7.66	174.96	44	209.941	0	COPA	High	High
2120449807322370000	Q9Y2Z0	2287842	1967339	2287842	1068304	18	5	7	5	365	5.16	7.12	5	15.778	0	SUGT1	High	High
-5616510837335030000	P15586	15659108	13462112	15659108	7310189	11	6	11	6	552	8.31	17.43	6	21.838	0	GNS	High	High
905993394095765000	Q96S52	2264197	1946458	2264197	1056965	11	4	5	4	555	6.49	1.68	4	10.933	0	PIGS	High	High
-4424001123554830000	Q96CW1	31229714	26834521	31229714	14571667	33	13	29	13	435	9.54	27.21	13	45.354	0	AP2M1	High	High
-938207872866175000	Q00534	711030.9	610921.4	711030.9	331742.2	12	3	6	2	326	6.46	1.69	3	8.089	0	CDK6	High	Peak Found
8835450799000800000	Q8N1F7	16969282	14571133	16969282	7912409	26	22	52	22	819	5.72	37.24	22	68.959	0	NUP93	High	High
-820540277266	P47895	10765743	9244188	10765743	5019774	29	14	33	12	512	7.25	30.12	14	56.703	0	ALDH1A3	High	High

0810000																		
5250221047475220000	Q7LBC6	558645.2	479676.3	558645.2	260473.5	1	2	2	2	1761	7.18	2.06	2	5.34	0	KDM3B	High	Peak Found
-2013502918217230000	O14929	7241157	6216774	7241157	3375829	13	5	16	5	419	5.69	16.01	5	19.682	0	HAT1	High	High
5209638232977490000	Q13641	3513685	3015347	3513685	1637392	7	3	4	3	420	6.83	0	3	6.738	0	TPBG	High	High
-1143885462092760000	Q8N2K0	1505413	1291464	1505413	701290	14	5	6	5	398	8.65	3.89	5	13.692	0	ABHD12	High	High
5430220103658380000	Q969Q0	441113.6	378401.4	441113.6	205479.3	16	2	5	1	106	10.65	10.6	2	5.723	0	RPL36AL	High	Peak Found
17737275167797300	Q8NE86	708795.4	608010.8	708795.4	330161.7	3	1	2	1	351	8.65	1.73	1	3.204	0.002	MCU	High	Peak Found
-6701833234650980000	O43865	14863634	12745433	14863634	6921018	20	11	33	5	530	6.89	30.46	11	43.852	0	AHCYL1	High	High

5634853001095680000	Q9UBM7	16710740	14322038	16710740	7777145	15	6	10	6	475	8.7	20.42	6	25.377	0	DHCR7	High	High
7751322844419770000	P33121	4313719	3695401	4313719	2006675	11	7	13	7	698	7.15	10.08	7	22.727	0	ACSL1	High	High
3425744660855700000	O60568	33219069	28456658	33219069	15452519	23	15	42	15	738	6.05	40.35	15	46.737	0	PLOD3	High	High
7235259727744770000	Q7Z4W1	3805089	3259372	3805089	1769902	21	5	13	5	244	8.1	18.73	5	18.933	0	DCXR	High	High
-8785242188080330000	P28838	16809512	14395974	16809512	7817294	32	14	38	14	519	7.93	52.55	14	67.065	0	LAP3	High	High
-238819402127130000	P45877	4566802	3909645	4566802	2123013	10	2	6	2	212	8.4	12.51	2	8.655	0	PPIC	High	High
3269209104434090000	P40227	83004994	71027241	83004994	38569173	32	19	58	19	531	6.68	87.76	19	79.618	0	CCT6A	High	High
-496978519951	Q13098	8589119	7348218	8589119	3990225	21	9	18	9	491	6.74	16.94	9	30.003	0	GPS1	High	High

8400000																		
2015286030572760000	P24752	15951687	13642749	15951687	7408278	38	13	21	13	427	8.85	24.84	13	51.277	0	ACAT1	High	High
5949568928941640000	O43676	499494.4	427162.7	499494.4	231957.7	11	1	2	1	98	9.2	0	1	2.191	0.009	NDUFB3	High	High
-6826541305752520000	P23381	12418791	10618309	12418791	5765948	18	9	25	9	471	6.23	38.67	9	45.768	0	WARS	High	High
-4711793945183500000	P23434	540270.3	461882.7	540270.3	250811.3	6	1	3	1	173	4.88	3.35	1	3.432	0.002	GCSH	High	High
4143955501491180000	P55084	43156067	36891831	43156067	20032981	32	14	35	14	474	9.41	36.65	14	51.354	0	HADHB	High	High
4636367879172640000	P24666	3204065	2737242	3204065	1486376	16	4	9	4	158	6.74	13.59	4	20.487	0	ACP1	High	High
-153878497684	Q8N5C6	639787.6	546319.4	639787.6	296662	2	2	4	2	995	8.72	2.05	2	7.342	0	SRBD1	High	High

1210000																		
5778599060552410000	P52272	59885152	51135869	59885152	27767771	40	28	85	28	730	8.7	119.21	28	119.394	0	HNRNPM	High	High
6965600281876420000	P46063	31324574	26747590	31324574	14524462	24	14	31	14	649	7.88	25.1	14	47.595	0	RECQL	High	High
-1437948072660630000	O60664	49417138	42172954	49417138	22900734	49	15	49	15	434	5.44	88.64	15	83.611	0	PLIN3	High	High
3951923410761950000	Q9UQ80	55423225	47292782	55423225	25680900	46	19	62	19	394	6.55	73.82	19	78.607	0	PA2G4	High	High
-2598946853791070000	Q9Y295	11802120	10064967	11802120	5465473	26	7	10	7	367	8.9	11.49	7	24.241	0	DRG1	High	High
8964751153975720000	Q96RE7	380906.6	324711.9	380906.6	176324.9	2	1	1	1	527	5.74	0	1	2.591	0.006	NACC1	High	Peak Found
-698968614531	O00483	2812893	2397754	2812893	1302027	37	3	6	3	81	9.38	7.81	3	11.909	0	NDUFA4	High	High

8520000																		
-1233301175711810000	P21926	5091360	4339575	5091360	2356473	12	3	8	3	228	7.15	5.87	3	9.235	0	CD9	High	High
3703372283530200000	Q9NP97	2206787	1878709	2206787	1020175	34	2	5	2	96	7.25	6.41	2	10.323	0	DYNLRB1	High	High
2564632356119290000	P04843	90320740	76869705	90320740	41741744	38	22	71	22	607	6.38	114.26	22	110.766	0	RPN1	High	High
7072952226669660000	P08648	10328033	8789335	10328033	4772780	9	9	17	9	1049	5.77	20.64	9	29.379	0	ITGA5	High	High
9112422287150270000	Q96LJ7	638715.3	543161.4	638715.3	294947.2	8	2	4	2	313	7.83	1.74	2	6.701	0	DHRS1	High	High
6270527551906760000	P07951	9403424	7996046	9403424	4342009	41	16	41	2	284	4.7	72.46	16	76.936	0	TPM2	High	High
-2262993958771160000	P40926	1.22E+08	1.04E+08	1.22E+08	56234993	53	15	60	15	338	8.68	112.61	15	78.752	0	MDH2	High	High

-2063418146080870000	O76094	5982870	5083886	5982870	2760649	15	10	14	10	671	9.26	16.71	10	32.207	0	SRP72	High	High
-3169952320646040000	O43426	884876	751900.1	884876	408296.4	2	3	5	3	1573	7.42	0	3	7.369	0	SYNJ1	High	High
8369123714013870000	Q07065	1E+08	85071290	1E+08	46195364	52	27	85	27	602	5.92	153.63	27	146.602	0	CKAP4	High	High
6509099056822070000	Q02978	8918799	7572378	8918799	4111948	32	10	33	10	314	9.91	30.76	10	33.11	0	SLC25A11	High	High
-2220573577254260000	Q96A33	6333707	5377494	6333707	2920084	18	6	11	6	483	4.87	8.8	6	28.052	0	CCDC47	High	High
-6199563702112540000	Q86XP3	2655980	2254388	2655980	1224176	4	3	3	3	938	7.02	0	3	7.817	0	DDX42	High	Peak Found
2564730176707540000	O95479	2662585	2259887	2662585	1227163	9	7	11	7	791	7.3	17.32	7	24.316	0	H6PD	High	High

-5372925708799750000	Q15046	23660840	20080866	23660840	10904301	28	20	41	20	597	6.35	39.94	20	66.487	0	KARS	High	High
2402053168044670000	P06576	1.99E+08	1.69E+08	1.99E+08	91705529	45	19	116	19	529	5.4	209.1	19	146.517	0	ATP5B	High	High
4445906156822000000	Q969V3	12962472	10995383	12962472	5970707	28	13	31	13	563	6.89	41.27	13	68.951	0	NCLN	High	High
-3659608677254820000	P53582	1386177	1175146	1386177	638126.9	7	2	5	2	386	7.17	6.24	2	11.534	0	METAP1	High	High
-27127465578060100	Q96SU4	1772948	1502605	1772948	815944	5	3	8	3	736	6.18	6.24	3	12.134	0	OSBPL9	High	High
3880920923163150000	P35222	13871389	11750965	13871389	6381002	26	16	41	14	781	5.86	55.3	16	63.905	0	CTNNB1	High	High
-951770295276680000	P09874	11289126	9561988	11289126	5192345	18	15	31	15	1014	8.88	23.87	15	53.798	0	PARP1	High	High

3107589471447250000	Q9NV06	533436.5	451718.8	533436.5	245292.1	4	2	3	2	445	9.19	0	2	3.753	0.001	DCAF13	High	Peak Found
7211345083341150000	Q15382	4359529	3690726	4359529	2004136	34	6	12	6	184	5.92	6.87	6	18.908	0	RHEB	High	High
-6790579379401260000	Q8IU85	847688.5	717147.3	847688.5	389424.9	2	1	2	1	385	7.21	0	1	2.363	0.008	CAMK1D	High	High
-5329658274444490000	O95394	7849621	6635091	7849621	3602983	16	8	12	8	542	6.25	12.37	8	22.416	0	PGM3	High	High
-2657182436335150000	Q9Y3I0	29448191	24888175	29448191	13514763	31	14	42	14	505	7.23	40	14	57.682	0	C22orf28; RTCB	High	High
8961190954250980000	Q16891	13783676	11648562	13783676	6325395	17	12	26	12	758	6.48	35.69	12	51.851	0	IMMT	High	High
-8494300980345170000	P48059	3844961	3248991	3844961	1764265	11	3	9	3	325	8.05	12.46	3	13.464	0	LIMS1	High	High

560931000169040000	P42167	960844.7	811753.4	960844.7	440797.9	17	6	7	2	454	9.38	7.04	6	16.758	0	TMPO	High	High
2874885037648560000	O60869	1983482	1675454	1983482	909804.2	10	1	5	1	148	9.95	10.78	1	15.019	0	EDF1	High	High
-1416539979039470000	Q99426	19527989	16495188	19527989	8957208	33	8	22	8	244	5.15	33.94	8	29.514	0	TBCB	High	High
-5466542288326950000	P59998	42269722	35699456	42269722	19385499	28	5	22	5	168	8.43	32.98	5	22.468	0	ARPC4	High	High
6307111791221070000	P41221	1150851	971853.2	1150851	527735.2	11	4	5	4	380	8.46	0	4	11.593	0	WNT5A	High	Peak Found
2638301692080510000	O95817	2800981	2365265	2800981	1284385	11	8	15	8	575	6.95	16.05	8	27.688	0	BAG3	High	High
8117017983706300000	P27348	1.65E+08	1.39E+08	1.65E+08	75565615	48	18	88	11	245	4.78	181.44	18	120.075	0	YWHAQ	High	High
-813585421492	Q9BY44	8991181	7589325	8991181	4121151	25	12	28	12	585	8.87	21.25	12	34.636	0	EIF2A	High	High

2220000																		
1964707792463710000	O43776	33373616	28168300	33373616	15295935	24	13	39	13	548	6.25	37.71	13	46.334	0	NARS	High	High
-2173979595876030000	Q9Y4I1	854392	720913.6	854392	391470.1	3	4	5	4	1855	8.48	3.58	4	12.416	0	MYO5A	High	High
5165886701298410000	Q15459	23619306	19927723	23619306	10821141	22	16	34	16	793	5.22	48.32	16	63.545	0	SF3A1	High	High
2805084755290840000	Q09161	3811844	3215896	3811844	1746294	8	5	10	5	790	6.43	10.44	5	23.336	0	NCBP1	High	High
-7073830227573610000	Q9NR45	6159434	5195058	6159434	2821017	18	5	11	5	359	6.74	17.52	5	28.573	0	NANS	High	High
1985777424174470000	Q14669	1862050	1570436	1862050	852777.3	4	6	8	6	1992	8.48	5.45	6	18.131	0	TRIP12	High	High
677526654360538000	Q99733	14426606	12165658	14426606	6606189	15	6	22	4	375	4.69	24.81	6	32.792	0	NAP1L4	High	High

-3195090244308710000	P12111	3.21E+08	2.71E+08	3.21E+08	1.47E+08	29	89	271	89	3177	6.68	433.37	89	412.369	0	COL6A3	High	High
-5725917965276370000	O95361	2184236	1841023	2184236	999711.4	7	4	5	4	564	5.49	3.68	4	9.37	0	TRIM16	High	High
3320992659371110000	Q15392	15048586	12683803	15048586	6887552	15	8	26	8	516	8.16	23.09	8	20.777	0	DHCR24	High	High
3819136565778510000	P13639	6.85E+08	5.77E+08	6.85E+08	3.14E+08	43	44	274	43	858	6.83	497.34	44	249.978	0	EEF2	High	High
8590032029601750000	O75935	10378165	8744282	10378165	4748315	23	5	10	5	186	5.47	10.8	5	13.824	0	DCTN3	High	High
1944955918091710000	Q8IVL6	1493507	1258078	1493507	683160.9	8	4	10	4	736	6.32	4.53	4	16.163	0	LEPREL2; P3H3	High	High
5347375933895690000	P06737	33606998	28308238	33606998	15371924	20	17	31	12	847	7.17	22.74	17	54.566	0	PYGL	High	High

3792046426100680000	O43395	996176.7	838818.4	996176.7	455494.7	4	3	3	3	683	9.5	1.87	3	6.43	0	PRPF3	High	Peak Found
-7970699774180830000	Q9Y230	57157141	48124470	57157141	26132523	41	19	56	19	463	5.64	108.2	19	88.742	0	RUVBL2	High	High
361994698644895000	P07355	6.7E+08	5.64E+08	6.7E+08	3.06E+08	64	29	166	29	339	7.75	287.61	29	167.758	0	ANXA2	High	High
-3259776130493610000	Q6PI48	1033212	869549.4	1033212	472182.2	4	3	4	3	645	8.02	0	3	8.186	0	DARS2	High	High
6576828257658510000	Q9BQE3	6.57E+08	5.53E+08	6.57E+08	3E+08	49	21	174	8	449	5.1	297.97	21	154.655	0	TUBA1C	High	High
487381900913570000	Q9H8Y8	2268104	1908665	2268104	1036442	7	3	5	3	452	4.82	4.38	3	7.786	0	GORASP2	High	High
2291665734159650000	P62310	1505941	1267263	1505941	688148.4	12	1	4	1	102	4.7	6.8	1	3.057	0.003	LSM3	High	High
964900113043376000	Q9Y265	43616888	36696701	43616888	19927022	50	18	55	18	456	6.42	81.21	18	78.572	0	RUVBL1	High	High

846923677132301000	Q9UBS8	351543.9	295749.9	351543.9	160598	2	1	1	1	474	4.75	0	1	3.297	0.002	RNF14	High	Peak Found
7312941423538820000	P31350	11161455	9389835	11161455	5098863	31	11	30	8	389	5.38	37.99	11	45.844	0	RRM2	High	High
-3957617844882620000	Q7Z3B4	1603001	1348431	1603001	732224.3	5	3	6	3	507	7.02	4.65	3	7.74	0	NUP54	High	High
-4501130865097890000	O60763	18637289	15675797	18637289	8512262	24	20	40	20	962	4.91	58.26	20	78.24	0	USO1	High	High
6073254106150690000	Q99575	14332659	12053847	14332659	6545473	23	20	44	20	1024	9.22	58.24	20	78.874	0	POP1	High	High
6918046484963300000	Q9NX47	854800	718884.8	854800	390368.4	8	2	5	2	278	8.7	2.76	2	6.475	0	MARCH5	High	High
-6440689549983680000	Q9Y5M8	10249904	8619774	10249904	4680705	25	7	9	7	271	9.04	7.14	7	16.779	0	SRPRB	High	High
303868984056	Q8WWM7	5557464	4673588	5557464	2537849	6	7	10	7	10	8.59	5.56	7	18.357	0	ATXN2L	High	High

3550000										75								
-1511702505276580000	Q12931	62677756	52707000	62677756	28620926	29	19	46	18	704	8.21	54.93	19	79.119	0	TRAP1	High	High
2247391639658700000	P36639	1242326	1044639	1242326	567259	20	3	7	3	197	5.27	11.48	3	13.447	0	NUDT1	High	High
6289921617317730000	Q9BWF3	4743818	3987330	4743818	2165198	18	6	11	6	364	7.08	13.25	6	23.85	0	RBM4	High	High
-1764632123327770000	P46060	23996783	20168905	23996783	10952108	27	16	40	16	587	4.68	59.21	16	71.073	0	RANGAP1	High	High
-7652533075202520000	Q92734	19908309	16731039	19908309	9085279	29	8	18	8	400	5.1	30.58	8	51.757	0	TFG	High	High
-2508313563334930000	O75027	3429348	2880611	3429348	1564228	7	5	9	5	752	9.33	3.97	5	17.515	0	ABCB7	High	High
251615080073	P55011	5648283	4744399	5648283	2576301	13	12	16	12	12	6.4	24.33	12	45.659	0	SLC12A2	High	High

3110000										12								
3583580330514190000	P55809	29221427	24542163	29221427	13326872	30	15	31	15	520	7.46	37.75	15	63.907	0	OXCT1	High	High
5339836283997490000	O95163	1288908	1082416	1288908	587772.9	7	6	7	6	1332	5.94	6.59	6	20.294	0	IKBKAP; ELP1	High	High
5219092479354340000	O94855	9090053	7632855	9090053	4144788	13	11	18	11	1032	7.25	21.92	11	39.636	0	SEC24D	High	High
-507311622488501000	Q9Y2G5	4386491	3682685	4386491	1999769	11	4	9	4	429	6.6	14.79	4	16.705	0	POFUT2	High	High
8999845406277560000	Q9H089	3224207	2705655	3224207	1469223	4	3	3	3	658	6.38	0	3	5.639	0	LSG1	High	High
-8796563562417500000	P51532	4896009	4106325	4896009	2229814	7	10	13	6	1647	7.88	8.17	10	34.972	0	SMARCA4	High	High
-9088967002815650000	P26641	1.71E+08	1.43E+08	1.71E+08	77789614	34	19	73	19	437	6.67	109.82	19	100.094	0	EEF1G	High	High

-5625236690753070000	P22061	25720076	21558298	25720076	11706575	47	9	16	9	227	7.21	29.26	9	42.738	0	PCMT1	High	High
-7755566090060090000	P35914	645662.5	541019.4	645662.5	293784	8	2	3	2	325	8.54	3.92	2	8.128	0	HMGCL	High	High
-2654300565608210000	P07711	420001.2	351752.5	420001.2	191008.4	7	2	2	2	333	5.45	0	2	4.699	0	CTSL; CTSL1	High	Peak Found
4041474047181680000	P58335	944341.8	790723.2	944341.8	429378.1	5	2	5	1	489	7.46	5.92	2	9.456	0	ANTXR2	High	High
8321429417116910000	P07339	46061854	38562553	46061854	20940216	29	10	32	10	412	6.54	52.55	10	36.643	0	CTSD	High	High
-3292354779633860000	A5YKK6	4037417	3380013	4037417	1835413	5	9	9	9	2376	7.11	10.68	9	26.796	0	CNOT1	High	High
896567990825277000	O94925	10972200	9183761	10972200	4986961	20	11	34	11	669	7.77	38.53	11	40.872	0	GLS	High	High

-3815266739088280000	P49419	39463528	33021769	39463528	17931463	37	18	45	18	539	7.99	58.79	18	75.406	0	ALDH7A1	High	High
6744792230054970000	O15173	11631933	9731594	11631933	5284445	33	8	30	7	223	4.88	27.08	8	32.438	0	PGRMC2	High	High
-5236519405045950000	P19022	2075751	1736290	2075751	942839.3	4	3	11	3	906	4.81	6.51	3	12.687	0	CDH2	High	High
3676506683142230000	Q9NZN3	4176989	3493747	4176989	1897172	14	8	15	2	535	6.57	11.47	8	25.801	0	EHD3	High	Peak Found
8905060599663970000	Q9Y5K8	2382065	1992420	2382065	1081923	12	3	11	3	247	9.36	5.36	3	11.469	0	ATP6V1D	High	High
2504854661788260000	O75787	443173.4	370552.5	443173.4	201217.2	2	1	2	1	350	6.1	0	1	2.469	0.007	ATP6AP2	High	Peak Found
-8151909637929930000	Q14554	27448501	22949466	27448501	12462006	31	16	47	16	519	7.91	73.77	16	82.378	0	PDIA5	High	High

7405227159340640000	P62195	14831441	12397183	14831441	6731912	34	12	27	11	406	7.55	34.43	12	46.053	0	PSMC5	High	High
-8775714319667420000	Q14914	6507918	5439190	6507918	2953586	24	8	14	8	329	8.29	18.47	8	33.443	0	PTGR1	High	High
7165899401853600000	P04424	1408729	1177065	1408729	639169.3	8	4	5	4	464	6.48	1.88	4	7.641	0	ASL	High	High
-849081801659546000	P30533	9188639	7675373	9188639	4167877	29	10	24	10	357	8.78	26.58	10	41.683	0	LRPAP1	High	High
6178483006887400000	Q8TCT9	11996670	10019041	11996670	5440534	15	4	29	4	377	6.43	19.09	4	14.712	0	HM13	High	High
1210057456196530000	Q9Y276	160025.8	133560.6	160025.8	72526.01	2	1	2	1	419	8.5	0	1	2.339	0.008	BCS1L	High	Peak Found
-2591250137459750000	O43252	11288602	9421281	11288602	5115939	19	10	20	10	624	6.86	29.26	10	35.495	0	PAPSS1	High	High

4804495115062900000	P41252	44413944	37065333	44413944	20127197	25	29	85	29	1262	6.15	112.18	29	116.148	0	IARS	High	High
-1870726190365740000	P61160	29594084	24693032	29594084	13408796	26	10	26	10	394	6.74	36.84	10	47.194	0	ACTR2	High	High
4832691486978410000	Q5BJH7	1497332	1248860	1497332	678155.1	12	2	6	2	314	9.16	2.66	2	15.362	0	YIF1B	High	High
3629072451512350000	P52701	8613395	7182979	8613395	3900497	12	14	22	14	1360	6.9	21.39	14	50.419	0	MSH6	High	High
-8011700536364290000	Q8TBQ9	2511933	2094685	2511933	1137455	13	1	5	1	72	8.95	5.54	1	2.232	0.009	TMEM167A	High	High
-4375562830836440000	Q15386	832963.5	694499.9	832963.5	377127	3	3	5	3	1083	6.71	5.96	3	10.952	0	UBE3C	High	High
-2820627768769540000	P51571	22557449	18801475	22557449	10209567	25	4	14	4	173	6.15	28.19	4	23.441	0	SSR4	High	High

7174373993236330000	Q15233	1.92E+08	1.6E+08	1.92E+08	87015937	38	21	58	19	471	8.95	83.06	21	91.063	0	NONO	High	High
5017693853216970000	Q969S3	345892.4	288023.3	345892.4	156402.3	3	1	1	1	477	6.15	1.67	1	3.942	0	ZNF622	High	Peak Found
-7032726270839270000	P27144	9483140	7895524	9483140	4287423	43	9	23	9	223	8.4	29.11	9	32.412	0	AK4; LOC100507855	High	High
-5515893546974730000	E9PRG8	306771.3	255322.4	306771.3	138645	11	1	5	1	123	11.53	6.18	1	4.164	0		High	High
-7619751379045490000	P04062	5196091	4323410	5196091	2347696	13	7	12	7	536	7.61	9.12	7	22.375	0	GBA	High	High
753058757647153000	P61803	8611212	7163950	8611212	3890164	16	2	5	2	113	7.08	0	2	6.301	0	DAD1	High	High
1853475733980270000	P17858	24485367	20364681	24485367	11058418	27	18	57	14	780	7.5	89.77	18	86.766	0	PFKL	High	High

-9142633453092600000	P63104	9.45E+08	7.86E+08	9.45E+08	4.27E+08	61	21	112	13	245	4.79	193.65	21	148.885	0	YWHAZ	High	High
2749950876556130000	P0DMV9	1.45E+08	1.2E+08	1.45E+08	65374237	36	26	95	22	641	5.66	141.23	26	134.46	0	HSPA1B; HSPA1A	High	High
-3614500297026830000	Q9Y266	48596194	40394148	48596194	21934808	46	16	44	16	331	5.38	40.31	16	66.852	0	NUDC	High	High
-5986296192720160000	Q9Y617	25106720	20866307	25106720	11330810	36	13	25	13	370	7.66	45.07	13	52.611	0	PSAT1	High	High
-9147063981897790000	P04632	18550687	15411718	18550687	8371795	19	5	22	5	268	5.2	48.12	5	32.173	0	CAPNS1	High	High
-93919318191628600	Q92499	33080286	27491581	33080286	14928463	27	19	44	19	740	7.23	65.25	19	77.75	0	DDX1	High	High
5048841209035300000	Q9H845	5497878	4568674	5497878	2480879	15	8	17	8	621	7.96	10.2	8	25.881	0	ACAD9	High	High

4060364465058460000	Q15435	4402858	3658660	4402858	1986724	16	7	10	7	360	4.91	5.68	7	17.611	0	PPP1R7	High	High
-6665456693960710000	Q86V88	268703.4	223269.9	268703.4	121239.9	7	1	2	1	176	6.39	1.8	1	2.758	0.004	MDP1	High	Peak Found
-62154573745771900	Q9NRG9	2560169	2126447	2560169	1154702	6	3	11	3	546	7.5	4.88	3	9.441	0	AAAS	High	High
-1566971242327950000	Q13308	4233549	3515938	4233549	1909223	7	7	10	7	1070	7.09	8.41	7	20.492	0	PTK7	High	High
1626787301321380000	Q6NUK1	11202939	9303384	11202939	5051918	24	10	16	10	477	6.33	19.21	10	26.605	0	SLC25A24	High	High
3977916920798510000	Q9Y6A9	338425	281039.8	338425	152610.1	7	1	1	1	169	8.72	0	1	2.751	0.004	SPCS1	High	Peak Found
-4243510454319930000	P33992	16754615	13913215	16754615	7555146	29	18	34	18	734	8.37	40.05	18	55.511	0	MCM5	High	High

-3240734639385880000	P53004	11632646	9656905	11632646	5243887	33	9	18	9	296	6.44	13.5	9	25.434	0	BLVRA	High	High
-3317416548883550000	P00533	11080712	9193051	11080712	4992005	17	17	27	17	1210	6.68	25.04	17	58.82	0	EGFR	High	High
8587567595163410000	Q9UIQ6	3701698	3071040	3701698	1667635	5	5	8	5	1025	5.73	4.76	5	14.471	0	LNPEP	High	High
6565186831187670000	P05976	5755682	4774930	5755682	2592880	12	2	4	2	194	5.03	5.93	2	6.731	0	MYL1	High	High
-1343514979661680000	P22234	26145932	21680892	26145932	11773146	28	12	36	12	425	7.23	49.54	12	64.476	0	PAICS	High	High
-5939378456610210000	O95819	5098700	4227534	5098700	2295633	8	10	17	10	1239	7.46	15.07	10	34.798	0	MAP4K4	High	High
-182822964944	P31153	35977026	29827692	35977026	16197017	39	15	42	15	395	6.48	45.93	15	57.647	0	MAT2A	High	High

7770000																		
-8337602067628070000	O00159	31562992	26161506	31562992	14206207	19	20	54	20	1063	9.41	71.51	20	76.032	0	MYO1C	High	High
-8139328666383470000	Q8IZH2	1748222	1448928	1748222	786796.2	5	7	7	7	1706	7.21	4.31	7	19.334	0	XRN1	High	High
3546401136209870000	O75821	8088922	6703525	8088922	3640145	23	6	9	6	320	6.13	16.09	6	26.213	0	EIF3G	High	High
5779519367271370000	Q15645	21027001	17419646	21027001	9459206	37	14	40	14	432	6.09	61.12	14	60.862	0	TRIP13	High	High
6167412320153060000	P35613	12839149	10636419	12839149	5775782	26	8	21	8	385	5.66	27.76	8	36.12	0	BSG	High	High
-5608231469546370000	O00161	565922.5	468770.5	565922.5	254551.5	13	2	3	2	211	5.01	3.83	2	7.675	0	SNAP23	High	High
702949377974	Q7L1Q6	43437128	35976037	43437128	19535688	29	15	42	11	419	5.92	53.87	15	45.732	0	BZW1	High	High

6440000																		
4461868117343580000	Q66K14	1187348	983345.9	1187348	533975.9	2	2	4	2	1250	5.25	2.29	2	6.318	0	TBC1D9B	High	High
-2806048333038510000	Q96FQ6	1901881	1574751	1901881	855120.5	27	3	4	3	103	6.79	5.46	3	12.695	0	S100A16	High	High
7171798116455750000	O15258	4839305	4006761	4839305	2175749	9	2	4	2	196	9.54	6.01	2	5.989	0	RER1	High	High
-3055099900900340000	Q9P0M9	545725.4	451678.3	545725.4	245270.1	7	1	2	1	148	10.42	1.65	1	3.8	0.001	MRPL27	High	High
-3154605407471030000	Q70UQ0	9585835	7929482	9585835	4305863	16	6	15	6	350	9.17	23.34	6	22.693	0	IKBIP	High	High
-3841868444462990000	P43155	3119932	2579741	3119932	1400850	9	6	11	6	626	8.44	3.31	6	14.475	0	CRAT	High	High

-1845786618234340000	P55263	4529453	3744568	4529453	2033373	17	6	14	6	362	6.7	13.09	6	14.098	0	ADK	High	High
-2586862151232690000	P07237	2.96E+08	2.45E+08	2.96E+08	1.33E+08	57	38	137	38	508	4.87	220.23	38	191.854	0	P4HB	High	High
-8787023427386060000	P53365	2618805	2164031	2618805	1175111	10	3	3	3	341	6.04	4.85	3	11.246	0	ARFIP2	High	Peak Found
7179197342339510000	P52907	1.92E+08	1.59E+08	1.92E+08	86121012	55	10	29	8	286	5.69	47.28	10	59.509	0	CAPZA1	High	High
-4100918330742860000	Q96GK7	693210.5	572650.7	693210.5	310960.5	10	2	4	2	314	8.24	4.47	2	8.124	0	FAHD2A	High	High
5049018417817340000	P09497	8186505	6761267	8186505	3671500	15	4	9	4	229	4.64	12.4	4	12.276	0	CLTB	High	High
-697394183825	P56192	16524319	13644600	16524319	7409283	25	17	34	17	900	6.16	41.48	17	70.182	0	MARS	High	High

5410000																		
-5793722554403520000	Q14764	87363752	72095965	87363752	39149511	35	30	101	30	893	5.48	138.92	30	161.902	0	MVP	High	High
854871700707894000	Q9NZL4	3970303	3275998	3970303	1778930	14	4	6	4	359	5.21	2.71	4	10.348	0		High	High
3927890985374290000	P11586	44270699	36509781	44270699	19825521	30	29	63	29	935	7.3	88.62	29	112.321	0		High	High
-3785691322693480000	O95834	1229422	1013894	1229422	550564.2	4	2	3	2	649	6.32	2.8	2	15.011	0	EML2	High	Peak Found
124053754926249000	Q9UBF2	2839142	2340503	2839142	1270939	7	6	8	4	871	5.81	6.39	6	18.433	0	COPG2	High	High
3042753504910150000	P13804	7383929	6085977	7383929	3304804	18	5	11	5	333	8.38	10.29	5	16.747	0	ETFA	High	High
-7680692845404350000	P30085	13683486	11276320	13683486	6123261	47	7	14	7	196	5.57	23.48	7	38.906	0	CMPK1	High	High

-7480340352347870000	Q99459	840367.1	692505.1	840367.1	376043.7	3	3	4	3	802	8.18	1.95	3	6.277	0	CDC5L	High	High
-3959572878681580000	P17987	82362992	67863978	82362992	36851460	47	24	83	24	556	6.11	124.63	24	119.521	0	TCP1	High	High
-3472169871785600000	P61009	6059829	4992873	6059829	2711227	18	3	8	3	180	8.62	8.98	3	12.999	0	SPCS3	High	High
3499849658499640000	Q9UHQ9	1185928	977105	1185928	530587	7	2	3	2	305	9.38	4.6	2	8.094	0	CYB5R1	High	High
-8985858541616020000	Q7L5N7	3648799	3005453	3648799	1632019	9	5	6	5	544	6.55	7.16	5	17.372	0	LPCAT2	High	High
5964536598066080000	P47756	45771302	37693786	45771302	20468459	51	13	40	13	277	5.59	76.11	13	73.794	0	CAPZB	High	High
-261941695740	P49321	46095775	37948493	46095775	20606769	12	8	20	8	788	4.3	28.26	8	40.152	0	NASP	High	High

5680000																		
-160533791377362000	P07195	2.05E+08	1.69E+08	2.05E+08	91509642	45	19	95	17	334	6.05	139.72	19	111.196	0	LDHB	High	High
-45443448741042800	P24928	3375800	2776076	3375800	1507463	3	4	6	4	1970	7.37	3.86	4	10.155	0	POLR2A	High	High
6335847497915130000	Q9BTY7	1379449	1133667	1379449	615603.4	8	3	5	3	390	4.81	5.59	3	9.48	0	FAM203B; FAM203A; HGH1	High	High
-5703778072310230000	Q9Y6I3	1151226	945870.9	1151226	513626.3	7	3	3	3	576	4.83	2.33	3	10.462	0	EPN1	High	High
-3283541064302850000	O75533	31770089	26102942	31770089	14174405	27	29	65	29	1304	7.09	66.9	29	116.054	0	SF3B1	High	High
5567026577194710000	Q9Y2Z9	343121.8	281857.5	343121.8	153054.1	3	1	2	1	468	7.3	2.5	1	5.696	0	COQ6	High	High
906200433272310000	Q8NBS9	60800710	49942050	60800710	27119504	34	15	34	15	432	5.97	39.24	15	63.239	0	TXNDC5	High	High

-8717511238945880000	Q12769	1480790	1215941	1480790	660279.6	2	3	6	3	1436	5.5	1.78	3	7.14	0	NUP160	High	High
-5375173174660800000	Q16698	43841965	36000390	43841965	19548912	44	13	30	13	335	9.28	51.71	13	59.966	0	DECR1	High	High
6115802108714270000	P78330	1611833	1322300	1611833	718034.7	15	3	4	3	225	5.69	4.12	3	12.315	0	PSPH	High	High
-7999052709415310000	Q9P0V9	3373607	2767487	3373607	1502799	9	4	8	4	454	6.8	4.71	4	15.639	0	SEPT10	High	High
-7722253779426460000	O00571	9603131	7875324	9603131	4276454	16	10	27	9	662	7.18	47.04	10	41.323	0	DDX3X	High	High
-5869916031675070000	O95486	3178312	2606326	3178312	1415286	6	6	13	6	1093	7.66	16.17	6	22.319	0	SEC24A	High	High
-452016239658	P51148	20584703	16877624	20584703	9164878	39	7	22	4	216	8.41	32.84	7	32.171	0	RAB5C	High	High

6860000																		
4447457189107330000	O60493	6085418	4989185	6085418	2709224	34	6	12	5	162	8.66	9.26	6	15.086	0	SNX3	High	High
-7169835918379750000	P39687	12151909	9959230	12151909	5408056	27	7	14	6	249	4.09	11.1	7	22.261	0	ANP32A	High	High
-9157567409128220000	Q86SQ0	387050.7	317197	387050.7	172244.1	2	2	2	2	1253	7.43	0	2	3.679	0.001	PHLDB2	High	Peak Found
-5442629486260090000	Q05397	2820127	2310705	2820127	1254758	7	6	10	6	1052	6.62	7.55	6	17.617	0	PTK2	High	High
4390061241352570000	Q9HC07	1748835	1431671	1748835	777424.9	17	3	4	3	324	7.02	7.45	3	14.516	0	TMEM165	High	High
426395396663470000	Q96J84	409986.2	335620.6	409986.2	182248.5	1	1	1	1	757	5.73	2.01	1	2.622	0.006	KIRREL; KIRREL1	High	Peak Found
7294352330531960000	Q99439	17158953	14045694	17158953	7627085	30	9	22	8	309	7.33	20.28	9	25.597	0	CNN2	High	High

7444936349459710000	Q32P28	46611758	38146482	46611758	20714281	30	18	55	17	736	5.14	63.53	18	90.919	0	LEPRE1; P3H1	High	High
4773020118127390000	P30740	9012798	7375051	9012798	4004796	24	8	14	8	379	6.28	16.66	8	31.456	0	SERPINB1	High	High
2381350149897150000	P51572	44568925	36449668	44568925	19792878	44	12	25	12	246	8.44	45.05	12	52.978	0	BCAP31	High	High
7356302297912560000	O75400	2460707	2012290	2460707	1092712	3	4	7	4	957	7.56	1.66	4	8.362	0	PRPF40A	High	High
3650651295291500000	O75369	32605530	26662495	32605530	14478253	27	55	123	43	2602	5.73	154.34	55	221.047	0	FLNB	High	High
1756983135160370000	Q8NBJ5	27484219	22467594	27484219	12200341	21	14	47	14	622	7.31	53.59	14	62.402	0	COLGALT1; GLT25D1	High	High
-10968856719763800	P62081	36849033	30121732	36849033	16356686	25	7	28	7	194	10.1	38.84	7	38.413	0	RPS7	High	High
-326633204403	O43324	5593376	4571585	5593376	2482460	36	7	16	7	174	8.54	25.32	7	28.565	0	EEF1E1	High	High

1460000																		
-91982353768306600	P00492	11890833	9711160	11890833	5273349	27	5	11	5	218	6.68	20.56	5	19.95	0	HPRT1	High	High
5527327330749920000	P61019	49952540	40783024	49952540	22145975	46	9	40	9	212	6.54	68.75	9	52.308	0	RAB2A	High	High
44025785413676800	A1L0T0	820528.1	669888.3	820528.1	363762.4	3	2	4	2	632	8.15	1.73	2	5.825	0	ILVBL	High	High
3418505594273780000	P48735	30817491	25150710	30817491	13657324	33	15	33	14	452	8.69	27.02	15	53.652	0	IDH2	High	High
313388091027813000	Q9UHG3	11355565	9267379	11355565	5032367	22	11	23	11	505	6.18	23.45	11	43.221	0	PCYOX1	High	High
-4952006714438070000	Q9Y4L1	81121089	66202110	81121089	35949033	26	24	81	24	999	5.22	92.91	24	121.084	0	HYOU1	High	High
6670278986062400000	P17050	2698795	2202047	2698795	1195754	8	3	5	3	411	5.19	8.3	3	9.433	0	NAGA	High	High
914599161153	Q16527	557588.2	454940.1	557588.2	247041.3	12	2	2	2	193	8.62	1.61	2	4.316	0	CSRP2	High	Peak Found

7020000																		
-7426540496609800000	O15118	1085027	885026.6	1085027	480586.7	2	3	6	3	1278	5.36	9.2	3	8.185	0	NPC1	High	High
5135985212415510000	P43686	23182927	18904401	23182927	10265457	33	15	40	15	418	5.21	50.84	15	55.613	0	PSMC4	High	High
-221730190353322000	Q9UL63	199586.2	162736.7	199586.2	88369.19	1	1	1	1	735	6.34	0	1	2.405	0.008	MKLN1	Peak Found	High
7978508680545550000	Q1KMD3	18973342	15460626	18973342	8395420	24	16	35	16	747	4.91	42.39	16	59.282	0	HNRNPUL2	High	High
-3853404113387130000	Q9UK59	877650.9	715121.8	877650.9	388325	3	2	2	2	544	5.47	2.26	2	5.006	0	DBR1	High	High
-880435081762619000	Q09666	3.07E+08	2.5E+08	3.07E+08	1.36E+08	51	155	393	155	5890	6.15	510.96	155	701.501	0	AHNAK	High	High
-681400762000750000	O60749	6027794	4910806	6027794	2666663	17	8	26	7	519	5.12	34.17	8	39.786	0	SNX2	High	High

-2890284807861640000	P22102	40432634	32936303	40432634	17885053	25	22	50	22	1010	6.7	74.49	22	78.129	0	GART	High	High
4192951457052180000	P06756	19757158	16092432	19757158	8738503	20	19	34	19	1048	5.68	30.27	19	61.127	0	ITGAV	High	High
-2007388225348890000	P00568	5687807	4631873	5687807	2515197	28	5	14	4	194	8.63	17.73	5	21.562	0	AK1	High	High
3912553749902590000	P15880	15793982	12858288	15793982	6982300	33	9	23	9	293	10.24	26.23	9	30.245	0	RPS2	High	High
3366486650245990000	P10515	10041712	8174201	10041712	4438750	17	8	18	8	647	7.84	10.43	8	28.798	0	DLAT	High	High
1516801179277520000	Q9HB71	14463392	11772476	14463392	6392683	49	12	29	12	228	8.25	34.42	12	50.587	0	CACYBP	High	High
5381305641147490000	Q9HAV7	779898.1	634686.5	779898.1	344647.1	9	2	4	2	217	8.12	6.15	2	7.746	0	GRPEL1	High	High

3602445909866350000	P19338	2.93E+08	2.38E+08	2.93E+08	1.29E+08	33	29	99	29	710	4.7	170.63	29	147.965	0	NCL	High	High
322091060557130000	Q8N0U8	382427.4	311210.7	382427.4	168993.4	6	1	1	1	176	9.13	2.12	1	3.033	0.003	VKORC1L1	High	Peak Found
-5434950316255040000	P42224	25294889	20581925	25294889	11176385	34	21	54	21	750	6.05	66.85	21	83.242	0	STAT1	High	High
-5988193438521900000	P26640	26493497	21555135	26493497	11704857	22	26	50	26	1264	7.59	67.92	26	109.559	0	VARS; VARS2	High	High
-911764670660096000	P08195	38565176	31371175	38565176	17035158	28	15	39	15	630	5.01	60.03	15	86.849	0	SLC3A2	High	High
-5340528139668480000	O95831	13442524	10934494	13442524	5937643	25	13	31	13	613	8.95	35.06	13	51.501	0	AIFM1	High	High
6019619507196980000	P61158	46345961	37697664	46345961	20470564	38	14	50	14	418	5.88	78.92	14	65.602	0	ACTR3	High	High

-5976996630127450000	O75815	289969.6	235761.6	289969.6	128023.1	2	2	3	2	825	7.96	0	2	4.094	0	BCAR3	High	High
-1213395878895100000	P26038	3.81E+08	3.1E+08	3.81E+08	1.68E+08	58	50	173	37	577	6.4	279.69	50	250.834	0	MSN	High	High
-6993572615210090000	Q9UJV9	2938930	2389495	2938930	1297542	16	8	12	8	622	6.84	7.27	8	24.499	0	DDX41	High	High
-5230612763215460000	Q99829	15623988	12702403	15623988	6897652	16	11	20	11	537	5.83	30.67	11	41.466	0	CPNE1	High	High
-5357648313694030000	Q8IUD2	1681313	1366605	1681313	742093	5	4	5	4	1116	5.97	4.2	4	14.314	0	ERC1	High	Peak Found
8479889696518380000	P37802	1.01E+08	82429978	1.01E+08	44761081	56	11	42	11	199	8.25	94.75	11	48.717	0	TAGLN2	High	High
-242309	Q8NI27	1864860	1515126	1864860	822742.9	2	4	7	4	15	8.44	7.2	4	11.031	0	THOC2	High	High

002880936000										93								
2851468461982240000	P30626	5622561	4567784	5622561	2480395	27	4	12	4	198	5.59	11.9	4	15.473	0	SRI	High	High
-1936662048644360000	P55060	54409310	44200625	54409310	24001799	21	19	49	19	971	5.77	54.29	19	69.757	0	CSE1L	High	High
213953215988823000	Q92896	9210633	7479669	9210633	4061606	12	14	27	14	1179	6.9	21.73	14	46.034	0	GLG1	High	High
6456635449604070000	P30419	1181555	959332.1	1181555	520936	6	2	3	2	496	7.8	7.96	2	13.14	0	NMT1	High	Peak Found
-6444296704773160000	Q9NSD9	26343799	21388228	26343799	11614224	23	14	38	14	589	6.84	42.19	14	45.289	0	FARSB	High	High
-4061147392346930000	P61006	96306622	78144024	96306622	42433724	45	10	26	4	207	9.07	29.7	10	43.191	0	RAB8A	High	High
-720458376712	Q6PL18	263879.2	214070.6	263879.2	116244.5	2	2	2	2	13	6.32	0	2	5.833	0	ATAD2	High	High

1540000										90								
397277040626667000	Q9UBB4	11844003	9607928	11844003	5217291	27	12	19	12	475	5.25	20.72	12	45.162	0	ATXN10	High	High
-378984088295691000	P49023	1514586	1228334	1514586	667009.1	4	2	4	2	591	6.19	6.22	2	5.828	0	PXN	High	High
-8512684419442890000	P24844	6393106	5184686	6393106	2815385	51	7	28	2	172	4.92	26.6	7	25.48	0	MYL9	High	High
-2447610796588380000	Q96SZ5	283023	229497.4	283023	124621.5	3	1	2	1	270	6.04	2.09	1	3.446	0.002	ADO	High	Peak Found
7307754887580190000	Q13287	785359.8	636751.4	785359.8	345768.4	17	4	4	4	307	5.34	5.9	4	17.512	0	NMI	High	High
-1279180482371060000	Q3ZAQ7	3393404	2751006	3393404	1493850	35	3	4	3	101	7.24	1.68	3	7.345	0	VMA21	High	High
7553358112482030000	O00767	182040.9	147534.3	182040.9	80113.97	3	1	1	1	359	9	0	1	2.408	0.008	SCD	High	Peak Found

8779201903022420000	Q96JJ7	7779278	6301929	7779278	3422070	13	6	14	6	454	4.91	13.41	6	20.113	0	TMX3	High	High
4549741875275550000	Q99627	2559196	2073071	2559196	1125718	30	4	7	4	209	5.38	8.21	4	17.695	0	COPS8	High	High
-165801227635855000	Q00839	1.08E+08	87531400	1.08E+08	47531252	26	20	83	19	825	6	125.94	20	104.141	0	HNRNPU	High	High
7273803465605430000	P05455	24430005	19781050	24430005	10741495	32	18	36	18	408	7.12	57.81	18	87.304	0	SSB	High	High
4752916691292970000	Q15057	1446999	1171211	1446999	635990.3	3	3	3	3	778	6.8	0	3	6.029	0	ACAP2	High	High
-5272993671693510000	Q15181	15212404	12311692	15212404	6685488	35	9	21	8	289	5.86	31.67	9	48.355	0	PPA1	High	High
2958719848471140000	P54687	282022.3	228221.5	282022.3	123928.7	3	1	2	1	386	5.3	0	1	3.439	0.002	BCAT1	High	Peak Found
945913478307562000	Q9Y2Q9	1468404	1188277	1468404	645257.3	16	3	4	3	187	9.1	3.42	3	9.836	0	MRPS28	High	High

-9012123817603870000	P46977	26301515	21279356	26301515	11555104	11	8	19	8	705	8.07	30.16	8	29.804	0	STT3A	High	High
4854644692515830000	Q86X76	1044882	845355.4	1044882	459044.4	10	3	5	3	327	7.74	1.81	3	9.323	0	NIT1	High	High
-5100210059596340000	P04899	35517809	28725162	35517809	15598321	37	12	40	7	355	5.54	50.94	12	53.875	0	GNAI2	High	High
4892989875718200000	Q9UL46	12843083	10382420	12843083	5637856	34	8	14	8	239	5.73	19.29	8	30.667	0	PSME2	High	High
6687087814607390000	O15270	223778.7	180843.7	223778.7	98201.67	1	1	1	1	562	7.78	0	1	2.382	0.008	SPTLC2	High	Peak Found
2002757993896460000	Q9NQW6	6874419	5553644	6874419	3015737	6	7	17	7	1124	8.07	16.28	7	18.686	0	ANLN	High	High
3084200639085860000	O14497	1020191	824000.7	1020191	447448.4	2	3	6	3	2285	6.7	0	3	9.328	0	ARID1A	High	Peak Found

-3187719389386550000	Q9Y2V2	571221.9	461366.2	571221.9	250530.8	11	1	4	1	147	8.21	0	1	3.76	0.001	CARHSP1	High	Peak Found
-5062792901890910000	P52895	9112418	7353379	9112418	3993028	28	9	32	5	323	7.49	47.52	9	48.942	0	AKR1C2	High	High
-2491360545906130000	P54920	14930020	12046757	14930020	6541623	51	11	28	11	295	5.36	52.33	11	73.018	0	NAPA	High	High
3673508278002200000	P36542	22089714	17813259	22089714	9672946	23	8	27	8	298	9.22	28.92	8	27.654	0	ATP5C1	High	High
-1721823371863970000	Q92973	13762172	11096600	13762172	6025669	15	13	26	13	898	4.98	27.87	13	42.918	0	TNPO1	High	High
682895518506643000	P11021	6.03E+08	4.86E+08	6.03E+08	2.64E+08	49	40	221	38	654	5.16	443.43	40	290.611	0	HSPA5	High	High
-2647028449518710000	P16383	235846.3	189956.2	235846.3	103149.9	1	1	1	1	781	5.99	0	1	2.484	0.007	GCFC2	High	Peak Found

-6195268452072660000	P40189	1348514	1085476	1348514	589434.8	4	3	4	3	918	5.95	2.43	3	8.346	0	IL6ST	High	Peak Found
5184627923008410000	Q07157	5485782	4413988	5485782	2396881	7	10	17	10	1748	6.7	16.02	10	29.654	0	TJP1	High	High
-410325427073227000	P68104	1.11E+09	8.9E+08	1.11E+09	4.83E+08	47	20	173	8	462	9.01	366.68	20	179.977	0	EEF1A1	High	High
-7845595134204640000	Q8NE71	6331484	5093335	6331484	2765780	17	11	17	11	845	6.8	15.67	11	48.341	0	ABCF1	High	High
-4830366311257210000	P48147	5299607	4262806	5299607	2314787	12	7	8	7	710	5.86	5.46	7	26.084	0	PREP	High	High
-8022290740510180000	P49257	37378582	30064670	37378582	16325700	30	13	36	13	510	6.77	47.71	13	67.508	0	LMAN1	High	High
5761790178817740000	Q96P70	8632124	6942498	8632124	3769911	12	11	19	11	1041	4.81	7.9	11	32.604	0	IPO9	High	High

-1011353078992680000	P49736	10331067	8307772	10331067	4511282	19	15	31	15	904	5.52	44.15	15	61.573	0	MCM2	High	High
4299442073717890000	Q9Y6A4	2374560	1909503	2374560	1036897	16	3	6	3	193	9.76	5.23	3	8.789	0	C16orf80; CFAP20	High	High
-7097595206598810000	P09012	17695702	14222823	17695702	7723269	27	7	18	5	282	9.83	24.83	7	21.748	0	SNRPA	High	High
304179204082467000	Q12841	2351004	1888548	2351004	1025518	13	4	5	4	308	5.52	1.63	4	10.828	0	FSTL1	High	High
-2770447616449310000	Q9Y678	55942300	44919304	55942300	24392055	32	25	65	23	874	5.47	86.84	25	105.829	0	COPG1	High	High
9193943049168300000	Q02818	6872626	5518239	6872626	2996511	20	8	12	8	461	5.25	12	8	28.82	0	NUCB1	High	High
916743004623214000	P62266	14299343	11479709	14299343	6233705	16	3	10	3	143	10.49	26.31	3	15.967	0	RPS23	High	High
-561310133181	O43294	121353.7	97374.41	121353.7	52876.2	2	1	2	1	461	7.03	0	1	2.071	0.01	TGFB1I1	High	Peak Found

9550000																		
-3138004166962980000	Q92688	9882714	7928434	9882714	4305294	16	6	8	5	251	4.06	10.63	6	24.698	0	ANP32B	High	High
3427861429656690000	O95197	28908873	23189898	28908873	12592565	3	4	14	3	1032	4.96	23.11	4	20.218	0	RTN3	High	High
-1597744866915900000	P51114	14675596	11762262	14675596	6387137	18	9	30	7	621	6.15	45.17	9	44.518	0	FXR1	High	High
2020319621841400000	P07741	16863573	13515888	16863573	7339390	33	5	30	5	180	6.02	49.89	5	32.096	0	APRT	High	High
-5964921006077680000	Q92522	683878.1	548090.7	683878.1	297623.9	5	1	2	1	213	10.76	2.04	1	3.879	0	H1FX	High	High
7481349692904650000	Q9UPY5	972275.2	779170	972275.2	423104.5	1	1	2	1	501	9.19	0	1	2.457	0.007	SLC7A11	High	High
737995412531	P48643	55255388	44280340	55255388	24045085	35	21	64	20	541	5.66	81.58	21	98.138	0	CCT5	High	High

9750000																		
-393102318263345000	P51688	1763582	1413265	1763582	767430.5	5	2	6	2	502	6.95	5.31	2	9.47	0	SGSH	High	High
2905303877157190000	Q10469	372034.9	298089.9	372034.9	161868.6	2	1	1	1	447	8.76	0	1	2.21	0.009	MGAT2	High	Peak Found
-1553540605597610000	P51965	3176410	2544838	3176410	1381897	8	2	3	2	193	8.53	3.63	2	4.017	0	UBE2E1	High	High
-6716133539155150000	P82650	4248587	3403372	4248587	1848097	19	6	10	6	360	7.9	10.85	6	21.505	0	MRPS22	High	High
-4650231133703770000	Q96RF0	724009.8	579813.2	724009.8	314849.8	4	3	3	3	628	5.68	0	3	6.581	0	SNX18	High	Peak Found
-2363615815347430000	Q01995	1888828	1512508	1888828	821321.1	16	3	3	3	201	8.84	4.37	3	9.391	0	TAGLN	High	Peak Found

-1119286615561910000	P00558	5.69E+08	4.55E+08	5.69E+08	2.47E+08	83	41	206	35	417	8.1	318.74	41	275.116	0	PGK1	High	High
4636491781207180000	P14868	55146384	44152596	55146384	23975718	52	23	63	23	501	6.55	87.14	23	91.248	0	DARS	High	High
7931435872977730000	P05556	59810414	47864385	59810414	25991292	24	20	71	20	798	5.39	108.06	20	87.738	0	ITGB1	High	High
5950667386624240000	Q01082	1.12E+08	89280144	1.12E+08	48480854	40	81	192	81	2364	5.57	268.62	81	357.214	0	SPTBN1	High	High
3062212909163770000	P19838	234554.6	187583.5	234554.6	101861.5	1	1	1	1	968	5.4	0	1	2.152	0.009	NFKB1	High	Peak Found
-8706245126759520000	Q9BRP4	680160.1	543823	680160.1	295306.4	6	2	3	2	392	6.32	0	2	5.171	0	PAAF1	High	High
1395564513229480000	O94826	8904373	7119369	8904373	3865956	18	8	15	8	608	7.12	10.6	8	19	0	TOMM70A; TOMM70	High	High

948534183126948000	Q8IWE5	182661.3	146032.4	182661.3	79298.44	1	1	1	1	1019	4.93	0	1	3.063	0.003	PLEKHM2	High	Peak Found
4900440247780220000	Q13616	3559754	2844782	3559754	1544772	12	10	12	10	776	8	5.73	10	25.33	0	CUL1	High	High
-7515792609303090000	P61204	14432344	11533560	14432344	6262947	31	7	31	4	181	7.43	53.37	7	36.786	0	ARF3	High	High
-9171365705039640000	P45974	17040379	13616481	17040379	7394014	25	17	34	17	858	5.03	37.78	17	70.166	0	USP5	High	High
-5991107467839090000	Q99747	6198614	4953082	6198614	2689620	26	8	16	8	312	5.41	13.98	8	25.399	0	NAPG	High	High
8637497860104460000	P67812	3440311	2748704	3440311	1492600	12	2	5	2	179	9.48	8.99	2	6.977	0	SEC11A	High	High
6669951735420190000	O00154	32846991	26239003	32846991	14248289	33	10	38	9	380	8.54	53.33	10	52.628	0	ACOT7	High	High

-1901322686263970000	Q32P44	292138.1	233339.6	292138.1	126707.9	2	1	2	1	896	7.12	1.87	1	5.562	0	EML3	High	High
-2398790545086000000	P45880	13882040	11087113	13882040	6020518	20	5	11	5	294	7.56	15.8	5	18.378	0	VDAC2	High	High
3490068427957950000	P49588	29943050	23907454	29943050	12982212	29	26	45	26	968	5.53	50.42	26	92.468	0	AARS	High	High
-7903572994601530000	Q9Y6N5	1457224	1163471	1457224	631787.6	11	5	7	5	450	9.11	6	5	15.156	0	SQRDL; SQOR	High	High
-8487408262660340000	P11388	9720980	7760875	9720980	4214306	9	14	31	11	1531	8.72	22.74	14	46.009	0	TOP2A	High	High
6062683060595130000	Q9NVE7	620701.1	495492.8	620701.1	269062.2	3	2	3	2	773	6.28	4.72	2	7.739	0	PANK4	High	High
-504997331308259000	P17174	9251044	7383378	9251044	4009318	20	8	19	8	413	7.01	28.05	8	27.771	0	GOT1	High	High

6737848688669360000	Q9UNZ2	2058911	1643029	2058911	892196.8	26	7	10	7	370	5.1	17.55	7	26.276	0	NSFL1C	High	High
8473772251373630000	Q12907	13884538	11079931	13884538	6016618	22	9	31	9	356	6.95	47.47	9	45.815	0	LMAN2	High	High
-1823478182965960000	O43150	115496.2	92155.48	115496.2	50042.22	2	2	2	1	1006	6.68	0	2	3.332	0.002	ASAP2	High	Peak Found
2268308097842940000	Q9NPL8	1221213	974353.8	1221213	529093.1	12	3	3	3	285	8.5	3.92	3	12.016	0	TIMMDC1	High	Peak Found
6432358186010660000	Q15006	5813679	4638481	5813679	2518785	14	4	7	4	297	6.57	11.84	4	18.728	0	EMC2	High	High
460406497697363000	P61018	289832.8	231199.2	289832.8	125545.6	11	2	2	1	213	6.06	0	2	4.659	0	RAB4B	Peak Found	High
3142300216691490000	Q9UJS0	1799344	1434810	1799344	779129.8	10	6	9	4	675	8.62	9.28	6	17.144	0	SLC25A13	High	High
7053379414769100000	Q92905	6725720	5362347	6725720	2911859	26	7	9	7	334	6.54	16.17	7	26.799	0	COPS5	High	High

-2220837361715170000	P08238	1.09E+09	8.71E+08	1.09E+09	4.73E+08	55	51	268	28	724	5.03	465.88	51	330.819	0	HSP90AB1	High	High
8610404900026580000	P27797	2.52E+08	2.01E+08	2.52E+08	1.09E+08	46	21	103	21	417	4.44	147.31	21	138.194	0	CALR	High	High
-8012908085941330000	Q99832	1.05E+08	83800667	1.05E+08	45505391	52	27	81	27	543	7.65	111.22	27	122.388	0	CCT7	High	High
-7705238959085990000	P08865	88908399	70826814	88908399	38460337	44	11	41	11	295	4.87	85.02	11	61.233	0	RPSA	High	High
-7821443227356310000	Q96EL2	899280.4	716179.2	899280.4	388899.3	5	1	1	1	167	9.38	0	1	2.74	0.005	MRPS24	High	Peak Found
2936777588717640000	O95070	2381192	1896195	2381192	1029671	5	1	5	1	293	8.95	11.19	1	6.455	0	YIF1A	High	High
-194185232775	Q93009	5864089	4669386	5864089	2535567	8	10	17	10	1102	5.55	10.13	10	33.195	0	USP7	High	High

2540000																		
4101448346661800000	Q15819	18825290	14984868	18825290	8137074	35	5	23	5	145	8.09	21.71	5	20.288	0	UBE2V2	High	High
-2651873567954200000	Q9P2J5	33579488	26706672	33579488	14502242	26	28	61	28	1176	7.3	68.84	28	91.14	0	LARS	High	High
-719373628240286000	O00231	31214590	24820625	31214590	13478082	42	17	50	17	422	6.48	73.48	17	77.842	0	PSMD11	High	High
-1191576538122750000	Q9Y646	3430143	2727504	3430143	1481088	8	4	7	4	472	6.18	9.6	4	13.863	0	CPQ	High	High
5788074156748140000	Q96IJ6	6208502	4935268	6208502	2679947	16	6	14	6	420	7.21	19.24	6	27.932	0	GMPPA	High	High
-8133937274084100000	P28074	14949116	11881401	14949116	6451832	26	7	21	7	263	6.92	29.52	7	32.176	0	PSMB5	High	High
300888284752	Q15041	5404698	4295446	5404698	2332511	14	3	11	3	203	9.32	15.31	3	10.741	0	ARL6IP1	High	High

4470000																		
8469309847663240000	Q9UEY8	7382623	5864813	7382623	3184707	12	8	14	8	706	6.32	15.63	8	28.648	0	ADD3	High	High
1010665989767420000	Q96CS3	5046020	4008122	5046020	2176488	15	5	11	5	445	5.62	8.28	5	29.488	0	FAF2	High	High
-6096160842803460000	Q9NQX3	309970.1	246006.3	309970.1	133586.2	1	1	1	1	736	5.43	0	1	2.639	0.005	GPHN	High	Peak Found
-1694766210594210000	P10301	1232519	977942	1232519	531041.5	29	4	12	2	218	6.93	13.67	4	16.268	0	RRAS	High	Peak Found
1619484696952210000	P24534	33141589	26295540	33141589	14278989	36	8	22	6	225	4.67	28.61	8	36.883	0	EEF1B2	High	High
-1627682917177360000	Q92616	19604099	15553496	19604099	8445851	12	30	53	30	2671	7.47	35.45	30	91.191	0	GCN1L1; GCN1	High	High
-201536	P48163	11055378	8769794	11055378	4762169	13	8	16	8	572	6.13	16.71	8	23.178	0	ME1	High	High

554680763000																		
7964397888877460000	P50897	3434589	2723612	3434589	1478974	14	3	8	3	306	6.52	6.16	3	12.07	0	PPT1	High	High
-6062730820723700000	P82094	394838.4	313097.3	394838.4	170017.9	3	2	2	2	1093	4.92	2.61	2	5.875	0	TMF1	High	Peak Found
1112180940687260000	Q15185	10315014	8178228	10315014	4440937	34	6	12	6	160	4.54	12.15	6	21.185	0	PTGES3	High	High
-4684280243301620000	Q9H3H5	1041802	825962.1	1041802	448513.5	4	2	3	2	408	8	0	2	3.709	0.001	DPAGT1	High	High
-2894972574115960000	P11234	574996.8	455827.6	574996.8	247523.2	23	4	12	2	206	6.62	6.07	4	9.795	0	RALB	High	High
8717421015690560000	Q9NQR4	5821122	4614550	5821122	2505790	16	5	12	5	276	7.21	17.98	5	18.972	0	NIT2	High	High
-829307987825	P14625	6.46E+08	5.12E+08	6.46E+08	2.78E+08	46	45	209	43	803	4.84	326.66	45	260.677	0	HSP90B1	High	High

1600000																		
1252857797912050000	Q96GQ5	249796.8	197932.5	249796.8	107481.2	5	2	2	2	468	6.93	0	2	5.515	0	C16orf58	High	Peak Found
1721155162359160000	Q9UBQ0	2352294	1863660	2352294	1012004	13	2	4	2	182	6.79	5.8	2	16.656	0	VPS29	High	High
-1679721907195940000	P50502	32664808	25877510	32664808	14051991	18	6	18	6	369	5.27	26.48	6	32.761	0	ST13	High	High
-8333902830713900000	P05388	63706213	50465437	63706213	27403714	32	11	35	11	317	5.97	70.54	11	60.923	0	RPLP0	High	High
-2613638019378130000	P04844	39192604	31041724	39192604	16856260	20	10	43	10	631	5.69	71.06	10	73.27	0	RPN2	High	High
9080862626962090000	O75312	2392266	1893943	2392266	1028448	10	4	7	4	459	4.73	11.71	4	16.568	0	ZNF259; ZPR1	High	High
736819519647	Q9BUB7	1555827	1231473	1555827	668713.9	9	2	5	2	260	8.91	6.54	2	9.758	0	TMEM70	High	High

6720000																		
-6252367434931230000	P49327	1.62E+08	1.28E+08	1.62E+08	69696187	28	62	184	62	2511	6.44	240.17	62	256.875	0	FASN	High	High
1839612719642200000	P09417	1070456	847242.2	1070456	460069	5	1	2	1	244	7.37	3.86	1	3.151	0.003	QDPR	High	High
-772083225745130000	P11142	8.19E+08	6.48E+08	8.19E+08	3.52E+08	62	41	228	29	646	5.52	421.52	41	277.083	0	HSPA8	High	High
8494127544242020000	P62249	1.05E+08	82698071	1.05E+08	44906660	57	11	57	11	146	10.21	97.15	11	40.975	0	RPS16	High	High
7448021442665090000	O95347	10175764	8049306	10175764	4370930	17	20	28	20	1197	8.43	24.46	20	60.388	0	SMC2	High	High
-4288023909312920000	P06744	1.17E+08	92814404	1.17E+08	50400026	36	19	78	19	558	8.32	104.16	19	93.393	0	GPI	High	High
-277938126228	P17655	36057144	28518294	36057144	15485988	19	15	42	15	700	4.98	63.42	15	55.285	0	CAPN2	High	High

9930000																		
2962281166201310000	P35579	1.13E+09	8.92E+08	1.13E+09	4.84E+08	62	148	567	125	1960	5.6	1131.03	148	893.983	0	MYH9	High	High
7742249596596200000	Q7KZF4	80102030	63285789	80102030	34365414	40	33	101	33	910	7.17	146.96	33	143.576	0	SND1	High	High
-6205796797264400000	Q96DH6	185323.7	146408.9	185323.7	79502.88	5	1	1	1	328	8.48	0	1	3.159	0.003	MSI2	High	Peak Found
-9169618704545610000	P04075	5.24E+08	4.14E+08	5.24E+08	2.25E+08	65	27	156	23	364	8.09	321.07	27	199.618	0	ALDOA	High	High
5778403088850180000	Q16850	2951369	2331230	2951369	1265903	14	6	12	6	503	8.53	6.74	6	24.742	0	CYP51A1	High	High
-3580192836813550000	O95425	2088409	1649570	2088409	895748.8	4	7	9	7	2214	6.98	7.51	7	19.157	0	SVIL	High	High
-485890932253	Q16881	36224155	28612058	36224155	15536904	22	16	50	16	649	7.39	77.62	16	75.021	0	TXNRD1	High	High

2710000																		
868843744032576000	P48444	23812601	18805148	23812601	10211561	33	16	44	16	511	6.21	52.92	16	60.936	0	ARCN1	High	High
2726683976815450000	P00338	9.57E+08	7.55E+08	9.57E+08	4.1E+08	44	20	133	18	332	8.27	251.37	20	148.055	0	LDHA	High	High
4113768837461090000	A0AVT1	8142707	6425641	8142707	3489248	13	12	20	12	1052	6.14	21.59	12	34.889	0	UBA6	High	High
-572232975925861000	P46779	5145527	4060202	5145527	2204769	24	4	10	4	137	12.02	10.4	4	9.593	0	RPL28	High	High
-4910055014827060000	Q14653	1926610	1519996	1926610	825387.3	7	2	2	2	427	5.34	3.44	2	10.38	0	IRF3	High	High
4368793239132190000	P23634	15731553	12408852	15731553	6738248	19	19	27	10	1241	6.6	26.06	19	69.178	0	ATP2B4	High	High
479686062085054000	Q15365	60073663	47376130	60073663	25726160	51	12	37	8	356	7.09	68.08	12	62.659	0	PCBP1	High	High
599076344580	O75083	1.1E+08	86540889	1.1E+08	46993385	47	23	64	23	606	6.65	94.83	23	133.751	0	WDR1	High	High

8980000																		
3304952133381540000	Q99614	2899878	2286359	2899878	1241537	12	3	6	3	292	4.84	5.59	3	10.947	0	TTC1	High	High
2127950675318560000	Q9UJA5	813704.4	641441.3	813704.4	348315.1	4	2	2	2	497	7.55	2.14	2	5.44	0	TRMT6	High	Peak Found
7337665348008380000	O60701	1.39E+08	1.09E+08	1.39E+08	59409808	53	24	76	24	494	7.12	129.57	24	124.436	0	UGDH	High	High
7273003467290870000	Q9UNE7	1733596	1366167	1733596	741855	18	5	9	5	303	5.87	5.94	5	14.506	0	STUB1	High	High
4994086141420820000	P02794	55523288	43742776	55523288	23753178	56	10	31	10	183	5.55	36.58	10	61.178	0	FTH1	High	High
3542088452759520000	Q9BQA9	144880.1	114139.1	144880.1	61979.73	4	1	1	1	187	6.8	0	1	2.67	0.005	C17orf62	High	Peak Found
523405854747225000	Q16222	32491349	25597154	32491349	13899752	32	16	41	16	522	6.33	41.18	16	47	0	UAP1	High	High
576422257998	Q6UWP7	3234791	2548302	3234791	1383778	15	5	9	5	414	8.62	13.35	5	16.16	0	LCLAT1	High	High

6530000																			
-3862869163996360000	Q7LBR1	1369750	1078581	1369750	585690.4	9	2	3	2	199	8.1	4.38	2	5.474	0	CHMP1B	High	Peak Found	
6400995593712080000	Q9NYU2	21111429	16620806	21111429	9025421	13	22	47	19	1555	5.63	36.8	22	66.645	0	UGGT1	High	High	
1937254779408720000	Q9Y4F1	142772.1	112353.7	142772.1	61010.23	1	1	2	1	1045	8.15	1.6	1	2.361	0.008	FARP1	High	High	
-8424290997106930000	O43854	2762583	2173986	2762583	1180516	4	2	8	2	480	7.28	11.41	2	5.012	0	EDIL3	High	High	
-11876406759909500	O75352	730434.8	574757.2	730434.8	312104.3	4	1	4	1	247	8.94	0	1	4.04	0	MPDU1	High	High	
4146618986531710000	P46782	41640589	32764082	41640589	17791533	29	7	20	7	204	9.72	36.67	7	30.257	0	RPS5	High	High	
5775237487534850000	P62750	61229745	48175170	61229745	26160054	42	11	46	11	156	10.45	86.41	11	61.113	0	RPL23A	High	High	

-6592618097650740000	Q9UDY2	8447314	6646193	8447314	3609012	16	17	32	17	1190	7.4	35.35	17	58.52	0	TJP2	High	High
-2009427819375180000	P62333	27549406	21673197	27549406	11768967	52	18	46	18	389	7.49	45.99	18	75.917	0	PSMC6	High	High
-8595092831186630000	Q99961	10294366	8098040	10294366	4397393	27	8	14	8	368	5.43	17.84	8	32.729	0	SH3GL1	High	High
3453697574232540000	O43707	3.91E+08	3.08E+08	3.91E+08	1.67E+08	60	56	222	40	911	5.44	369.9	56	353.906	0	ACTN4	High	High
-1205545228874980000	Q92900	18717746	14715717	18717746	7990920	28	27	58	27	1129	6.61	52.32	27	98.326	0	UPF1	High	High
162110754595933000	P53007	10749275	8445477	10749275	4586058	19	6	19	6	311	9.89	25.08	6	24.005	0	SLC25A1	High	High
5468069501493150000	P37837	1.09E+08	85799664	1.09E+08	46590886	39	13	54	13	337	6.81	93.8	13	64.904	0	TALDO1	High	High

5622553416518630000	P07093	1303994	1024104	1303994	556108.6	12	4	9	4	398	9.29	4.27	4	14.347	0	SERPINE2	High	High
1011976368699820000	P41091	39498422	31012702	39498422	16840500	36	14	32	14	472	8.4	44.42	14	68.162	0	EIF2S3	High	High
8460064629514410000	Q8TED1	7284919	5716858	7284919	3104365	26	6	12	6	209	9.35	10.73	6	15.849	0	GPX8	High	High
-5363989837697160000	Q9Y4C2	1341021	1052326	1341021	571433.5	4	4	8	4	921	6.54	3.97	4	10.483	0	FAM115A; TCAF1	High	High
-5521793610303510000	P52789	9021165	7076114	9021165	3842468	20	17	38	14	917	6.05	58.95	17	75.338	0	HK2	High	High
8472410284542290000	P61289	5972509	4682316	5972509	2542589	26	6	15	6	254	5.95	19.79	6	25.45	0	PSME3	High	High
4594033010686820000	P48637	12481339	9784805	12481339	5313339	27	10	28	10	474	5.92	22.32	10	29.769	0	GSS	High	High

1855541352356260000	Q2PZI1	1531782	1200686	1531782	651996	5	3	3	3	675	8.95	3.84	3	8.167	0	DPY19L1	High	Peak Found
8551730055028160000	P08579	5250134	4114214	5250134	2234098	25	6	16	4	225	9.72	22.69	6	17.102	0	SNRPB2	High	High
7555095530053000000	P50416	32135873	25179802	32135873	13673122	23	19	56	19	773	8.65	61.04	19	73.044	0	CPT1A	High	High
3271098639454740000	Q9H9B4	18415301	14428973	18415301	7835213	35	11	34	10	322	9.07	49.04	11	40.731	0	SFXN1	High	High
-6017876226430420000	P46821	38601485	30229887	38601485	16415417	18	32	70	30	2468	4.81	83.54	32	146.529	0	MAP1B	High	High
2017591020137150000	O14966	215489.5	168725.5	215489.5	91621.25	11	2	3	1	203	7.18	6.26	2	6.67	0	RAB7L1; RAB29	High	Peak Found
-6341568024441870000	O75131	47997339	37579346	47997339	20406315	31	17	48	17	537	5.85	57.38	17	72.854	0	CPNE3	High	High

3877327812570750000	P50570	18978482	14857487	18978482	8067904	25	20	40	10	870	7.44	40.67	20	71.088	0	DNM2	High	High
-7682312476163040000	P82914	1325323	1037168	1325323	563202.2	7	2	4	2	257	10.48	0	2	3.773	0.001	MRPS15	High	High
6210925912035560000	Q14980	27621472	21614643	27621472	11737171	18	35	68	35	2115	5.78	85.52	35	136.589	0	NUMA1	High	High
5370481134034690000	P22307	25193269	19712349	25193269	10704189	17	10	26	10	547	6.89	36.39	10	37.278	0	SCP2	High	High
-5155444033397580000	Q06265	2010039	1572480	2010039	853887.1	5	2	4	2	439	5.29	1.99	2	9.262	0	EXOSC9	High	High
-9211405473836280000	Q53EP0	7143938	5588090	7143938	3034441	17	15	22	15	1204	5.95	18.06	15	53.752	0	FNDC3B	High	High
-9069432479560650000	Q08AM6	2151913	1682841	2151913	913815.2	8	7	8	7	782	6.13	5.41	7	18.533	0	VAC14	High	High

-2050551167936510000	Q01813	1.08E+08	84480372	1.08E+08	45874484	30	23	93	20	784	7.55	160.54	23	135.922	0	PFKP	High	High
-7073332157955070000	P50914	35866616	28044243	35866616	15228569	28	6	27	6	215	10.93	43.72	6	33.088	0	RPL14	High	High
2956941067343710000	O75718	22759097	17786290	22759097	9658301	23	9	28	9	401	5.73	30.78	9	42.332	0	CRTAP	High	High
3985188646102160000	P53041	3146770	2458731	3146770	1335139	16	7	11	7	499	6.28	8.3	7	24.237	0	PPP5C	High	High
6048249212605460000	P30876	5045319	3941357	5045319	2140233	9	10	18	10	1174	6.87	9.47	10	25.599	0	POLR2B	High	High
4397536938893970000	O43491	3951911	3087069	3951911	1676338	7	7	9	7	1005	5.44	14.05	7	20.062	0	EPB41L2	High	High
4340156669496060000	Q9Y3C6	1468800	1147138	1468800	622918.1	23	3	6	3	166	7.99	2.13	3	9.743	0	PPIL1	High	High

3518541281494460000	P42330	32715821	25546744	32715821	13872379	33	10	26	6	323	7.94	35.84	10	45.173	0	AKR1C3	High	High
5192872055406060000	O95168	874412.7	682720.4	874412.7	370730.5	17	2	3	2	129	9.85	0	2	3.915	0	NDUFB4	High	High
1535429357355420000	Q9UL25	2763355	2157108	2763355	1171352	20	4	11	4	225	7.94	11.84	4	20.28	0	RAB21	High	High
-7303961078788050000	P49792	12798316	9990389	12798316	5424976	6	18	33	18	3224	6.2	36.6	18	67.041	0	RANBP2	High	High
-4615289327887400000	Q9Y490	1.62E+08	1.27E+08	1.62E+08	68812698	38	82	214	70	2541	6.07	339.26	82	428.801	0	TLN1	High	High
2767038677570430000	P82663	1021044	796659.8	1021044	432601.8	15	2	2	2	173	8.82	1.83	2	7.314	0	MRPS25	High	High
6000337729666770000	Q9Y5Z9	349600.3	272766.7	349600.3	148117.6	3	1	3	1	338	8.15	1.72	1	3.919	0	UBIAD1	High	High

-8210818149912640000	P11216	48431289	37779801	48431289	20515166	29	26	58	21	843	6.86	75.18	26	103.764	0	PYGB	High	High
-6229866583710040000	Q96C23	212339.2	165616.6	212339.2	89933.02	4	1	1	1	342	6.65	0	1	2.65	0.005	GALM	High	Peak Found
3736107449275980000	Q96M96	1967919	1534657	1967919	833348.8	11	6	7	6	766	6.13	9.21	6	30.798	0	FGD4	High	High
8009088926221670000	P40616	12003904	9360325	12003904	5082839	31	4	8	4	181	5.72	14.16	4	17.692	0	ARL1	High	High
-5439043522832820000	Q8WZA0	685843.9	534711.1	685843.9	290358.5	4	1	3	1	190	4.94	1.94	1	2.723	0.005	LZIC	High	High
-631503824976752000	Q9BYD2	1582145	1233287	1582145	669699	7	2	6	2	267	10.08	1.68	2	5.036	0	MRPL9	High	High
4445993330794880000	P54577	72107665	56206308	72107665	30521118	60	32	90	32	528	7.05	132.54	32	132.02	0	YARS	High	High

-6681537936157470000	Q9UH65	5006454	3901635	5006454	2118663	10	7	9	7	585	5.87	5.83	7	15.672	0	SWAP70	High	High
4360705577473030000	Q969X5	9820632	7652992	9820632	4155724	21	6	17	6	290	7.06	20.6	6	29.35	0	ERGIC1	High	High
-143757823348630000	Q8IV08	4191349	3265723	4191349	1773351	10	5	10	5	490	6.47	13.78	5	16.174	0	PLD3	High	High
-2561010377563700000	Q8WX92	351702.6	273945.2	351702.6	148757.6	6	3	5	3	580	6.13	2.03	3	8.138	0	COBRA1; NELFB	High	High
5846443296709180000	Q9Y248	1560856	1215437	1560856	660005.7	11	2	3	2	185	5.44	0	2	7.703	0	GINS2	High	Peak Found
-1007819713485120000	P83916	361059.6	281037.6	361059.6	152608.9	15	2	5	1	185	4.93	3.85	2	6.669	0	CBX1	High	High
289518910119416000	P61966	1120291	871954	1120291	473488	25	4	9	2	158	5.73	3.91	4	12.065	0	AP1S1	High	High

-3468928122779260000	P51398	2995504	2330510	2995504	1265512	16	6	10	6	398	8.88	11.74	6	19.212	0	DAP3	High	High
-6026735558710270000	P36507	13177248	10251539	13177248	5566785	24	10	19	7	400	6.55	14.91	10	36.466	0	MAP2K2	High	High
7630142415015690000	P14314	1.07E+08	83553129	1.07E+08	45370974	44	19	64	19	528	4.41	108.97	19	94.747	0	PRKCSH	High	High
6421674880825800000	Q96C19	20251881	15752394	20251881	8553856	42	11	30	11	240	5.2	41.04	11	42.956	0	EFHD2	High	High
-1337751321720820000	O76074	530091.8	412236.7	530091.8	223852.5	3	3	4	3	875	6.09	5.38	3	9.626	0	PDE5A	High	High
-8625862126903740000	P48556	11359869	8832513	11359869	4796226	14	6	14	6	350	9.7	15.36	6	15.259	0	PSMD8	High	High
3178523695898660000	Q9NYF8	4622847	3593072	4622847	1951108	9	8	14	8	920	9.98	5.72	8	22.954	0	BCLAF1	High	High

-6996937188134370000	Q16531	35081149	27253922	35081149	14799410	28	28	59	28	1140	5.26	69.76	28	111.506	0	DDB1	High	High
-2350188829608930000	Q8TDD1	2742099	2129939	2742099	1156598	9	6	11	6	881	10.02	7.45	6	17.454	0	DDX54	High	High
1025854285268430000	P56377	643884.8	499895.1	643884.8	271452.8	15	3	6	1	157	5.47	8.14	3	9.358	0	AP1S2	High	High
-5003304044033610000	O43175	57603676	44710600	57603676	24278725	32	15	56	15	533	6.71	85.14	15	98.904	0	PHGDH	High	High
8505612402410570000	P17900	8122404	6303486	8122404	3422916	9	2	4	2	193	5.31	6.07	2	6.025	0	GM2A	High	High
-4560958255263080000	Q9Y6M9	247283	191846.4	247283	104176.3	11	1	2	1	179	8.38	0	1	4.943	0	NDUFB9	High	High
-153889659346	P23528	2.89E+08	2.24E+08	2.89E+08	1.22E+08	62	18	76	12	166	8.09	118.27	18	108.493	0	CFL1	High	High

6490000																		
4503550903694700000	O60504	649042.8	503359.3	649042.8	273333.9	2	1	2	1	671	9.45	2.64	1	4.666	0	SORBS3	High	High
-5215357132518720000	P30040	14813396	11481484	14813396	6234669	35	9	31	9	261	7.31	32.4	9	39.316	0	ERP29	High	High
1941330484708970000	P18621	46308621	35889758	46308621	19488836	42	8	31	8	184	10.17	37.7	8	37.195	0	RPL17	High	High
6978617919950450000	O43592	9931724	7694011	9931724	4177998	11	10	18	10	962	5.39	32.92	10	37.165	0	XPOT	High	High
-4122456609389430000	Q04724	953072	738333.8	953072	400929.6	3	2	5	2	770	7.24	0	2	4.965	0	TLE1	High	High
-104196132274101000	Q9GZT4	300411.2	232723.9	300411.2	126373.6	6	2	3	2	340	6.54	0	2	5.252	0	SRR	High	Peak Found
4405181458594490000	Q9NYL2	220327	170622.6	220327	92651.4	3	2	3	2	800	7.87	4.47	2	5.72	0	ZAK; MAP3K20	High	High

-8470537921174600000	Q9NZM1	48359479	37428990	48359479	20324669	23	43	92	43	2061	6.18	111.19	43	165.563	0	MYOF	High	High
1734790582977370000	P54136	73020755	56490522	73020755	30675452	40	26	76	26	660	6.68	109.28	26	119.376	0	RARS	High	High
6241342632728060000	O43148	4678719	3617964	4678719	1964625	11	5	8	5	476	6.61	7.39	5	15.476	0	RNMT	High	High
-4915588766185390000	Q86U44	708206.6	547553.3	708206.6	297332.1	3	2	2	2	580	6.42	2	2	5.551	0	METTL3	High	Peak Found
8452486629110950000	Q9BVC6	28400416	21952149	28400416	11920444	13	4	8	4	243	10.48	14.12	4	14.025	0	TMEM109	High	High
-4518477556068480000	P30046	932676.3	720389.4	932676.3	391185.5	23	2	3	2	118	7.3	2.18	2	9.135	0	DDT	High	High
7226837977459100000	P27701	2397060	1851300	2397060	1005292	9	2	3	2	267	5.24	2.61	2	4.825	0	CD82	High	High

-5156803787758570000	Q14444	27240996	21038722	27240996	11424435	12	10	16	10	709	5.25	19.59	10	33.614	0	CAPRIN1	High	High
57787487465406300	Q08945	5150191	3976511	5150191	2159323	12	8	17	8	709	6.87	4.08	8	21.412	0	SSRP1	High	High
-5378807455886260000	Q13740	15644315	12077784	15644315	6558472	18	10	29	10	583	6.25	39.05	10	42.038	0	ALCAM	High	High
-2935309689569440000	Q9BYD6	1746103	1347967	1746103	731972	9	3	6	3	325	8.78	7.16	3	11.441	0	MRPL1	High	High
710012249327667000	P48449	11538740	8907238	11538740	4836803	21	13	30	13	732	6.61	34.62	13	49.335	0	LSS	High	High
7075703887839200000	Q14CX7	2866706	2212286	2866706	1201314	6	6	9	6	972	6.64	7.16	6	16.404	0	NAA25	High	High
1047661720353370000	Q03426	776191.1	598809.4	776191.1	325165.2	6	2	3	2	396	6.46	1.98	2	6.061	0	MVK	High	High
-103706969365	P31943	67910894	52377310	67910894	28441897	29	10	41	5	449	6.3	60.12	10	71.107	0	HNRNPH1	High	High

2570000																		
-4414098033661640000	O43488	10550017	8136523	10550017	4418290	22	6	14	6	359	7.17	30.71	6	28.784	0	AKR7A2	High	High
-502282286759796000	O00411	328195.6	253092	328195.6	137433.9	1	1	1	1	1230	8.98	0	1	2.717	0.005	POLRMT	High	Peak Found
3893915452985840000	Q9Y3T9	2185392	1684723	2185392	914837.3	5	4	6	4	749	5.62	5.37	4	10.007	0	NOC2L	High	High
-1037081558044950000	P21796	16145043	12446011	16145043	6758426	30	7	23	7	283	8.54	40.94	7	38.186	0	VDAC1	High	High
-7824590419555300000	P63165	2748479	2118287	2748479	1150271	29	3	7	3	101	5.52	7.71	3	8.036	0	SUMO1	High	High
-7401329575601910000	P13674	31777760	24484608	31777760	13295618	35	18	58	18	534	6.01	93.94	18	114.337	0	P4HA1	High	High
-878843	Q92621	7572615	5834184	7572615	3168075	6	13	22	13	20	6.19	19.19	13	41.991	0	NUP205	High	High

9396421030000										12								
4913950505531810000	Q96GA7	552765.8	425555.4	552765.8	231084.9	4	1	2	1	329	6.89	3.88	1	4.086	0	SDSL	High	Peak Found
-8364917298053460000	Q9UQ88	3932469	3026668	3932469	1643540	6	5	10	5	783	5.36	3.55	5	13.547	0	CDK11A	High	High
-3651526396460300000	P17693	780783.1	600920.4	780783.1	326311.5	7	2	6	1	338	5.69	15.03	2	11.159	0	HLA-G	High	Peak Found
7748730165992040000	Q6UWE0	539895	415474.8	539895	225610.9	3	2	2	2	723	5.94	2.79	2	9.501	0	LRSAM1	High	Peak Found
-6346583217180840000	P09601	907833.3	698479.8	907833.3	379288.1	8	2	3	2	288	8.25	4.15	2	7.057	0	HMOX1	High	High
-7972522835468460000	O43432	1872997	1441036	1872997	782510.8	6	9	20	3	1585	5.38	20.19	9	30.578	0	EIF4G3	High	Peak Found

1535897714363240000	Q15019	9897084	7610530	9897084	4132666	28	9	20	9	361	6.6	24.03	9	35.304	0	SEPT2	High	High
-6508547570803100000	Q00169	999555.2	768613.7	999555.2	417372.2	8	2	5	1	270	6.55	8.95	2	8.342	0	PITPNA	High	High
-8133999276561540000	Q86X55	7717401	5933484	7717401	3221997	13	7	15	7	608	6.73	16.66	7	22.849	0	CARM1	High	High
-2006906941817680000	Q9Y520	633126.4	486505.2	633126.4	264181.8	0	1	2	1	2896	9.13	4.36	1	4.048	0	PRRC2C	High	High
-4235464357787340000	Q15370	5447422	4185740	5447422	2272938	38	4	8	4	118	4.88	5.68	4	11.289	0	TCEB2; ELOB	High	High
6370225409205050000	Q9UHX1	26345320	20241081	26345320	10991301	22	12	43	12	559	5.29	54.91	12	55.982	0	PUF60	High	High
-717220726012	P11172	8125015	6242255	8125015	3389666	17	8	19	8	480	7.24	13.93	8	23.869	0	UMPS	High	High

3360000																		
-1017068245716450000	O95782	41879886	32175221	41879886	17471771	33	31	80	22	977	7.03	99.3	31	111.616	0	AP2A1	High	High
-7382790352375510000	Q15393	33393758	25654428	33393758	13930853	19	21	54	21	1217	5.26	64.26	21	85.593	0	SF3B3	High	High
4405938644803010000	Q9HDC9	46067072	35389882	46067072	19217394	39	17	43	17	416	6.16	38.43	17	58.194	0	APMAP	High	High
-7405730642826190000	P00390	13751885	10562644	13751885	5735721	28	10	28	10	522	8.5	40.11	10	49.613	0	GSR	High	High
6514754949609890000	Q9UQE7	19444075	14932496	19444075	8108635	20	22	35	22	1217	7.18	27.5	22	55.131	0	SMC3	High	High
-431742904065760000	O75955	680650	522672.5	680650	283821.3	8	3	3	3	427	7.49	3.45	3	7.467	0	FLOT1	High	Peak Found
-720012431907	P35998	46900878	36013389	46900878	19555970	57	22	57	22	433	5.95	75.28	22	103.972	0	PSMC2	High	High

7870000																		
-1819125433981470000	Q15404	7237359	5555519	7237359	3016755	13	4	10	4	277	8.65	10.39	4	10.132	0	RSU1	High	High
1318421355123860000	P15291	704484.1	540697.3	704484.1	293609.2	4	2	3	2	398	8.65	1.74	2	4.91	0	B4GALT1	High	High
-2091961222017630000	Q13123	311843.3	239323.6	311843.3	129957.4	2	1	3	1	557	6.64	3.6	1	2.509	0.006	IK	High	Peak Found
-5036533815918110000	Q9NP72	7484556	5743208	7484556	3118674	20	4	10	4	206	5.24	15.83	4	14.484	0	RAB18	High	High
4989435524198560000	P48506	463658.1	355678.6	463658.1	193140.4	4	3	5	2	637	6.09	3.62	3	7.816	0	GCLC	High	Peak Found
7234885358333900000	O75915	10010169	7677647	10010169	4169112	20	3	8	3	188	9.77	7.87	3	10.962	0	ARL6IP5	High	High
481963025818	P02792	11152990	8552995	11152990	4644442	37	7	15	7	175	5.78	15.83	7	34.772	0	FTL	High	High

9500000																		
5981355964815440000	P35611	9361927	7178250	9361927	3897929	11	7	23	7	737	5.83	37.78	7	32.317	0	ADD1	High	High
-5365621191326050000	P54819	15058784	11543572	15058784	6268384	44	9	25	9	239	7.81	33.58	9	42.828	0	AK2	High	High
5729262198654750000	O43396	10484984	8030736	10484984	4360846	40	9	16	9	289	4.96	21.29	9	46.895	0	TXNL1	High	High
9091705123101460000	P99999	14813319	11344290	14813319	6160170	39	5	10	5	105	9.57	11.8	5	23.247	0	CYCS	High	High
2329486021897550000	P78347	5728635	4384258	5728635	2380737	12	12	18	12	998	6.39	21.84	12	38.619	0	GTF2I	High	High
-4306142825200870000	P11766	50413406	38578447	50413406	20948847	25	11	40	11	374	7.49	49.55	11	53.144	0	ADH5	High	High
-288029560002	Q8WW59	2011897	1539257	2011897	835846.4	15	3	5	3	207	6.93	4.27	3	7.306	0	SPRYD4	High	High

4900000																		
4157182932771800000	Q07955	28991442	22179582	28991442	12043944	32	9	40	9	248	10.36	54.9	9	37.957	0	SRSF1	High	High
4087674429968210000	P63010	45436704	34759429	45436704	18875046	25	23	60	11	937	5.38	57.05	23	83.998	0	AP2B1	High	High
-2309568012740460000	Q02750	3887731	2973783	3887731	1614822	15	6	20	3	393	6.62	11.31	6	23.046	0	MAP2K1	High	High
-1553618402164790000	O96005	6367799	4864883	6367799	2641726	11	7	13	7	669	6.3	9.57	7	19.666	0	CLPTM1	High	High
-7097724274168320000	Q03701	2958440	2259394	2958440	1226895	5	6	9	6	1054	5.94	9.12	6	17.478	0	CEBPZ	High	High
-93138434393619400	Q9NTI5	841540.2	642588.2	841540.2	348937.9	2	3	5	3	1447	8.47	0	3	9.782	0	PDS5B	High	High
873234617547	Q86UP2	18412818	14058740	18412818	7634169	18	23	43	23	13	5.64	51.44	23	75.224	0	KTN1	High	High

0670000										57								
-1630318732500350000	P62280	81089701	61912439	81089701	33619658	51	10	39	10	158	10.3	55.64	10	32.695	0	RPS11	High	High
7001879318500670000	P11940	1.32E+08	1.01E+08	1.32E+08	54888570	38	24	70	19	636	9.5	106.91	24	116.962	0	PABPC1	High	High
-2305783293341840000	Q14204	1.31E+08	99661059	1.31E+08	54117893	26	113	269	113	4646	6.4	291.99	113	426.201	0	DYNC1H1	High	High
-5768546020460860000	O15160	1495782	1141560	1495782	619889.2	12	3	6	3	346	5.5	5.02	3	12.072	0	POLR1C	High	High
2130579522809330000	Q14534	3287763	2509066	3287763	1362472	13	5	9	5	574	8.63	8.36	5	19.627	0	SQLE	High	High
602378726444352000	O75828	2668758	2036179	2668758	1105685	40	11	28	7	277	6.18	43.71	11	55.529	0	CBR3	High	High
-623213905995	O94874	5999411	4577329	5999411	2485579	12	9	16	9	794	6.79	15.94	9	28.03	0	UFL1	High	High

2850000																		
-7489520263377730000	Q96DI7	3991257	3045065	3991257	1653530	11	4	6	4	357	8.1	5.97	4	12.796	0	SNRNP40	High	High
-8184691105517630000	Q9P035	3905912	2979550	3905912	1617954	14	5	11	5	362	8.94	7.61	5	12.919	0	PTPLAD1; HACD3	High	High
5104868076877430000	Q9HC38	6299499	4803718	6299499	2608512	14	4	12	4	313	5.6	17.45	4	19.888	0	GLOD4	High	High
-7233412111740590000	P19367	35089227	26754610	35089227	14528273	22	18	40	15	917	6.8	65.11	18	73.603	0	HK1	High	High
5853240527723210000	Q9H7Z7	2883601	2198225	2883601	1193679	12	4	8	4	377	9.16	13.71	4	15.074	0	PTGES2	High	High
-3353134017530950000	Q9Y5K5	2807617	2139993	2807617	1162058	17	5	10	5	329	5.33	5.8	5	17.438	0	UCHL5	High	High
-699160	Q14789	6159616	4694645	6159616	2549283	4	13	15	13	32	5	8.7	13	37.783	0	GOLGB1	High	High

270543076000										59								
1714630333721310000	Q6NVY1	1534373	1169445	1534373	635031.6	5	2	3	2	386	8.19	4.03	2	4.952	0	HIBCH	High	High
-8778274040303130000	Q12929	6251500	4763454	6251500	2586648	15	10	16	10	822	7.5	5.86	10	29.828	0	EPS8	High	High
-6496925005546950000	Q13509	59109346	45012177	59109346	24442487	47	22	211	7	450	4.93	320.79	22	140.804	0	TUBB3	High	High
9177231757861530000	P25685	26659041	20300652	26659041	11023649	20	7	14	6	340	8.63	11.4	7	23.931	0	DNAJB1	High	High
1761615694951130000	P62888	41395436	31518878	41395436	17115363	43	6	31	6	115	9.63	54.42	6	36.366	0	RPL30	High	High
-6980948205033410000	Q8WXI9	467345.8	355740.5	467345.8	193174	6	2	2	1	593	9.7	2.24	2	6.203	0	GATAD2B	Peak Found	High
502609981125	P11166	6781971	5162127	6781971	2803135	5	3	7	3	492	8.72	3.96	3	10.239	0	SLC2A1	High	High

4180000																		
3200158126373330000	Q6IBS0	10375971	7897186	10375971	4288325	32	9	20	8	349	6.84	16.83	9	29.198	0	TWF2	High	High
-3079353284966780000	P27635	36316172	27614727	36316172	14995334	30	8	32	8	214	10.08	43.68	8	33.88	0	RPL10	High	High
-6258947584785220000	P62906	86911175	66054762	86911175	35869020	44	10	35	10	217	9.94	59.19	10	62.194	0	RPL10A	High	High
-9010932242184770000	P10644	4887286	3714369	4887286	2016975	13	5	8	5	381	5.35	3.63	5	13.357	0	PRKAR1A	High	High
1188306556528860000	Q15691	18285187	13890026	18285187	7542554	40	11	27	10	268	5.14	31.62	11	45.312	0	MAPRE1	High	High
-5982542769367780000	O75116	4112576	3123606	4112576	1696179	9	12	19	9	1388	6.02	19.02	12	37.395	0	ROCK2	High	High

-1337195466672690000	Q9BZX2	1566192	1189085	1566192	645696	14	3	4	3	261	6.7	4.23	3	10.649	0	UCK2	High	High
-2983241461472980000	P21266	10287942	7809848	10287942	4240899	40	8	27	8	225	5.54	28.03	8	32.041	0	GSTM3	High	High
4010780836197460000	Q15427	1620829	1230363	1620829	668110.8	3	1	2	1	424	8.56	2.38	1	7.313	0	SF3B4	High	High
-2084755087725050000	P05023	66598773	50548751	66598773	27448954	34	30	71	30	1023	5.49	96.59	30	126.817	0	ATP1A1	High	High
-4978148206285390000	Q8TCJ2	4582674	3477868	4582674	1888550	6	5	14	5	826	8.91	11.76	5	18.923	0	STT3B	High	High
2558659697255910000	Q92747	9555656	7251378	9555656	3937639	27	9	22	9	370	8.18	15.43	9	28.976	0	ARPC1A	High	High
7001709167677340000	Q9Y305	2377818	1804349	2377818	979796.6	13	5	8	5	439	8.6	4.04	5	17.149	0	ACOT9	High	High

-7238306351176710000	Q14974	71261837	54074961	71261837	29363755	21	16	58	16	876	4.78	94.02	16	89.769	0	KPNB1	High	High
240711896831941000	P23396	1.86E+08	1.41E+08	1.86E+08	76535366	72	20	107	20	243	9.66	154.27	20	94.596	0	RPS3	High	High
4471349255838510000	P23526	50948275	38639371	50948275	20981930	42	18	64	17	432	6.34	87.89	18	87.741	0	AHCY	High	High
5195647642425590000	O43795	52998417	40189362	52998417	21823605	20	23	55	23	1136	9.38	63.7	23	91.645	0	MYO1B	High	High
6529470217009580000	O15228	223836.2	169728.4	223836.2	92165.84	2	1	1	1	680	6.57	0	1	2.377	0.008	GNPAT	High	Peak Found
699961986535192000	P84095	1448040	1097903	1448040	596182.6	17	2	3	2	191	8.12	8.21	2	10.841	0	RHOG	High	High
-2641060010654770000	O00232	19441724	14739656	19441724	8003920	29	14	25	14	456	7.65	36.15	14	49.639	0	PSMD12	High	High
-505337551256	Q0VDF9	1294908	981289	1294908	532859	8	4	4	4	509	5.59	1.8	4	9.192	0	HSPA14	High	High

8540000																		
-9971868778520760000	Q9NYL9	3649069	2765164	3649069	1501538	13	4	8	4	352	5.19	14.43	4	17.45	0	TMOD3	High	High
9216293239382480000	Q96TA2	1585551	1201390	1585551	652378.2	8	5	8	5	773	8.76	1.91	5	12.632	0	YME1L1	High	High
-1612190288293860000	P60842	2.34E+08	1.77E+08	2.34E+08	96093629	51	22	111	13	406	5.48	194.59	22	141.37	0	EIF4A1	High	High
-5691153666596770000	Q15746	1589539	1204216	1589539	653912.5	2	3	4	3	1914	6.15	3.9	3	7.559	0	MYLK	High	High
4578218238237370000	Q13336	1164104	881834	1164104	478853	4	2	3	2	389	7.15	0	2	4.664	0	SLC14A1	High	Peak Found
-2771631356277900000	Q01650	9759461	7387321	9759461	4011459	13	5	15	5	507	7.72	20.98	5	37.241	0	SLC7A5	High	High
-748536286897	P14174	31713718	23998539	31713718	13031673	8	1	6	1	115	7.88	15.17	1	4.214	0	MIF	High	High

2290000																		
-667534914643 37200	Q13813	1.38E+08	1.04E+08	1.38E+08	56554233	41	92	203	92	2472	5.35	305.54	92	431.67	0	SPTAN1	High	High
8980263924004460000	P28340	1866961	1411693	1866961	766576.6	5	5	7	5	1107	7.03	3.49	5	12.615	0	POLD1	High	High
8320203953169680000	Q9NZL9	7004598	5294595	7004598	2875068	24	6	15	6	334	7.36	24.82	6	24.776	0	MAT2B	High	High
9003669345530140000	Q15738	10816265	8174756	10816265	4439052	31	9	15	9	373	8.06	8.24	9	25.544	0	NSDHL	High	High
-5779447610884950000	Q9NPD8	3823544	2889193	3823544	1568888	11	2	2	2	197	7.99	0	2	4.377	0	UBE2T	High	Peak Found
4839928451091060000	Q86SF2	1704733	1287794	1704733	699297	2	2	7	2	657	7.11	0	2	5.139	0	GALNT7	High	High
4955977949004150000	Q9NRW3	870038.8	657225.7	870038.8	356886.3	13	2	4	2	190	7.59	2.63	2	13.216	0	APOBEC3C	High	High

-6676189443443120000	Q9P273	479957.8	362523.5	479957.8	196857.3	1	2	2	2	2699	6.42	1.95	2	4.022	0	TENM3	High	Peak Found
-2508229497243420000	P82673	571688.5	431525.6	571688.5	234326.8	6	2	4	2	323	8.24	0	2	5.311	0	MRPS35	High	High
-9100750251972440000	Q14C86	1188108	896716.5	1188108	486934.5	2	3	4	3	1478	5.22	2.26	3	7.99	0	GAPVD1	High	High
6440494837518410000	Q9Y3B3	7793452	5877692	7793452	3191701	18	4	12	4	224	6.89	13.35	4	19.077	0	TMED7; TMED7-TICAM2	High	High
3541019235990540000	Q9UNM6	20719620	15625255	20719620	8484817	39	15	30	15	376	5.81	25.89	15	48.466	0	PSMD13	High	High
4723246947420560000	O94915	296141.9	223323.9	296141.9	121269.2	1	2	2	2	3013	5.58	0	2	3.866	0	FRYL	High	Peak Found
-1824011876683040000	Q9ULA0	5549690	4184393	5549690	2272207	13	6	12	6	485	7.58	8.3	6	24.803	0		High	High

6942352143814950000	P49368	95806248	72191726	95806248	39201511	55	28	87	28	545	6.49	168.13	28	140.086	0	CCT3	High	High
-3731678403857660000	P10768	6396133	4819355	6396133	2617004	22	6	20	6	282	7.02	21.96	6	25.263	0	ESD	High	High
5002411829564880000	Q9GZQ8	7647096	5761640	7647096	3128682	22	3	9	3	125	8.94	2.05	3	11.112	0	MAP1LC3B	High	High
-7175242905837580000	O95747	8916004	6717187	8916004	3647563	25	10	23	8	527	6.43	16.97	10	34.709	0	OXSR1	High	High
564322716370963000	O14880	2575192	1939738	2575192	1053316	14	2	3	2	152	9.38	6.64	2	7.085	0	MGST3	High	High
-6890346438132180000	Q14165	3301177	2485789	3301177	1349832	17	4	6	4	292	5.41	8.59	4	13.41	0	MLEC	High	High
-5986352754293270000	P61513	20210917	15216721	20210917	8262975	51	4	11	4	92	10.43	20.95	4	21.917	0	RPL37A	High	High

-6332903783671190000	P62070	4269162	3214001	4269162	1745265	25	4	13	2	204	6.01	13.31	4	14.054	0	RRAS2	High	High
5094708502747750000	P61313	16839046	12674135	16839046	6882302	36	7	17	7	204	11.62	26.76	7	27.637	0	RPL15	High	High
2652497111054870000	Q96HE7	24404996	18359544	24404996	9969589	37	15	33	15	468	5.68	32.49	15	61.223	0	ERO1L; ERO1A	High	High
7904029165854260000	P62191	47508601	35717263	47508601	19395168	53	19	46	18	440	6.21	76.71	19	81.896	0	PSMC1	High	High
1748573463469300000	Q99661	4726022	3552149	4726022	1928886	14	10	16	9	725	7.83	13.53	10	30.447	0	KIF2C	High	High
-7290473429658360000	Q8IXM6	1439865	1082032	1439865	587564.5	6	2	5	2	262	8.63	5.5	2	4.63	0	NRM	High	High
-4485483695822680000	Q9Y5R8	963476	724005.8	963476	393149.3	12	2	3	2	145	9.16	0	2	3.68	0.001	TRAPPC1	High	High

8705476780399290000	P46940	1.1E+08	82337227	1.1E+08	44710715	31	50	135	50	1657	6.48	179.72	50	211.591	0	IQGAP1	High	High
7463522735319840000	O15031	472610.6	354938.1	472610.6	192738.3	0	1	2	1	1838	6.24	1.78	1	2.835	0.004	PLXNB2	High	High
4369524592651400000	P43490	15722024	11801662	15722024	6408532	30	12	36	12	491	7.15	46.98	12	47.893	0	NAMPT	High	High
-652499996560304000	P32969	20615114	15472657	20615114	8401954	23	5	12	5	192	9.95	19.86	5	26.735	0	RPL9	High	High
-4827998788629080000	Q12834	1918453	1439208	1918453	781518	6	3	6	3	499	9.23	10.66	3	10.983	0	CDC20	High	High
1477701677307100000	P27694	10379224	7785357	10379224	4227600	18	10	28	10	616	7.21	30.22	10	42.85	0	RPA1	High	High
-3661455698439900000	Q96AG4	32180574	24137887	32180574	13107342	30	9	34	9	307	9.57	45.17	9	33.081	0	LRRC59	High	High

-4533705047032380000	P62241	34102965	25578273	34102965	13889500	51	9	32	9	208	10.32	54.35	9	43.386	0	RPS8	High	High
627927697280554000	O60841	9266427	6948243	9266427	3773031	14	12	30	12	1220	5.49	50.51	12	61.032	0	EIF5B	High	High
-1518063030880020000	P12814	1.11E+08	83368710	1.11E+08	45270830	58	48	161	32	892	5.41	272.65	48	283.643	0	ACTN1	High	High
412989267492622000	Q12874	20291155	15211108	20291155	8259928	24	10	26	10	501	5.38	32.51	10	55.205	0	SF3A3	High	High
-5605315492068940000	Q16270	2316678	1736540	2316678	942974.9	22	4	7	4	282	7.9	4.64	4	11.854	0	IGFBP7	High	High
7688952667704270000	O14818	27743893	20793165	27743893	11291093	39	10	36	10	248	8.46	69.83	10	56.467	0	PSMA7	High	High
5513100547130870000	P13797	85743327	64239796	85743327	34883458	43	25	75	21	630	5.6	138.75	25	129.2	0	PLS3	High	High

3289616204838970000	P62851	69091332	51752847	69091332	28102802	30	5	19	5	125	10.11	27.83	5	19.509	0	RPS25	High	High
6256395688163030000	O75643	18851137	14118701	18851137	7666729	13	28	49	28	2136	6.06	38.89	28	81.128	0	SNRNP200	High	High
2123612860387900000	P08243	6459955	4838101	6459955	2627183	16	9	19	9	561	6.86	7.92	9	31.931	0	ASNS	High	High
-2355880035621830000	O15067	11600490	8686638	11600490	4717014	10	12	25	12	1338	5.76	27.33	12	38.906	0	PFAS	High	High
-9134021061211310000	Q9HA77	1479895	1107948	1479895	601637.1	7	3	4	3	564	8.34	7.89	3	13.171	0	CARS2	High	High
2799366669898550000	O00469	55236537	41353277	55236537	22455633	32	21	60	21	737	6.71	63.05	21	84.709	0	PLOD2	High	High
491541445040481000	P02545	91916553	68809230	91916553	37364749	44	34	95	33	664	7.02	166	34	177.72	0	LMNA	High	High
-265190	Q6IQ22	1968811	1473206	1968811	799979.4	16	4	4	3	244	8.41	3.74	4	11.044	0	RAB12	High	Peak Found

665490175000																		
-8633064063620320000	Q6PGP7	3269207	2446158	3269207	1328311	5	7	10	7	1564	7.53	6.2	7	22.877	0	TTC37	High	High
-8415712224574130000	P26196	9816043	7344011	9816043	3987941	24	10	21	10	483	8.66	22.74	10	43.9	0	DDX6	High	High
2703376622299260000	Q9UKF6	1887563	1411876	1887563	766676.3	8	5	8	5	684	5.6	1.79	5	13.215	0	CPSF3	High	High
1245532746290230000	P61353	69148054	51713964	69148054	28081688	46	7	23	7	136	10.56	31.04	7	33.023	0	RPL27	High	High
-7644336301377240000	Q8IZ83	1428554	1068348	1428554	580133.7	6	3	7	3	802	6.79	7.24	3	15.89	0	ALDH16A1	High	High
8429981221702180000	P20340	14397272	10762664	14397272	5844336	35	8	20	3	208	5.54	15.98	8	26.037	0	RAB6A	High	High
-572048462967	Q8WUA2	226455	169282.4	226455	91923.64	3	1	1	1	492	5.92	0	1	3.807	0.001	PPIL4	High	Peak Found

9420000																		
-5434559911423020000	O15460	34050403	25449011	34050403	13819308	33	18	37	18	535	5.71	50.55	18	83.357	0	P4HA2	High	High
-515522588376759000	O60506	45823006	34241792	45823006	18593959	40	21	65	14	623	8.59	96.22	21	97.045	0	SYNCRIP	High	High
6546682892855640000	P12268	64733235	48352426	64733235	26256307	36	18	54	16	514	6.9	95.69	18	82.967	0	IMPDH2	High	High
4754969911618930000	Q5H9R7	785272.5	586534.4	785272.5	318499.6	3	2	2	2	873	4.6	1.79	2	5.619	0	PPP6R3	High	Peak Found
-4683582367495080000	Q6IA86	3200918	2390117	3200918	1297880	7	6	7	6	826	5.96	3.81	6	15.754	0	ELP2	High	High
5926930809007950000	Q92598	35733652	26680172	35733652	14487852	31	25	65	22	858	5.39	86.89	25	107.495	0	HSPH1	High	High
-210619804157	Q9UMS4	26491656	19775321	26491656	10738384	30	12	24	12	504	6.61	40.09	12	53.433	0	PRPF19	High	High

1120000																		
-5258533483115150000	Q53H96	4374343	3264833	4374343	1772868	18	4	19	4	274	7.72	24.58	4	20.387	0	PYCRL; PYCR3	High	High
-7740854368523390000	Q9NR30	39772999	29684111	39772999	16119049	32	25	57	23	783	9.28	67.28	25	96.474	0	DDX21	High	High
-199396300438152000	P25788	29284511	21852029	29284511	11866077	28	8	35	8	255	5.33	53.07	8	40.867	0	PSMA3	High	High
6836075231128360000	Q15008	17247380	12869462	17247380	6988368	37	17	45	17	389	5.62	39.81	17	61.871	0	PSMD6	High	High
-1039523901046500000	P42704	58690604	43791907	58690604	23779857	25	36	79	36	1394	6.13	82.96	36	140.099	0	LRPPRC	High	High
-102790818838241000	O94875	307581.1	229442.8	307581.1	124591.9	2	1	3	1	1100	8.31	2.19	1	3.399	0.002	SORBS2	High	High
488660228545	Q92696	470952	351306.9	470952	190766.5	1	1	3	1	567	5.67	0	1	2.595	0.006	RABGGTA	High	High

2210000																		
-1224423870794080000	P49356	627397.1	468006.5	627397.1	254136.6	4	2	2	2	437	5.82	2.18	2	5.828	0	FNTB; CHURC1-FNTB	High	Peak Found
-6259860464076080000	Q9NR31	26307848	19623413	26307848	10655895	26	7	19	3	198	6.68	23.89	7	21.991	0	SAR1A	High	High
7224643183628000000	O00303	25431223	18958168	25431223	10294654	24	8	22	8	357	5.45	32.18	8	43.735	0	EIF3F	High	High
2618292350510680000	P98082	3670432	2734431	3670432	1484849	7	5	10	5	770	5.53	17.09	5	17.633	0	DAB2	High	High
7326537803977900000	P02786	52746587	39274327	52746587	21326723	37	25	71	25	760	6.61	116.16	25	117.576	0	TFRC	High	High
-2595781739747330000	Q14677	1973105	1468706	1973105	797536	7	4	7	4	625	6.42	6.55	4	15.891	0	CLINT1	High	High
158556248226	O15372	10560220	7857680	10560220	4266873	20	7	16	7	352	6.54	17.2	7	18.674	0	EIF3H	High	High

8550000																		
-7890686096526140000	Q13347	29151033	21689620	29151033	11777885	44	11	39	11	325	5.64	47.09	11	60.769	0	EIF3I	High	High
-729542664091126000	Q93008	1473453	1096267	1473453	595294.1	2	5	6	5	2554	5.8	5.37	5	16.239	0		High	High
1870630596764220000	Q15643	2479924	1844909	2479924	1001821	3	6	6	6	1979	5.26	5.97	6	18.177	0	TRIP11	High	Peak Found
-7270924996778500000	P12110	15421833	11465378	15421833	6225923	12	11	25	11	1019	6.21	39.82	11	40.136	0	COL6A2	High	High
-5982176321518380	Q92572	1434928	1066766	1434928	579274.4	6	1	6	1	193	5.39	8.86	1	4.13	0	AP3S1	High	High
6481945204603060000	Q9UBB6	2543586	1890811	2543586	1026747	12	7	17	7	729	5.48	15.45	7	30.364	0	NCDN	High	High
1978266596079070000	Q01518	1.35E+08	1E+08	1.35E+08	54338491	42	21	77	20	475	8.06	132.82	21	116.278	0	CAP1	High	High

6488855965708190000	P14618	1.07E+09	7.92E+08	1.07E+09	4.3E+08	65	38	223	38	531	7.84	413.33	38	254.702	0	PKM	High	High
-6716088986514920000	Q13617	6582064	4889266	6582064	2654966	21	16	24	16	745	6.92	14.37	16	48.781	0	CUL2	High	High
8498450919961840000	Q14315	1.66E+08	1.23E+08	1.66E+08	67013544	40	92	264	76	2725	5.97	431.82	92	472.956	0	FLNC	High	High
-8588470506095450000	P21912	5680479	4217181	5680479	2290011	11	3	9	3	280	8.76	12.28	3	12.79	0	SDHB	High	High
-4845069297566420000	P35221	29025648	21548051	29025648	11701011	32	26	57	21	906	6.29	76.27	26	104.291	0	CTNNA1	High	High
5673730343044780000	Q96HS1	2403635	1783733	2403635	968601.7	12	4	7	4	289	8.68	0	4	9.388	0	PGAM5	High	High
5007041047885350000	Q15417	6946460	5154646	6946460	2799073	29	7	13	6	329	6.05	15.89	7	30.091	0	CNN3	High	High

8119478820336470000	O14786	2074953	1539145	2074953	835785.9	6	4	8	4	923	5.88	12.24	4	18.313	0	NRP1	High	High
6184402302268640000	Q5JRX3	22216906	16474232	22216906	8945828	27	23	41	23	1037	6.92	48.59	23	84.342	0	PITRM1	High	High
7129667850847220000	P52209	83968044	62244241	83968044	33799833	40	18	57	18	483	7.23	76.35	18	73.57	0	PGD	High	High
-3067082233610550000	Q9Y639	4100555	3039679	4100555	1650605	9	4	9	4	398	7.99	8.79	4	11.977	0	NPTN	High	High
-3241887692250600000	Q15149	3.8E+08	2.82E+08	3.8E+08	1.53E+08	41	188	466	182	4684	5.96	684.12	188	755.448	0	PLEC	High	High
-6896953880380310000	P55072	71032931	52617901	71032931	28572543	36	28	69	28	806	5.26	100.41	28	127.964	0	VCP	High	High
-1205660111365880000	Q92599	991619.3	734286	991619.3	398731.6	10	5	7	3	483	6.28	7.19	5	12.972	0	SEPT8	High	Peak Found

-4347150339173570000	Q92878	10137045	7504911	10137045	4075313	11	14	22	14	1312	6.89	14.95	14	38.655	0	RAD50	High	High
-7237194878275620000	Q9Y224	11318574	8377655	11318574	4549229	40	10	20	10	244	6.65	24.71	10	31.753	0	C14orf166; RTRAF	High	High
-5974744531153520000	Q01581	63951648	47301737	63951648	25685763	40	19	51	19	520	5.41	78.81	19	104.498	0	HMGCS1	High	High
-2846560637843550000	O14579	5420725	4008504	5420725	2176695	23	6	23	6	308	5.12	15.76	6	34.44	0	COPE	High	High
7433951425016970000	Q7L523	3058945	2260362	3058945	1227421	11	3	9	3	313	7.72	17.6	3	15.512	0	RRAGA	High	High
-1866201622056550000	Q13263	16670936	12315842	16670936	6687742	20	16	42	16	835	5.77	58.87	16	74.328	0	TRIM28	High	High
691088126068	Q9UEE9	269175.3	198787	269175.3	107945.2	3	1	2	1	299	4.81	0	1	2.845	0.004	CFDP1	High	High

3280000																		
-8623682461858750000	P54578	27215486	20097652	27215486	10913416	44	17	49	17	494	5.3	65.92	17	80.76	0	USP14	High	High
8408211072324920000	P78344	23940212	17676111	23940212	9598472	22	20	36	20	907	7.14	36.58	20	61.232	0	EIF4G2	High	High
8251896987743930000	P53680	5205466	3838922	5205466	2084609	11	2	8	2	142	6.18	7.18	2	4.557	0	AP2S1	High	High
8658437840418650000	P12109	30166655	22242817	30166655	12078282	16	12	38	12	1028	5.43	50.07	12	49.99	0	COL6A1	High	High
-8452919191869770000	Q8WVV9	1021885	753449.1	1021885	409137.5	2	1	3	1	542	7.72	2.69	1	8.106	0	HNRNPLL; HNRPLL	High	High
-8781846477469750000	P38606	36285973	26749804	36285973	14525663	32	19	59	19	617	5.52	68.19	19	71.866	0	ATP6V1A	High	High
152016408240	P07737	2.83E+08	2.09E+08	2.83E+08	1.13E+08	78	15	96	15	140	8.27	136.23	15	99.148	0	PFN1	High	High

7220000																		
8196142567442190000	P20645	892271.3	657686.1	892271.3	357136.3	8	2	2	2	277	5.83	2.22	2	5.34	0	M6PR	High	Peak Found
-5459324364456550000	Q04637	56222659	41428532	56222659	22496498	26	36	71	31	1599	5.33	85.78	36	142.753	0	EIF4G1	High	High
121458031031999000	P35232	12597533	9281838	12597533	5040218	38	10	30	10	272	5.76	34.9	10	46.012	0	PHB	High	High
-2014809331643560000	Q709F0	477067.5	351497.7	477067.5	190870.1	2	1	3	1	780	8.02	4.79	1	4.964	0		High	High
-5660529774365680000	Q6WCQ1	333228.2	245437.4	333228.2	133277.3	1	1	1	1	1025	6.21	0	1	2.585	0.006	MPRIP	High	Peak Found
7156197728076020000	L0R8F8	476919	351122.7	476919	190666.4	29	2	2	2	70	10.58	0	2	3.245	0.002	SMCR7L; MIEF1	High	Peak Found
-873791131724	Q15631	6882545	5066196	6882545	2751043	17	4	9	4	228	6.44	8.78	4	12.261	0	TSN	High	High

3540000																		
-5836561258245120000	Q6P6C2	446620.6	328404.6	446620.6	178330.1	3	1	1	1	394	9.09	2	1	4.469	0	ALKBH5	High	Peak Found
-283870965535324000	Q9UNH6	1259652	926158.1	1259652	502921.9	5	2	6	2	387	5.11	3.57	2	7.566	0	SNX7	High	High
8315542899134670000	O94804	843460.9	620064.1	843460.9	336706.9	4	4	6	3	968	6.95	3.67	4	12.665	0	STK10	High	Peak Found
3211102690049460000	P11310	8360114	6145485	8360114	3337118	18	7	16	7	421	8.37	22.22	7	26.236	0	ACADM	High	High
8110306690791420000	P29218	3813952	2803511	3813952	1522361	21	6	11	6	277	5.26	2.24	6	17.789	0	IMPA1	High	High
5464068146291930000	P49840	611703.3	449633.2	611703.3	244159.6	7	4	7	1	483	8.75	0	4	9.871	0	GSK3A	High	High
96752444028096400	Q8WWC4	270619	198738.7	270619	107919	5	1	2	1	291	9.17	1.62	1	6.647	0	C2orf47; MAIP1	High	High

-8431694603937650000	P15531	1.09E+08	80269959	1.09E+08	43588148	66	9	47	4	152	6.19	78.41	9	53.294	0	NME1	High	High
9050576250017760000	P60604	471250.7	345876.6	471250.7	187817.7	22	2	3	2	165	4.7	0	2	5.53	0	UBE2G2	High	Peak Found
1566902771694480000	P51153	5101921	3743872	5101921	2032995	33	7	27	4	203	9.19	32.88	7	26.385	0	RAB13	High	High
-4844902181037290000	P62314	5203227	3818086	5203227	2073295	38	4	13	4	119	11.56	18.27	4	19.389	0	SNRPD1	High	High
2821733550000510000	Q9NX46	3529073	2588663	3529073	1405694	13	4	15	4	363	5.07	17.32	4	16.221	0	ADPRHL2	High	High
-1051331415144910000	P49189	10947885	8030241	10947885	4360577	15	7	12	7	494	5.87	11.15	7	27.969	0	ALDH9A1	High	High
3876031868592720000	Q9BXF6	364283.9	267182	364283.9	145085	1	1	2	1	653	9.23	1.68	1	2.49	0.007	RAB11FIP5	High	High

2874142018275290000	A6NHR9	2610881	1914832	2610881	1039791	4	8	14	8	2005	7.3	9.59	8	18.538	0	SMCHD1	High	High
-3441186793177340000	Q969M3	1463559	1072912	1463559	582612.1	5	1	5	1	257	4.36	11.5	1	6.257	0	YIPF5	High	High
1970257073034710000	Q6RW13	2183098	1599525	2183098	868573	16	2	5	2	159	6.14	4.7	2	6.134	0	AGTRAP	High	High
1046790815186600000	Q8TB22	902771.6	661017.1	902771.6	358945.1	6	4	6	4	786	7.43	5.62	4	12.923	0	SPATA20	High	Peak Found
-573717286669998000	Q9BY43	4286342	3138309	4286342	1704163	14	2	2	2	222	4.7	1.63	2	6.711	0	CHMP4A	High	High
6460055541994450000	Q14019	34238951	25059948	34238951	13608039	61	9	24	9	142	5.67	27.04	9	32.298	0	COTL1	High	High
3813032805198600000	Q9NVA2	11762590	8609080	11762590	4674898	25	10	20	8	429	6.81	25.18	10	41.528	0	SEPT11	High	High
227235059761	O00743	2752388	2014346	2752388	1093829	10	3	7	3	305	5.69	3.96	3	10.213	0	PPP6C	High	High

9460000																		
-2227436002470210000	P05141	1.83E+08	1.34E+08	1.83E+08	72599442	32	11	49	6	298	9.69	84.25	11	50.938	0	SLC25A5	High	High
-8479423548841090000	P21283	9917408	7256737	9917408	3940549	31	13	23	13	382	7.46	14.11	13	33.514	0	ATP6V1C1	High	High
1494220967360300000	Q96KA5	2158643	1578796	2158643	857316.8	9	4	7	4	538	8.56	8.45	4	14.136	0	CLPTM1L	High	High
-2534211096191500000	O00560	1011053	739314	1011053	401461.9	4	1	4	1	298	7.53	4.85	1	4.988	0	SDCBP	High	High
-319216844392798000	Q9Y4R8	962592.8	703436.9	962592.8	381979.9	3	2	2	2	837	5.76	1.88	2	5.16	0	TELO2	High	Peak Found
-1162437005396670000	Q99442	4709647	3441454	4709647	1868776	10	4	13	4	399	7.12	11.97	4	18.152	0	SEC62	High	High

-6565119968553580000	Q01433	7863086	5745482	7863086	3119909	10	9	14	9	879	6.93	12.62	9	22.671	0	AMPD2	High	High
3114131480513410000	P62701	1.94E+08	1.42E+08	1.94E+08	77132204	62	19	68	19	263	10.15	109.63	19	87.588	0	RPS4X	High	High
-6831775040715790000	Q15043	364551.5	266111.4	364551.5	144503.7	2	1	2	1	492	5.33	0	1	3.589	0.001	SLC39A14	High	High
-976033423709961000	Q96A26	3384360	2470444	3384360	1341499	25	4	5	4	154	9.77	6.24	4	9.067	0	FAM162A	High	High
-8280603052220460000	P78357	4683449	3417487	4683449	1855762	7	9	13	9	1384	7.05	14.85	9	29.238	0	CNTNAP1	High	High
-8993945663539590000	P61026	14474121	10560917	14474121	5734784	50	9	25	6	200	8.38	24.85	9	28.492	0	RAB10	High	High
2992293195289620000	Q15005	12194971	8897180	12194971	4831342	50	11	22	11	226	8.47	19.2	11	31.598	0	SPCS2	High	High

-7195591263418840000	P29373	2199962	1604550	2199962	871301.7	12	2	4	2	138	5.4	1.73	2	5.848	0	CRABP2	High	High
-6011940264998510000	O00429	8883493	6478696	8883493	3518058	19	13	21	13	736	6.81	20.23	13	42.543	0	DNM1L	High	High
4198310778640920000	Q9Y3F4	21988134	16031715	21988134	8705533	35	11	25	11	350	5.12	36.44	11	47.36	0	STRAP	High	High
-7438852567896600000	Q7L5N1	6011287	4381965	6011287	2379492	28	7	17	7	327	5.73	10.73	7	30.315	0	COPS6	High	High
-2767928129327740000	O14950	73241254	53387833	73241254	28990631	51	9	30	4	172	4.84	37.48	9	36.477	0	MYL12B; MYL12A	High	High
-5217952952055260000	P49207	14998679	10929350	14998679	5934849	36	5	11	5	117	11.47	11	5	13.384	0	RPL34	High	High
356572395877	Q08378	3504313	2552533	3504313	1386075	10	11	18	11	14	5.44	15.14	11	36.823	0	GOLGA3	High	High

0540000										98								
9192930488046830000	P25789	33333728	24277670	33333728	13183247	31	9	26	9	261	7.72	47.36	9	41.288	0	PSMA4	High	High
4988344905656770000	Q92665	265952.9	193688.2	265952.9	105176.5	4	1	1	1	395	9.29	2.45	1	5.752	0	MRPS31	High	Peak Found
-2550625188107110000	P14550	14393202	10480910	14393202	5691338	26	9	21	8	325	6.79	31.51	9	28.883	0	AKR1A1	High	High
-2887572586952260000	Q9HCE1	6196722	4511983	6196722	2450094	12	11	15	11	1003	8.82	10.57	11	32.052	0	MOV10	High	High
-7065009906831580000	P07900	3.08E+08	2.24E+08	3.08E+08	1.22E+08	49	45	224	25	732	5.02	394.93	45	314.938	0	HSP90AA1	High	High
-4595887854081880000	Q99460	28647708	20856484	28647708	11325476	31	23	49	23	953	5.39	71.52	23	111.026	0	PSMD1	High	High

6124389197736880000	P40925	43485817	31656865	43485817	17190293	35	10	19	10	334	7.36	32.97	10	46.176	0	MDH1	High	High
-930823423680918000	Q53H82	6784454	4938740	6784454	2681832	17	4	6	4	288	6.8	5.33	4	12.416	0	LACTB2	High	Peak Found
6028775668578430000	Q9NZN4	82016891	59701803	82016891	32419240	37	20	57	15	543	6.46	56.09	20	80.492	0	EHD2	High	High
5641207754234890000	Q9H583	151636.8	110312.4	151636.8	59901.76	0	1	1	1	2144	6.54	0	1	2.363	0.008	HEATR1	High	Peak Found
-1034815253104010000	P50990	1.39E+08	1.01E+08	1.39E+08	54829904	53	28	115	28	548	5.6	188.37	28	139.435	0	CCT8	High	High
-7841395992400370000	Q9NVG8	260569.5	189497.8	260569.5	102901	3	1	1	1	400	5.24	0	1	2.535	0.006	TBC1D13	High	Peak Found
-6072286448447600000	Q9UBE0	12481612	9074100	12481612	4927413	28	10	21	10	346	5.3	27.07	10	41.68	0	SAE1	High	High

1274747088330590000	O94973	3669399	2667383	3669399	1448441	13	13	33	4	939	6.96	44.43	13	41.785	0	AP2A2	High	High
-7650985732461440000	Q15035	1538867	1118492	1538867	607362.9	8	3	8	3	370	9.22	7.45	3	10.473	0	TRAM2	High	High
8973265240341480000	Q06203	1932334	1404110	1932334	762458.9	6	3	5	3	517	6.76	5.66	3	11.925	0	PPAT	High	Peak Found
-7575182119282230000	P18669	1.48E+08	1.08E+08	1.48E+08	58405436	49	13	66	10	254	7.18	154.28	13	132.58	0	PGAM1; LOC643576	High	High
-9074819377594510000	Q9BQ67	1762188	1279648	1762188	694874	12	4	7	4	446	4.92	5.92	4	14.756	0	GRWD1	High	High
-5765890645847650000	Q14790	280894.5	203939.7	280894.5	110743.2	4	2	2	2	479	5.1	0	2	6.295	0	CASP8	High	High
407233124236787000	Q96DB5	11231655	8153677	11231655	4427605	20	7	17	6	314	8.5	19.72	7	23.318	0	FAM82B; RMDN1	High	High

769937513119 03600	Q9GZZ9	1145652	831241.5	1145652	451380.3	4	2	4	2	404	4.84	1.87	2	5.073	0	UBA5	High	High
790269224436 9350000	Q9Y6G9	8628606	6257185	8628606	3397773	15	7	22	7	523	6.42	19.56	7	30.633	0	DYNC1LI1	High	High
344344608498 2210000	Q9UN37	1741674	1262669	1741674	685653.6	11	4	7	3	437	7.8	6.19	4	10.973	0	VPS4A	High	Peak Found
-761565841475 1440000	Q86T03	321550.3	233060.9	321550.3	126556.6	8	1	1	1	277	8.91	0	1	2.444	0.007	TMEM55B; PIP4P1	High	Peak Found
476900213143 6570000	Q00610	2.06E+08	1.5E+08	2.06E+08	81186579	37	59	180	59	1675	5.69	283.72	59	314.131	0	CLTC	High	High
-565285794591 5370000	Q9BQ69	2118376	1535288	2118376	833691.1	14	3	5	3	325	9.51	7.92	3	18.833	0	MACROD1	High	High
-313391797621 8080000	Q9Y3D8	1320363	956739.2	1320363	519528	10	2	4	2	172	4.58	3.51	2	5.211	0	AK6; TAF9	High	High
663436327465	Q14839	16951644	12282935	16951644	6669873	10	18	41	13	19	5.86	44.93	18	68.98	0	CHD4	High	High

0920000										12								
7512060777885110000	P63244	1.02E+08	73929225	1.02E+08	40145007	45	14	64	14	317	7.69	125.9	14	103.015	0	GNB2L1; RACK1	High	High
3910954529377130000	Q9UPN3	21846598	15824481	21846598	8593001	5	32	46	32	7388	5.39	44.06	32	110.285	0	MACF1	High	High
-990721738298047000	O00299	90282769	65381891	90282769	35503638	56	11	31	11	241	5.17	38.69	11	54.809	0	CLIC1	High	High
-3620399161639760000	Q99471	2656183	1921557	2656183	1043443	16	3	5	3	154	6.33	5.52	3	6.364	0	PFDN5	High	High
9138201911436170000	Q13952	223061.6	161207.7	223061.6	87538.93	3	1	2	1	458	6.1	0	1	3.751	0.001	NFYC	High	Peak Found
7726317818688940000	Q9NQC3	53543990	38692810	53543990	21010948	10	7	30	7	1192	4.5	58.21	7	62.273	0	RTN4	High	High
-6736379060781020000	P21399	9874047	7134782	9874047	3874326	10	10	18	10	889	6.68	4.63	10	26.488	0	ACO1	High	High

1709691025135320000	O00499	869962.5	628597	869962.5	341340.4	3	2	5	2	593	5.06	1.74	2	3.676	0.001	BIN1	High	High
721169718826189000	Q8IX12	2101906	1518740	2101906	824705.5	4	4	9	4	1150	5.76	3.24	4	8.54	0	CCAR1	High	High
8095037981369690000	P52434	1587888	1147258	1587888	622983.3	27	3	5	3	150	4.68	3.94	3	13.418	0	POLR2H	High	High
2935045971182120000	Q7Z3E5	454854	328454.4	454854	178357.1	3	2	2	2	818	6.2	2.24	2	6.613	0		High	Peak Found
-8778470671322450000	Q71UI9	3049058	2201272	3049058	1195333	31	4	16	2	128	10.58	18.31	4	11.787	0	H2AFV	High	High
-5646758734319930000	Q16555	1.61E+08	1.16E+08	1.61E+08	63115045	42	19	86	18	572	6.38	158.38	19	139.829	0	DPYSL2	High	High
-8752239950939410000	P18754	9939022	7172105	9939022	3894592	19	6	11	6	421	7.52	17.38	6	24.145	0	RCC1	High	High

-2500202292612520000	Q99623	11706613	8446887	11706613	4586824	41	11	23	11	299	9.83	30.53	11	45.911	0	PHB2	High	High
-1684590373995140000	P35237	17313466	12487916	17313466	6781181	42	13	27	13	376	5.27	34.09	13	68.896	0	SERPINB6	High	High
5859818642723020000	Q96RS6	11650125	8400725	11650125	4561757	18	9	19	9	583	5.11	27.93	9	30.865	0	NUDCD1	High	High
-3249613255715030000	P30041	38200204	27533429	38200204	14951187	46	11	26	11	224	6.38	34.3	11	43.648	0	PRDX6	High	High
8428260618384840000	Q15029	8882968	6401557	8882968	3476170	13	12	31	11	972	5	37.8	12	45.077	0	EFTUD2	High	High
4966290939603230000	O00461	2130536	1534890	2130536	833475.3	11	7	8	7	696	4.77	13.07	7	26.341	0	GOLIM4	High	High
5619484675981050000	P10809	3.75E+08	2.7E+08	3.75E+08	1.46E+08	67	40	152	40	573	5.87	314.1	40	225.705	0	HSPD1	High	High

2229585917492550000	P53992	7824765	5633986	7824765	3059364	12	10	23	10	1094	7.06	22.56	10	43.251	0	SEC24C	High	High
4286201540320340000	Q92990	1450211	1044063	1450211	566946.6	6	3	3	3	594	5.33	1.68	3	9.476	0	GLMN	High	Peak Found
-6142144855289120000	Q96HC4	15120686	10884043	15120686	5910247	23	11	29	11	596	8.21	30.44	11	45.25	0	PDLIM5	High	High
4058456233631450000	Q06830	4.3E+08	3.09E+08	4.3E+08	1.68E+08	63	15	97	11	199	8.13	151.16	15	112.283	0	PRDX1	High	High
-4925331307854050000	Q14739	884138.3	635926.3	884138.3	345320.3	3	2	3	2	615	9.36	1.67	2	5.261	0	LBR	High	Peak Found
-5330197461004940000	Q15582	9243839	6641095	9243839	3606244	8	5	14	5	683	7.71	10.46	5	12.917	0	TGFBI	High	High
-2531564844693560000	O75475	4942299	3550554	4942299	1928020	11	6	11	5	530	9.13	10.68	6	12.784	0	PSIP1	High	High

-3295496481374290000	Q9UHD9	863777.5	620330.7	863777.5	336851.7	9	4	4	2	624	5.22	4.35	4	10.75	0	UBQLN2	High	Peak Found
2957906910240920000	P22033	2472143	1775294	2472143	964019.1	5	3	4	3	750	6.93	4.4	3	14.701	0	MUT	High	High
155528844813379000	Q68CZ2	323435.1	232254.9	323435.1	126118.9	2	2	3	2	1445	6.81	0	2	8.72	0	TNS3	High	High
-1108045160068180000	P20073	22227202	15959512	22227202	8666325	30	13	29	13	488	5.68	40.21	13	53.549	0	ANXA7	High	High
3867074619681450000	P21589	1039554	746314.8	1039554	405263.5	8	4	8	4	574	7.03	2.8	4	11.121	0	NT5E	High	High
7216682931187910000	Q8WUP2	3142573	2255835	3142573	1224963	4	1	1	1	373	6.02	0	1	3.062	0.003	FBLIM1	High	Peak Found
1365521486549270000	Q6ICB0	272141	195329.5	272141	106067.7	5	1	1	1	168	4.94	0	1	2.079	0.01	DESI1	High	Peak Found

-9193624318579360000	Q9UGI8	6534389	4689263	6534389	2546361	24	10	17	10	421	7.68	26.49	10	49.355	0	TES	High	High
-2333470476748250000	P56134	1775784	1274313	1775784	691976.8	14	1	2	1	94	9.67	0	1	3.078	0.003	ATP5J2	High	High
3580976773696250000	P07686	13112867	9407898	13112867	5108672	17	10	25	9	556	6.76	26.36	10	34.11	0	HEXB	High	High
-4658645955564380000	Q86Y82	2297926	1648302	2297926	895060	13	3	8	3	276	5.59	8.17	3	9.688	0	STX12	High	High
-383335111069342000	Q6PD74	210936.7	151274.8	210936.7	82145.16	4	1	1	1	315	4.64	0	1	2.942	0.004	AAGAB	High	Peak Found
565785606400917000	P26639	68831583	49358037	68831583	26802374	31	24	63	24	723	6.67	101.25	24	90.329	0	TARS	High	High
-7454477479257430000	P09486	7051205	5054094	7051205	2744471	17	6	14	6	303	4.84	20.32	6	25.025	0	SPARC	High	High

-4497117085705840000	Q9UHY1	352455.1	252385.1	352455.1	137050	3	1	2	1	535	5.08	0	1	2.542	0.006	NRBP1	High	Peak Found
-2158743068394570000	P29401	4.97E+08	3.55E+08	4.97E+08	1.93E+08	44	30	142	30	623	7.66	288.31	30	200.953	0	TKT	High	High
5023215591343920000	Q12768	2725191	1949932	2725191	1058851	7	8	14	8	1159	6.98	7.47	8	24.292	0	KIAA0196; WASHC5	High	High
-5840686282858320000	P17936	944753.8	675919.9	944753.8	367037.7	3	1	3	1	291	8.69	4.1	1	3.158	0.003	IGFBP3	High	High
990936575845663000	Q16706	3499758	2503727	3499758	1359572	6	6	7	6	1144	7.58	9.54	6	15.068	0	MAN2A1	High	High
3949489536824210000	O15162	481714.3	344521.2	481714.3	187081.7	9	2	2	2	318	4.94	0	2	5.346	0	PLSCR1	High	High
6290331068262670000	Q9NRQ2	407735.3	291608.5	407735.3	158349.1	4	1	3	1	329	5.82	0	1	4.425	0	PLSCR4	High	High

-4773459124454050000	P49915	33715327	24106421	33715327	13090255	27	15	49	15	693	6.87	88.06	15	77.76	0	GMPS	High	High
-140730978004471000	Q96AY3	36829932	26323299	36829932	14294063	30	17	53	16	582	5.62	36.84	17	61.699	0	FKBP10	High	High
8211327054168000000	Q11201	457684.4	327064.7	457684.4	177602.5	5	1	5	1	340	9.09	8.71	1	8.703	0	ST3GAL1	High	High
-7543040224215830000	Q96EY4	246786.5	176278.6	246786.5	95722.73	7	1	1	1	203	9.26	0	1	4.974	0	TMA16	High	Peak Found
7727744455345480000	Q13868	729448.7	520973.1	729448.7	282898.5	9	2	3	2	293	7.5	0	2	5.069	0	EXOSC2	High	High
8594312239036200000	Q9H0X4	1071079	764466	1071079	415119.9	5	2	3	2	552	6.28	2.54	2	7.103	0	ITFG3; FAM234A	High	Peak Found
6215285744053410000	Q53H12	10869456	7754481	10869456	4210834	6	3	5	3	422	8.09	3.36	3	6.481	0	AGK	High	High

272489793728892000	Q14696	1901027	1356199	1901027	736442.5	17	4	7	4	234	7.78	6.72	4	10.992	0	MESDC2; MESD	High	High
-8995832376771220000	Q9UBS4	9110393	6496121	9110393	3527520	26	9	15	9	358	6.18	16.07	9	40.476	0	DNAJB11	High	High
4567106574901640000	O60826	220053.5	156836.6	220053.5	85165.3	4	1	1	1	627	6.74	0	1	2.558	0.006	CCDC22	High	Peak Found
-3009900426920290000	B5ME19	44652502	31798684	44652502	17267304	27	26	72	26	914	5.64	104.3	26	102.649	0	EIF3CL	High	High
-1351774447437730000	Q9NQW7	6739256	4799264	6739256	2606094	11	7	12	7	623	5.67	16.34	7	21.693	0	XPNPEP1	High	High
-8311044657227680000	Q9BZE9	528046.3	376036.7	528046.3	204195.3	3	1	1	1	553	6.64	1.84	1	3.01	0.003	ASPSCR1	High	Peak Found
8205136122998850000	P60981	33277430	23695875	33277430	12867321	44	12	32	9	165	7.85	39.84	12	45.522	0	DSTN	High	High

4196328193324920000	Q9HBL0	287914.9	204851.8	287914.9	111238.5	1	1	1	1	1735	7.75	0	1	2.08	0.01	TNS1	High	Peak Found
2014587900474490000	Q9P015	581424.1	413646.8	581424.1	224618.3	7	2	3	2	296	10.01	5.75	2	6.893	0	MRPL15	High	Peak Found
-7310593134022690000	P57081	680010.4	483552	680010.4	262578.1	3	1	2	1	412	7.11	1.78	1	4.1	0	WDR4	High	Peak Found
8724268624419380000	P50213	11266228	8011203	11266228	4350239	23	8	17	8	366	6.92	20.57	8	32.718	0	IDH3A	High	High
3864595288643470000	P61011	7996033	5685560	7996033	3087370	21	8	14	8	504	8.75	22.05	8	28.011	0	SRP54	High	High
-5875198809388000000	Q9H3H3	4039414	2871500	4039414	1559280	12	3	10	3	292	6.32	10.97	3	14.636	0		High	High
-4888368935124000000	Q92692	1148348	816118.9	1148348	443168.5	3	2	4	2	538	4.82	1.75	2	3.325	0.002	PVRL2; NECTIN2	High	High

-7689745804882420000	Q6XQN6	7972627	5664806	7972627	3076100	15	8	19	8	538	5.68	26.84	8	35.438	0	NAPRT1; NAPRT	High	High
5839197527488620000	P09110	2912413	2069304	2912413	1123672	21	5	10	5	424	8.44	6.91	5	23.94	0	ACAA1	High	High
2426861074950700000	P22314	33899352	24080946	33899352	13076422	21	20	54	20	1058	5.76	65.02	20	77.042	0	UBA1	High	High
-7017874985935530000	P18206	3.71E+08	2.63E+08	3.71E+08	1.43E+08	57	58	179	58	1134	5.66	304.08	58	286.3	0	VCL	High	High
-8826261878749580000	P39023	98099207	69611511	98099207	37800404	45	21	60	21	403	10.18	78.53	21	83.512	0	RPL3	High	High
-6182186368676620000	Q13405	199091.2	141247.4	199091.2	76700.07	8	1	3	1	166	9.45	3.54	1	4.351	0	MRPL49	High	Peak Found
-755691628289	O15347	9219481	6540084	9219481	3551393	27	6	10	5	200	8.37	9.05	6	24.85	0	HMGB3	High	High

3620000																		
7247108035044900000	P62244	1.11E+08	79026416	1.11E+08	42912880	56	9	37	9	130	10.13	51.31	9	29.209	0	RPS15A	High	High
-6527665309278310000	O15145	15895525	11272153	15895525	6120998	20	4	16	4	178	8.59	21.23	4	16.683	0	ARPC3	High	High
4388597074234390000	P04083	2.62E+08	1.86E+08	2.62E+08	1.01E+08	60	20	77	20	346	7.02	136.3	20	125.316	0	ANXA1	High	High
-8533826193911870000	Q86VP6	50189225	35588144	50189225	19325054	27	32	82	32	1230	5.78	115.4	32	124.57	0	CAND1	High	High
-4632135778776140000	P18085	1.76E+08	1.25E+08	1.76E+08	67913231	37	8	42	6	180	7.14	67.51	8	31.414	0	ARF4	High	High
-5829960447854690000	O43399	13017870	9224691	13017870	5009187	45	10	20	10	206	5.36	23.52	10	42.628	0	TPD52L2	High	High

-2088102652964780000	Q13310	12474371	8838362	12474371	4799402	22	15	37	10	644	9.26	32.12	15	61.384	0	PABPC4	High	High
-2160621710341350000	O14964	2394621	1695914	2394621	920914.4	8	6	11	6	777	6.16	5.5	6	18.38	0	HGS	High	High
-4567547795878980000	P21333	1.36E+09	9.63E+08	1.36E+09	5.23E+08	56	130	577	116	2647	6.06	1079.39	130	927.878	0	FLNA	High	High
1629883586476670000	Q9BT78	10313658	7299514	10313658	3963778	25	9	23	9	406	5.83	29.55	9	44.183	0	COPS4	High	High
-224896883052759000	P13987	4738924	3352871	4738924	1820674	25	3	6	3	128	6.48	2.74	3	11.122	0	CD59	High	High
-2311027039317610000	Q9Y3B8	1214059	858940.9	1214059	466421.6	9	2	3	2	237	6.87	2.01	2	5.349	0	REXO2	High	High
-903357879216	Q99729	13288689	9398792	13288689	5103727	25	9	18	8	332	8.21	32.07	9	37.263	0	HNRNPAB	High	High

6200000																		
-8804712893716090000	Q96S66	1778235	1257600	1778235	682901.3	7	4	7	4	551	5.55	1.95	4	14.16	0	CLCC1	High	High
-5706954355209200000	Q99436	9047932	6397383	9047932	3473903	20	5	10	5	277	7.68	11.34	5	15.985	0	PSMB7	High	High
8725302829576240000	P63151	9631369	6809725	9631369	3697813	13	5	16	5	447	6.2	17.28	5	18.91	0	PPP2R2A	High	High
6853752766827670000	P00846	3168177	2239957	3168177	1216340	4	1	4	1	226	10.1	9.01	1	4.792	0	ATP6	High	High
4473457085686510000	Q16186	9008186	6368469	9008186	3458203	10	4	8	4	407	5.07	7.64	4	10.999	0	ADRM1	High	High
-7983353042055500000	Q6SZW1	591283.9	417908.8	591283.9	226932.6	6	3	3	3	724	6.55	1.69	3	7.765	0	SARM1	High	Peak Found
-613397	P34932	74113484	52363955	74113484	28434646	40	29	57	27	840	5.19	66.44	29	110.677	0	HSPA4	High	High

371873318000																		
4733081690105890000	Q9UKN8	627112	443036.6	627112	240577.5	2	2	5	2	822	6.65	3.75	2	5.248	0	GTF3C4	High	High
2527179580342070000	Q9NUQ9	5045655	3563068	5045655	1934815	27	6	17	6	324	6.06	24.47	6	33.934	0	FAM49B	High	High
7590147556392160000	P07384	30640808	21636888	30640808	11749251	24	18	44	18	714	5.67	65.76	18	72.118	0	CAPN1	High	High
8611870974223820000	Q8NHP8	4401512	3107715	4401512	1687550	9	6	9	6	589	6.8	1.71	6	14.909	0	PLBD2	High	High
6575553270224250000	P55010	11860943	8371984	11860943	4546150	19	8	25	8	431	5.58	36.23	8	31.059	0	EIF5	High	High
7469427958651960000	Q9GZL7	2796786	1973427	2796786	1071609	12	4	6	4	423	5.9	3.99	4	12.646	0	WDR12	High	High
-5903683586191110000	Q13200	75795212	53432585	75795212	29014933	39	31	83	31	908	5.2	129.23	31	163.661	0	PSMD2	High	High

4306036841848920000	Q08752	5054455	3562371	5054455	1934437	16	7	14	7	370	7.21	14.24	7	21.802	0	PPID	High	High
1957491016862490000	P13073	18740236	13205893	18740236	7171057	34	6	11	6	169	9.51	16.83	6	21.328	0	COX4I1	High	High
6888826320597160000	Q16630	5864227	4131686	5864227	2243586	11	5	7	5	551	7.15	16.87	5	23.462	0	CPSF6	High	High
864018122010782000	P35080	20163336	14204415	20163336	7713274	34	5	20	5	140	6.99	22.35	5	25.578	0	PFN2	High	High
3930283768056640000	P31948	1.25E+08	88215512	1.25E+08	47902738	48	34	95	34	543	6.8	150.56	34	153.672	0	STIP1	High	High
-8295272483259110000	Q9Y333	365319.6	257346.6	365319.6	139744.2	28	2	3	2	95	6.52	2.28	2	8.152	0	LSM2	High	Peak Found
-8651766820472930000	Q12792	5751018	4049756	5751018	2199096	13	6	10	5	350	6.96	14.78	6	22.812	0	TWF1	High	High
228345059361	P41240	3605363	2538762	3605363	1378597	15	7	15	7	450	7.06	9.33	7	20.359	0	CSK	High	High

8710000																		
7857445100061980000	Q8WVY7	6690024	4709802	6690024	2557514	23	8	15	8	318	6.46	11.87	8	21.463	0	UBLCP1	High	High
2452511729080930000	O95159	3959039	2787046	3959039	1513420	16	4	6	4	310	8.07	3.87	4	11.621	0	ZFPL1	High	High
-2194368294587710000	Q16513	601288.1	423142.6	601288.1	229774.6	2	2	2	1	984	6.3	1.93	2	6.563	0	PKN2	High	Peak Found
-6985960340352960000	P62714	13312185	9365143	13312185	5085455	28	8	20	6	309	5.43	28.12	8	33.27	0	PPP2CB	High	High
2548334964560560000	Q9GZT8	744378.2	523633.4	744378.2	284343.1	3	1	7	1	377	6.65	7.49	1	3.097	0.003	NIF3L1	High	High
-4357852675593890000	P25205	12787861	8993399	12787861	4883591	19	13	29	13	808	5.77	24.94	13	42.32	0	MCM3	High	High
-316366779189	Q63ZY3	3190338	2243178	3190338	1218089	7	5	7	5	851	5.63	8.18	5	19.526	0	KANK2	High	High

5920000																		
513185524036398000	Q9NUQ3	746534.6	524875.5	746534.6	285017.6	3	2	2	2	528	7.52	1.83	2	4.713	0	TXLNG	High	High
3658516986896560000	P60903	18859322	13254074	18859322	7197220	37	4	18	4	97	7.37	28.31	4	16.795	0	S100A10	High	High
-5278147799990610000	P78318	478301	335972.3	478301	182439.5	3	1	1	1	339	5.38	2.27	1	4.293	0	IGBP1	High	Peak Found
6959373169283390000	Q6P587	630189.4	442658.3	630189.4	240372	3	1	1	1	224	7.39	0	1	2.103	0.01	FAHD1	High	Peak Found
-9208084483559460000	Q13423	4328161	3040120	4328161	1650844	9	9	17	9	1086	8.09	14.71	9	30.718	0	NNT	High	High
8809739148280000000	P46776	39187157	27524435	39187157	14946303	21	3	15	3	148	11	24.46	3	13.103	0	RPL27A	High	High
-5304133680658960000	Q16629	5708179	4006822	5708179	2175782	26	6	14	6	238	11.82	16.11	6	21.96	0	SRSF7	High	High

-3607998631011870000	Q9UBQ7	5087314	3570077	5087314	1938621	8	2	5	2	328	7.39	4.37	2	4.806	0	GRHPR	High	High
-5190108194104210000	O43615	4026052	2824434	4026052	1533723	12	5	12	5	452	8.32	20.7	5	21.778	0	TIMM44	High	High
8529586358305540000	P04181	32131414	22536347	32131414	12237675	30	12	52	12	439	7.03	61.75	12	68.875	0	OAT	High	High
-6265096555801140000	P06132	5534991	3881712	5534991	2107845	14	4	15	4	367	6.14	12.46	4	17.429	0	UROD	High	High
4573654140983490000	P12004	37185563	26073660	37185563	14158504	48	12	33	12	261	4.69	36.59	12	47.36	0	PCNA	High	High
4010508153477030000	Q14103	48492312	33970660	48492312	18446729	36	14	26	12	355	7.81	57.98	14	89.987	0	HNRNPD	High	High
1095568573092080000	Q16762	1545248	1082488	1545248	587812.3	12	3	6	3	297	7.25	1.95	3	7.584	0	TST	High	High

-8740985986820790000	P43304	13858786	9707485	13858786	5271353	28	21	34	21	727	7.69	28.28	21	61.539	0	GPD2	High	High
33731746096027900	Q8TC12	11382217	7971902	11382217	4328898	33	8	17	8	318	8.82	36.73	8	43.695	0	RDH11	High	High
3394918450034890000	P55884	34436457	24114377	34436457	13094576	24	18	49	18	814	5	61.18	18	78.295	0	EIF3B	High	High
-5359713146649650000	P31150	18845112	13195294	18845112	7165301	40	15	45	9	447	5.14	42.52	15	57.93	0	GDI1	High	High
6971879414843480000	Q9NRP0	2257978	1580987	2257978	858506.9	8	1	6	1	149	9.13	10.11	1	7.788	0	OSTC	High	High
7021380242978730000	Q92797	609818.3	426821.5	609818.3	231772.4	1	1	2	1	1274	6.13	1.95	1	2.471	0.007	SYMPK	High	Peak Found
-7705673583208630000	P04183	1896729	1327390	1896729	720798.5	19	3	6	3	234	8.51	1.72	3	10.816	0	TK1	High	High

6180366262275080000	P00505	36246739	25358832	36246739	13770339	26	13	47	13	430	9.01	85.05	13	48.948	0	GOT2	High	High
8375301801822560000	P36776	12293492	8597846	12293492	4668797	15	14	30	14	959	6.39	40.28	14	56.873	0	LONP1	High	High
6795582567929620000	P61916	3856518	2696823	3856518	1464427	19	3	5	3	151	7.65	12.52	3	9.467	0	NPC2	High	High
5288684708523740000	Q14240	5899033	4124462	5899033	2239663	38	14	70	5	407	5.48	118.73	14	89.636	0	EIF4A2	High	High
1302469806401560000	P49748	90209068	63067081	90209068	34246651	45	25	74	25	655	8.75	94.98	25	117.626	0	ACADVL	High	High
8013178931392780000	Q9H6V9	296232.1	207079.2	296232.1	112448	6	2	3	2	325	6.54	0	2	5.334	0	C2orf43; LDAH	High	Peak Found
3301543360737880000	Q9Y281	3482058	2434041	3482058	1321732	31	8	19	2	166	7.88	20.49	8	24.959	0	CFL2	High	High
4321322024900280000	Q9UDW1	402190.6	281122.9	402190.6	152655.2	27	1	1	1	63	9.47	2.62	1	8.645	0	UQCR10	High	Peak Found

7589685267009720000	Q9H0D6	5044600	3523963	5044600	1913581	8	5	7	5	950	7.47	7.61	5	17.193	0	XRN2	High	High
6373190460563600000	Q7L014	9530436	6655917	9530436	3614292	13	13	23	13	1031	9.29	20.23	13	42.761	0	DDX46	High	High
-3873794915793820000	Q9BYT8	8350543	5831664	8350543	3166707	18	11	18	11	704	6.64	17.05	11	40.831	0	NLN	High	High
-1308264510970570000	Q8WV24	2026857	1415133	2026857	768444.8	4	2	3	2	401	9.89	5.41	2	3.064	0.003	PHLDA1	High	High
545380139494389000	P08962	6917846	4829052	6917846	2622269	5	2	9	2	238	7.81	11.28	2	11.403	0	CD63	High	High
-3813014236257880000	P21291	1028618	717973.1	1028618	389873.3	20	2	4	2	193	8.57	2.81	2	10.902	0	CSRP1	High	High
-2746041825889840000	Q15059	1755154	1224993	1755154	665195	4	3	5	3	726	9.36	0	3	6.61	0	BRD3	High	High

-8021819990662320000	O43760	2170938	1514997	2170938	822672.7	8	2	6	2	224	4.94	3.47	2	5.908	0	SYNGR2	High	High
-7968684939462890000	O14974	1522404	1062294	1522404	576846.4	4	3	4	3	1030	5.4	1.99	3	8.575	0	PPP1R12A	High	High
-8651135355279390000	Q8N2G8	291939.8	203693.5	291939.8	110609.5	6	3	3	3	530	7.88	0	3	6.042	0	GHDC	High	High
8124726233717470000	P50750	615024.1	428992.4	615024.1	232951.2	7	3	5	2	372	8.79	3.52	3	9.109	0	CDK9	High	Peak Found
1365252663665010000	P49589	16431823	11456413	16431823	6221055	22	16	26	16	748	6.76	32.01	16	58.303	0	CARS	High	High
-8484028389043520000	P13010	84701777	59044746	84701777	32062445	33	24	70	24	732	5.81	106.84	24	118.414	0	XRCC5	High	High
-885056090803738000	P47897	39936205	27834881	39936205	15114882	40	27	82	27	775	7.15	100.64	27	113.157	0	QARS	High	High

-4853611610042410000	Q04760	15008990	10442520	15008990	5670491	32	6	15	6	184	5.31	10.46	6	17.375	0	GLO1	High	High
7463755477932390000	Q9H4M9	13753773	9564484	13753773	5193701	28	12	29	8	534	6.83	33.8	12	56.063	0	EHD1	High	High
2840679928359390000	Q6P1N0	184526.8	128189.6	184526.8	69609.42	1	1	2	1	951	8.09	0	1	3.155	0.003	CC2D1A	High	Peak Found
5897019344324900000	Q9NP79	2536369	1761783	2536369	956682.3	9	3	5	3	307	6.29	6.3	3	8.792	0	VTA1	High	High
-2982172693089120000	P28066	27102091	18823304	27102091	10221420	44	8	16	8	241	4.79	27.95	8	41.624	0	PSMA5	High	High
-5615919369887990000	P49903	611988.5	424980.7	611988.5	230772.8	10	3	4	3	392	5.97	1.92	3	10.853	0	SEPHS1	High	Peak Found
-8817189782038060000	P52943	728721.6	505710.4	728721.6	274610.6	9	1	1	1	208	8.72	2.9	1	6.301	0	CRIP2	Peak Found	High

-4534936647316470000	Q49AN0	728491.2	505448.8	728491.2	274468.5	2	1	1	1	593	8.35	0	1	2.11	0.01	CRY2	Peak Found	High
-2034922387344060000	P12955	8581689	5952972	8581689	3232579	17	7	14	7	493	6	12.27	7	23.601	0	PEPD	High	High
8990859399562710000	Q10567	12354383	8558259	12354383	4647301	21	21	56	9	949	5.06	48.78	21	75.222	0	AP1B1	High	High
120260569084323000	Q92626	1831642	1268662	1831642	688908.1	3	4	8	4	1479	7.17	1.76	4	12.618	0	PXDN	High	High
-4669719094448440000	Q9BSJ8	15325699	10606105	15325699	5759321	19	17	34	17	1104	5.83	35.73	17	58.039	0	ESYT1	High	High
576551174696518000	Q5T447	1897830	1313032	1897830	713002	4	3	4	3	861	5.64	1.84	3	7.418	0	HECTD3	High	High
5286637805257650000	Q9BQG0	20503788	14171872	20503788	7695602	19	24	46	24	1328	9.28	39.65	24	92.759	0	MYBBP1A	High	High

-2934429115890700000	P49821	2116479	1461967	2116479	793876.5	12	6	8	6	464	8.21	5.2	6	15.017	0	NDUFV1	High	High
5080369595642030000	Q9BVG4	242104.1	167069.5	242104.1	90722.01	5	1	2	1	233	4.79	0	1	2.263	0.009	CXorf26; PBDC1	High	High
5014851106120830000	Q96G23	3037638	2095911	3037638	1138120	7	2	6	2	380	8.98	2.39	2	7.744	0	CERS2	High	High
-3819481250379260000	Q5JWF2	4224484	2914753	4224484	1582768	6	6	13	5	1037	5.03	17.59	6	21.55	0	GNAS	High	High
2832146552711630000	Q02218	9886497	6819498	9886497	3703120	20	16	24	10	1023	6.86	23.59	16	48.58	0	OGDH	High	High
2683396977362810000	P17931	20137329	13881416	20137329	7537879	34	8	32	8	250	8.56	27.17	8	32.749	0	LGALS3	High	High
-2154176655508350000	Q13642	11869380	8175015	11869380	4439192	19	6	16	6	323	8.97	14.63	6	27.787	0	FHL1	High	High

7446725702153580000	Q9Y2T2	2594394	1786820	2594394	970278	11	4	7	3	418	6.93	7.29	4	12.125	0	AP3M1	High	High
4039607230823650000	P42025	3708164	2553758	3708164	1386740	28	7	13	2	376	6.4	15.44	7	28.117	0	ACTR1B	High	High
8037503062771540000	Q8IXI2	1278780	880397.3	1278780	478072.9	5	3	6	2	618	6.27	2.18	3	10.099	0	RHOT1	High	High
-5469574299663460000	P11233	6225415	4285781	6225415	2327262	28	5	16	3	206	7.11	12.88	5	16.249	0	RALA	High	High
-796628633734857000	Q13439	886069.3	609871	886069.3	331171.8	2	4	6	4	2230	5.39	3.39	4	12.034	0	GOLGA4	High	High
3460049962588220000	Q96CX2	2026776	1394892	2026776	757453.7	19	5	8	5	325	5.64	2.01	5	13.552	0	KCTD12	High	High
5154209306994620000	Q14938	715288.6	492244.1	715288.6	267298.1	3	1	1	1	502	8.68	0	1	2.301	0.008	NFIX	High	Peak Found
-695024727111	Q9H3N1	9291082	6392079	9291082	3471023	14	4	12	4	280	4.98	12.26	4	13.396	0	TMX1	High	High

2540000																		
-8579706144891720000	O00267	1482909	1020187	1482909	553981.3	5	4	6	4	1087	5.06	8.99	4	17.813	0	SUPT5H	High	High
2420499355867480000	Q96SQ9	1280346	880695.4	1280346	478234.8	9	4	5	4	504	8.62	4.54	4	11.734	0	CYP2S1	High	High
4552444705912860000	Q14160	4031592	2773095	4031592	1505845	6	9	11	9	1630	5.07	5.19	9	22.751	0	SCRIB	High	High
-2163956888383820000	Q96A65	1143574	786356	1143574	427006.6	3	3	6	3	974	6.49	5.24	3	11.03	0	EXOC4	High	High
1767684544015940000	P14678	14710120	10114699	14710120	5492478	15	4	7	4	240	11.19	8.19	4	13.151	0	SNRPB	High	High
4996428185622590000	Q9NZB2	7499958	5154922	7499958	2799223	13	12	20	12	1118	8.88	20.53	12	40.145	0	FAM120A	High	High
-348064151225	Q9NRF8	476583.5	327534	476583.5	177857.3	8	4	7	2	586	6.9	10.43	4	14.48	0	CTPS2	High	High

2910000																		
8595633984951090000	Q52LW3	1060607	728488.2	1060607	395583.3	2	3	5	3	1261	6.74	3.25	3	8.177	0	ARHGAP29	High	High
8211407093643670000	Q8NDZ4	485288.3	333228.5	485288.3	180949.5	8	3	4	3	430	8.53	3.99	3	10.943	0	C3orf58	High	High
-109273256828227000	Q07866	6742422	4629311	6742422	2513806	19	10	20	7	573	6.2	22.58	10	36.568	0	KLC1	High	High
-3997402182379010000	O15371	10428725	7159334	10428725	3887658	30	11	23	11	548	6.05	18.28	11	42.318	0	EIF3D	High	High
-9137773696009280000	P58546	2896767	1987310	2896767	1079148	38	4	7	4	118	5.52	17.79	4	30.105	0	LUZP6; MTPN	High	High
-366099160936262000	Q9NQG5	2587450	1774952	2587450	963833.5	17	5	8	5	326	5.97	5.14	5	14.245	0	RPRD1B	High	High
7061207721996520000	P08758	3.7E+08	2.54E+08	3.7E+08	1.38E+08	56	22	106	21	320	5.05	123.98	22	92.721	0	ANXA5	High	High

5413684009215810000	O15143	24647646	16897113	24647646	9175461	35	10	25	10	372	8.35	41.6	10	43.914	0	ARPC1B	High	High
-5062141414675450000	Q9P0L0	17941275	12297609	17941275	6677841	41	9	28	8	249	8.62	47.3	9	32.572	0	VAPA	High	High
3643630707960140000	Q4KWH8	3859739	2645451	3859739	1436531	0	1	10	1	1693	7.74	3.55	1	3.416	0.002	PLCH1	High	High
7866325673474560000	Q14241	222539.5	152497.2	222539.5	82808.96	1	1	1	1	798	9.57	0	1	2.26	0.009	TCEB3; ELOA	High	Peak Found
-3464884523288300000	Q12972	553227.5	378890.2	553227.5	205744.7	4	2	2	2	351	7.37	0	2	7.429	0	PPP1R8	High	Peak Found
2100878916131080000	P36871	67542127	46255538	67542127	25117656	51	28	74	28	562	6.76	107.26	28	118.143	0	PGM1	High	High
-7798556211851200000	Q96AX1	215959.1	147887.5	215959.1	80305.77	1	1	1	1	596	6.96	0	1	2.231	0.009	VPS33A	High	Peak Found

6184092773991490000	P39748	10169164	6963404	10169164	3781264	20	6	11	6	380	8.62	13.01	6	21.746	0	FEN1	High	High
8991968867262950000	Q9P2K8	171201.5	117198.2	171201.5	63640.91	1	2	2	2	1649	6.28	1.7	2	5.934	0	EIF2AK4	High	High
1978965257736530000	Q9NYK5	1147632	785499.3	1147632	426541.4	6	2	2	2	338	7.65	1.65	2	6.059	0	MRPL39	High	High
2337219862284100000	Q9Y262	30311669	20736459	30311669	11260300	39	20	51	20	564	6.34	55.41	20	73.934	0	EIF3L	High	High
1479790111935500000	Q9P258	22596072	15452781	22596072	8391161	31	14	35	14	522	8.78	39.62	14	58.587	0	RCC2	High	High
4819248738273410000	O14981	309506.2	211557	309506.2	114879.5	1	2	2	2	1849	6.52	1.68	2	4.998	0	BTAF1	High	Peak Found
-6270565792586480000	Q9UHY7	792337.5	541546.1	792337.5	294070.1	8	2	2	2	261	4.78	0	2	3.919	0	ENOPH1	High	Peak Found
792913260090	Q53GL7	762905.1	521410.6	762905.1	283136.1	4	3	4	3	10	4.97	0	3	7.865	0	PARP10	High	High

0670000										25								
2666399162137350000	P38117	32373368	22089131	32373368	11994827	47	13	40	13	255	8.1	45.19	13	54.832	0	ETFB	High	High
743722838785437000	Q12846	1135673	774531.9	1135673	420585.9	7	2	3	2	297	6.28	1.72	2	6.157	0	STX4	High	Peak Found
-9003778122824520000	Q9BRT3	416883.3	284293.9	416883.3	154377.1	10	1	1	1	115	4.37	1.79	1	3.398	0.002	MIEN1	High	Peak Found
5891546535660200000	Q6P1X6	1038356	707921.6	1038356	384415.2	10	2	3	2	216	9.14	4.74	2	6.843	0	C8orf82	High	High
-6145009283245850000	P62487	270687.8	184523.9	270687.8	100200	5	1	1	1	172	5.54	0	1	2.535	0.006	POLR2G	High	Peak Found
7944368975479600000	O94979	34459912	23489505	34459912	12755258	15	18	46	18	1220	6.89	59.49	18	66.777	0	SEC31A	High	High
3107110472416440000	P30536	11874878	8091479	11874878	4393831	9	2	6	2	169	9.36	9.62	2	4.383	0	TSPO	High	High

-3306595998206250000	P62753	33050920	22519614	33050920	12228588	29	9	33	9	249	10.84	44.53	9	47.149	0	RPS6	High	High
-5808192534094490000	Q9HC35	3414532	2326375	3414532	1263267	9	8	12	8	981	6.4	5.82	8	22.736	0	EML4	High	High
-9144815558027080000	Q96FV9	1227047	835650.5	1227047	453774.5	7	5	5	5	657	4.98	2.08	5	12.689	0	THOC1	High	Peak Found
-4770714879888360000	Q5GLZ8	10522257	7165379	10522257	3890940	18	15	24	15	1057	6.19	12.41	15	45.982	0	HERC4	High	High
-4383082791475620000	Q9NYP7	891340.6	606926.1	891340.6	329572.7	3	1	3	1	299	9.42	1.68	1	3.517	0.002	ELOVL5	High	High
8545908257350260000	O15144	46210831	31461820	46210831	17084380	36	13	49	13	300	7.36	69.83	13	75.6	0	ARPC2	High	High
513337833647549000	P11717	5358278	3646877	5358278	1980325	4	9	17	9	24	5.94	10.4	9	32.104	0	IGF2R	High	High

										91								
-7048585715635480000	Q13885	10676846	7265125	10676846	3945104	47	20	219	1	445	4.89	392.38	20	137.23	0	TUBB2A	High	High
-1080022240978710000	P49721	4741698	3226085	4741698	1751827	22	5	14	5	201	7.02	15.64	5	16.813	0	PSMB2	High	High
777273144622755000	P51649	382938.6	260472.3	382938.6	141441.5	1	1	1	1	535	8.28	0	1	2.449	0.007	ALDH5A1	High	Peak Found
4757838979120080000	P17405	291228.2	198076.9	291228.2	107559.6	2	1	3	1	631	7.28	1.61	1	2.167	0.009		High	High
-7605118544862910000	Q9H0U3	6547501	4451887	6547501	2417461	14	6	12	6	335	9.63	9.25	6	17.023	0	MAGT1	High	High
-2146106311129580000	P35244	2018740	1372580	2018740	745337.7	21	2	4	2	121	5.08	6.52	2	8.533	0	RPA3	High	High
-853110493720	Q9P003	2725643	1852954	2725643	1006190	14	1	2	1	139	6.65	5.09	1	8.967	0	CNIH4	High	High

1260000																		
3073317532419450000	Q07954	7126017	4843642	7126017	2630192	5	19	31	19	4544	5.39	23.57	19	66.987	0	LRP1	High	High
6028966564330730000	Q6PI78	2116212	1437448	2116212	780562.4	9	2	4	2	240	8.6	4.23	2	7.289	0	TMEM65	High	High
-6810289511595070000	Q15758	14276310	9689626	14276310	5261655	16	9	20	9	541	5.48	19.27	9	40.867	0	SLC1A5	High	High
2271404358644780000	P46778	21199640	14386805	21199640	7812315	21	3	12	3	160	10.49	19.42	3	18.18	0	RPL21	High	High
-4905591351439670000	P50995	22806829	15476999	22806829	8404311	34	17	49	17	505	7.65	61.72	17	65.53	0	ANXA11	High	High
3450396974392380000	Q13112	229460.4	155624.2	229460.4	84506.96	3	1	1	1	559	7.5	0	1	2.816	0.004	CHAF1B	High	Peak Found
3208600518816650000	Q96IU4	1601820	1086284	1601820	589873.3	22	3	5	3	210	6.4	4.36	3	13.359	0	ABHD14B	High	High

-3039044237151120000	P61163	9920951	6727506	9920951	3653167	30	8	14	3	376	6.64	17.33	8	35.039	0	ACTR1A	High	High
-4642176776796330000	P35580	9259958	6277440	9259958	3408772	16	30	93	11	1976	5.54	144.31	30	160.199	0	MYH10	High	High
1400843138587650000	P98175	350507.4	237610.3	350507.4	129027	1	1	1	1	930	5.97	0	1	2.954	0.004	RBM10	Peak Found	High
7011076575076350000	O00442	1189230	805807.2	1189230	437569	3	1	4	1	366	7.85	1.78	1	3.206	0.002	RTCA	High	High
9133260577685850000	O00410	24982269	16922155	24982269	9189059	26	25	51	24	1097	4.94	50.37	25	93.098	0	IPO5	High	High
-8034118436781940000	Q9BT22	399848.2	270783	399848.2	147040.4	5	2	3	2	464	7.23	3.47	2	5.495	0	ALG1	High	High
-7145479494712380000	P80217	1019128	689869	1019128	374612.3	10	3	5	3	286	6.09	8.34	3	8.681	0	IFI35	High	High

-3405603430523790000	P52888	11876130	8024233	11876130	4357314	17	12	20	12	689	6.05	19.58	12	39.057	0	THOP1	High	High
8114162095880210000	Q9H3S7	1471249	993420.9	1471249	539446.8	4	4	4	4	1636	6.92	3.83	4	14.811	0	PTPN23	High	High
2214005141303330000	P12956	84853717	57264929	84853717	31095970	38	22	67	22	609	6.64	116.46	22	112.394	0	XRCC6	High	High
1683899515612250000	O94776	8159010	5506009	8159010	2989870	12	7	13	6	668	9.66	13.1	7	24.29	0	MTA2	High	High
3562387644317080000	Q8N3C0	2782455	1876883	2782455	1019184	4	8	11	8	2202	7.09	7.41	8	22.049	0	ASCC3	High	High
-7115142250704830000	P08670	49460017	33343864	49460017	18106366	55	29	60	28	466	5.12	71.06	29	118.792	0	VIM	High	High
275596885495269000	Q14166	3442960	2320967	3442960	1260330	9	5	13	5	644	5.53	8.76	5	18.237	0	TTLL12	High	High
-666453734404	Q08209	14275794	9617362	14275794	5222415	19	10	19	10	521	5.86	20.31	10	31.503	0	PPP3CA	High	High

7950000																		
7460100142715470000	P09960	7238819	4876435	7238819	2647999	15	8	19	8	611	6.18	17.75	8	37.06	0	LTA4H	High	High
-8277375164580500000	Q14683	7524883	5068722	7524883	2752415	13	14	21	14	1233	7.64	19.7	14	38.878	0	SMC1A	High	High
5541233720560710000	O43809	5690238	3832067	5690238	2080887	26	6	10	6	227	8.82	18.31	6	30.744	0	NUDT21	High	High
-8527092773428760000	Q13561	9684344	6517367	9684344	3539057	30	10	23	10	401	5.21	32.81	10	34.375	0	DCTN2	High	High
3018434530730780000	Q9BUN8	4307481	2898424	4307481	1573900	8	2	6	2	251	9.51	0	2	5.38	0	DERL1	High	High
2616426161905970000	Q9UG63	2095143	1409711	2095143	765500.3	5	3	9	3	623	7.37	11.06	3	11.931	0	ABCF2	High	High
1385876623079040000	Q9UFC0	247130	166278.1	247130	90292.23	2	1	1	1	647	7.21	0	1	4.714	0	LRWD1	High	Peak Found

-5747732458404130000	Q9HBH5	290576.2	195501.2	290576.2	106161	2	1	1	1	336	8.79	1.6	1	2.724	0.005	RDH14	High	Peak Found
6534553132144550000	Q09028	5772696	3879531	5772696	2106661	27	10	24	4	425	4.89	40.84	10	41.767	0	RBBP4	High	High
-2836831182499160000	O00148	51802431	34805736	51802431	18900191	29	14	32	5	427	5.68	40.76	14	48.666	0	DDX39A	High	High
961314597478386000	P57740	1020040	685262.7	1020040	372111	6	4	4	4	925	5.43	4.14	4	14.257	0	NUP107	High	High
3652435183447380000	Q9H2D1	428855.8	287928	428855.8	156350.5	4	1	1	1	315	9.45	2.1	1	3.746	0.001	SLC25A32	High	Peak Found
-1822975009015350000	P60228	43859358	29433438	43859358	15982929	29	12	44	12	445	6.04	40.92	12	41.054	0	EIF3E	High	High
3559430331434450000	O95630	489482.1	328354.6	489482.1	178302.9	6	2	2	2	424	6.29	2.04	2	5.563	0	STAMBP	High	Peak Found

4135876604710410000	P49593	163741.1	109811.8	163741.1	59629.95	8	3	3	3	454	5.1	2.3	3	11.702	0	PPM1F	High	Peak Found
-8237731062455560000	P40763	3977546	2666795	3977546	1448121	7	5	11	5	770	6.3	4.65	5	17.449	0	STAT3	High	High
3559425132157330000	P16152	70903275	47535678	70903275	25812797	65	14	52	10	277	8.32	70.35	14	75.525	0	CBR1; SETD4	High	High
8166763961734870000	P54727	12881978	8630128	12881978	4686327	19	8	15	6	409	4.84	13.57	8	26.163	0	RAD23B	High	High
8906999809398880000	Q9BQ95	307679.8	206107.8	307679.8	111920.5	6	1	1	1	431	6.29	0	1	3.019	0.003	ECSIT	High	Peak Found
1353658345851560000	Q9BR76	13833508	9265236	13833508	5031203	17	8	20	8	489	5.88	17.38	8	28.364	0	CORO1B	High	High
1677605172328810000	Q6IAA8	1850210	1237336	1850210	671897.3	30	3	5	3	161	5.15	2.22	3	11.592	0	LAMTOR1	High	High
-552607950692	O14617	7817951	5226432	7817951	2838054	9	9	17	9	11	8.48	16.98	9	37.731	0	AP3D1	High	High

9440000										53								
550691139419414000	O00194	483258.9	323061.9	483258.9	175428.9	5	1	1	1	218	5.52	0	1	2.274	0.008	RAB27B	Peak Found	High
-4885390381985600000	P08842	1062167	709421.9	1062167	385229.9	3	2	3	2	583	7.66	4.4	2	5.677	0	STS	High	Peak Found
-6720248987179000000	O60313	9138254	6102354	9138254	3313697	19	14	28	14	960	7.87	17.54	14	50.134	0	OPA1	High	High
-4187498386560220000	P29317	1422210	949160.2	1422210	515412.5	4	3	6	3	976	6.23	1.68	3	8.783	0	EPHA2	High	High
6313231503744560000	O00754	2002036	1336078	2002036	725516.1	5	4	4	4	1011	7.28	4.7	4	13.996	0	MAN2B1	High	High
2826650301699750000	Q9HCU5	158709.9	105914.7	158709.9	57513.75	4	1	1	1	417	7.88	0	1	2.28	0.008	PREB	High	Peak Found
-782452157780	Q53GS9	5402938	3604372	5402938	1957244	15	7	8	7	565	8.91	3.56	7	18.776	0	USP39	High	High

4770000																		
1789313073450950000	Q9BSR8	1067649	712144.9	1067649	386708.5	7	2	2	2	244	4.65	3.64	2	5.332	0	YIPF4	High	Peak Found
1141032543915840000	Q13011	36932906	24623157	36932906	13370853	30	10	37	10	328	8	61.8	10	55.556	0	ECH1	High	High
4822956659812590000	P17706	5737023	3824809	5737023	2076946	15	5	7	5	415	8.29	2.17	5	15.332	0	PTPN2	High	High
144982604897555000	P52306	6665737	4443836	6665737	2413090	22	11	26	11	607	5.31	28.71	11	47.723	0	RAP1GDS1	High	High
-3028691531671680000	Q14697	1.95E+08	1.3E+08	1.95E+08	70751531	35	35	153	35	944	6.14	243.65	35	226.544	0	GANAB	High	High
5626279923656500000	P25325	3067301	2044203	3067301	1110042	22	5	10	5	297	6.6	4.96	5	24.375	0	MPST	High	High
6713486079386730000	P50454	1.34E+08	89507587	1.34E+08	48604360	39	15	64	15	418	8.69	137.9	15	108.703	0	SERPINH1	High	High
-915816	O14980	25447829	16949049	25447829	9203663	23	21	55	21	10	6.06	58.27	21	96.931	0	XPO1	High	High

600694735000										71								
3944775931209300000	P60660	1.29E+08	85941794	1.29E+08	46668065	52	8	44	8	151	4.65	82.33	8	51.226	0	MYL6	High	High
-4939366548436880000	Q7L0Y3	1449316	964960.9	1449316	523992.6	9	4	5	4	403	9.36	3.32	4	8.135	0	TRMT10C	High	High
-728373557133263000	Q12797	1E+08	66810309	1E+08	36279297	34	28	88	28	758	5.01	116.94	28	112.872	0	ASPH	High	High
1339372435308810000	Q8IWT0	222655.1	148096.6	222655.1	80419.32	6	1	2	1	167	4.49	1.76	1	5.088	0	ZBTB8OS	High	High
-4517606527837170000	Q9Y5P6	3806716	2531493	3806716	1374650	21	6	9	6	360	6.61	10.4	6	21.113	0	GMPPB	High	High
7625852785660980000	Q15126	3308797	2198741	3308797	1193959	31	6	10	6	192	5.73	7.7	6	19.163	0	PMVK	High	High
4502753596309460000	Q6NUQ4	16104708	10701539	16104708	5811144	21	13	26	13	689	9.14	32.22	13	51.863	0	TMEM214	High	High

-1974674273488480000	Q5RI15	637097.3	423236.1	637097.3	229825.4	22	2	6	2	118	8.76	7.49	2	10.522	0	COX20	High	High		
7690147936594120000	P08397	1461312	970682.4	1461312	527099.4	5	2	5	2	361	7.18	3.37	2	5.638	0	HMBS	High	High		
1168885672839200000	Q9HC52	404101.3	268318.7	404101.3	145702.3	2	1	1	1	389	9.91	0	1	2.609	0.006	CBX8	High	Peak Found		
-3128972635678320000	Q9BUT1	1531999	1017214	1531999	552367.1	13	3	4	3	245	7.65	3.73	3	9.55	0	BDH2	High	High		
-5012764256203320000	P07942	4708256	3125729	4708256	1697332	5	9	11	9	1786	4.94	13.2	9	26.704	0	LAMB1	High	High		
4837224883862520000	Q9BYD1	1126615	747706.8	1126615	406019.4	19	3	5	3	178	9.16	0	3	7.475	0	MRPL13	High	High		
9022416781062350000	Q9UPU5	3436571	2280165	3436571	1238174	4	9	20	9	2620	6.14	5.76	9	27.094	0	USP24	High	High		

6062365738304160000	Q9BXY0	1011036	670665.8	1011036	364184.6	10	3	6	3	300	5.38	4.17	3	8.787	0	MAK16	High	High
-220696745626841000	O43293	454114.2	301101.9	454114.2	163504.2	3	1	1	1	454	6.89	0	1	3.522	0.002	DAPK3	Peak Found	High
516954531369469000	P60900	34672498	22975524	34672498	12476156	46	10	33	10	246	6.76	51.97	10	49.011	0	PSMA6	High	High
3453678624484120000	Q8WUM4	47144246	31215107	47144246	16950410	42	37	90	37	868	6.52	94.42	37	139.797	0	PDCD6IP	High	High
-7289415820017290000	P82675	541979.9	358311.9	541979.9	194570.3	6	2	2	2	430	9.92	1.89	2	4.603	0	MRPS5	High	Peak Found
6438294564185070000	Q9BPW8	15070760	9961390	15070760	5409229	23	9	27	7	284	9.31	27.1	9	29.519	0	NIPSNAP1	High	High
-1787588940823920000	P61224	25729559	16997239	25729559	9229831	35	7	22	2	184	5.78	35.1	7	31.294	0	RAP1B	High	High
217003866787	O15027	6699621	4424814	6699621	24022760	1	1	1	1	23	5.8	0	1	2.399	0.008		High	Peak Found

3590000										57								
1560013588739460000	Q9H7D7	990607.8	654016.4	990607.8	355143.6	3	2	4	2	661	6.16	1.85	2	5.968	0	WDR26	High	High
-3705631994362760000	P11413	1.8E+08	1.19E+08	1.8E+08	64410873	52	29	92	29	515	6.84	131.61	29	157.085	0	G6PD	High	High
5966605329502240000	P10599	40364676	26644157	40364676	14468295	41	4	21	4	105	4.92	33.61	4	28.415	0	TXN	High	High
-1832410475537090000	P11047	2419083	1596501	2419083	866931	4	5	7	5	1609	5.12	5.43	5	19.724	0	LAMC1	High	High
8862135404577210000	Q9UJZ1	1872147	1235497	1872147	670898.9	9	2	3	2	356	7.39	3.66	2	12.878	0	STOML2	High	Peak Found
-4942949843641680000	Q9NQ66	342099.8	225642.8	342099.8	122528.4	1	1	1	1	1216	6.23	0	1	3.356	0.002	PLCB1	Peak Found	High
823100698756	P18084	3000174	1978637	3000174	1074438	8	7	9	7	799	6.06	3.89	7	14.661	0	ITGB5	High	High

3480000																		
7707178006407490000	Q9UM47	271662.3	179094.3	271662.3	97251.68	0	1	1	1	2321	5.39	0	1	3.383	0.002	NOTCH3	High	Peak Found
-3377056009907550000	O14745	3455932	2277451	3455932	1236700	19	5	13	5	358	5.77	12.75	5	17.644	0	SLC9A3R1	High	High
9072204051848650000	O75832	1624102	1070077	1624102	581072.5	29	4	10	4	226	6.1	10.52	4	14.991	0	PSMD10	High	High
5166856508772240000	P01111	6048631	3984891	6048631	2163873	23	4	6	1	189	5.17	12.05	4	15.255	0	NRAS	High	High
-4871053517443560000	P57772	1010992	665991.6	1010992	361646.4	7	4	6	4	596	8.35	4.77	4	19.068	0	EEFSEC	High	High
-3876256361286900000	P62269	1.23E+08	81147849	1.23E+08	44064859	46	13	53	13	152	10.99	81.65	13	65.924	0	RPS18	High	High
-832180465232	Q9BXJ9	9070886	5969847	9070886	3241743	19	16	25	16	866	7.42	19.64	16	48.736	0	NAA15	High	High

9170000																		
-6012063852270540000	Q9H8M9	456013.2	299988.9	456013.2	162899.8	7	1	1	1	152	6.93	1.8	1	2.994	0.003	EVA1A	High	Peak Found
-8220013005457950000	Q15424	4990157	3280353	4990157	1781295	13	8	13	8	915	5.47	15.47	8	30.266	0	SAFB	High	High
621836974049986000	Q9Y3E7	1190513	782308.9	1190513	424808.9	8	2	4	2	222	5.12	0	2	4.85	0	CHMP3; RNF103-CHMP3	High	High
8927503928993300000	O00423	6281926	4127213	6281926	2241157	12	8	15	8	815	7.06	16.67	8	27.924	0	EML1	High	High
6704752873658690000	P37268	32383838	21258120	32383838	11543573	41	14	36	14	417	6.54	42.21	14	60.959	0	FDFT1	High	High
-5845152794455650000	O75179	1859063	1220362	1859063	662680.5	2	6	6	6	2603	6.52	0	6	11.715	0	ANKRD17	High	High
7662049170687480000	Q9Y376	2515369	1650164	2515369	896071.1	13	5	8	5	341	6.89	7.05	5	13.842	0	CAB39	High	High

-1079834079423300000	Q8TD19	1710122	1121328	1710122	608902.6	5	4	5	4	979	5.74	4.3	4	13.245	0	NEK9	High	Peak Found
3145308466496440000	O43292	2613953	1713789	2613953	930620.5	5	3	4	3	621	8.06	4.08	3	9.983	0	GPAA1	High	High
8070749143709810000	Q14195	1.07E+08	70219978	1.07E+08	38130813	43	21	66	20	570	6.49	108.25	21	115.441	0	DPYSL3	High	High
1199984255511240000	P85037	984938.4	645442.5	984938.4	350487.8	4	3	3	3	733	9.32	0	3	5.455	0	FOXK1	High	Peak Found
2524487954234530000	Q9Y6N7	168850.7	110628.5	168850.7	60073.42	1	1	1	1	1651	6.04	1.66	1	2.75	0.004	ROBO1	High	Peak Found
8048199414682780000	Q06323	28370579	18583646	28370579	10091281	47	12	40	12	249	6.02	30.66	12	47.028	0	PSME1	High	High
8218333417811030000	Q9H299	25441504	16663927	25441504	9048837	32	4	6	4	93	4.93	8.37	4	10.492	0	SH3BGRL3	High	High
140007278281	P62424	21383652	14004359	21383652	7604639	44	10	32	10	266	10.61	46.54	10	45.213	0	RPL7A	High	High

3280000																		
2670140620025600000	Q6S8J3	35076067	22968346	35076067	12472259	10	10	132	1	1075	6.2	197.35	10	88.758	0	POTEE	High	High
-1352370190722130000	O75746	176507.5	115545.9	176507.5	62743.66	4	3	4	1	678	8.38	1.89	3	6.471	0	SLC25A12	High	Peak Found
-4643868144451560000	P41567	883601.1	578340.1	883601.1	314049.9	28	2	6	2	113	7.44	4.88	2	7.92	0	EIF1	High	High
-8211975543562730000	P13984	640498.1	419211.7	640498.1	227640.1	12	2	2	2	249	9.23	0	2	7.116	0	GTF2F2	High	Peak Found
-5604627173476330000	O75534	18291401	11964288	18291401	6496841	20	17	40	17	798	6.25	47.67	17	54.456	0	CSDE1	High	High
5964794776079930000	P56537	4112257	2689743	4112257	1460583	23	4	11	4	245	4.68	17.62	4	21.229	0	EIF6	High	High

1987024820899370000	Q9BRA2	9504566	6210228	9504566	3372275	27	3	13	3	123	5.52	21.35	3	13.992	0	TXNDC17	High	High
7598274604461760000	P49750	513955.5	335755.6	513955.5	182321.8	1	2	3	2	2146	6.55	1.95	2	5.698	0		High	High
7462216625703250000	O43847	7598027	4963092	7598027	2695055	8	8	19	8	1151	4.98	18.25	8	31.486	0		High	High
-8882666347281850000	P48739	11363767	7419162	11363767	4028749	32	9	25	8	271	6.87	33.4	9	34.961	0	PITPNB	High	High
8115328096255320000	P29966	5370277	3505166	5370277	1903374	23	5	9	5	332	4.45	15.14	5	26.513	0	MARCKS	High	High
7523949865199720000	P50579	4137531	2699102	4137531	1465665	7	2	4	2	478	5.82	3.62	2	6.776	0	METAP2	High	Peak Found
3099345167640790000	Q00796	6197696	4041912	6197696	2194837	25	8	15	8	357	7.97	18.64	8	21.41	0	SORD	High	High
-649048403866	Q13283	13871145	9043492	13871145	4910792	23	7	21	7	466	5.52	16.85	7	41.06	0	G3BP1	High	High

4760000																		
5146194763557840000	Q9Y613	558236.3	363902.7	558236.3	197606.2	2	2	2	2	1164	6.39	0	2	4.356	0	FHOD1	High	Peak Found
117262061430317000	Q96T88	663583.9	431783.7	663583.9	234466.9	2	2	4	2	793	7.56	3.88	2	5.362	0	UHRF1	High	High
6140952741663270000	Q9H3U5	687058.4	446667.3	687058.4	242549	3	1	1	1	465	6.74	1.75	1	4.192	0	MFSD1	Peak Found	High
6718063858029520000	P50395	1.17E+08	75975279	1.17E+08	41256053	58	24	75	18	445	6.47	91.42	24	106.633	0	GDI2	High	High
-4248741172720890000	P62304	7082457	4603868	7082457	2499990	25	2	6	2	92	9.44	4.48	2	6.291	0	SNRPE	High	High
-7586099756274290000	Q71UM5	612649.6	398059.5	612649.6	216154.1	25	2	3	1	84	9.45	1.9	2	5.698	0	RPS27L	High	Peak Found
4284649004718390000	Q68CQ7	252046.9	163708.9	252046.9	88897.12	4	1	1	1	371	9.35	0	1	3.891	0	GLT8D1	High	Peak Found

4921032794514210000	Q8WXX5	302424.8	196391	302424.8	106644.2	15	3	3	3	260	5.73	0	3	5.885	0	DNAJC9	High	High
3783621162172780000	P61225	1853363	1203279	1853363	653404.1	18	3	7	3	183	4.81	6.43	3	9.403	0	RAP2B	High	High
8244788051015200000	O43447	2868954	1862350	2868954	1011292	17	3	3	3	177	8.07	2.52	3	7.971	0	PPIH	High	High
-7210504404319700000	P33240	463209	300508	463209	163181.7	9	3	4	3	577	6.83	3.81	3	7.095	0	CSTF2	High	High
7003283534467840000	P40937	4243925	2752326	4243925	1494566	14	5	8	5	340	7.2	4.05	5	12.715	0	RFC5	High	High
1799847195195980000	Q5VYK3	7592979	4921041	7592979	2672221	7	13	16	13	1845	7.12	13.69	13	34.228	0	KIAA0368	High	High
-2992609788783460000	O60831	2488393	1612389	2488393	875558.4	12	2	4	2	178	9.19	9	2	6.931	0	PRAF2	High	High

3353104297975980000	O43242	31278931	20261496	31278931	11002386	43	20	53	20	534	8.44	72.27	20	108.747	0	PSMD3	High	High
1134562392053290000	Q96ER9	708521.2	458953.8	708521.2	249220.9	8	2	4	2	411	8.19	5.39	2	9.279	0	CCDC51	High	High
-3338411764392510000	P45954	1848084	1196958	1848084	649971.5	6	3	5	3	432	6.99	4.09	3	6.923	0	ACADSB	High	High
152076562272560000	Q13409	8719944	5647329	8719944	3066609	13	8	15	8	638	5.2	24.35	8	34.295	0	DYNC1I2	High	High
-7730919708393100000	P02765	2173158	1407190	2173158	764131.6	5	2	3	2	367	5.72	5.93	2	5.103	0		High	High
9167307694424550000	O95336	14046386	9090628	14046386	4936388	49	10	26	10	258	6.05	22.67	10	43.261	0	PGLS	High	High
-3137976061761610000	Q16512	92512.45	59865.34	92512.45	32508.04	2	2	2	1	942	6.37	0	2	4.704	0	PKN1	High	Peak Found

-859474664897497000	Q8WUY1	4471119	2893271	4471119	1571102	27	6	9	6	208	9.55	1.71	6	12.293	0	THEM6	High	High
-5831318780158990000	Q9H0C8	1747229	1129758	1747229	613480.7	6	3	3	3	392	7.09	2.11	3	9.071	0	ILKAP	High	High
5802259196988860000	Q9NUY8	1842678	1191098	1842678	646789.2	5	3	6	3	699	5.41	5.8	3	10.591	0	TBC1D23	High	High
4819475231177220000	O94966	331864.6	214466.5	331864.6	116459.5	1	2	2	2	1318	6.28	0	2	3.878	0	USP19	High	Peak Found
-8690205901175620000	Q9NXS2	1623799	1049261	1623799	569769.3	6	2	4	2	382	9.82	0	2	6.37	0	QPCTL	High	High
1595519607435160000	P31942	22092844	14261505	22092844	7744275	17	5	10	5	346	6.87	18.13	5	20.815	0	HNRNPH3	High	High
6535746548150780000	Q6MZP7	597044.5	385029	597044.5	209078.2	1	1	3	1	749	9.01	0	1	2.364	0.008	LIN54	High	High

9042131036706180000	Q13485	126050.7	81239.81	126050.7	44114.8	1	1	1	1	552	6.99	0	1	2.134	0.01	SMAD4	High	Peak Found
623640857413414000	P61981	27267649	17572021	27267649	9541949	48	16	74	8	247	4.89	108.09	16	69.434	0	YWHAG	High	High
636638520442762000	P54725	3081310	1985572	3081310	1078204	12	5	11	3	363	4.58	9.85	5	14.224	0	RAD23A	High	High
4430928365987490000	Q9Y277	4456779	2871495	4456779	1559278	14	5	8	5	283	8.66	6.29	5	14.788	0	VDAC3	High	High
6707926075363840000	Q99798	26112329	16786771	26112329	9115543	29	19	38	19	780	7.61	63.03	19	72.905	0	ACO2	High	High
-8097806180956090000	Q16576	49992311	32118919	49992311	17441198	25	10	29	4	425	5.05	37.03	10	41.158	0	RBBP7	High	High
1687384969150090000	Q96B97	379193.8	243622.3	379193.8	132291.6	2	1	1	1	665	6.62	0	1	2.77	0.004	SH3KBP1	High	Peak Found
1738440604026580000	P60510	430865.8	276680.8	430865.8	150243.1	7	3	6	1	307	5.06	8.73	3	15.425	0	PPP4C	High	Peak Found

-5379927886480690000	O95456	1452345	932080.9	1452345	506138.1	14	4	4	4	288	7.17	3.82	4	11.713	0	PSMG1	High	High
5237090727857760000	O76054	1473481	945324.1	1473481	513329.4	13	4	7	4	403	7.84	0	4	9.971	0	SEC14L2	High	High
-6612546022737720000	P80303	6219095	3986852	6219095	2164938	14	6	12	6	420	5.12	12.82	6	28.095	0	NUCB2	High	High
-5570011762610830000	Q99685	520447	333296.7	520447	180986.6	12	3	4	3	303	6.99	1.62	3	8.458	0	MGLL	High	High
6640986681869860000	P40123	5996003	3838994	5996003	2084648	16	6	22	5	477	6.37	32.98	6	40.066	0	CAP2	High	High
8234678623153020000	Q15165	696991.1	446166.9	696991.1	242277.3	6	2	2	2	354	5.6	0	2	5.34	0		High	Peak Found
-5965687288212210000	Q7L2H7	8052530	5154194	8052530	2798828	17	7	10	7	374	5.63	15.46	7	28.345	0	EIF3M	High	High

-4795865386403900000	Q9Y4Z0	2204206	1410452	2204206	765902.7	15	3	5	3	139	9.99	0	3	4.635	0	LSM4	High	High
8924414405991960000	P98179	983157	628358.8	983157	341211	18	2	4	2	157	8.91	1.65	2	7.419	0	RBM3	High	High
4832137807520600000	O60934	870576.6	555924.4	870576.6	301877.8	3	2	2	2	754	6.9	1.78	2	5.732	0	NBN	High	Peak Found
-2487454136105370000	O15321	3635994	2321015	3635994	1260356	13	7	14	7	606	7.17	12.54	7	22.292	0	TM9SF1	High	High
-5978569898189530000	O75694	7400843	4723895	7400843	2565167	12	13	27	13	1391	6.16	39.65	13	49.016	0	NUP155	High	High
2502404100025200000	P27824	93656952	59741132	93656952	32440596	37	21	66	21	592	4.6	105.85	21	109.303	0	CANX	High	High
-6816817128509430000	P10155	14211498	9062830	14211498	4921293	8	5	13	5	538	8.03	18.18	5	18.524	0	TROVE2	High	High

-8775687582443830000	Q9H7C4	1094059	697506.5	1094059	378759.6	6	3	3	3	482	4.61	1.77	3	6.596	0	SYNC	High	Peak Found
-6585678134614880000	Q9NTK5	20217876	12887744	20217876	6998296	40	15	35	15	396	7.81	42.14	15	56.836	0	OLA1	High	High
-6990547013884610000	Q5JSH3	1404267	895114.8	1404267	486064.7	2	2	5	2	913	5.45	2.1	2	7.498	0	WDR44	High	High
-6066576864868270000	Q9Y263	2880381	1835825	2880381	996888.7	13	8	12	8	795	6.37	15.02	8	25.901	0	PLAA	High	High
-2297110738803860000	P30044	43742535	27844428	43742535	15120066	21	4	26	4	214	8.7	34.12	4	25.655	0	PRDX5	High	High
-4396257609671430000	Q8IZL8	2185224	1389638	2185224	754600.6	7	6	7	6	1130	4.34	1.69	6	17.019	0	PELP1	High	High

9046713427756660000	Q05209	186095.8	118248.6	186095.8	64211.27	1	1	1	1	780	5.62	0	1	2.386	0.008	PTPN12	High	Peak Found
-4642544614061210000	P38159	22763706	14455846	22763706	7849805	24	12	38	8	391	10.05	52.22	12	54.053	0	RBMX	High	High
-77584884133553600	Q9UMY4	1792054	1137713	1792054	617800.5	19	4	11	3	162	8.44	4.92	4	9.096	0	SNX12	High	High
7261585696304070000	O14681	1050181	666527.8	1050181	361937.6	2	1	2	1	340	9.72	1.73	1	2.617	0.006	EI24	High	High
3767865638315370000	O60684	11485713	7288720	11485713	3957917	15	8	12	4	536	4.98	12.45	8	25.793	0	KPNA6	High	High
-8676466481375870000	P08133	1.62E+08	1.03E+08	1.62E+08	55920511	47	32	144	32	673	5.6	206.2	32	161.557	0	ANXA6	High	High
4272754808403180000	Q6PIU2	23249326	14741564	23249326	8004956	26	10	28	10	408	7.23	28.99	10	45.621	0	NCEH1	High	High
607862214588	Q6YHK3	2967242	1880831	2967242	1021328	4	5	8	5	14	5.85	7.92	5	14.226	0	CD109	High	High

6940000										45								
-3273487821867750000	Q96DV4	1962966	1244048	1962966	675542	10	4	9	4	380	7.53	2.05	4	11.452	0	MRPL38	High	High
-8451694186259980000	Q86W92	1946481	1233481	1946481	669804.2	5	6	8	6	1011	5.55	4.36	6	17.092	0	PPFIBP1	High	High
-5611096213630670000	P11279	21668471	13730724	21668471	7456050	9	4	11	4	417	8.75	17.5	4	13.847	0	LAMP1	High	High
4240995388596950000	Q08379	3966730	2513199	3966730	1364716	11	9	13	9	1002	5.02	17.45	9	32.193	0	GOLGA2	High	High
-3013067335374830000	Q13153	188348.4	119316.4	188348.4	64791.11	6	3	3	1	545	5.76	0	3	5.605	0	PAK1	High	Peak Found
3951709946097310000	P78362	1369245	867251	1369245	470934.1	4	3	5	2	688	4.97	3.63	3	7.851	0	SRPK2	High	Peak Found

7804593392060360000	P14324	10623221	6722223	10623221	3650298	13	5	14	5	419	6.15	21.82	5	23.433	0	FDPS	High	High
-5794386193398090000	Q9NY33	6723920	4254460	6723920	2310254	15	9	19	9	737	5.1	16.32	9	33.961	0	DPP3	High	High
4489642059379380000	Q6PD62	1237202	782671.7	1237202	425006	2	3	3	3	1173	6.77	1.83	3	5.687	0	CTR9	High	Peak Found
6727890501693240000	Q9UH99	7062059	4467261	7062059	2425810	16	9	19	9	717	6.73	28.4	9	40.494	0	SUN2	High	High
-2429984818239480000	O60488	4086525	2583111	4086525	1402679	15	10	16	9	711	8.38	19.06	10	35.748	0	ACSL4	High	High
8561643854279220000	P15927	3500128	2211313	3500128	1200786	14	3	6	3	270	6.15	1.78	3	8.759	0	RPA2	High	High
-9151222149414700000	Q92538	7363124	4647285	7363124	2523566	8	13	22	13	1859	5.73	21.99	13	44.592	0	GBF1	High	High

-6625337675714680000	P33176	41061899	25913609	41061899	14071593	34	31	64	31	963	6.51	84.03	31	131.57	0	KIF5B	High	High
-4721604686666430000	P07947	2027094	1279003	2027094	694523.7	7	4	5	4	543	6.74	6.22	4	11.759	0	YES1	High	High
1332536426068470000	Q9UGP8	6109304	3851509	6109304	2091444	14	9	19	9	760	5.31	22.67	9	30.849	0	SEC63	High	High
-445851658510322000	P55769	5181833	3266724	5181833	1773895	19	2	3	2	128	8.46	2.89	2	8.917	0	NHP2L1; SNU13	High	High
7881821209807720000	O75153	11865031	7474351	11865031	4058718	11	15	31	15	1309	6.13	32.39	15	50.242	0	CLUH; KIAA0664	High	High
3797665255168140000	P46087	5256226	3310132	5256226	1797466	9	6	11	6	812	9.23	16.99	6	22.214	0	NOP2	High	High
6390005039364420000	Q96FZ7	328102.6	206396.9	328102.6	112077.5	6	1	1	1	201	5.31	2.2	1	6.237	0	CHMP6	High	Peak Found

4751284344252290000	P06748	2.29E+08	1.44E+08	2.29E+08	78217715	36	11	48	11	294	4.78	71.77	11	72.682	0	NPM1	High	High
-537602968940689000	P30153	58306401	36626756	58306401	19889041	31	16	55	16	589	5.11	62.59	16	71.154	0	PPP2R1A	High	High
-5658162067205240000	Q969E2	1705157	1069955	1705157	581006.6	5	1	3	1	229	8.82	4.62	1	4.352	0	SCAMP4	High	High
-3881079650873620000	P29466	1139250	714821.3	1139250	388161.8	10	3	4	3	404	5.91	1.75	3	10.486	0	CASP1	High	High
-2629792950046780000	Q12849	2647052	1659249	2647052	901004.5	6	3	7	3	480	6.19	6.34	3	10.572	0	GRSF1	High	High
-8135385361793160000	Q9BQA1	5228227	3276999	5228227	1779474	6	2	6	2	342	5.17	8.69	2	7.608	0	WDR77	High	High
299367675131543000	Q9NWS8	79919.16	50077.23	79919.16	27192.91	2	1	1	1	449	8.69	0	1	2.504	0.007	RMND1	High	Peak Found

-5997632050867010000	Q9UGR2	250663	157019.9	250663	85264.88	3	3	4	3	977	7.25	3.53	3	7.951	0		High	High
6397306723786700000	O76024	2950876	1847887	2950876	1003438	6	5	7	5	890	8.05	5.49	5	11.704	0	WFS1	High	High
2859585786570280000	P08237	5444370	3409070	5444370	1851191	17	10	27	8	780	7.99	20.7	10	44.464	0	PFKM	High	High
2613471079569610000	P14866	40764533	25524952	40764533	13860545	27	11	36	11	589	8.22	52.97	11	52.327	0	HNRNPL	High	High
1391397350537540000	O95210	320789	200858.6	320789	109070.1	4	1	1	1	358	5.73	0	1	2.24	0.009	STBD1	High	Peak Found
-7463306019950860000	O60784	1141012	714073.8	1141012	387756	8	2	3	2	492	4.7	2.38	2	6.925	0	TOM1	High	High
8833094404749320000	Q9Y285	9019977	5630651	9019977	3057553	22	9	18	9	508	7.8	19.37	9	40.937	0	FARSA	High	High
-811271	Q8TAQ2	2627179	1638635	2627179	889810.8	4	6	10	2	12	5.69	11.36	6	16.304	0	SMARCC2	High	Peak Found

2839841420000										14								
-6804108956499340000	Q96G03	7912726	4934598	7912726	2679583	9	6	10	6	612	6.73	7.8	6	24.109	0	PGM2	High	High
-294736394566280000	Q16204	2162945	1348589	2162945	732309.9	11	5	7	5	474	7.34	11.05	5	17.549	0	CCDC6	High	High
7331213768411440000	O95777	802463.6	499528.1	802463.6	271253.5	10	1	2	1	96	4.48	4.15	1	2.552	0.006	NAA38; LSM8	High	Peak Found
6853958471097070000	Q5T9L3	7810730	4861728	7810730	2640013	9	6	17	6	541	7.36	11.05	6	13.004	0	WLS	High	High
-3988284594388070000	Q92974	4497321	2797789	4497321	1519254	9	9	16	9	986	7.27	24.13	9	29.076	0	ARHGEF2	High	High
-5432753500567820000	O75390	34257498	21309905	34257498	11571693	20	11	38	11	466	8.32	39.74	11	41.135	0	CS	High	High

-1831448905794710000	P84098	16419130	10212525	16419130	5545599	13	3	6	3	196	11.47	11.98	3	14.016	0	RPL19	High	High
-5424828533558390000	O95352	632657.1	393353.1	632657.1	213598.4	1	1	1	1	703	6.24	0	1	3.939	0	ATG7	High	Peak Found
-1166927996766440000	P28482	6417413	3988533	6417413	2165851	27	8	21	6	360	6.98	11.66	8	29.982	0	MAPK1	High	High
-5554010069989840000	Q9BZZ5	8254743	5129336	8254743	2785329	14	7	16	7	524	7.34	12.03	7	23.571	0	API5	High	High
-2088218542987520000	P49005	929393.5	576500.1	929393.5	313050.8	9	3	5	3	469	5.58	3.68	3	11.891	0	POLD2	High	High
-4563440572163440000	P60520	1367494	847429.1	1367494	460170.5	13	2	4	2	117	8.1	3.57	2	5.151	0	GABARAPL2	High	High

-5479844659917980000	Q9NQ48	434980.2	269491.6	434980.2	146339.2	5	1	1	1	299	5.36	0	1	3.234	0.002	LZTFL1	High	Peak Found
7738217365816990000	P41250	46690720	28927061	46690720	15707957	28	22	63	22	739	7.03	79.68	22	103.376	0	GARS	High	High
-3557205944014880000	P20339	3043942	1885316	3043942	1023763	33	6	21	3	215	8.15	29.85	6	27.075	0	RAB5A	High	High
-3902641514385650000	Q5BKZ1	757516.6	469095.9	757516.6	254728.2	8	4	5	4	582	5.15	1.68	4	11.741	0	ZNF326	High	High
7547854547274610000	Q66K74	1145928	709392.2	1145928	385213.8	4	3	5	3	1059	7.3	4.61	3	7.775	0	MAP1S	High	High
8735053905858640000	Q16401	10743768	6646124	10743768	3608975	22	10	21	10	504	5.48	28.49	10	35.011	0	PSMD5	High	High
3821236340275180000	Q9UL42	716889.5	443375.4	716889.5	240761.5	4	2	2	2	364	4.86	0	2	3.925	0	PNMA2	High	Peak Found

3972038562420250000	P61254	54014546	33401582	54014546	18137709	50	11	33	2	145	10.55	47.22	11	46.019	0	RPL26	High	High
1754940364025580000	Q9Y6R0	265048.8	163824	265048.8	88959.63	3	2	5	1	609	8.85	0	2	5.404	0	NUMBL	High	Peak Found
-4344816618369670000	Q16537	1629947	1007379	1629947	547026.5	8	3	6	3	467	6.95	2.44	3	9.377	0	PPP2R5E	High	High
6857356928160300000	Q9C0E8	2176960	1344386	2176960	730027.8	15	5	8	5	428	5.11	1.68	5	16.081	0	KIAA1715; LNPK	High	High
3253297407861630000	O95155	1569787	969083.7	1569787	526231.3	2	3	6	3	1302	6.55	0	3	9.744	0	UBE4B	High	High
-6970631393786120000	Q8WU79	5197235	3207320	5197235	1741637	11	5	14	4	429	8.87	23.1	5	18.497	0	SMAP2	High	High
-6570966043889320000	Q15437	1579657	974353.8	1579657	529093.1	7	5	12	3	767	6.89	11.14	5	16.215	0	SEC23B	High	High

3303304153352380000	P33527	2594461	1599072	2594461	868327.2	4	6	10	6	1531	7.11	5.47	6	19.604	0	ABCC1	High	High
7607180034342260000	Q6P179	5158217	3178513	5158217	1725994	8	8	12	8	960	6.71	15.84	8	24.074	0	ERAP2	High	High
-3685684818087550000	P49755	34396637	21180695	34396637	11501529	21	6	22	6	219	7.44	40.15	6	44.049	0	TMED10	High	High
9116145290149310000	P42226	1864287	1147899	1864287	623331.5	4	4	5	4	847	6.23	0	4	10.265	0	STAT6	High	High
-6493019114107400000	Q6NUM9	1930456	1188143	1930456	645184.6	7	4	7	4	610	8.28	6.58	4	15.718	0	RETSAT	High	High
6312780505627040000	Q96E39	177882.5	109476.8	177882.5	59448.05	14	5	12	1	390	9.89	23.51	5	23.561	0	RBMXL1	High	Peak Found
-1224070834007840000	Q9BW91	943068.4	579665.4	943068.4	314769.6	7	2	3	2	350	8.22	2.09	2	7.247	0	NUDT9	High	Peak Found

-3274754965853710000	Q9BWD1	12053416	7403414	12053416	4020198	25	8	14	8	397	6.92	23.42	8	30.536	0	ACAT2	High	High
4314325281228420000	O15382	1681348	1031654	1681348	560208.3	15	4	7	4	392	8.65	8.9	4	19.054	0	BCAT2	High	High
7002487047349750000	P22392	8389097	5145099	8389097	2793889	57	8	44	3	152	8.41	74.75	8	39.423	0	NME2; NME1-NME2	High	High
5016840321552790000	Q96KP1	1124654	689065.8	1124654	374176.1	3	3	5	3	924	6.9	4.38	3	8.306	0	EXOC2	High	High
6574041226867980000	Q9HA65	2319365	1420690	2319365	771462.3	5	3	5	3	648	5.19	3.73	3	8.901	0	TBC1D17	High	High
3851021478040600000	P31939	38806558	23767635	38806558	12906288	35	16	48	16	592	6.71	65.73	16	66.085	0	ATIC	High	High
-1940412608894290000	Q6P9B6	706642.8	432726.2	706642.8	234978.8	4	1	2	1	456	6.24	2.22	1	3.73	0.001	KIAA1609; TLDC1	High	Peak Found
154040472327	P52333	603016.1	369185.6	603016.1	200475	1	1	2	1	11	7.18	0	1	2.114	0.01	JAK3	High	High

4750000										24								
8386147216843780000	Q9NUU7	9097073	5565943	9097073	3022415	23	11	19	11	478	6.58	18.09	11	34.851	0	DDX19A	High	High
4780754661784180000	O95373	19124271	11697778	19124271	6352121	16	17	32	17	1038	4.82	38.15	17	69.538	0	IPO7	High	High
-108024960006830000	Q99873	19147947	11711371	19147947	6359502	27	11	34	11	371	5.35	53.48	11	75.8	0		High	High
6333223318553190000	Q14919	1540622	941813.1	1540622	511422.8	10	2	5	2	205	5.17	8.38	2	7.751	0	DRAP1	High	High
8184976527645460000	Q8N5M4	269901.3	164981.4	269901.3	89588.1	16	2	3	2	171	8.92	0	2	8.883	0	TTC9C	High	High
2413702222549110000	P13693	24652306	15059763	24652306	8177744	33	5	28	5	172	4.93	33.94	5	33.673	0	TPT1	High	High
3983981062552350000	Q92600	1151652	703522.3	1151652	382026.3	6	2	3	2	299	8.03	0	2	4.606	0	RQCD1; CNOT9	High	High

-3285411290895150000	Q9H6H4	245768.3	150092.4	245768.3	81503.08	4	1	2	1	257	9.73	1.79	1	3.953	0	REEP4	High	Peak Found
2706346813500610000	Q9C0C9	6721728	4103052	6721728	2228037	7	7	8	7	1292	5.12	0	7	18.45	0	UBE2O	High	Peak Found
5740436620154940000	Q9BX68	1576172	961718.4	1576172	522231.8	15	2	3	2	163	9.16	0	2	7.676	0	HINT2	High	Peak Found
5860856138791640000	Q9Y5A9	1039318	633725.8	1039318	344125.4	8	4	8	2	579	8.79	14.4	4	15.987	0	YTHDF2	High	Peak Found
-7946497630754710000	Q9H223	11570734	7052129	11570734	3829443	26	15	20	8	541	6.76	23.17	15	47.226	0	EHD4	High	High
-7431062919970500000	P62891	86096891	52458062	86096891	28485748	24	2	12	2	51	12.56	22.11	2	10.983	0	RPL39; RPL39P3	High	High
9139942290346830000	P30084	11353820	6914430	11353820	3754670	18	6	20	6	290	8.07	22.37	6	28.046	0	ECHS1	High	High

-8481404637455690000	Q9C0B1	1416096	862388.7	1416096	468293.8	5	3	5	3	505	5.22	0	3	6.958	0	FTO	High	High
-8127793230594190000	Q9ULH1	526465.3	320602.2	526465.3	174093.2	2	2	2	1	1129	7.31	2.01	2	6.205	0	ASAP1	High	Peak Found
609844474726423000	P63279	7542415	4592860	7542415	2494012	23	5	15	5	158	8.66	15.49	5	21.504	0	UBE2I	High	High
-3564822305630520000	Q96M27	2852052	1736387	2852052	942892.1	10	3	6	3	445	5.83	4.05	3	8.034	0	PRRC1	High	High
-5242933546907130000	Q9H0V9	656980.4	399761.4	656980.4	217078.2	6	2	2	2	348	8.38	0	2	3.396	0.002	LMAN2L	High	High
3596151466959210000	Q9HD45	7591470	4619008	7591470	2508211	13	7	14	7	589	7.21	6.87	7	26.21	0	TM9SF3	High	High
-7858307060055430000	Q9HAB8	3038074	1847848	3038074	1003417	15	5	10	5	311	6.71	8.48	5	13.447	0	PPCS	High	High

59184659773015500	Q969H8	27517997	16732900	27517997	9086290	27	4	20	4	173	6.68	35.82	4	36.225	0	C19orf10; MYDGF	High	High
-1468233501590490000	O60832	5703109	3467634	5703109	1882993	10	5	10	5	514	9.42	13.59	5	14.151	0	DKC1	High	High
-1053300951170360000	Q9UIG0	8715816	5298082	8715816	2876962	2	3	4	3	1483	8.48	1.77	3	4.895	0	BAZ1B	High	High
4457645785715090000	Q9BS26	9944756	6042766	9944756	3281339	14	5	13	5	406	5.26	12.76	5	27.296	0	ERP44	High	High
-61842363996650000	P19623	31852022	19339557	31852022	10501755	28	8	25	8	302	5.49	41.56	8	40.904	0	SRM	High	High
8286530119888380000	P36969	2037860	1237013	2037860	671722.1	28	4	6	4	197	8.37	2.49	4	16.208	0	GPX4	High	High
-3539568279205580000	P57764	2401335	1457643	2401335	791528.3	5	2	2	2	484	5.08	2.17	2	5.012	0	GSDMD	High	Peak Found

7530688595643980000	Q13510	4211954	2555465	4211954	1387667	7	3	9	3	395	7.62	11.47	3	9.889	0	ASAH1	High	High
-216911938682189000	Q27J81	2821319	1709693	2821319	928396.4	6	6	9	6	1249	5.38	5.96	6	17.487	0	INF2	High	High
-6626039175338170000	Q9NXH9	1797959	1089352	1797959	591539.3	9	5	7	5	659	7.64	4.44	5	14.433	0	TRMT1	High	High
5135742112230700000	Q92791	5066257	3068795	5066257	1666415	12	5	9	5	437	4.77	11.75	5	20.586	0	LEPREL4; P3H4	High	High
6101173507286650000	O43852	84481723	51163064	84481723	27782539	53	17	67	17	315	4.64	102.17	17	137.164	0	CALU	High	High
-1340431599886030000	O60783	455847.3	276054	455847.3	149902.7	10	1	1	1	128	11.41	0	1	3.607	0.001	MRPS14	High	Peak Found
3549742544018310000	O14744	6783594	4106859	6783594	2230104	17	10	23	10	637	6.29	13.31	10	28.573	0	PRMT5	High	High

7449781646133500000	O15533	1774049	1073181	1774049	582758.4	2	1	1	1	448	7.15	0	1	3.394	0.002	TAPBP	High	Peak Found
-8432467825191750000	Q05193	543454.4	328715.5	543454.4	178498.9	14	12	20	3	864	7.17	12.86	12	32.073	0	DNM1	High	High
-8271257472643540000	Q6PCE3	6189679	3742613	6189679	2032311	12	7	15	7	622	7.15	10.95	7	20.844	0	PGM2L1	High	High
1254845465696570000	Q9Y282	2507744	1515894	2507744	823160	7	3	3	3	383	6.06	2.15	3	6.441	0	ERGIC3	High	Peak Found
-6289103138971430000	Q92520	6072461	3670456	6072461	1993129	26	6	13	6	227	8.29	14.69	6	22.402	0	FAM3C; WNT16	High	High
-7801299591553380000	P52298	1571244	949669.3	1571244	515688.9	12	2	6	2	156	8.21	4.03	2	7.496	0	NCBP2	High	High
-4844416647535230000	O00233	3416933	2065002	3416933	1121336	19	4	8	4	223	6.95	10.98	4	14.283	0	PSMD9	High	High

6825792350743240000	P07738	5051087	3051101	5051087	1656807	27	6	14	6	259	6.54	7.27	6	19.058	0	BPGM	High	High
4308008512399910000	A1X283	3402709	2054742	3402709	1115765	6	5	10	5	911	8.69	4.13	5	14.521	0	SH3PXD2B	High	High
6803776007100690000	P51580	1157453	698770.6	1157453	379446	9	3	4	3	245	6.23	0	3	6.797	0	TPMT	High	High
-2753373290981070000	Q8IXI1	1831943	1103603	1831943	599277.8	5	3	7	2	618	5.86	3.64	3	11.899	0	RHOT2	High	High
-4556564044268480000	Q9NR19	988914.3	595681.2	988914.3	323466.5	3	3	4	3	701	6.46	0	3	6.803	0	ACSS2	High	Peak Found
-3173318319984310000	Q9BXR0	351286.1	211580.2	351286.1	114892.2	3	1	2	1	403	7.23	0	1	3.491	0.002	QTRT1	High	Peak Found
8372900262212060000	P09651	1.74E+08	1.05E+08	1.74E+08	56762322	49	20	70	16	372	9.13	129.1	20	144.036	0	HNRNPA1	High	High

688091036263169000 0	Q969N2	4466090	2684741	4466090	1457866	10	6	8	6	578	8.38	6.12	6	18.526	0	PIGT	High	High
-186690803580481000 0	P22059	3499298	2102963	3499298	1141950	11	7	12	7	807	7.3	6.21	7	18.447	0	OSBP	High	High
-378745977637446000 0	Q9Y5L0	1969481	1183307	1969481	642558.5	7	5	7	5	923	5.57	6.75	5	16.273	0	TNPO3	High	High
117864606115256000 0	P40938	2490137	1495484	2490137	812076.6	14	4	6	4	356	8.34	3.94	4	14.619	0	RFC3	High	High
-630046723299452000 0	Q8NBX0	5489140	3290631	5489140	1786877	16	5	8	5	429	9.14	9.62	5	26.988	0	SCCPDH	High	High
-831205750513070000 0	P06865	3594322	2153460	3594322	1169371	12	7	11	6	529	5.16	6.49	7	16.674	0	HEXA	High	High
-523312844645	Q8IVD9	2261472	1354492	2261472	735515.4	6	2	2	2	361	5.25	0	2	4.01	0	NUDCD3	High	Peak Found

1310000																		
-7988829974470130000	P29322	273048.1	163447.1	273048.1	88754.98	1	1	1	1	1005	8.06	1.8	1	3.64	0.001	EPHA8	High	Peak Found
-8157399823022380000	Q99567	1216516	728074.8	1216516	395358.8	11	5	5	5	741	5.69	7.06	5	19.994	0	NUP88	High	High
-1411210494299510000	Q9H5K3	199580.5	119423.8	199580.5	64849.46	5	1	1	1	350	6.1	0	1	2.082	0.01	POMK; SGK196	High	Peak Found
-4081821255654730000	Q13618	2433746	1455276	2433746	790243.4	7	5	7	5	768	8.48	3.27	5	12.884	0	CUL3	High	High
-5198752139468270000	Q96PK6	39094026	23355878	39094026	12682696	11	6	12	6	669	9.67	17.45	6	34.157	0	RBM14; RBM14-RBM4	High	High
-7369563390658140000	L0R6Q1	474480.8	283391.1	474480.8	153886.9	10	1	1	1	103	8.1	1.77	1	2.973	0.003	SLC35A4	High	Peak Found

4045429465415990000	Q3ZCQ8	5327783	3179785	5327783	1726685	16	6	10	6	353	8.37	9.44	6	18.234	0	TIMM50	High	High
947873724462567000	Q9UNQ2	1474997	879414.6	1474997	477539.3	10	3	7	3	313	9.99	4.2	3	10.024	0	DIMT1	High	High
3948795116760060000	Q7Z2Z2	595901.7	355107.5	595901.7	192830.3	1	2	3	2	1120	5.91	1.71	2	4.209	0	EFTUD1; EFL1	High	High
-7637669709604960000	Q12888	1189869	709002	1189869	385001.8	3	4	4	4	1972	4.7	4.69	4	10.457	0	TP53BP1	High	High
-7806294032372270000	O15260	12614552	7514208	12614552	4080361	10	3	10	3	269	7.78	12.93	3	10.059	0	SURF4	High	High
4678846149867540000	Q9H4A4	5960840	3546827	5960840	1925996	16	8	16	8	650	5.74	21.24	8	29.275	0	RNPEP	High	High
1235307152007640000	O14949	1137165	676509.4	1137165	367357.7	28	2	8	2	82	10.08	10.88	2	10.779	0	UQCRQ	High	High
541662558935	P09038	2091973	1243823	2091973	675419.9	6	2	4	2	288	11.18	1.99	2	4.064	0	FGF2	High	High

2090000																		
6962760823323830000	O43681	10431417	6193145	10431417	3362998	36	8	19	8	348	4.91	22.15	8	34.401	0	ASNA1	High	High
1246779234316330000	P49773	17772616	10538886	17772616	5722820	44	5	13	5	126	6.95	26.18	5	35.222	0	HINT1	High	High
2530096589731870000	Q96EK5	1700702	1008029	1700702	547379.1	11	5	8	5	621	5.49	3	5	17.469	0	KIAA1279; KIF1BP	High	High
-2687954280174460000	Q96S97	3476957	2059490	3476957	1118343	14	3	10	3	322	8.15	9.31	3	10.247	0	MYADM	High	High
-65550439794871300	Q9BXS5	7387269	4372689	7387269	2374455	23	8	21	8	423	7.3	10.74	8	33.107	0	AP1M1	High	High
3959098677357930000	O43747	8499818	5024846	8499818	2728589	9	8	13	8	822	6.8	12.41	8	30.157	0	AP1G1	High	High
6072091565905250000	P42574	1438492	849053	1438492	461052.3	7	2	5	2	277	6.54	4.01	2	5.206	0	CASP3	High	High

4784462983212680000	Q96KP4	3964693	2339118	3964693	1270187	15	7	12	7	475	5.97	6.58	7	20.038	0	CNDP2	High	High
1871609499973020000	Q07021	5939371	3503720	5939371	1902588	17	3	9	3	282	4.84	9.51	3	16.026	0	C1QBP	High	High
7222850354556000000	P20839	9870199	5816347	9870199	3158390	16	7	13	5	514	6.9	11.91	7	19.049	0	IMPDH1	High	High
-3011469150095820000	Q9Y696	42530973	25030271	42530973	13591924	57	10	24	10	253	5.59	48	10	70.103	0	CLIC4	High	High
5530158233237020000	Q15075	14129979	8308881	14129979	4511884	21	25	38	25	1411	5.68	46.62	25	99.555	0	EEA1	High	High
4342326397945370000	O75150	647143	380484.4	647143	206610.4	3	3	3	3	1001	6.29	2.16	3	10.334	0	RNF40	High	Peak Found
3090295747624370000	Q9NTJ5	9443516	5551831	9443516	3014752	30	16	24	16	587	7.12	22.28	16	53.666	0	SACM1L	High	High
264563073648	P04080	20134320	11836571	20134320	6427488	55	4	22	4	98	7.56	41.01	4	32.771	0	CSTB	High	High

8310000																		
9194436757358920000	Q5W0V3	1656206	973354.3	1656206	528550.3	7	5	7	5	765	5.29	6.3	5	16.937	0	FAM160B1	High	High
-2843591473366040000	P62136	25395779	14921455	25395779	8102640	35	10	25	3	330	6.33	28.13	10	55.093	0	PPP1CA	High	High
1500073560633060000	P40261	14132680	8303096	14132680	4508742	14	4	8	4	264	5.74	16.17	4	13.652	0	NNMT	High	High
-1707703891335850000	Q7Z478	12956627	7609156	12956627	4131920	5	7	11	7	1369	8.09	8.19	7	19.428	0	DHX29	High	High
6538585977691410000	Q9BYB4	321352.5	188513.4	321352.5	102366.4	2	1	3	1	327	7.97	0	1	2.204	0.009	GNB1L	High	High
309399874667332000	Q9BQE5	2055865	1205556	2055865	654640.3	12	4	7	4	337	6.74	6.49	4	12.887	0	APOL2	High	High
-8490301965517190000	Q9Y5P8	226178	132212.9	226178	71794.17	1	1	1	1	575	5.14	0	1	2.463	0.007	PPP2R3B	High	Peak Found

-3782870004774890000	Q15796	967365.3	565413.5	967365.3	307030.5	5	2	4	2	467	6.58	1.87	2	5.358	0	SMAD2	High	Peak Found
-1811724669038290000	Q96RP9	6768892	3954232	6768892	2147225	13	8	13	8	751	7.01	19.05	8	23.958	0	GFM1	High	High
7020268087964790000	Q9Y3B7	2602037	1518816	2602037	824746.4	30	5	7	5	192	9.91	9.48	5	14.184	0	MRPL11	High	High
215729870143927000	P52815	1811768	1056904	1811768	573919.3	12	3	3	3	198	8.87	1.74	3	8.913	0	MRPL12	High	Peak Found
934468542703033000	Q15477	1563850	912191.6	1563850	495337.8	7	6	7	6	1246	6.06	6.87	6	20.729	0	SKIV2L	High	High
-2330995334301040000	Q9P2X0	365937.1	213405.2	365937.1	115883.2	13	1	2	1	92	5.94	2.18	1	4.057	0	DPM3	High	High
6271910638518900000	Q92552	1128213	657682.5	1128213	357134.4	6	3	3	3	414	6.18	1.97	3	4.683	0	MRPS27	High	Peak Found
731872381753	Q5VW36	263129.9	153225.7	263129.9	83204.52	1	1	1	1	18	6.62	1.7	1	2.264	0.009	FOCAD	High	Peak Found

7280000										01								
-6640745081908680000	O75521	1048241	609285.9	1048241	330854.1	9	3	3	3	394	9	2.16	3	9.259	0	ECI2	High	High
-4213076317646870000	P16219	9904379	5755918	9904379	3125575	6	2	2	2	412	7.99	1.81	2	6.739	0	ACADS	High	High
8998868825977100000	Q9H3Z4	2023971	1175197	2023971	638154.8	21	4	6	4	198	5.07	7.87	4	17.549	0	DNAJC5	High	High
-6755213045612550000	P53367	3337512	1936556	3337512	1051587	14	5	10	5	373	6.7	10.04	5	15.511	0	ARFIP1	High	High
1498458301552960000	P13716	1371226	795275.2	1371226	431849.9	12	3	3	3	330	6.79	2.59	3	11.726	0	ALAD	High	High
4612354553252090000	Q12981	242599	140688.9	242599	76396.82	4	1	1	1	228	8.95	0	1	3.35	0.002	BNIP1	High	Peak Found
431721942919	Q9H6Z4	5614414	3249643	5614414	1764619	13	6	11	6	567	4.78	10.62	6	22.616	0	RANBP3	High	High

9650000																		
-2115401272640630000	P41226	2581849	1494172	2581849	811364.2	1	1	1	1	1012	6.04	1.88	1	2.262	0.009	UBA7	High	Peak Found
-1042758069650350000	Q6P1M0	1147273	663717.4	1147273	360411.5	5	3	6	3	643	8.47	3.49	3	7.687	0	SLC27A4	High	High
-474422990152712000	P61923	4625058	2675459	4625058	1452826	14	3	8	3	177	4.81	7.28	3	12.443	0	COPZ1	High	High
-2969987479368300000	O14657	1294263	747925.8	1294263	406138.3	4	2	4	2	336	8.54	0	2	4.606	0	TOR1B	High	High
-100353375695918000	P53999	26173304	15116505	26173304	8208556	46	6	20	6	127	9.6	42.53	6	39.965	0	SUB1	High	High
295020939105715000	Q9NRS6	322473.5	186107.1	322473.5	101059.8	2	1	1	1	342	5.19	0	1	2.337	0.008	SNX15	High	Peak Found
-797104145851	P61201	8668651	5001489	8668651	2715906	25	10	18	10	443	5.53	15.57	10	34.687	0	COPS2	High	High

0100000																		
32097797701443300	Q14008	30458833	17560611	30458833	9535753	11	21	38	21	2032	7.8	43.07	21	72.072	0	CKAP5	High	High
1558588285545910000	O75531	7707192	4442743	7707192	2412496	43	3	4	3	89	6.09	8.38	3	12.676	0	BANF1	High	High
-6467566476375470000	Q03154	1634557	941457.6	1634557	511229.8	10	4	5	4	408	6.18	0	4	7.949	0	ACY1	High	Peak Found
4481377694352560000	P15121	81539035	46924064	81539035	25480679	34	10	30	9	316	6.98	39.4	10	52.592	0	AKR1B1	High	High
2494116660910270000	Q9H814	589457.2	339212.3	589457.2	184198.9	2	1	2	1	394	5.4	0	1	2.484	0.007	PHAX	High	High
-1541335516874270000	Q14527	755233.2	434300	755233.2	235833.3	5	4	5	4	1009	8.6	4.56	4	11.421	0	HLTF	High	Peak Found
8660233523405070000	A0FGR8	7726197	4442118	7726197	2412156	14	11	18	11	921	9.26	22.16	11	36.94	0	ESYT2	High	High

-2333101891394800000	O95573	14862029	8544795	14862029	4639990	24	14	28	13	720	8.38	26.63	14	48.697	0	ACSL3	High	High
-3363379489977260000	P00441	21525455	12372881	21525455	6718715	31	5	15	5	154	6.13	29.14	5	32.334	0	SOD1	High	High
3705334322242740000	Q12788	675913.6	388236.9	675913.6	210820.2	5	3	4	3	808	6.9	1.76	3	6.404	0	TBL3	High	Peak Found
-398928808883879000	P07311	1437248	824548.5	1437248	447745.9	35	3	5	3	99	9.31	11.76	3	13.629	0	ACYP1	High	High
479793662138796000	P61221	27669341	15873539	27669341	8619640	20	10	30	10	599	8.34	38.2	10	53.783	0	ABCE1	High	High
3551068469878700000	Q9H0U6	1035745	594031	1035745	322570.4	12	2	2	2	180	9.54	3.53	2	5.174	0	MRPL18	High	Peak Found
-8081850607027730000	Q9P0J1	929369	532823.3	929369	289333.4	4	3	4	3	537	6.67	1.6	3	5.927	0	PDP1	High	Peak Found

-4379760637260400000	Q9NS69	758121	434487.4	758121	235935.1	8	1	2	1	142	4.34	0	1	4.577	0	TOMM22	High	Peak Found
-8106966376198230000	Q01844	5548778	3174670	5548778	1723907	12	6	16	6	656	9.33	20.6	6	40.519	0	EWSR1	High	High
-3157784244312670000	Q9UKD2	5567261	3184550	5567261	1729273	35	8	14	8	239	8.29	11.1	8	30.049	0	MRTO4	High	High
8696690186535490000	Q9NPJ3	1763650	1007672	1763650	547185.7	9	1	3	1	140	9.14	6.8	1	5.717	0	ACOT13	High	High
-8566707279340010000	Q9Y6M7	4095922	2339268	4095922	1270268	8	9	13	9	1214	6.71	14.84	9	33.553	0	SLC4A7	High	High
-932703795777546000	Q96BJ3	212890.1	121578.3	212890.1	66019.39	7	2	2	2	306	6.55	1.61	2	4.591	0	AIDA	High	Peak Found
-652977595040	Q9UBC2	4174792	2383293	4174792	1294174	11	10	18	10	864	5.11	13.19	10	31.173	0	EPS15L1	High	High

4900000																		
1304014562382960000	O60216	1232275	700572.4	1232275	380424.5	8	4	5	4	631	4.65	7.34	4	15.457	0	RAD21	High	Peak Found
2661453749404060000	P48651	3150618	1790184	3150618	972104.6	9	3	9	3	473	8.43	5.87	3	13.966	0	PTDSS1	High	High
3081383647301720000	Q16540	628130.9	356786.9	628130.9	193742.2	10	1	2	1	153	9.69	4.75	1	6.326	0	MRPL23	High	High
338471368414013000	Q96T60	533529.6	302962.3	533529.6	164514.4	4	2	3	2	521	8.46	2.15	2	6.057	0	PNKP	High	High
4783590985173720000	Q96CN7	749033.1	425047.3	749033.1	230808.9	4	1	2	1	298	7.39	5.6	1	4.849	0	ISOC1	High	Peak Found
6826176289632430000	P29992	6377275	3618038	6377275	1964665	19	7	14	6	359	5.69	11.48	7	21.109	0	GNA11	High	High
5305679341133580000	Q86WR0	315198	178492.5	315198	96924.88	6	1	1	1	208	6.8	0	1	2.584	0.006	CCDC25	High	Peak Found
-819873802843	Q7Z2W4	4644430	2629581	4644430	1427913	8	7	14	7	902	8.4	13.9	7	22.235	0	ZC3HAV1	High	High

7080000																		
-5122002380756430000	Q9Y6E2	28280261	15983571	28280261	8679390	32	16	35	12	419	6.68	39.15	16	52.371	0	BZW2	High	High
-1318153562653380000	O43837	4139914	2339070	4139914	1270160	23	7	10	7	385	8.46	2.48	7	23.344	0	IDH3B	High	High
6733467377289630000	Q13838	5871675	3316617	5871675	1800988	27	13	33	4	428	5.67	45.17	13	47.695	0	DDX39B	High	High
-4476528208765430000	Q9Y5S9	1849045	1043951	1849045	566885.6	22	3	7	3	174	5.72	5.43	3	12.195	0	RBM8A; LOC101060541	High	High
2963381124708490000	Q5JTH9	751623.6	424235.2	751623.6	230368	3	4	5	4	1297	8.75	1.86	4	9.589	0	RRP12	High	High
-2106414634070180000	Q8WVM8	1232571	694096.7	1232571	376908	6	4	4	4	642	6.27	0	4	10.718	0	SCFD1	High	Peak Found

5909232235632670000	Q13418	9100977	5116360	9100977	2778283	25	11	26	11	452	8.07	19.08	11	38.738	0	ILK	High	High
-4559140579210940000	P62917	87506269	49155010	87506269	26692126	22	7	26	7	257	11.03	53.21	7	28.998	0	RPL8	High	High
7629412907813040000	Q14997	3068396	1719500	3068396	933722	3	6	8	6	1843	6.9	2.46	6	18.68	0	PSME4	High	High
-2758038755542970000	P61769	4683813	2624173	4683813	1424977	17	2	6	2	119	6.52	11.9	2	9.581	0	B2M	High	High
-3107820624180600000	O75879	321825	180183.7	321825	97843.23	3	2	2	2	557	8.63	1.93	2	4.239	0	PET112; GATB	High	Peak Found
3707152110506340000	Q9P2B2	3004767	1681821	3004767	913261.3	8	6	11	6	879	6.61	6.05	6	18.396	0	PTGFRN	High	High
5118939103406680000	Q8TEX9	5208305	2909980	5208305	1580176	6	6	12	6	1081	4.96	7.6	6	18.419	0	IPO4	High	High

3531951912742510000	Q14643	601874	336129.9	601874	182525.1	1	4	5	3	2758	6.04	1.81	4	9.596	0	ITPR1	High	Peak Found
-519635040323081000	P60953	16087795	8981371	16087795	4877059	21	4	13	4	191	6.55	10.87	4	16.812	0	CDC42	High	High
-507762156530799000	O43670	279973.6	156164.3	279973.6	84800.27	5	2	2	2	478	9.1	2.14	2	5.1	0	ZNF207	High	Peak Found
-4326632445175340000	P55209	11994560	6686847	11994560	3631088	17	6	25	4	391	4.46	32.49	6	40.664	0	NAP1L1	High	High
-493599392499449000	P53990	3578969	1991076	3578969	1081193	9	4	9	4	364	5.35	9.38	4	12.864	0	IST1	High	High
-1455362657326630000	Q02543	46731444	25989922	46731444	14113033	39	7	21	7	176	10.71	39.46	7	29.404	0	RPL18A	High	High
5045228690157750000	Q9Y4E6	934903.4	519532.8	934903.4	282116.4	1	1	2	1	1490	6.92	0	1	2.519	0.006	WDR7	High	Peak Found

-1264593624988500000	Q14694	5256375	2919986	5256375	1585609	14	8	14	8	798	5.31	12.15	8	28.87	0	USP10	High	High
4128551820349880000	P51531	216364.9	120164.7	216364.9	65251.78	4	5	7	1	1590	7.2	8.17	5	21.152	0	SMARCA2	High	High
-8008274239603490000	P51991	40006357	22202093	40006357	12056168	30	15	40	12	378	9.01	54.63	15	79.817	0	HNRNPA3	High	High
-4605522544337800000	Q9Y3U8	1813303	1004359	1813303	545386.4	20	2	4	2	105	11.59	7.19	2	6.047	0	RPL36	High	High
-3689472973843480000	O00505	2308621	1276946	2308621	693406.4	9	4	9	2	521	4.94	8.93	4	24.772	0	KPNA3	High	High
3667927535039760000	Q9BV10	195161.7	107885.9	195161.7	58584.16	2	1	1	1	488	9.58	0	1	3.21	0.002	ALG12	High	Peak Found
1669697587989020000	Q96FW1	14364210	7932233	14364210	4307357	27	6	15	6	271	4.94	29.14	6	28.22	0	OTUB1	High	High

-4088167174397650000	Q9BV20	3452042	1902319	3452042	1032996	20	6	9	6	369	6.3	7.83	6	19.143	0	MRI1	High	High
2698789299991650000	Q16611	1962881	1080565	1962881	586767.7	5	1	1	1	211	6.01	0	1	2.543	0.006	BAK1	Peak Found	High
7573763493243980000	P10909	1732898	953588.6	1732898	517817.2	4	2	3	2	449	6.27	0	2	4.548	0	CLU	High	Peak Found
-3774842769131470000	Q9Y2R5	1791716	984989.7	1791716	534868.5	36	3	7	3	130	9.85	3.73	3	11.562	0	MRPS17	High	High
-6056381579766230000	Q9UNH7	6517261	3582196	6517261	1945202	17	8	17	7	406	6.16	21.63	8	30.475	0	SNX6	High	High
8122664404387130000	Q96JP5	140137.1	77009.28	140137.1	41817.54	1	1	1	1	570	7.36	0	1	2.396	0.008	ZFP91	High	Peak Found
3251544276452060000	P26368	5400321	2964328	5400321	1609688	17	5	13	5	475	9.09	7.83	5	31.939	0	U2AF2	High	High

-2375108519350620000	Q13547	14592674	8004175	14592674	4346422	22	8	14	4	482	5.48	16.85	8	32.292	0	HDAC1	High	High
-4634393773107860000	P19474	599353.2	327690.4	599353.2	177942.2	4	2	3	2	475	6.38	1.87	2	5.444	0	TRIM21	High	High
325736081283403000	P35637	20081011	10971965	20081011	5957990	17	9	33	7	526	9.36	61.94	9	68.094	0	FUS	High	High
5272844491039920000	P09661	2228423	1217335	2228423	661036.6	13	3	6	3	255	8.62	6.21	3	10.838	0	SNRPA1	High	High
-486365272632798000	Q8WUW1	664291.4	361974.8	664291.4	196559.3	43	3	5	3	75	5.45	4.13	3	8.472	0	BRK1	High	High
-4920792406220340000	Q8NBM8	288069.7	156696.7	288069.7	85089.38	6	2	3	2	494	7.31	0	2	5.019	0	PCYOX1L	High	High
-6687870354662640000	Q8N3D4	1758462	956298.6	1758462	519288.7	4	5	8	4	1523	4.83	9.37	5	18.01	0	EHBP1L1	High	High

8218797192216080000	P08581	293894.7	159779.2	293894.7	86763.2	1	1	1	1	1390	7.33	0	1	2.311	0.008	MET	High	Peak Found
-6865032565796740000	O15427	1066322	579071.3	1066322	314447	6	3	4	3	465	7.96	0	3	5.791	0	SLC16A3	High	High
-5486623488286150000	Q9UBT2	19982415	10850021	19982415	5891773	26	14	28	14	640	5.29	32.53	14	52.386	0	UBA2	High	High
372640762268439000	O14521	439254.8	238348.5	439254.8	129427.9	6	1	1	1	159	8.63	0	1	2.711	0.005	SDHD	High	Peak Found
-7930539283880310000	Q96DZ1	2301755	1248733	2301755	678086.5	9	4	9	4	483	6.28	7.37	4	13.357	0	ERLEC1	High	High
3313635736816900000	O96019	12041482	6528933	12041482	3545337	37	12	21	12	429	5.6	29.08	12	49.336	0	ACTL6A	High	High
7214504578644120000	P62942	7013735	3800800	7013735	2063908	30	2	5	2	108	8.16	12.46	2	14.615	0	FKBP1A	High	High

-7494244166220140000	O15075	2249578	1217255	2249578	660993.3	8	5	10	5	740	8.66	6.85	5	15.227	0	DCLK1	High	High
-5828511565450600000	P46939	816420.6	441455.6	816420.6	239719	1	4	4	4	3433	5.33	2.23	4	11.611	0	UTRN	High	High
-7491273576453500000	Q9BYN0	2395561	1294780	2395561	703090.9	14	2	2	2	137	8.19	2.37	2	6.568	0	SRXN1	High	Peak Found
-5119731611670530000	O60610	3267110	1764385	3267110	958095.4	3	4	6	4	1272	5.41	3.35	4	14.573	0	DIAPH1	High	High
2333660704476730000	O15397	2136539	1153365	2136539	626299.7	5	5	8	5	1037	5.16	1.93	5	12.463	0	IPO8	High	High
-6575601821171360000	P39019	90529724	48851710	90529724	26527429	63	14	49	14	145	10.32	81.2	14	66.806	0	RPS19	High	High
433554454622	Q9NRX4	1954119	1054007	1954119	572346.5	18	2	3	2	125	6.07	1.9	2	10.245	0	PHPT1	High	Peak Found

2160000																		
6604733771635370000	P84085	1266053	682785.5	1266053	370765.8	19	4	20	1	180	6.79	38.91	4	17.93	0	ARF5	Peak Found	High
-3983028009750610000	Q8N122	746443.7	401974.6	746443.7	218280	1	2	2	2	1335	6.89	0	2	4.48	0	RPTOR	High	High
-5054374183751910000	Q13884	345342.6	185928.6	345342.6	100962.8	5	2	3	2	538	8.63	1.84	2	6.731	0	SNTB1	High	Peak Found
1330138417621240000	P07099	77906237	41912548	77906237	22759328	34	17	33	17	455	7.25	37.93	17	50.322	0	EPHX1	High	High
-7476977248749320000	Q7Z4H8	2383762	1282340	2383762	696335.6	8	4	10	4	507	8.24	7.68	4	11.222	0	KDELC2	High	High
-5267554590826270000	P62380	256858.5	138007.3	256858.5	74940.63	5	1	2	1	186	9.54	1.77	1	3.604	0.001	TBPL1	High	Peak Found

4943408873611000000	Q02790	15121143	8120354	15121143	4409510	38	13	24	13	459	5.43	32	13	52.912	0	FKBP4	High	High
-5996898070928510000	Q96AC1	20724256	11122131	20724256	6039533	23	15	38	14	680	6.7	54.94	15	58.704	0	FERMT2	High	High
2260332764015540000	Q9BTW9	4572418	2453888	4572418	1332509	9	8	15	8	1192	6.19	12.36	8	29.747	0	TBCD	High	High
-2214301499697930000	Q96C86	2829305	1517056	2829305	823791.2	14	5	11	5	337	6.38	7.31	5	16.25	0	DCPS	High	High
-8105981666538770000	P80297	2937288	1572797	2937288	854059.2	33	2	3	2	61	7.96	7.9	2	9.341	0	MT1X	High	High
5815145421053800000	Q16543	15964700	8542026	15964700	4638486	24	10	15	10	378	5.25	22.33	10	39.325	0	CDC37	High	High
-5626917880360890000	Q8N201	420703.8	224632.2	420703.8	121979.7	1	2	2	2	2190	6.13	0	2	3.729	0.001	INTS1	High	Peak Found

3259747315826000000	Q93096	973087.5	517721.8	973087.5	281133	10	2	4	2	173	8.97	0	2	5.323	0	PTP4A1	High	High
-5301300435928160000	P47755	7567014	4022472	7567014	2184281	33	6	20	4	286	5.85	20.94	6	26.57	0	CAPZA2	High	High
-2227008394942670000	Q9BUQ8	4552337	2418657	4552337	1313378	9	8	11	8	820	9.55	11.52	8	20.18	0	DDX23	High	High
-2452493756132290000	P55268	343974.5	182690.4	343974.5	99204.41	1	1	1	1	1798	6.52	0	1	2.547	0.006	LAMB2	High	Peak Found
-6934522081491210000	P11908	496078.5	263319.5	496078.5	142987.6	11	3	8	1	318	6.61	19.5	3	18.095	0	PRPS2	High	Peak Found
-8716615983158350000	P27695	6072235	3222100	6072235	1749663	19	6	11	6	318	8.12	9.18	6	20.63	0	APEX1	High	High
147736943924	Q15155	10744260	5700416	10744260	3095437	12	13	22	13	12	5.81	24.32	13	44.869	0	NOMO1	High	High

2670000										22								
-985839782283071000	P10619	5036422	2671650	5036422	1450758	13	5	8	5	480	6.61	10.3	5	20.076	0	CTSA	High	High
9019009658753380000	P54840	2272645	1204944	2272645	654308.1	3	2	3	1	703	6.83	3.91	2	6.117	0	GYS2	High	High
-2886709730536470000	Q9BTT0	2351050	1245588	2351050	676378.7	10	2	5	2	268	3.85	8.9	2	8.288	0	ANP32E	High	High
-5842118922115640000	P36405	6509141	3447345	6509141	1871975	22	3	3	3	182	7.24	1.96	3	11.756	0	ARL3	High	High
-5309458315612800000	O00178	1448793	767195.9	1448793	416602.3	5	3	5	3	669	8.34	0	3	7.866	0	GTPBP1	High	High
-7785225310514610000	Q9NZ45	2669553	1412138	2669553	766818.3	34	3	5	3	108	9.09	8.31	3	17.322	0	CISD1	High	High

7644877244443840000	Q13177	11552084	6103699	11552084	3314427	34	14	20	12	524	5.96	16.31	14	45.83	0	PAK2	High	High
-3089932679620530000	Q15800	944667.7	498923.4	944667.7	270925.1	8	2	2	2	293	7.23	1.77	2	5.49	0	MSMO1	High	High
1414945439185650000	P84090	1967264	1038220	1967264	563773.8	17	2	4	2	104	5.92	3.68	2	5.54	0	ERH	High	High
-4945442823809020000	O95861	4068255	2144509	4068255	1164510	21	6	9	6	308	5.69	5.73	6	16.783	0	BPNT1	High	High
-8654613729152940000	P23786	5008150	2639275	5008150	1433178	16	9	12	9	658	8.18	8.72	9	31.461	0	CPT2	High	High
5777043105444540000	P00387	73399071	38640099	73399071	20982325	60	16	60	16	301	7.59	114.46	16	94.032	0	CYB5R3	High	High
-4492999109905150000	Q15121	2411992	1268632	2411992	688891.7	17	2	3	2	130	5.02	4.5	2	6.47	0	PEA15	High	High

-3028893805167110000	O75351	924412	483554.4	924412	262579.4	9	3	6	2	444	7.23	4.57	3	11.194	0	VPS4B	Peak Found	High
2454508349601290000	Q99447	1815640	948999.9	1815640	515325.4	7	3	6	3	389	6.92	6.19	3	10.084	0	PCYT2	High	High
-4160171774517720000	P51452	2876566	1503256	2876566	816297	19	3	8	3	185	7.8	11.76	3	14.942	0	DUSP3	High	High
-580922087846668000	P48060	834906.6	435840.9	834906.6	236670.1	12	2	2	2	266	8.48	2.74	2	7.939	0	GLIPR1	High	Peak Found
5110647921577960000	O95139	251704.7	131370.8	251704.7	71336.88	10	1	1	1	128	9.63	2.17	1	3.764	0.001	NDUFB6	High	Peak Found
-4914593963434680000	Q6P3X3	1254011	654475.8	1254011	355393.1	2	2	2	2	843	5.59	0	2	3.531	0.002	TTC27	High	Peak Found
1198198230857350000	O43169	7773145	4049168	7773145	2198777	31	4	12	4	150	4.97	20.67	4	24.223	0		High	High

1749149961980630000	P27361	2190673	1140996	2190673	619583.3	15	5	9	3	379	6.74	4.84	5	16.21	0	MAPK3	High	Peak Found
4578961420069990000	P31930	6002687	3120186	6002687	1694322	20	7	13	7	480	6.37	23.15	7	36.428	0	UQCRC1	High	High
5730773249092850000	P04216	8886842	4613815	8886842	2505392	16	2	4	2	161	8.73	7.68	2	8.89	0	THY1	High	High
-3113703590403150000	Q96EP5	2717665	1410891	2717665	766141.4	8	2	6	2	407	8.56	9.21	2	13.11	0	DAZAP1	High	High
9037349892504840000	O00764	2900306	1505063	2900306	817278.7	15	5	6	5	312	6.13	3.96	5	16.329	0	PDXK	High	High
6281324760537670000	O14495	1681647	872133.8	1681647	473585.6	15	4	7	4	311	9.14	2.13	4	12.871	0	PPAP2B; PLPP3	High	High
-3208333928556770000	Q9NW15	3242504	1678333	3242504	911367.3	7	6	10	6	660	7.4	3.35	6	16.669	0	ANO10	High	High

-4468909642596950000	Q15020	4136222	2132250	4136222	1157853	7	6	7	6	963	5.57	13.02	6	19.24	0	SART3	High	High
3348063048302490000	P50851	2216815	1141366	2216815	619783.7	1	2	2	2	2863	5.6	2.06	2	4.839	0	LRBA	Peak Found	High
2594801350968760000	Q99986	689134.9	354341.2	689134.9	192414.1	6	3	3	3	396	8.91	1.72	3	6.391	0	VRK1	High	Peak Found
9033657698734280000	Q9UQ16	2973156	1528449	2973156	829977.4	6	7	11	1	869	8.35	10.43	7	18.375	0	DNM3	High	Peak Found
984760743341076000	Q9Y547	2168177	1112874	2168177	604312.2	36	3	5	3	144	5.03	7.44	3	25.137	0	HSPB11	High	High
-7187438447843370000	Q14435	735565.5	377177.4	735565.5	204814.6	4	2	4	2	633	7.99	3.83	2	7.694	0	GALNT3	High	Peak Found
8219779968186530000	Q9UI26	1788660	916977.8	1788660	497936.8	5	5	9	5	975	5.25	5.44	5	16.444	0	IPO11	High	High
526636027818	Q9HB07	1530632	784150.3	1530632	425808.9	12	3	3	3	376	6.77	2.06	3	13.256	0		High	Peak Found

9720000																		
1322674044200340000	Q12789	363888.1	185989.8	363888.1	100996.1	1	1	1	1	2109	7.3	0	1	2.215	0.009	GTF3C1	High	Peak Found
-7431042407162150000	Q6UW68	3319432	1695609	3319432	920748.5	16	2	5	2	189	8.62	4.23	2	10.768	0	TMEM205	High	High
-3246576723643330000	Q8N668	470099.6	240063.2	470099.6	130359	7	1	1	1	190	6.2	2.17	1	4.501	0	COMMD1	High	Peak Found
-407639025915272000	Q969P5	6544261	3337694	6544261	1812433	6	1	1	1	355	9.36	0	1	2.075	0.01	FBXO32	High	Peak Found
8335740267375580000	P09132	493741.1	251408.2	493741.1	136519.5	20	2	4	2	144	9.85	9.56	2	14.269	0	SRP19	High	High
3646138308514800000	Q9Y6C9	1372483	697425.7	1372483	378715.7	7	2	2	2	303	7.97	1.77	2	4.515	0	MTCH2	High	Peak Found
-337196153993	Q5VZK9	889851.1	452121.4	889851.1	245510.7	1	1	1	1	1371	7.85	0	1	3.397	0.002	LRRC16A; CARMIL1	High	Peak Found

8120000																		
-377515032386624000	P78346	763698.8	387960.1	763698.8	210669.9	6	2	2	2	268	8.91	0	2	4.333	0	RPP30	High	Peak Found
-9179155935409940000	Q15750	624873.9	316350.7	624873.9	171784.6	2	1	2	1	504	5.52	0	1	3.811	0.001	TAB1	High	High
-3476524128370900000	P53634	5846451	2955035	5846451	1604642	8	5	18	5	463	6.99	16.55	5	15.688	0	CTSC	High	High
6507340278371620000	Q9NYL4	4933477	2477479	4933477	1345319	14	3	4	3	201	9.39	3.47	3	10.266	0	FKBP11	High	High
-767357545140157000	P51784	144757.5	72682	144757.5	39467.74	2	2	3	1	963	5.45	1.96	2	4.854	0	USP11	High	Peak Found
-5820221568948660000	P61081	7261644	3643234	7261644	1978347	27	6	14	6	183	7.69	13.09	6	16.899	0	UBE2M	High	High
607951343933	Q9UGV2	956622.1	479675.3	956622.1	260473	7	2	2	2	375	5.31	2	2	6.216	0	NDRG3	High	Peak Found

5480000																		
4764020307768200000	P61586	35089723	17582708	35089723	9547752	46	9	35	3	193	6.1	38.86	9	42.163	0	RHOA	High	High
-4143266038878470000	P26006	3054877	1529686	3054877	830649.2	6	6	13	6	1051	6.77	8.02	6	17.132	0	ITGA3	High	High
3586034237824910000	P11117	821588.7	411044.7	821588.7	223205.3	4	2	3	2	423	6.74	1.61	2	5.475	0	ACP2	High	Peak Found
6302515266357760000	Q9NR46	227946.5	113974.4	227946.5	61890.29	4	2	2	1	395	5.99	1.78	2	5.44	0	SH3GLB2	High	Peak Found
-4653311583040180000	Q9H2P9	472687.7	236171.1	472687.7	128245.5	3	1	1	1	285	5.31	0	1	2.137	0.01	DPH5	High	Peak Found
-5564485370032930000	Q9UBV8	5107930	2551375	5107930	1385446	10	4	5	4	284	6.54	4.49	4	9.15	0	PEF1	High	High
864453171457	Q8WVM0	164105.4	81947.92	164105.4	44499.32	4	1	1	1	346	9.26	0	1	3.763	0.001	TFB1M	High	Peak Found

0170000																		
-9119671646432560000	P13929	1605709	800056.3	1605709	434446.1	12	4	17	1	434	7.71	26.55	4	38.639	0	ENO3	High	High
3508396415838450000	Q5JXB2	4312690	2143928	4312690	1164195	24	4	9	4	153	5.92	4.29	4	12.299	0	UBE2NL	High	High
-8001945499400240000	Q99519	2025039	1006102	2025039	546333	8	3	4	3	415	5.88	8.47	3	11.013	0	NEU1	High	High
-2602489287625280000	P62330	7063785	3503501	7063785	1902469	25	4	14	3	175	8.95	20.31	4	24.159	0	ARF6	High	High
-1938619972574950000	Q9ULW0	902433.4	447268.6	902433.4	242875.6	5	3	4	3	747	9.23	1.8	3	7.339	0	TPX2	High	High
-1773607396615150000	Q16774	831787.9	411435.3	831787.9	223417.4	6	1	1	1	197	6.55	0	1	2.34	0.008	GUK1	Peak Found	High

-5625038226073760000	P61421	795939.9	393156.9	795939.9	213491.9	7	2	4	2	351	5	4.1	2	5.457	0	ATP6V0D1	High	High
-3949732143901390000	O43427	300285.3	148176.1	300285.3	80462.48	3	1	1	1	364	6.48	0	1	3.173	0.003	FIBP	High	Peak Found
-4658172871140460000	Q9P2B4	374123.8	184405	374123.8	100135.5	5	3	3	3	639	8.06	0	3	5.504	0	CTTNBP2NL	High	High
978357741075337000	Q6UN15	344637.2	169526.9	344637.2	92056.41	3	2	3	2	594	5.59	2.21	2	8.464	0	FIP1L1	High	High
-1594484432885070000	O75663	2075101	1020553	2075101	554180.1	20	4	5	4	272	5.91	6.17	4	12.868	0	TIPRL	High	High
-2714329419910620000	P47985	979433.6	481497.5	979433.6	261462.5	14	3	6	3	274	8.32	0	3	7.106	0	UQCRFS1	High	High
5797798922783160000	Q2TAY7	3152574	1545987	3152574	839501	11	5	8	5	513	7.18	4.09	5	18.895	0	SMU1	High	High

5664998384620820000	Q13895	1095944	537386.3	1095944	291811.2	15	5	7	5	437	8.12	4.09	5	20.032	0	BYSL	High	High
-2273417600798460000	Q10570	714027.2	349800.2	714027.2	189948.3	2	3	3	3	1443	6.4	1.6	3	7.811	0	CPSF1	High	Peak Found
-4833705581656820000	Q96RT1	620386.8	303911.8	620386.8	165030	1	2	2	2	1412	5.5	0	2	4.068	0	ERBB2IP; ERBIN	High	Peak Found
-253239951813173000	Q9H2M9	1803271	882638.1	1803271	479289.7	3	4	12	4	1393	5.62	6.84	4	16.201	0	RAB3GAP2	High	High
-7497019614434810000	Q9NQH7	26314216	12869504	26314216	6988391	3	2	4	2	507	6.83	3.9	2	3.991	0	XPNPEP3	High	High
8260610778738380000	Q13907	3405620	1661493	3405620	902223.1	19	5	10	5	227	6.34	10.71	5	19.823	0	IDI1	High	High
-6769080269671980000	Q9NVP1	993440.3	484497.8	993440.3	263091.7	5	3	4	3	670	9.5	2	3	6.477	0	DDX18	High	High

-1047543099056460000	P09104	3954104	1927163	3954104	1046487	21	8	18	6	434	5.03	30.82	8	44.944	0	ENO2	High	High
-2064398730154580000	Q15061	891220.7	433970.7	891220.7	235654.5	2	2	2	2	677	5.57	1.64	2	5.531	0	WDR43	High	Peak Found
5373690399274410000	Q8N543	520128.9	252345	520128.9	137028.2	3	1	2	1	542	5.11	2.91	1	7.983	0	OGFOD1	High	Peak Found
-5604354164667520000	Q13769	600349	291225.7	600349	158141.2	5	3	3	3	683	6.87	1.67	3	10.435	0	THOC5	High	Peak Found
-1393255483118750000	Q9H2U2	1320821	640382.8	1320821	347740.3	11	3	10	2	334	7.39	6.28	3	11.358	0	PPA2	High	High
-1970513305898250000	Q9H2D6	3949213	1912227	3949213	1038377	3	7	9	7	2365	8.48	11.18	7	22.124	0	TRIOBP	High	High
120024261784	P31749	1381910	667990.5	1381910	362731.8	7	3	4	2	480	6.07	3.86	3	13.192	0	AKT1	High	High

8960000																		
2687801448742430000	Q9H4A6	1212298	585764	1212298	318081.2	5	1	1	1	298	6.44	2.31	1	5.837	0	GOLPH3	Peak Found	High
2762375354567500000	Q9UI14	325962.9	157422.6	325962.9	85483.54	8	1	2	1	185	7.34	0	1	2.082	0.01	RABAC1	High	Peak Found
2107690767117560000	P04150	369106.5	178170.8	369106.5	96750.2	1	1	1	1	777	6.38	2.05	1	2.363	0.008	NR3C1	High	Peak Found
-5767987325400280000	Q14728	828934.1	400080.9	828934.1	217251.7	7	3	3	3	455	9.6	0	3	6.449	0	MFSD10	High	Peak Found
2169446649926960000	Q9NYI0	174850.3	84324.1	174850.3	45789.63	2	2	2	2	1048	5.99	1.99	2	6.595	0	PSD3	High	High
-5453029631849900000	Q9NRN7	1984554	956386.4	1984554	519336.4	10	3	5	3	309	6.8	9.73	3	12.345	0	AASDHPPT	High	High
-864712874356	P78417	18987151	9123977	18987151	4954497	39	12	30	12	241	6.6	35.18	12	50.412	0	GSTO1	High	High

1790000																		
-34001002036977300	O76003	3905107	1875673	3905107	1018527	12	4	7	4	335	5.39	9.41	4	15.15	0	GLRX3	High	High
-3343567279109480000	P61326	2991138	1434504	2991138	778963.6	21	3	5	3	146	6.11	7.7	3	10.263	0	MAGOH	High	High
1517122428760550000	Q9C0G6	1587503	759109.3	1587503	412211.1	0	1	3	1	4158	6	2.1	1	4.36	0	DNAH6	High	High
-1669243048092860000	Q8WU90	508064.9	242937.4	508064.9	131919.7	7	2	2	2	426	5.31	0	2	6.65	0	ZC3H15	High	Peak Found
1703725255333180000	O14530	647590.2	308328.1	647590.2	167428.2	4	1	1	1	226	5.88	0	1	3.462	0.002	TXNDC9	High	Peak Found
6257292579144490000	Q92544	1718648	815232.5	1718648	442687.1	2	2	3	2	642	6.54	0	2	3.461	0.002	TM9SF4	High	High
1440931390251670000	Q96AA3	1146823	543167.6	1146823	294950.6	4	2	2	2	541	8.85	2.22	2	4.599	0	RFT1	High	Peak Found

1217217274349290000	Q9Y4E8	2422963	1147421	2422963	623072.2	7	7	11	6	981	5.22	6.08	7	17.267	0	USP15	High	High
-3318017659961930000	Q9NVA1	85485.58	40412.45	85485.58	21944.75	3	1	1	1	299	8.91	0	1	2.302	0.008	UQCC; UQCC1	High	Peak Found
-2974134927255500000	Q6VY07	925638.7	436023.7	925638.7	236769.4	4	3	3	3	963	7.74	2.38	3	10.255	0	PACS1	High	Peak Found
4375346843613750000	O94901	4017517	1892255	4017517	1027531	9	7	10	7	812	7.08	11.09	7	17.815	0	SUN1	High	High
8425175822669480000	Q9NRY5	251748.8	118404.1	251748.8	64295.71	2	1	1	1	505	4.88	2.03	1	2.688	0.005	FAM114A2	High	Peak Found
-5138409153076520000	Q9HBI1	1974941	927916.7	1974941	503876.8	12	4	9	3	364	6.73	8.76	4	15.424	0	PARVB	High	High
1450891647387080000	Q9BUL8	3281472	1541650	3281472	837146.2	12	3	8	3	212	8.19	4.2	3	8.469	0	PDCD10	High	High

1058361017873510000	P63173	4378079	2050879	4378079	1113667	33	3	9	3	70	10.1	11.06	3	17.598	0	RPL38	High	High
-1462166031620210000	Q12882	483001.7	226256.4	483001.7	122861.6	3	3	4	3	1025	7.05	0	3	6.606	0	DPYD	High	Peak Found
6653368877228170000	Q9NRX2	967443.9	451930.1	967443.9	245406.8	10	2	2	2	175	10.11	1.65	2	3.113	0.003	MRPL17	High	Peak Found
-6644865826182150000	Q6DKJ4	2811627	1313099	2811627	713038.4	14	4	9	4	435	4.97	14.23	4	25.52	0	NXN	High	High
7500811780682680000	Q8N8S7	3795246	1771157	3795246	961772.6	7	4	10	4	591	6.93	8.08	4	18.083	0	ENAH	High	High
3054856418516560000	Q8IZ07	297733.6	138638.5	297733.6	75283.38	2	1	1	1	590	5.02	0	1	3.355	0.002	ANKRD13A	High	Peak Found
775419555860448000	Q5J8M3	135685.5	63140.3	135685.5	34286.41	5	1	1	1	183	8.62	0	1	2.824	0.004	EMC4	High	Peak Found
169516752189	P53609	1083117	500358.5	1083117	271704.4	7	3	3	3	377	6.83	3.49	3	7.271	0	PGGT1B	High	Peak Found

7820000																		
-3817415269467870000	P16435	4679200	2161568	4679200	1173774	7	5	11	5	677	5.58	15.15	5	17.999	0	POR	High	High
-8390687985957490000	Q5VTR2	804106.6	370762.3	804106.6	201331.1	4	3	3	3	975	5.94	1.8	3	10.387	0	RNF20	High	Peak Found
-1655669329646530000	Q92643	854159.5	393585	854159.5	213724.3	8	3	6	3	395	6.16	1.85	3	6.831	0	PIGK	High	High
-1377000983824550000	Q8N5N7	271730.6	125125.8	271730.6	67945.74	7	1	1	1	158	7.88	0	1	2.399	0.008	MRPL50	High	Peak Found
-401785042072942000	O94808	16974746	7792294	16974746	4231367	8	6	19	4	682	7.37	9.96	6	15.635	0	GFPT2	High	High
-1100607226643900000	Q9UHR4	791042	362542.1	791042	196867.4	2	1	1	1	511	8.68	0	1	2.593	0.006	BAIAP2L1	High	Peak Found

-3037731205086650000	P09234	3060601	1401963	3060601	761293.3	25	3	5	3	159	9.67	8.13	3	11.261	0	SNRPC	High	High
4230777369959250000	O95239	5925089	2707557	5925089	1470256	9	11	16	11	1232	6.27	7.49	11	28.893	0	KIF4A	High	High
-6620262880976820000	Q15629	12244104	5564946	12244104	3021874	11	5	15	5	374	9.63	11.28	5	25.286	0	TRAM1	High	High
7478830788962870000	Q9Y3C4	488520.5	221643.5	488520.5	120356.7	4	1	1	1	175	6.79	0	1	3.071	0.003	TPRKB	High	Peak Found
2357277482665960000	P08134	1698164	770205.8	1698164	418236.8	39	8	31	2	193	6.58	30.95	8	35.416	0	RHOC	High	High
-9128345059399950000	P83436	123735.5	55707.95	123735.5	30250.5	1	1	1	1	770	5.47	0	1	2.619	0.006	COG7	High	Peak Found
-6767865713948380000	P49720	5006422	2251750	5006422	1222744	16	4	7	4	205	6.55	10.73	4	13.815	0	PSMB3	High	High

6271968905262140000	Q4VC31	1224337	549341.8	1224337	298303.3	21	3	4	3	144	7.81	3.39	3	6.967	0	CCDC58	High	Peak Found
-1124967282390940000	Q9HB90	2058410	922477.8	2058410	500923.4	12	4	5	4	399	5.1	1.93	4	17.399	0	RRAGC	High	High
-3006395004919520000	Q9BUP3	526032.6	234525.5	526032.6	127351.9	4	1	1	1	242	8.38	1.81	1	3.031	0.003	HTATIP2	High	Peak Found
-7144530855907260000	Q9H6R4	937272.5	416620.7	937272.5	226233.2	2	2	2	2	1146	7.64	1.7	2	5.201	0	NOL6	High	Peak Found
8248535501308070000	P62879	3526057	1566258	3526057	850508.7	24	8	26	1	340	6	36.27	8	36.856	0	GNB2	Peak Found	High
8565814559741760000	Q16566	162950.1	72200.97	162950.1	39206.53	6	2	3	1	473	5.82	0	2	5.292	0	CAMK4	High	Peak Found
-3665297553564580000	P51884	5563128	2460491	5563128	1336094	11	4	10	4	338	6.61	18.57	4	19.117	0	LUM	High	High

-6935074334363070000	O00762	374283.9	165279.9	374283.9	89750.17	4	1	1	1	179	7.37	0	1	2.124	0.01	UBE2C	High	Peak Found
4807008432777760000	Q9NTZ6	4153513	1832030	4153513	994827.9	7	7	11	7	932	8.63	9.8	7	20.58	0	RBM12	High	High
-3738159445275180000	Q96SL4	3186607	1402240	3186607	761443.6	17	4	4	4	187	8.27	3.75	4	7.876	0	GPX7	High	High
-6091940501454680000	Q9UNX4	1251756	550606.1	1251756	298989.8	3	4	5	4	943	6.64	3.48	4	9.211	0	WDR3	High	Peak Found
-8769135798573240000	P35658	1813477	797593.3	1813477	433108.7	2	4	4	4	2090	7.47	2.05	4	9.492	0	NUP214	High	Peak Found
-4229859227879040000	P16278	880212.3	386552.3	880212.3	209905.4	3	2	3	2	677	6.57	1.72	2	6.43	0	GLB1	High	Peak Found
298604944825	Q8N3U4	1607139	704974.7	1607139	382815	4	5	8	5	12	5.43	2.11	5	13.245	0	STAG2	High	High

2160000										31								
-7848338417616040000	P35270	977040.3	427858	977040.3	232335.2	18	4	5	4	261	8.05	0	4	8.612	0	SPR	High	High
7158651141849580000	Q9Y294	438579.9	191705.7	438579.9	104099.9	5	1	1	1	204	4.41	1.8	1	4.132	0	ASF1A	High	Peak Found
-3076741616427270000	Q8TDN6	3627915	1584941	3627915	860653.9	16	4	7	4	353	9.92	2.67	4	10.247	0	BRIX1	High	High
8329316207491270000	Q9H330	547784.5	239191.7	547784.5	129885.7	4	2	3	2	879	8.91	0	2	3.661	0.001		High	Peak Found
-1583280322088080000	Q9NPF4	1433487	625002.8	1433487	339388.7	3	1	2	1	335	6.35	0	1	2.772	0.004	OSGEP	High	Peak Found
-3045251361027410000	O43237	4134861	1802118	4134861	978585.2	17	6	9	6	492	6.38	12.03	6	23.272	0	DYNC1LI2	High	High

8387303708123160000	P07602	6813753	2958840	6813753	1606708	8	4	10	4	524	5.17	6.01	4	14.553	0	PSAP	High	High
6753985911223900000	P68032	1.98E+08	85921957	1.98E+08	46657293	44	23	240	6	377	5.39	345.21	23	181.658	0	ACTC1	High	High
9101384525569950000	Q9BZF1	400820.8	173836.5	400820.8	94396.59	6	3	5	3	889	6.96	1.72	3	8.565	0	OSBPL8	High	High
9134922039646340000	Q8NBF2	3234471	1399704	3234471	760066.4	5	5	8	5	726	5.55	5.73	5	11.339	0	NHLRC2	High	High
5833035186099410000	Q96KR1	1125465	485508.2	1125465	263640.4	4	3	4	3	1074	9.04	0	3	5.952	0	ZFR	High	Peak Found
-4278249940828630000	P32321	1145009	493328.2	1145009	267886.8	11	2	2	2	178	7.56	3.31	2	5.721	0	DCTD	High	Peak Found
-644721245290312000	Q9UNS2	5192307	2223130	5192307	1207203	9	4	7	4	423	6.65	7.42	4	12.837	0	COPS3	High	High
159200982606	Q6P1A2	1459931	623428.7	1459931	338533.9	7	3	3	3	487	8.69	5.13	3	8.831	0	LPCAT3	High	Peak Found

3750000																		
6807117171937690000	Q9BWM7	4395075	1876167	4395075	1018795	25	7	21	6	321	9.1	18.22	7	34.769	0	SFXN3	High	High
-4149430532130190000	Q13057	2648531	1128505	2648531	612800.3	6	3	10	3	564	6.99	8.35	3	9.344	0	COASY	High	High
2041332710814850000	Q9NRY4	3321287	1412322	3321287	766918.3	7	10	11	10	1499	6.64	1.87	10	25.24	0	ARHGAP35	High	High
4773428691670050000	P62834	727802.7	308632.2	727802.7	167593.3	28	6	19	1	184	6.67	29.97	6	23.975	0	RAP1A	High	Peak Found
-4877503792566380000	O75822	4933780	2087407	4933780	1133502	23	6	13	6	258	4.83	6.22	6	17.68	0	EIF3J	High	High
980650416735823000	Q9UHD1	3071947	1297622	3071947	704634.2	20	5	8	5	332	7.87	8.12	5	23.099	0	CHORDC1	High	High
7973813700341610000	Q96SB4	2731777	1153673	2731777	626467.1	5	3	5	2	655	6.16	1.98	3	9.446	0	SRPK1	High	High

-3867715510256540000	P49757	688958.3	289456.5	688958.3	157180.5	3	2	4	1	651	8.51	1.88	2	6.037	0	NUMB	High	Not Found
8760192209186550000	Q8NCA5	3435003	1438765	3435003	781277.3	21	7	13	5	518	8.95	11.66	7	25.124	0		High	High
1908270249902800000	P35573	3456579	1435427	3456579	779464.5	7	9	12	9	1532	6.76	2.27	9	25.489	0	AGL	High	High
-6588464217400970000	Q10471	4778945	1976996	4778945	1073547	15	8	16	8	571	8.35	9.49	8	26.658	0	GALNT2	High	High
-7333180136300150000	Q96S44	1752820	724920.1	1752820	393645.7	19	4	4	4	253	9.54	0	4	11.832	0	TP53RK	High	Peak Found
1800705943100210000	P54852	1993600	819287.4	1993600	444889	5	1	3	1	163	7.96	0	1	2.128	0.01	EMP3	High	Peak Found
-5381687560641580000	Q8TAD4	147119	60062.7	147119	32615.21	1	1	2	1	765	7.31	0	1	2.899	0.004	SLC30A5	High	Peak Found

-6193129291555450000	O43761	882320.4	358016.1	882320.4	194409.7	12	2	2	2	229	8.18	0	2	7.244	0	SYNGR3	High	Peak Found
-5281205603675220000	Q9NX76	867798.1	348579.6	867798.1	189285.5	6	1	4	1	183	5.29	4.22	1	4.473	0	CMTM6	High	High
718795190937465000	Q9Y6V7	535729.4	214934.9	535729.4	116713.8	4	2	2	2	483	9.06	0	2	5.702	0	DDX49	High	Peak Found
-7550223946587700000	Q13232	1570232	629652.6	1570232	341913.6	15	2	3	2	169	7.84	4.81	2	11.154	0	NME3	High	Peak Found
-8775799832689220000	Q8IV38	533677.2	213910.1	533677.2	116157.3	2	1	1	1	441	6.25	0	1	2.267	0.009	ANKMY2	High	Peak Found
-8621700409293050000	P23368	4032480	1616183	4032480	877618.7	14	8	12	8	584	7.61	15.2	8	27.623	0	ME2	High	High
3988641573535560000	Q9BTX1	2284884	898771.7	2284884	488050.5	6	4	8	4	674	9.09	7.26	4	10.71	0	NDC1; TMEM48	High	High

-779111087231144000	Q8N4V1	1019148	398217.9	1019148	216240.1	27	2	3	2	131	9.16	5.44	2	9.408	0	MMGT1	High	High
-2187506178089570000	Q8WXF7	540369.5	211125.1	540369.5	114645	2	1	1	1	558	6.18	1.97	1	2.931	0.004	ATL1	High	Peak Found
7161908197236870000	Q9BTV5	383558.1	148931.8	383558.1	80872.86	5	2	2	2	496	6.99	0	2	5.435	0	FSD1	High	Peak Found
-1084161266970480000	Q04206	3580222	1386374	3580222	752828.2	17	8	14	8	551	5.68	14.23	8	22.845	0	RELA	High	High
8134550695814120000	P30519	705593.8	273165.5	705593.8	148334.2	6	1	2	1	316	5.41	6.27	1	14.237	0	HMOX2	High	Peak Found
7420075580370480000	P14209	386822.4	149716.8	386822.4	81299.13	5	1	2	1	185	4.75	2.08	1	2.153	0.009	CD99	High	High
-3101677286908010000	Q9Y450	1310543	503482.5	1310543	273400.8	4	2	2	2	684	6.61	3.47	2	6.551	0	HBS1L	High	High

276241169312228000	O94851	950470.3	363374	950470.3	197319.2	3	3	3	3	1124	8.65	3.98	3	10.802	0	MICAL2	High	Peak Found
-2947777995853760000	Q53GQ0	6979430	2663031	6979430	1446078	12	3	8	3	312	9.32	4.25	3	11.502	0	HSD17B12	High	High
6930052097087830000	Q15003	1213884	462663.2	1213884	251235.1	3	2	2	2	741	5.06	2.4	2	7.058	0	NCAPH	High	Peak Found
5956791852298740000	Q9UPY8	519896.5	198140.5	519896.5	107594.1	6	2	5	1	281	5.54	5.39	2	6.755	0	MAPRE3	High	Peak Found
335007911528985000	Q9P0I2	844623.9	321472.5	844623.9	174565.8	9	2	2	2	261	6.81	0	2	4.175	0	EMC3	High	Peak Found
862030187180184000	O60238	632155.2	239378.6	632155.2	129987.2	5	1	1	1	219	5.85	1.96	1	3.101	0.003	BNIP3L	High	Peak Found
3818146926309850000	O95671	2481997	938904.5	2481997	509843.4	11	5	6	5	621	6.07	8.69	5	20.043	0	ASMTL	High	High
-4758086446517530000	P62316	7368361	2786390	7368361	1513064	33	4	6	4	118	9.91	12.88	4	11.61	0	SNRPD2	High	Peak Found

6364343737777030000	P08754	3065976	1159291	3065976	629517.8	30	9	25	4	354	5.69	31.03	9	29.893	0	GNAI3	High	High
-4000962258629930000	P62847	7161784	2704598	7161784	1468649	9	2	20	2	133	10.78	24.6	2	18.271	0	RPS24	High	High
-2971731893176670000	O15305	1445549	544154.6	1445549	295486.5	14	4	5	4	246	6.77	1.66	4	12.557	0	PMM2	High	High
1378614036355870000	Q9UIV1	222994.4	83844.76	222994.4	45529.34	4	1	1	1	285	4.84	0	1	3.542	0.002	CNOT7	High	Peak Found
-8439683798465210000	P08621	4053435	1523443	4053435	827259.1	17	6	8	6	437	9.94	9.79	6	18.406	0	SNRNP70	High	High
-5738973645522840000	Q5MNZ6	270386.4	101419.1	270386.4	55072.52	4	1	2	1	344	7.59	2.02	1	6.777	0	WDR45B; WDR45L	High	Peak Found
-911938199932515000	Q9NZQ3	1318754	493029.8	1318754	267724.8	5	4	4	4	722	6.38	1.87	4	10.505	0	NCKIPSD	High	Peak Found

8697682766688930000	Q9GZZ1	9488128	3541522	9488128	1923115	25	5	9	5	169	8.81	4.5	5	13.423	0	NAA50	High	High
-6052535811300550000	Q2NL82	777656.6	289697.1	777656.6	157311.1	3	2	4	2	804	7.42	4.9	2	10.131	0	TSR1	High	High
8229141518412530000	P68036	10143363	3777280	10143363	2051136	32	5	10	5	154	8.51	9.69	5	18.19	0	UBE2L3	High	High
3418914028943410000	P29144	1292494	481188.7	1292494	261294.8	3	4	5	4	1249	6.32	0	4	8.894	0	TPP2	High	Peak Found
5708958418174030000	P08574	3733633	1390002	3733633	754797.8	12	3	5	3	325	9	7.61	3	14.214	0	CYC1	High	High
1287590605065330000	Q8WVX9	1512015	562009.4	1512015	305182	8	4	4	4	515	9.17	1.62	4	11.508	0	FAR1	High	Peak Found
-5490413544944080000	Q9BPX5	5480466	2024959	5480466	1099592	29	3	3	3	153	6.6	2.29	3	11.443	0	ARPC5L	High	High

-2271867218298690000	Q96K37	269712.5	99573.2	269712.5	54070.18	2	1	2	1	410	9.79	1.9	1	3.242	0.002	SLC35E1	High	Peak Found
7367615421387890000	Q9Y5X3	1755530	645941.4	1755530	350758.7	12	5	8	4	404	6.76	5.97	5	15.869	0	SNX5	High	High
-8474719942598480000	Q8WYA6	2433518	889453.4	2433518	482990.5	9	5	8	5	563	5.05	0	5	14.865	0	CTNNBL1	High	High
2096625671163080000	O95999	339741.7	123501.2	339741.7	67063.56	4	1	1	1	233	5.74	1.62	1	3.205	0.002	BCL10	High	Peak Found
-8397270974056060000	Q9NRL3	446674	161972.7	446674	87954.3	5	3	3	2	753	5.4	3.79	3	8.395	0	STRN4	High	Peak Found
3124234521907950000	Q9NX24	1745643	632113.1	1745643	343249.7	19	2	2	2	153	8.22	4.92	2	9.453	0	NHP2	High	Peak Found
3559097466797420000	Q8TDW0	525152.9	189558.5	525152.9	102934	3	2	2	1	803	7.62	1.75	2	6.343	0	LRRC8C	High	Peak Found

-8476150003951290000	Q96KG9	7152117	2573122	7152117	1397255	12	7	13	7	808	6.3	12.28	7	27.458	0	SCYL1	High	High
-8102518268723220000	Q2M2I8	1764636	631130.7	1764636	342716.2	3	3	4	3	961	6.6	6.62	3	8.037	0	AAK1	High	High
1412779903324820000	Q8TF42	596005.6	212580.7	596005.6	115435.5	7	3	3	3	649	6.93	3.87	3	11.33	0	UBASH3B	High	Peak Found
-5620722506811580000	Q96GG9	1420539	505833.9	1420539	274677.7	12	3	4	3	259	5.34	0	3	8.021	0	DCUN1D1	High	High
7175023148943120000	Q8NEU8	740521	263469.7	740521	143069.2	3	2	3	2	664	4.94	1.93	2	6.498	0	APPL2	High	High
8199308046116630000	Q29RF7	1680251	595639.3	1680251	323443.7	3	4	5	4	1337	7.91	6.07	4	11.57	0	PDS5A	High	Peak Found
-6441918618070780000	P51610	3889088	1376124	3889088	747262.1	3	6	8	6	2035	7.46	5.83	6	19.523	0	HCFC1	High	High

-8413974576400380000	Q9NQT5	160725.4	56803.3	160725.4	30845.29	7	1	1	1	275	8.1	0	1	2.417	0.008	EXOSC3	High	Peak Found
3200038646673230000	Q9P287	1522547	535954.3	1522547	291033.6	10	3	3	3	314	4.61	1.74	3	8.069	0	BCCIP	High	Peak Found
-4828769571271310000	P46108	2898310	1017846	2898310	552710	17	3	4	3	304	5.55	2.89	3	14.856	0	CRK	High	High
3387363909375920000	Q0ZGT2	7667383	2687266	7667383	1459237	10	7	11	7	675	5.33	5.96	7	16.536	0	NEXN	High	High
-7501942323531490000	P43363	386954.9	135086.9	386954.9	73354.83	2	1	1	1	369	4.4	2.11	1	2.7	0.005	MAGEA10	High	Peak Found
9162404171034650000	Q9Y223	3196518	1115574	3196518	605778.5	8	5	8	5	722	6.8	10.26	5	19.836	0	GNE	High	High
-8913218516764720000	P34810	762059.9	265666.5	762059.9	144262.1	6	1	1	1	354	8.87	3.56	1	9.107	0	CD68	High	Peak Found

2529735565303520000	Q9BQS8	622372.6	215570.9	622372.6	117059.2	3	4	6	4	1478	4.92	4.34	4	14.996	0	FYCO1	High	Peak Found
7065461624018500000	Q96JB5	2231536	771090.3	2231536	418717	11	5	8	5	506	4.75	8.69	5	12.465	0	CDK5RAP3	High	High
-3152037375536320000	P68402	2596834	897136.7	2596834	487162.7	4	1	1	1	229	5.92	2.98	1	4.182	0	PAFAH1B2	High	Peak Found
-9154199525280400000	Q9UH17	1272858	439730.8	1272858	238782.4	8	2	3	2	382	6.06	5.19	2	15.154	0	APOBEC3B	High	Peak Found
6391839617332390000	P07203	463757.1	159732.7	463757.1	86737.96	10	2	2	2	203	6.55	1.65	2	5.103	0	GPX1	High	Peak Found
4305466471512970000	P0DMU9	1165016	397685.6	1165016	215951	9	2	2	2	189	9.55	0	2	3.122	0.003	LOC102723631; CT45A10; CT45A5; CT45A6; LOC102723680; CT45A9	High	High

-9216601272429100000	Q96GX9	528584.4	180022	528584.4	97755.45	13	2	3	2	242	7.12	2.55	2	8.777	0	APIP	High	High
-2468696703933970000	P23921	4020936	1357745	4020936	737281.8	8	6	7	6	792	7.15	7.41	6	20.216	0	RRM1	High	High
-4410933930265080000	P14927	188333.6	63569.98	188333.6	34519.73	18	1	2	1	111	8.78	2.17	1	4.278	0	UQCRB	High	Peak Found
-3696003956751780000	P37235	11931650	4023912	11931650	2185062	44	7	13	7	193	5.35	24.57	7	35.295	0	HPCAL1	High	High
-4002494153914850000	P51665	12398320	4139263	12398320	2247700	20	7	12	7	324	6.77	15.95	7	25.047	0	PSMD7	High	High
-7548254055495860000	Q9BZD4	297812	99194.84	297812	53864.73	2	1	2	1	464	8.27	0	1	2.504	0.007	NUF2	High	High
-446215	Q92922	2245002	746719.7	2245002	405483.3	5	6	12	2	11	5.76	17.96	6	22.917	0	SMARCC1	High	High

2622631020000										05							
-8613494787005000000	Q9BWJ5	904839.5	299110.5	904839.5	162422.8	26	2	2	2	86	6.35	2.08	2	7.18	0	SF3B5	High	Peak Found
-7378124284340210000	Q8NBI6	349435.7	113312.9	349435.7	61531.12	7	2	2	2	393	8.13	0	2	3.549	0.002	XXYLT1	High	Peak Found
394232053154577000	Q92530	703161.1	227909.4	703161.1	123759.3	7	2	4	2	271	5.74	5.38	2	5.692	0	PSMF1	High	Peak Found
5201863361074030000	O95071	756542.2	245134.2	756542.2	133112.6	2	4	4	4	2799	5.85	1.62	4	8.818	0	UBR5	High	Peak Found
660513057168003000	Q99805	2622928	847761	2622928	460350.7	8	4	7	4	663	7.44	6.19	4	12.366	0	TM9SF2	High	High
-6100436114582120000	Q93034	3137575	1010597	3137575	548773.9	7	5	9	5	780	7.94	7.47	5	13.738	0	CUL5	High	High
7145553044462340000	Q8NI36	955060.8	303331.3	955060.8	164714.8	4	4	5	4	951	7.53	3.24	4	8.661	0	WDR36	High	Peak Found

7474756329522260000	O14672	538538.9	169052.1	538538.9	91798.55	3	2	3	2	748	7.77	2.39	2	6.689	0	ADAM10	High	Peak Found
9041443353459390000	Q8N9N7	260717.6	81742.91	260717.6	44387.99	5	1	1	1	239	8.43	0	1	2.55	0.006	LRRC57	High	Peak Found
8976647445463940000	O43505	798010.1	245512.6	798010.1	133318.1	6	2	4	2	415	7.2	1.65	2	6.792	0	B3GNT1; B4GAT1	High	High
4260843407477110000	Q9Y6M0	1369149	420085.7	1369149	228114.7	11	3	4	3	314	7.62	1.64	3	10.468	0	PRSS21	High	Peak Found
5062311140288300000	O75063	384766.3	117742.7	384766.3	63936.57	2	1	1	1	409	6.87	0	1	2.588	0.006	FAM20B	High	Peak Found
-2662746858814380000	Q15024	1829818	556621.2	1829818	302256.1	13	3	6	3	291	5.19	4.23	3	14.434	0	EXOSC7	High	High
7834140751544990000	Q7Z6Z7	4564953	1378957	4564953	748800.7	2	8	13	8	4374	5.22	7.9	8	22.679	0	HUWE1	High	High
-327926567638	Q9BXB4	1608771	483968.5	1608771	262804.3	3	3	4	3	747	7.06	0	3	6.247	0	OSBPL11	High	High

6750000																		
8461791288305670000	Q92783	1118228	335658.8	1118228	182269.3	7	3	3	3	540	4.82	3.37	3	13.086	0	STAM	High	Peak Found
4007751392279350000	Q562R1	1536934	460620.3	1536934	250125.8	27	11	87	3	376	5.59	110.25	11	69.672	0	ACTBL2	High	High
6764230823219760000	Q9UJ14	305160.5	90787.55	305160.5	49299.4	2	1	1	1	662	5.1	0	1	4.128	0	GGT7	High	Peak Found
5039007955841060000	Q15397	1001639	292862	1001639	159029.8	5	3	4	3	648	9.64	0	3	7.167	0	KIAA0020; PUM3	High	Peak Found
-6403678717829710000	Q9H3K6	1228438	358886.1	1228438	194882.2	37	3	5	3	86	6.52	1.79	3	9.243	0	BOLA2B; BOLA2; LOC107984053	High	High
-3309717020774850000	Q96GQ7	722342	210764.2	722342	114449	4	3	4	3	796	9.28	1.83	3	6.966	0	DDX27	High	Peak Found
4487199997490170000	P01034	2577126	743710.3	2577126	403849.1	19	2	2	2	146	8.75	0	2	7.535	0	CST3	High	Peak Found

-2830448209821530000	Q9GZS3	3020732	864263.2	3020732	469311.7	9	3	7	3	305	5.47	0	3	9.403	0	WDR61	High	High
-3716051439609720000	Q9NW82	1309543	372288	1309543	202159.6	4	2	2	2	654	6.33	2.68	2	6.061	0	WDR70	High	High
928780824401578000	P61587	619320.3	175725.9	619320.3	95422.6	9	2	2	2	244	8.48	1.84	2	4.771	0	RND3	High	Peak Found
5016133696991680000	Q9Y3E5	1084718	306077.5	1084718	166206.1	29	3	3	3	179	8.73	2.92	3	11.221	0	PTRH2	High	Peak Found
-4662395530703890000	Q9HD15	939749.8	264993.3	939749.8	143896.5	10	2	2	2	236	7.03	3.85	2	7.506	0	SRA1	High	Peak Found
-8357658854123360000	O00629	6224288	1754706	6224288	952839.3	12	6	11	4	521	4.96	11.03	6	29.108	0	KPNA4	High	High
-7657012177295080000	O43684	45010890	12648814	45010890	6868552	30	9	17	9	328	6.84	28.7	9	41.889	0	BUB3	High	High

-7500020878555020000	Q9BZE4	1088057	298800.7	1088057	162254.6	3	2	3	2	634	9.5	1.72	2	6.255	0	GTPBP4	High	High
3786369172385550000	P19388	1347184	369323.2	1347184	200549.7	13	3	3	3	210	5.95	4.03	3	7.55	0	POLR2E	High	Peak Found
5616367941086100000	Q9NRW7	1054282	285726.2	1054282	155154.9	5	3	4	3	570	8.24	1.62	3	9.673	0	VPS45	High	Peak Found
-7952691654267690000	Q15067	1010733	269075.6	1010733	146113.3	8	3	6	3	660	8.16	5.61	3	14.966	0	ACOX1	High	High
-7694143023947030000	Q96I15	142617.1	37846.86	142617.1	20551.58	4	1	1	1	445	7.12	0	1	2.446	0.007	SCLY	High	Peak Found
8719545438223680000	Q9NWV8	997578.2	257403.3	997578.2	139775	3	1	2	1	329	4.64	2.35	1	3.407	0.002	BABAM1	High	High
-9195295084740600000	Q00577	5611732	1439169	5611732	781496.8	7	3	5	2	322	6.44	5.63	3	8.939	0	PURA	High	High

7347727386660610000	Q8IUI8	308857.7	78800.57	308857.7	42790.24	5	2	2	2	442	5.14	1.67	2	6.695	0	CRLF3	High	Peak Found
-3391234540397170000	Q9H2U1	10208860	2587943	10208860	1405303	19	15	27	15	1008	7.68	24.7	15	68.622	0	DHX36	High	High
-5795774045568990000	Q9GZR7	2621886	662745.7	2621886	359883.8	5	4	9	4	859	9.06	9.44	4	18.228	0	DDX24	High	High
-8635203665843220000	P33947	2624856	662675.9	2624856	359845.9	5	1	4	1	212	8.72	2.41	1	3.818	0.001	KDELR2	High	High
1655251497259480000	Q9BV38	1126282	278918.7	1126282	151458.3	9	4	4	4	432	6.7	2.2	4	11.583	0	WDR18	High	High
-6548479852001090000	P10253	1682642	409366.7	1682642	222294.1	6	4	4	4	952	6	4.12	4	10.369	0	GAA	High	Peak Found
676002530395001000	Q14766	700988	170516.3	700988	92593.67	3	4	5	4	1721	5.96	0	4	10.099	0	LTBP1	High	Peak Found

-7569413463716610000	Q8N6M0	3048722	740561.4	3048722	402139.2	12	3	7	3	293	6.05	8.12	3	13.63	0	OTUD6B	High	High
3729688095668830000	O75419	400573.5	96113.61	400573.5	52191.56	3	2	2	2	566	5.53	0	2	3.462	0.002	CDC45	High	Peak Found
7697367026041440000	Q96A49	2509395	600814.4	2509395	326253.9	15	4	6	4	352	4.53	6.74	4	14.27	0	SYAP1	High	High
2608297445267440000	Q96QR8	17803945	4243341	17803945	2304217	25	9	12	8	312	5.43	15.32	9	33.482	0	PURB	High	High
5875685193594640000	Q7Z3U7	1412501	327210.4	1412501	177681.6	2	4	6	4	1717	6.06	2.08	4	10.785	0	MON2	High	High
-6381027269296730000	Q7Z392	449281.2	103449.1	449281.2	56174.86	2	2	3	2	1133	7.14	0	2	5.261	0	TRAPPC11	High	Peak Found
-4362102402614180000	Q13443	646211.1	148377.4	646211.1	80571.81	6	3	3	3	819	7.52	1.89	3	8.982	0	ADAM9	High	Peak Found

-2838998273344650000	Q14999	468626.9	105282.1	468626.9	57170.21	1	2	3	2	1698	5.87	0	2	6.097	0	CUL7	High	Peak Found
5402441647647120000	O75880	1536974	338994.5	1536974	184080.6	5	2	2	2	301	8.88	1.68	2	4.174	0	SCO1	High	Peak Found
563058972091496000	P80723	3909571	849581.5	3909571	461339.3	42	4	5	4	227	4.63	11.93	4	24.529	0	BASP1	High	High
4637087941136750000	O15126	591179.6	128055	591179.6	69536.33	10	2	3	2	338	7.42	0	2	7.909	0	SCAMP1	High	Peak Found
-8144210320745120000	Q5T1J5	692009.2	149496.8	692009.2	81179.7	19	1	2	1	151	9.89	5.51	1	7.912	0	CHCHD2P9	High	Peak Found
4361926129006050000	Q9BSH4	562931.2	115326.7	562931.2	62624.62	10	2	2	2	297	8.13	4.95	2	8.362	0	TACO1	High	Peak Found
-381937601164646000	Q15257	6716823	1356409	6716823	736556.6	12	4	8	4	358	5.94	8.75	4	11.769	0	PPP2R4; PTPA	High	High
-562077513670	P61764	1821641	363563	1821641	197421.8	8	4	4	4	594	6.96	4.15	4	12.521	0	STXBP1	High	Peak Found

1760000																		
2863197479310680000	Q96EK6	866018.4	157054.7	866018.4	85283.73	19	3	4	3	184	7.99	3.54	3	9.561	0	GNPNAT1	High	Peak Found
-7226470696601850000	Q9H0A8	167373.6	29764.9	167373.6	16162.92	10	2	2	2	199	7.31	1.88	2	5.169	0	COMMD4	High	Peak Found
34707929049350500	P51151	2574306	456352	2574306	247808	12	2	5	2	201	5.47	7.47	2	7.643	0	RAB9A	High	High
-6002739480164060000	Q14137	1222248	216531	1222248	117580.6	7	4	5	4	746	6.19	6.21	4	16.143	0	BOP1	High	High
2950616405929120000	P54802	619366.2	108976	619366.2	59176.07	6	3	3	3	743	6.65	1.84	3	7.129	0	NAGLU	High	High
8206347650273110000	Q9H7C9	1092088	191179.1	1092088	103814	20	2	3	2	122	8.46	2.2	2	5.637	0	AAMDC	High	High
3728877322360880000	P62854	1151378	187925	1151378	102046.9	23	2	2	2	115	11	2.09	2	5.406	0	RPS26; LOC101929876; RPS26P25	High	Peak Found

6821127370945390000	Q5JVF3	471202	74651.68	471202	40537.31	8	2	2	2	399	8.53	4.39	2	10.215	0	PCID2	High	Peak Found
3530888468156290000	O00541	13737671	2122154	13737671	1152371	14	8	13	8	588	7.33	9.1	8	21.227	0	PES1	High	High
2580326738670540000	O60341	1669212	257162.6	1669212	139644.3	5	4	5	4	852	6.52	2.14	4	11.326	0	KDM1A	High	Peak Found
-6082008338753530000	O15056	531108.9	81012.6	531108.9	43991.42	2	3	3	3	1496	7.34	1.81	3	5.012	0	SYNJ2	High	Peak Found
9124303556043210000	Q9P0S9	2701306	400654.6	2701306	217563.2	9	1	1	1	112	9.88	0	1	3.929	0	TMEM14C	High	Peak Found
6899043111177720000	Q15208	180494.7	26307.52	180494.7	14285.5	4	1	1	1	465	7.15	0	1	2.241	0.009	STK38	High	Peak Found
4573054572388010000	Q9BWU0	831268.2	119103.7	831268.2	64675.63	3	2	2	2	796	5.19	2.49	2	7.246	0	SLC4A1AP	High	Peak Found
-833212871997	Q8IY67	1360832	192175.6	1360832	104355.1	4	2	2	2	606	8.48	1.71	2	4.145	0	RAVER1	High	Peak Found

2750000																		
4696526329741060000	Q14344	442365.7	60895.17	442365.7	33067.26	10	4	9	2	377	8	6.76	4	11.449	0	GNA13	High	High
4962513167934130000	P49643	1343415	166598.3	1343415	90466.13	4	2	3	2	509	7.91	0	2	6.939	0	PRIM2	High	Peak Found
-768624002376497000	O60725	1034876	122117	1034876	66311.91	6	1	1	1	284	7.96	3.37	1	8.842	0	ICMT	High	Peak Found
3667791429140770000	Q9NUM4	1549398	169191.6	1549398	91874.3	8	2	2	2	274	6.99	1.71	2	4.932	0	TMEM106B	High	Peak Found
907467384912736000	Q92504	1371325	142133.9	1371325	77181.48	3	1	3	1	469	6.87	9.49	1	6.318	0	SLC39A7	High	High
-4265990204774030000	O75175	1230075	117282.5	1230075	63686.68	2	1	1	1	753	6.2	0	1	4.538	0	CNOT3	High	Peak Found
-1674970657433910000	P51159	599679.6	50970.4	599679.6	27677.92	11	2	3	2	221	5.22	0	2	5.727	0	RAB27A	High	Peak Found

1873273852874600000	Q709C8	3513670	298120	3513670	161884.9	1	5	6	5	3753	6.83	3.83	5	12.493	0	VPS13C	High	High
2422870535062940000	Q9P0J0	1319360	45413.93	1319360	24660.65	17	2	3	2	144	8.43	3.01	2	6.318	0	NDUFA13	High	Peak Found
-3796638279955900000	Q9NZ32	696720.4	16643.89	696720.4	9037.957	6	3	4	3	417	7.37	0	3	5.387	0	ACTR10	High	High
5357467251583520000	Q49AR2	54551838	555605.3	54551838	301704.5	6	2	2	2	442	4.78	0	2	4.978	0	C5orf22	High	Peak Found
